# β‑d‑Arabinofuranose-configured Cyclitol Aziridines as Selective, Brain-penetrant Covalent GBA2 Inhibitors

**DOI:** 10.1021/jacs.6c09754

**Published:** 2026-09-29

**Authors:** Rob F. Lammers, Qin Su, Laura Mazo, Wendy A. Offen, Roelof Ottenhoff, Mats J. Bulterman, Martijn van der Lienden, Maria J. Ferraz, Florian Küllmer, Zahid Ilhan, Hans van den Elst, Ingeborg van der Made, Cindy P. A. A. van Roomen, Anne de Jongh, Adrianus M. C. H. van den Nieuwendijk, Rolf G. Boot, Noam Zelcer, Gideon J. Davies, Carme Rovira, Herman S. Overkleeft, Johannes M. F. G. Aerts, Marta Artola

**Affiliations:** † 4496 Departments of Medical Biochemistry and Bio-organic Synthesis, Leiden Institute of Chemistry (LIC), Leiden University, P.O. Box 9502, 2300 RA Leiden, The Netherlands; ‡ Departament de Química Inorgànica i Orgànica (Secció de Química Orgànica) and Institut de Química Teòrica i Computacional (IQTCUB), 16724 Universitat de Barcelona, Martí i Franquès 1, 08028 Barcelona, Spain; § Department of Chemistry, York Structural Biology Laboratory, 8748 University of York, Heslington, York YO10 5DD, U.K.; ∥ Department of Medical Biochemistry, Amsterdam University Medical Center, location Academic Medical Center, 1234 University of Amsterdam, Amsterdam 1105AZ, The Netherlands; ⊥ Amsterdam Gastroenterology, Endocrinology, and Metabolism Institute, Amsterdam University Medical Center, Amsterdam 1105AZ, The Netherlands; # Amsterdam Cardiovascular Sciences Institute, Amsterdam University Medical Center, Amsterdam 1105AZ, The Netherlands; ¶ Fundació Catalana de Recerca i Estudis Avançats (ICREA), Passeig Lluís Companys 23, 08010 Barcelona, Spain

## Abstract

Neutral glucosylceramidase GBA2 is a retaining β-d-glucosidase involved in sphingolipid metabolism and implicated in neurological lysosomal storage disorders, yet the lack of highly selective chemical inhibitors has hindered efforts to disentangle its function from that of glucosylceramide synthase (GCS) and lysosomal glucosylceramidase (GBA1). Here, we report the development of the first brain-penetrant, covalent inhibitor that selectively inactivates GBA2 in *vivo*. Building on β-d-arabinofuranose-configured (β-d-*Araf*) cyclitol aziridine activity-based probes previously shown to covalently and selectively label GBA2, we designed and synthesized a focused library of electrophile-bearing cyclitols, including epoxides, cyclic sulfates, and a diverse set of *N*-alkyl aziridines. Biochemical evaluation of their inhibitory activity against GBA1, GBA2, GBA3 and GCS revealed that several *N*-alkyl aziridines are highly selective, nanomolar GBA2 inhibitors. Lead compounds also demonstrated metabolic stability in murine plasma, robust cellular activity and high selectivity in living cells, with no detectable GCS inhibition. Notably, octyl-alkyne aziridine **5** selectively inhibited GBA2 in mouse brain, representing the first covalent GBA2 inhibitor with demonstrated *in vivo* activity and brain permeability. Together, these results establish β-d-*Araf* cyclitol aziridine **5** as a versatile inhibitor for selective, covalent GBA2 inhibition, well suited to dissect GBA2 biology *in vivo*, particularly in neurological lysosomal storage disorders.

## Graphical abstract

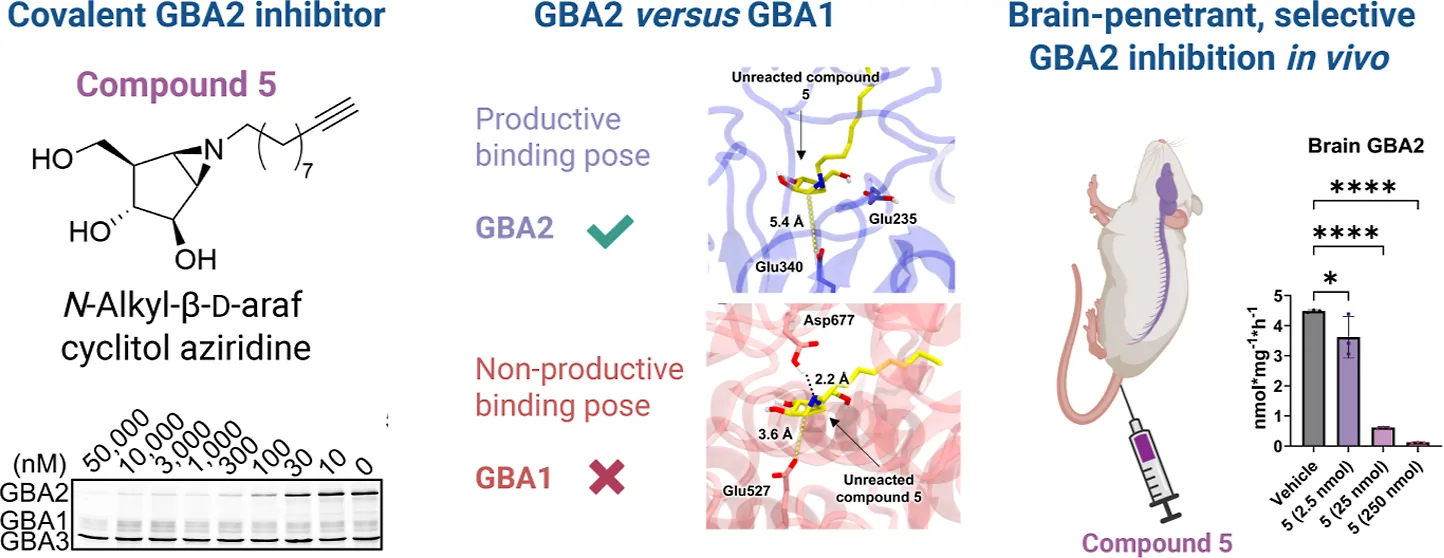

## Introduction

Neutral glucosylceramidase (GBA2, EC 3.2.1.45, CAZy GH116) is a retaining exo-β-d-glucosidase located at the plasma membrane and the membranes of the endoplasmic reticulum (ER) and Golgi apparatus.^1,2^ This enzyme consists of 927 amino acids, is encoded by the *GBA2* gene and has a molecular weight of approximately 105 kDa. GBA2 is a membrane-associated enzyme with its active site oriented toward the cytoplasm, where it processes glucosylceramide (GlcCer) into glucose and ceramide,^1,3^ a function that was previously attributed solely to the lysosomal glucocerebrosidase (GBA1 or GCase, EC 3.2.1.45, CAZy GH30, encoded by *GBA1*), the enzyme deficient in Gaucher disease (GD). Mutations in the *GBA1* gene have also been established as a significant genetic risk factor for Parkinson’s disease.^4,5^ In GBA1-deficient murine fibroblasts, increased levels of GBA2 and mRNA have been reported.^6^ Moreover, significantly higher spleen GlcCer levels were observed in *Gba1*/*Gba2* double knockout mice when compared to the sum of the single knockouts, suggesting cross-talk in GlcCer metabolism between the two enzymes.^6^

GBA2 hydrolyses glucosylceramide (GlcCer) via a Koshland double-displacement mechanism, in which Glu527 acts as the catalytic nucleophile and Asp677 serves as the catalytic acid/base residue (Figure 1A).^7^ Several GBA2 inhibitors have been reported, mainly iminosugar-based inhibitors which also inhibit glucosylceramide synthase (GCS).^8^ For instance, *N*-butyl-deoxynojirimycin (miglustat, also known as Zavesca) is a brain-penetrant iminosugar with dual inhibition activity against GBA2 (IC_50_ = 200 nm)^1^ and GCS (IC_50_ = 34.4 μm).^9^ Miglustat is approved as a GCS inhibitor for the treatment of type 1 GD, and it is also used to alleviate neurological symptoms of Niemann-Pick type C (NPC) disease, where it has demonstrated anti-inflammatory effects.^10,11^ NPC is a neurodegenerative lysosomal storage disorder caused by loss-of-function mutations in the *NPC1* or *NPC2* genes, which encode essential proteins involved in cholesterol transport from the lysosomes. Iminosugar-based inhibition of GBA2 (and possibly GCS) by miglustat and *N*-adamantanemethyloxypentyl-deoxynojirimycin (AMP-DNM)^12,13^ or through genetic knockout of *Gba2* ^12^ has been shown to increase lifespan in NPC mouse models. Treatment with iminosugars leads to an increase in GlcCer levels in the brain of NPC mice, which is paradoxical to the speculated substrate reduction mechanism of action of GCS inhibition. This suggests that the therapeutic benefit of iminosugar may instead rely on GBA2 inhibition, leading to elevated GlcCer in brain.^12,13^ In GD type 1 mouse models, genetic deletion of *GBA2* rescued disease phenotypes.^14^ In a mouse model of Sandhoff disease a neuropathic lysosomal storage disorder caused by mutations in the *HEXB* gene and resulting in GM2 ganglioside accumulation—treatment with iminosugar-based dual GCS/GBA2 inhibitors increased lifespan, and brain GlcCer levels were significantly elevated.^15^ Collectively, these results suggest that GBA2 inhibition might be a suitable therapeutic target to ameliorate multiple neurological LSDs.

**Figure 1:**
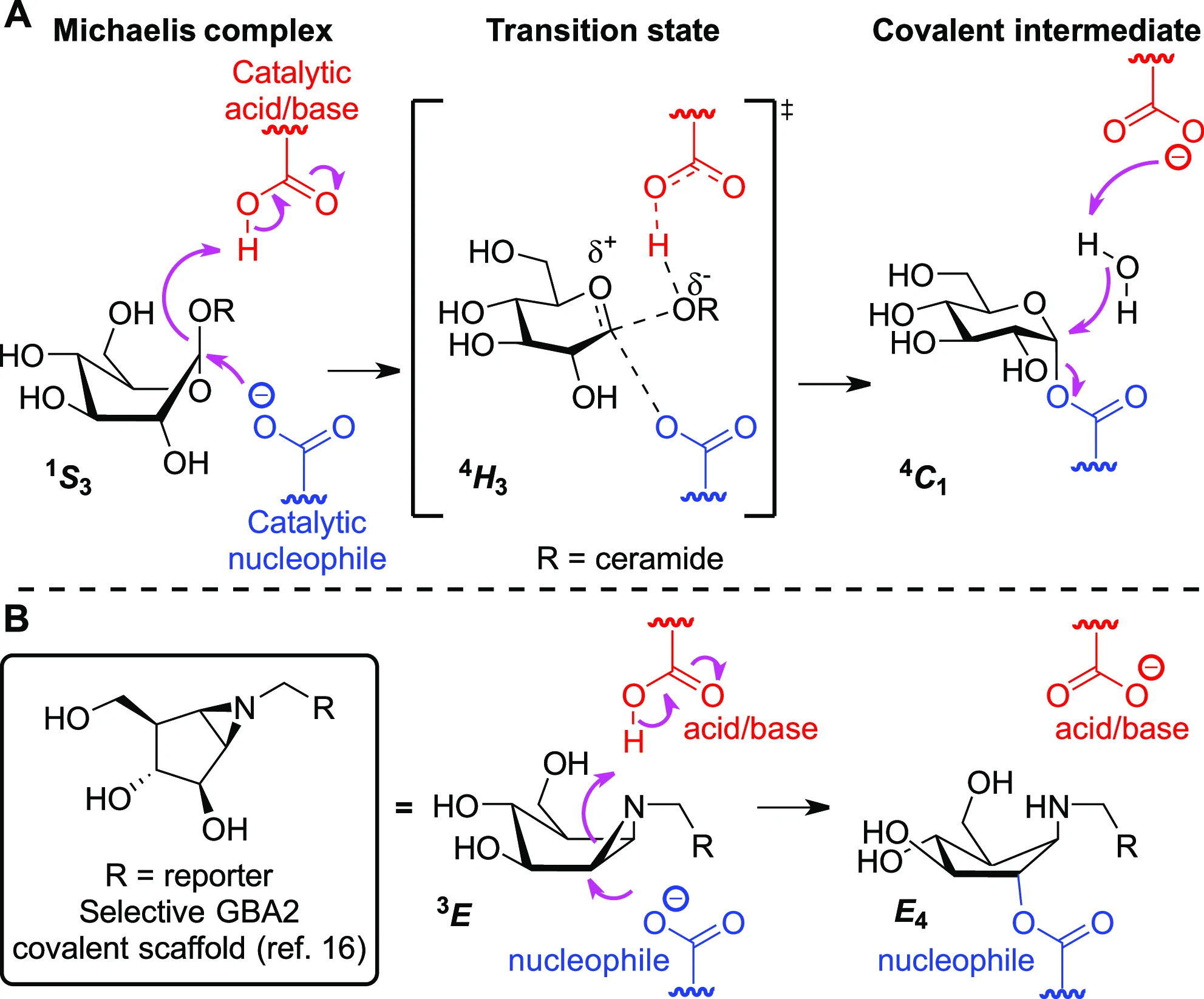
GBA2 reaction mechanism and inhibition by β-d-*Araf* cyclitol aziridines. (**A**) Conformational itinerary of the Koshland double displacement mechanism of retaining β-d-glucosidases. (**B**) β-d-*Araf* cyclitol aziridines adopt a ^3^ *E* conformation, mimicking the Michaelis complex ^1^ *S*_3_ conformation retaining β-d-glucosidase substrates adopt in the active site, and react covalently and irreversibly with GBA2.

The development of selective GCS and GBA2 inhibitors is essential to further dissect their interplay and clarify the mechanism of action underlying the therapeutic effects in NPC and Sandhoff disease.^12,15^ Selective GCS inhibitors are already available, most notably the non-brain penetrant eliglustat and the brain-permeable venglustat. GBA2-selective inhibitors that do not interfere with GCS activity have not been reported and would be highly advantageous. Recently, we identified β-d-arabinofuranose-configured (β-d-*Araf*) cyclitol aziridine-based probes that covalently and selectively inactivate and label GBA2 in cells (Figure 1B).^16^ We reasoned that the covalent mode-of-action of these probes could provide a strategy to selectively inhibit GBA2 without affecting GCS. In this work, we developed a focused library of GBA2 inhibitors derived from this β-d-*Araf* cyclitol aziridine scaffold with the aim of selectively inhibiting GBA2 *in vivo* (Figure 2). Notably, aziridine **5** showed selective GBA2 inhibition in brains of treated mice, representing the first reported selective covalent GBA2 inhibitor with demonstrated central activity.

**Figure 2:**
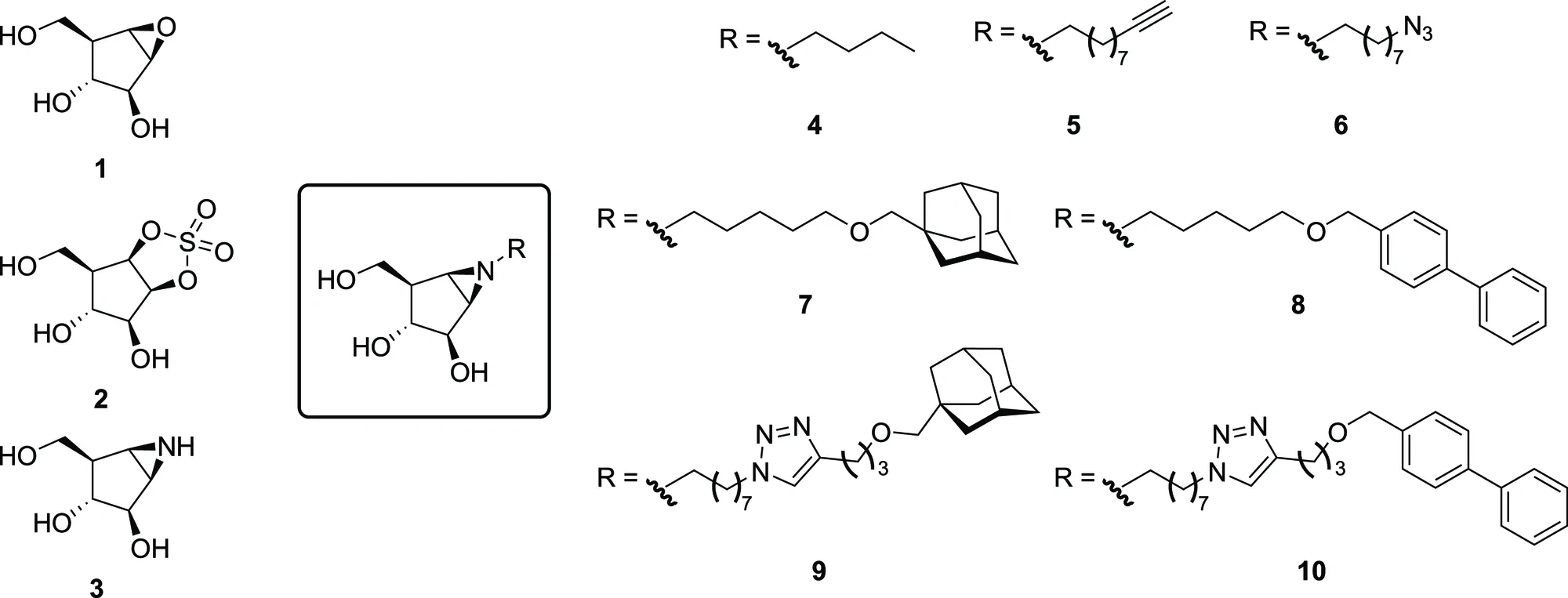
Chemical structures of β-d-*Araf* cyclitol epoxide **1**, cyclic sulfate **2**, aziridine **3** and *N*-alkyl aziridines **4−10**.

## Results

We designed a focused library of β-d-*Araf* cyclitols bearing the electrophilic warheads, epoxide **1**, cyclic sulfate **2**, and aziridine **3** (Figure 2). Inspired by our previously described GBA2-selective ABPs,^16^ and given the structural similarity between these aziridines and known iminosugar-based GBA2 inhibitors,^8^ we also synthesized *N*-butyl aziridine **4**, analogous to miglustat; alkyne **5** and azide **6** for functionalization by click chemistry; adamantyl aziridine **7** and biphenyl aziridine **8**, whose aglycons were inspired by known potent GBA2 inhibitors that also inhibit GCS,^17,18^ and adamantyl triazole **9** and biphenyl triazole **10**.

We based our synthesis on that of the previously reported synthesis of building block **3**,^19^ which we adapted to obtain the desired building block **16** (Scheme 1A). First, methyl-α-d-glucopyranose **11** was tosylated and benzoylated in a one-pot reaction to give protected sugar **12**,^19–21^ after which the primary tosylate was converted to iodide **13** under Finkelstein conditions.^19,21^ Iodide **13** was then subjected to Vasella fragmentation, followed by a 1,3-dipolar cycloaddition reaction with *N*-benzylhydroxylamine to afford **14**.^22,23^

**Scheme 1:**
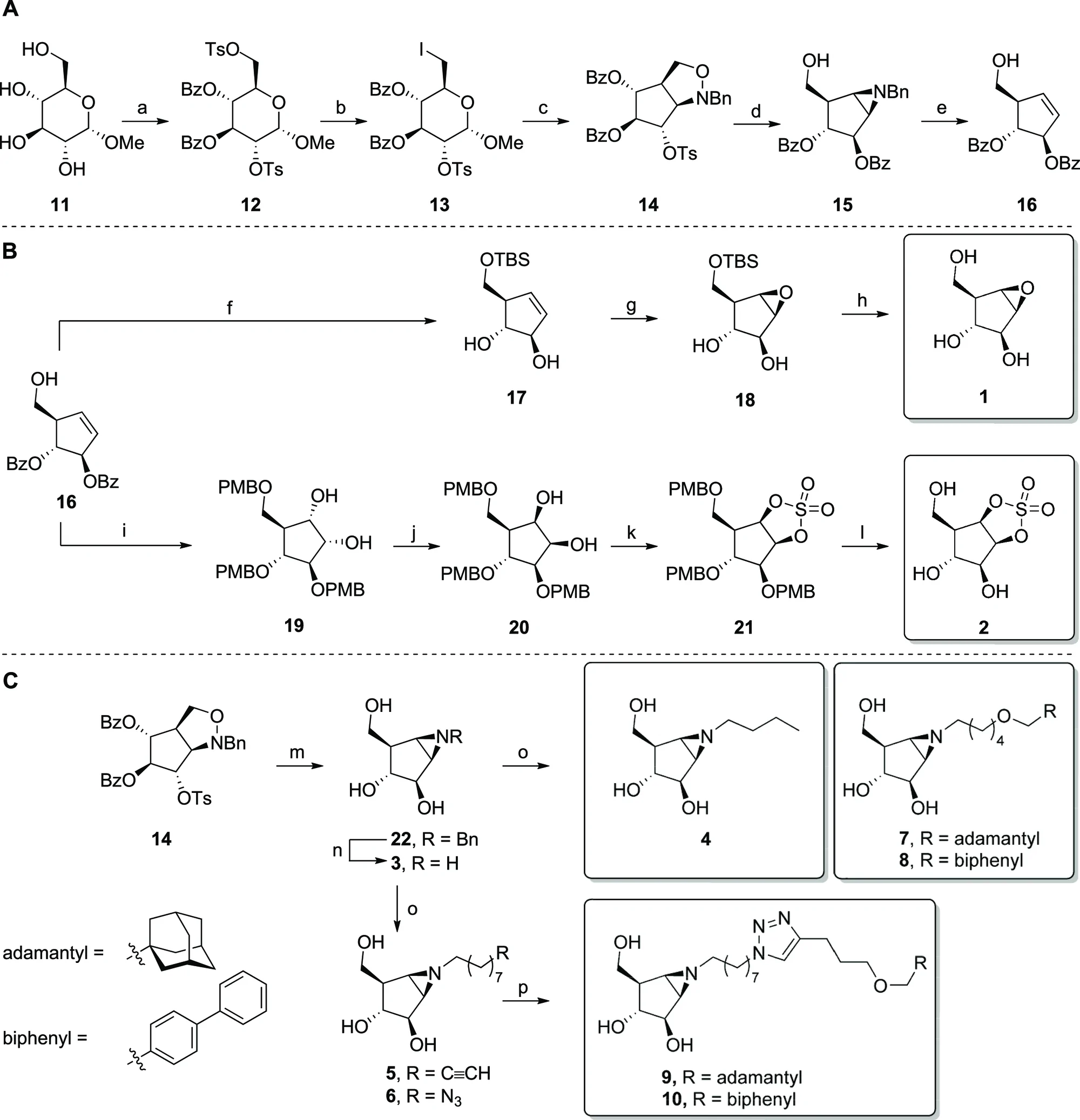
Synthesis of β-d-*Araf* Bearing an Epoxide, a Cyclic Sulfate or Functionalized Aziridines. Reagents and Conditions for (**A**) Synthesis of Intermediate **23**. (a) (i) TsCl, Pyr, rt, 24 h, (ii) BzCl, Pyr, rt, 24 h, Quant. Over 2 Steps; (b) NaI, Ac_2_O, Reflux, 4 h, 94%; (c) (i) Zn, EtOH, Reflux, 3 h, (ii) BnNHOH, Pyr/EtOH, 50 °C, 3 h, 70% Over 2 Steps; (d) Zn, AcOH, 35 °C, 80%; (e) *m-*CPBA, CH_2_Cl_2_, 80%. (**B**) Synthesis of Epoxide **1** and Cyclic Sulfate **2**. (f) (i) TBSCl, Imidazole, (ii) NaOMe, MeOH, 74% Over 2 Steps; (g) *m-*CPBA, NaHCO_3_, CH_2_Cl_2_, 85%; (h) TBAF, THF, 77%; (i) (i) NaOMe, MeOH, rt, 16 h, (ii) PMBCl, NaH, TBAI, DMF/THF, rt, 16 h, (iii) OsO_4_, NMO, Acetone/H_2_O, 16 h, 64% Over 3 Steps; (j) (i) Tf_2_O, Pyr, CH_2_Cl_2_, −10 °C, 90 min, (ii) CsOAc, DMF, −10 °C to rt, 16 h, (iii) NaOMe, MeOH, 48% Over 3 Steps; (k) SO_2_Cl_2_, Et_3_N, CH_2_Cl_2_, −65 °C, 16 h, 67%; (l) H_2_, Pd­(OH)_2_/C, rt, 3 h, 40%. (**C**) Synthesis of Aziridine **3** and its *N*-alkyl Analogues. (m) (i) Zn, AcOH, 35 °C, 4 h, (ii) NaOMe, MeOH, pH 10, rt, 3 h, 92% Over 2 Steps; (n) H_2_, Pd­(OH)_2_/C, H_2_O, rt, 2 h, 63%; (o) Alkyl Iodide, K_2_CO_3_, DMF, 80 °C, 18 h, 23% (**4**), 40% (**5**), 31% (**6**), 53% (**7**), 25% (**8**). (p) Alkyne, CuSO_4_, NaAsc, DMF, 51% (**9**) and 40% (**10**) From **6**

To prepare the epoxide derivative, we required the corresponding cyclopentene, which was obtained by oxidation of the corresponding aziridine precursor.^24^ First, the N–O bond was cleaved using activated zinc powder in acetic acid at 35 °C, which gave a significantly higher yield than reduction with Raney nickel.^19^ Subsequent addition of triethylamine promoted intramolecular displacement of the tosylate to form aziridine **15** without inducing hydrolysis or benzoate migration. Oxidation of aziridine **15** with *m*-CPBA afforded cyclopentene **16**, in line with the literature procedures (Scheme 1A).^20,24^ The primary alcohol in **16** was then protected as a TBS ether, and the secondary alcohols were deprotected to give cyclopentene **17**, which was oxidized to the corresponding epoxide **18** in excellent yield and stereoselectivity. Finally, global deprotection of **18** yielded epoxide **1** (Scheme 1B). To synthesize cyclic sulfate **2**, we first needed access to the β-configured diol, which proved challenging to obtain in a stereoselective fashion. We removed the benzoyl groups from compound **16**, after which all hydroxyls were protected with PMB groups, allowing for their later chemoselective removal in the presence of a cyclic sulfate. The resulting cyclopentene was oxidized using catalytic osmium tetroxide and *N*-methylmorpholine *N*-oxide affording the expected sterically less hindered α-diol **19**.^25^ Both alcohols were then simultaneously triflated and inverted using cesium acetate, after which the resulting β-diacetate was deacetylated to give β-diol **20**. The stereochemistry of β-diol **20** was confirmed by NOESY NMR experiments (Figure S2). Conversion of the diol to cyclic sulfate **21** was achieved using sulfonyl chloride. Attempts to oxidize the corresponding sulfite, generated by reacting the β-diol **20** with SOCl_2_ in CH_2_Cl_2_, were unsuccessful. Finally, cyclic sulfate **2** was obtained after PMB hydrogenolysis using Pearlman’s catalyst (Scheme 1B).

For the synthesis of functionalized aziridines, intermediate **14** was first subjected to N–O bond cleavage using activated zinc powder in acetic acid at 35 °C. Subsequent treatment with sodium methoxide in methanol afforded aziridine **22** in excellent yield. The benzyl group on the aziridine nitrogen was then removed by hydrogenolysis using Pearlman’s catalyst (Scheme 1C). The resulting deprotected aziridine **3** was *N*-alkylated with the corresponding alkyl iodides and potassium carbonate in dry DMF to provide *N*-butyl aziridine **4**, *N*-[dec-9-yn-1-yl] aziridine **5**, *N*-azidooctyl aziridine **6**, *N*-[5-(adamantan-1-yl-methoxy)-pentyl] aziridine **7** and *N*-[5-(biphenyl-4-yl-methoxy)-pentyl] aziridine **8**. *N*-Azidooctyl aziridine **6** was further functionalized via copper­(I)-catalyzed azide–alkyne cycloaddition to introduce adamantane- and biphenyl-containing moieties, affording aziridines **9** and **10**, respectively (Scheme 1C).

We then investigated the inhibitory activity of compounds **1**–**10** toward the human retaining β-glucosidases GBA1, GBA2, and GBA3. For this, we tested GBA1 inhibition with recombinant human GBA1 (rhGBA1, imiglucerase) and GBA2 or GBA3 in lysates of GBA1/GBA2 knockout HEK293T cells in which we overexpressed GBA2 or GBA3. These overexpression systems are required because of the low endogenous expression levels of GBA2 and GBA3.^16^

Inhibition was assessed using the fluorogenic substrate 4MU β-d-glucopyranoside (Figure 3A). In addition, we performed competitive activity-based protein profiling (cABPP) in lysates of HEK293T cells containing all β-glucosidases (endogenous GBA1 with overexpressed GBA2 and GBA3) to investigate their selectivity. Samples were preincubated with the inhibitor and subsequently treated with broad-spectrum retaining β-glucosidase ABP **23** (Figure S1), a fluorescent probe that efficiently labels all retaining β-glucosidases under these conditions.^26,27^ Labeled enzymes were separated by SDS-PAGE, followed by fluorescence scanning of the wet gel slabs and fluorescent quantification of the band intensities to obtain an in-gel fluorescence-based apparent IC_50_ value (Figure 3B,C). Epoxide **1**, cyclic sulfate **2**, and aziridine **3** were all inactive up to 100 μm against all human GBAs in the fluorogenic assays (Figure 3A). While *N*-butylaziridine **4** only inhibited GBA1 at high concentrations, octyl-alkyne **5** showed excellent inhibition potency and over 20x selectivity for GBA2 over GBA1, with apparent IC_50_ values of 319 nm for GBA2 and 8470 nm for rhGBA1 (Figure 3A), outperforming azido analogue **6**.^16^ Adamantyl aziridine **7** and biphenyl aziridine **8** exhibited similar activities toward both rhGBA1 and GBA2, with apparent IC_50_ values for GBA2 in the 140–220 nm range. Adamantyl triazole **9** and biphenyl triazole **10** both show comparable activities toward rhGBA1 and GBA2. When comparing these results from the fluorogenic substrate assays with the cABPP experiments in Figure 3B,C, we obtained very similar apparent IC_50_ values for GBA2, whereas higher apparent IC_50_ values were observed for GBA1 (Figure 3). This difference is likely due to the conditions used in the cABPP set up—pH 6.0 and absence of the additives Triton-X100 and sodium taurocholate—which are optimized for GBA2/3 inhibition but suboptimal for GBA1.

**Figure 3:**
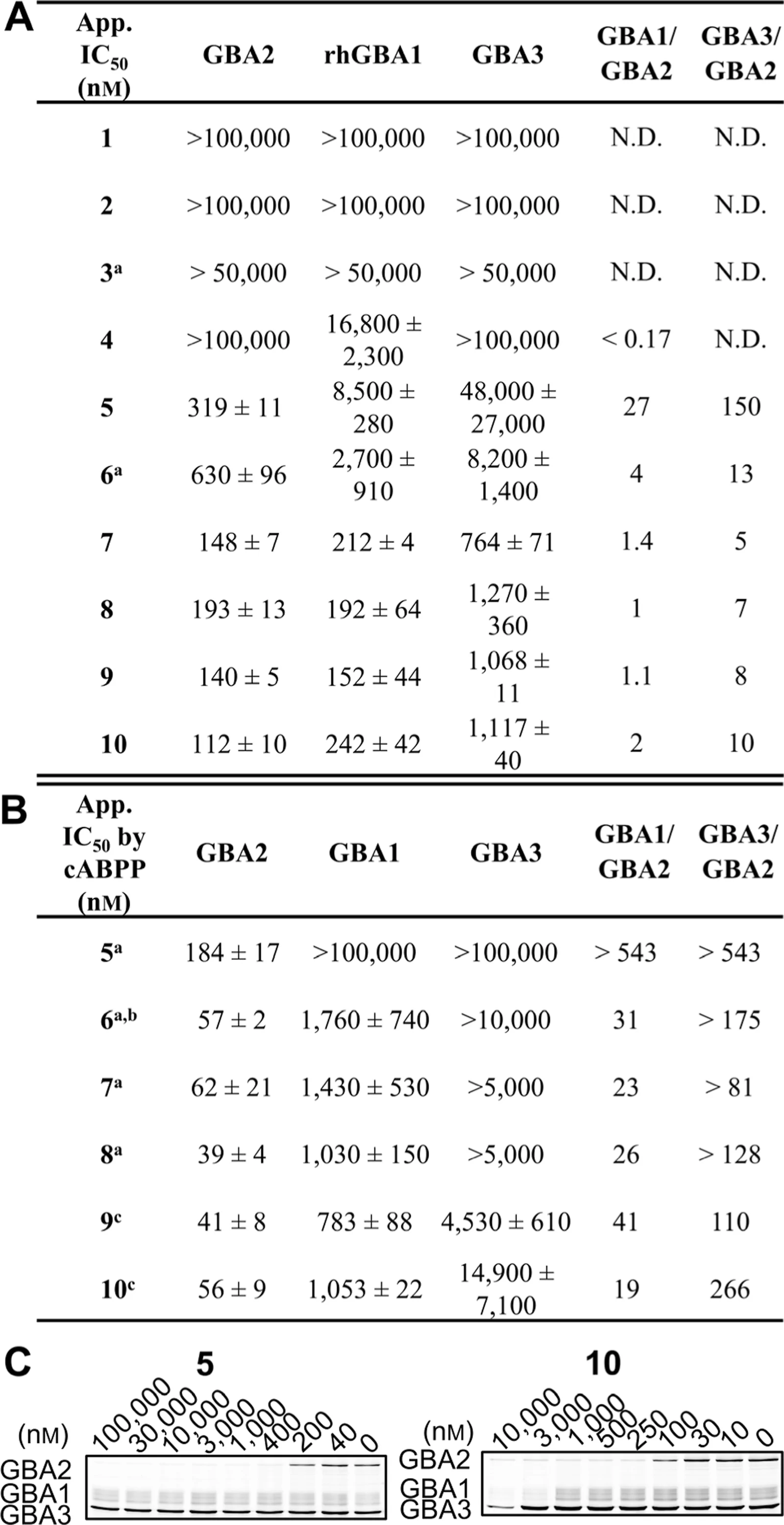
Inhibition of GBA1, GBA2 and GBA3 by β-d-*Araf* cyclitol aziridines. (**A**) Apparent half-maximal inhibitory concentration (IC_50_) values determined in a 4MU fluorogenic substrate assay using 4MU β-d-glucopyranoside. Enzyme sources: GBA2 = GBA1/GBA2 KO and GBA2 OE HEK293T cell lysate, rhGBA1 = imiglucerase (Cerezyme), GBA3 = GBA1/GBA2 KO and GBA3 OE HEK293T cell lysate. Error ranges = ± SD, *N* = 3 replicates. Individual data points are shown in Table S1. (**B**) Quantified apparent *in vitro* IC_50_ values in lysates of HEK293T cells overexpressing GBA2 and GBA3 as measured by competitive activity-based protein profiling using ABP **23** as broad-spectrum retaining β-glucosidase probe *in vitro*. ^a^Average of measured apparent IC_50_ values, *N* = 2 replicates. ^b^Apparent IC_50_ values from the literature.^16^ ^c^Error ranges = ± SD, *N* = 3 replicates. Individual data points are shown in Table S2. (**C**) Gel fluorescence images from the activity-based protein profiling assay using β-d-*Araf* cyclitol aziridines **5** and **10** as inhibitors and ABP **23** as broad-spectrum retaining β-glucosidase probe, *in vitro* in HEK293T cells overexpressing GBA2 and GBA3.

To assess the activity, selectivity, and cell permeability in living cells, *N*-alkylaziridines **5** (most selective) and **10** (apolar and relatively selective) were evaluated by cABPP in HEK293T cells expressing endogenous GBA1 and overexpressing GBA2 and GBA3. This cell-based system allows accurate quantification of enzyme activities that are otherwise lowly expressed in most cells (Figure 4A,B). In parallel, wild-type HEK293T cells were used to evaluate activity in a more physiologically relevant context (Figure 4B, right panel). Cells were incubated *in cellulo* with the inhibitors for 3 h, then collected, lysed, and subsequently incubated with **23** to label all residual active retaining β-glucosidases. As illustrated in Figure 4, both aziridines tested selectively inhibited GBA2 in living HEK293T cells overexpressing GBA2 and GBA3 exhibiting submicromolar apparent IC_50_ values against GBA2. For compound **5** we observed only partial inhibition of GBA1 at high concentrations (50 μm), while compound **10**, although it inhibits GBA2 at low nanomolar concentrations, also causes significant GBA1 inhibition at 1 μm concentration. In wild-type HEK293T cells (Figure 4B, right panel), the compounds retained excellent selectivity, although interpretation of these experiments is limited by the low GBA2 expression levels. Both compounds inhibited GBA2 at low nanomolar concentrations, with only partial inhibition of GBA1 detectable at 10–50 μm for compound **5** and ∼300 nm for compound **10**. Finally, we assessed their activity toward GCS, a known cotarget of iminosugars that can also inhibit GBA2, in living murine RAW 264.7 cells. Cells were treated with the inhibitors and 4-nitro-2,1,3-benzoxadiazole (NBD)- labeled ceramide, followed by lipid extraction, and glycosylation of NBD-ceramide was quantified by HPLC. Compound **5** displayed no detectable activity against GCS up to 100 μm, while compound **10** exhibited an apparent IC_50_ value of 10 μm for GCS (Figure 4A).

**Figure 4:**
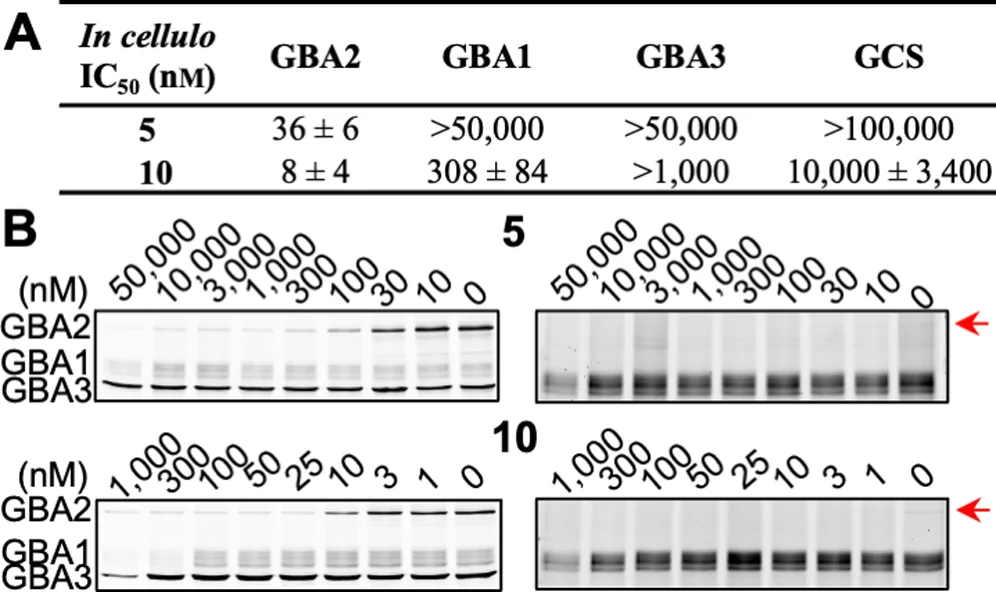
Inhibition of GBA1, GBA2, GBA3, and GCS by β-d-*Araf* cyclitol aziridines in living cells, as measured by competitive activity-based protein profiling using ABP **23** as broad-spectrum probe for retaining β-glucosidases (GBA1/2/3), and by an NBD-ceramide based fluorescence assay for GCS. (**A**) Quantified apparent IC_50_ values in GBA2/GBA3 OE HEK293T cells (GBA1/2/3) or RAW 264.7 cells (GCS). Individual data points are shown in Table S3. (**B**) Fluorescent gel images from activity-based protein profiling assays with β-d-*Araf* cyclitol aziridines as inhibitors and ABP **23** as broad-spectrum probe for retaining β-glucosidases in living GBA2/GBA3 OE HEK293T cells (left panel) and wild-type HEK293T cells (right panel). ABP-modified GBA2 is indicated by a red arrow.

Since compound **5** demonstrated significantly better GBA2 selectivity compared to **10**, we next investigated its ability to inhibit GBA2 in living mice. Of note, human and murine GBA2 share a high degree of sequence homology (∼87% identity),^28^ particularly within the catalytic domain and active-site residues. Compound **5** proved stable in mouse plasma (Figure S5), and exhibits significantly faster inactivation kinetics toward GBA2 than toward GBA1 (Figure S10). The compound was administered intravenously, and after 3 h, the mice were sacrificed. Mice were treated with 0, 2.5, 25, or 250 nmol of aziridine **5** (0, 0.035, 0.35, or 3.5 mg·kg^–1^, respectively). This time point was adapted from our previously reported *in vivo* study^29^ in which mice were sacrificed 2 h after compound administration. Relevant tissues were then collected and homogenized for analysis. Residual GBA1 and GBA2 activities were assessed both by cABPP using ABP **23** (Figure 5A) and fluorogenic substrate assays (Figure 5B), while residual GCS activity was evaluated using an NBD-ceramide fluorescence assay. Both methods revealed a clear dose-dependent inhibition of GBA2, with near-complete inhibition at 25 nmol in brain, liver, kidney and spleen. No significant inhibition of GBA1 or GCS was observed under any tested conditions (Figures 5 and S8).

**Figure 5:**
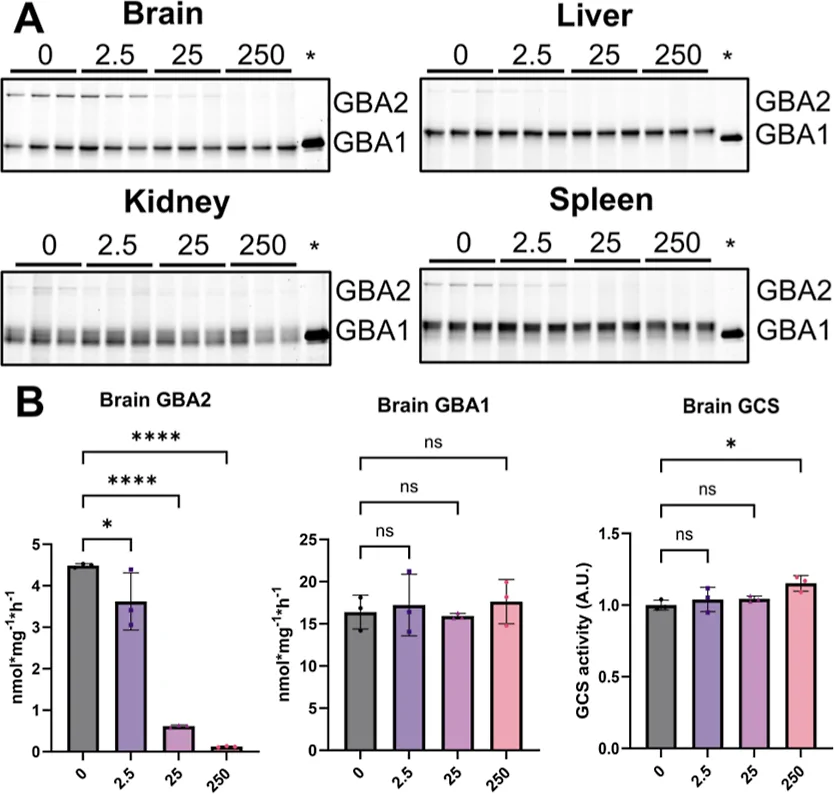
*In vivo* inhibition of GBA2 in mice by *N*-alkyl aziridine **5**. Mice were treated intravenously with 0, 2.5, 25, or 250 nmol of compound **5** and sacrificed after 3 h. (**A**) Active retaining β-glucosidase levels in brain, liver, kidney, and spleen homogenates were quantified by treatment of the samples with broad-spectrum β-glucosidase ABP **23**, protein separation by SDS-PAGE and fluorescence scanning of the resulting wet gel slabs. *Recombinant human GBA1. (**B**) Quantified GBA2 and GBA1 enzyme activities using 4-MU-based fluorogenic substrate assays and quantified GCS enzyme activities using NBD-based fluorescence assays, in mouse brain homogenates.

To gain structural insight into GBA2 binding, a crystal structure of compound **5** in complex with *Tx*GH116 was obtained. *Tx*GH116 is a bacterial homologue from *Thermoanaerobacterium xylanolyticum* that shares homology with human GBA2, and for which several crystal complex structures have been reported, including two with aziridines.^8,16^ Our previously reported β-d-*Araf* ABPs selectively targeted the catalytic nucleophile Glu527 of GBA2, as labeling was completely abolished in the E527 mutant and E527/D677G double mutant, whereas the D677G mutant exhibited significantly reduced labeling.^16^ In addition to this conserved reactivity, structural overlay of human GBA2 and *Tx*GH116 reveals high degree of similarity between their active sites (Figures S12 and S13). The structure revealed that compound **5** reacts with the enzyme, forming a covalent bond between Glu441 and the C1 atom of the β-d-*Araf N*-alkylaziridine **5** (Figure 6A). The substituents on the cyclopentane ring **5** produce hydrogen bonding networks with the enzyme active site similar to those observed in the crystal structure of *Tx*GH116 with *N*-azido-octyl aziridine **6** (PDB: 8R1M),^16^ interacting with the side chains of Asp452, His507, Glu777, Arg786, and Arg792, as well as the carbonyl oxygen and side-chain hydroxyl oxygen of Thr591. In both structures, the secondary amine resulting from aziridine ring opening interacts with Asp593, forming a direct hydrogen bond to its side-chain oxygen, with an additional interaction via a water molecule hydrogen-bonded to the other side-chain oxygen of Asp593. A slight difference is observed in the orientation of the *N*-alkyl chains between the two structures: beyond the fourth methylene, the chains diverge and point in opposite directions toward the protein surface. Notably, no electron density was observed for the alkyne or azido groups in either structure, and only seven methylene units of the octyl chain could be modeled (of which only three and five carbon atoms are at full occupancy for **5** and **6**, respectively).

**Figure 6:**
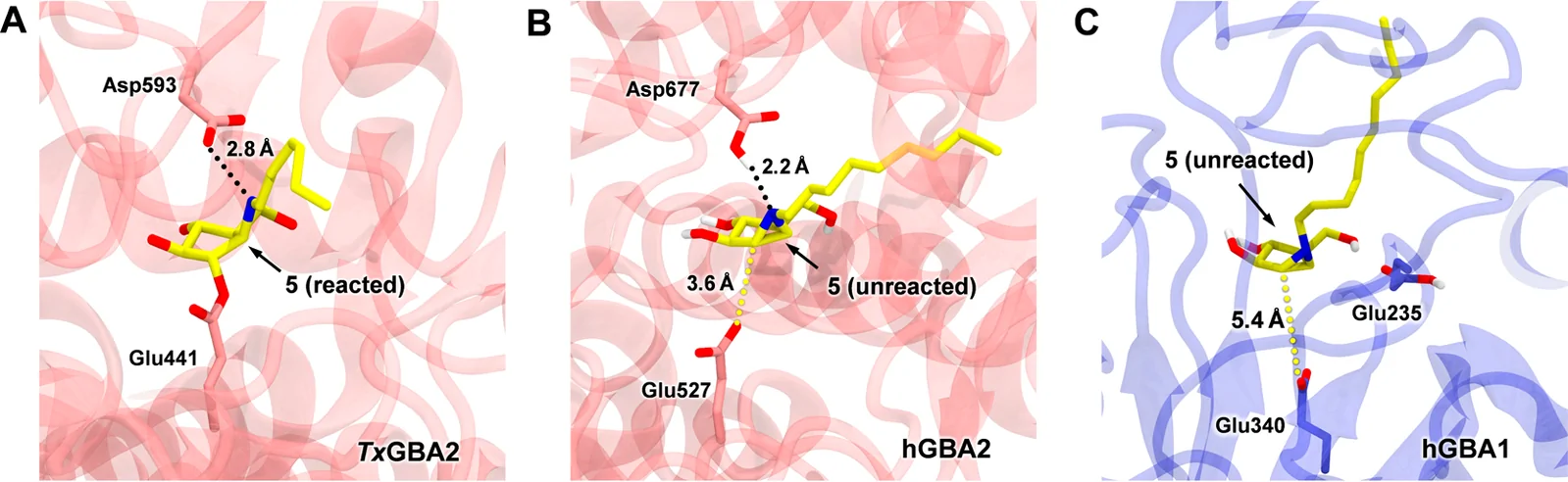
Structural basis for the selectivity of compound **5** for human GBA2 and its bacterial homologue over human GBA1. (**A**) Crystal structure of compound **5** bound to the bacterial GBA2 homologue *Tx*GH116. (**B**) Most populated binding pose of unreacted **5** obtained from MD simulations using the AlphaFold model of human GBA2. (**C**) Most populated binding pose of unreacted **5** obtained from MD simulations using the crystal structure of human GBA1. GBA2 and its bacterial homologue are shown in pink, whereas GBA1 is shown in blue. Yellow dotted lines indicate the distance of possible nucleophillic attack. Black dotted lines indicate the hydrogen bond interaction between the catalytic acid/base residue and the aziridine nitrogen atom. Because hydrogen atoms are not resolved in the X-ray structure (panel A) the corresponding distance was measured between the two heteroatoms. The reported distance values are averages over the MD simulations.

In parallel, docking and molecular dynamics (MD) simulations of compound **5** bound to hGBA2 and hGBA1 were performed to elucidate the molecular basis of its potency and selectivity (Figure 6B,C). The simulations indicate that **5** forms stable complexes with both hGBA2 and hGBA1 over the simulated time scale (1.5 μs). However, the conformational ensembles differ in their propensity to adopt reaction-competent geometries. In hGBA2, the aziridine warhead frequently samples prereactive (“near-attack”) conformations in which the catalytic nucleophile (Glu527) is favorably positioned and aligned for nucleophilic attack on the electrophilic center, with Asp677 suitably placed to assist charge stabilization and proton transfer during ring opening (Figure 6B). In contrast, in hGBA1 the inhibitor adopts a quite different binding pose, in which the electrophile is less accessible (longer nucleophilic attack distance) and misaligned with respect to the nucleophilic residue Glu340 (Figure 6C), thereby reducing the probability of nucleophilic attack. These observations suggest that the GBA2 selectivity of **5** arises from its greater tendency to adopt a reaction-competent binding mode in GBA2 than in GBA1.

To substantiate this model experimentally, kinetic parameters were determined for both enzymes. Measurements of enzymatic activity showed that GBA1 displays substantially higher turnover of the 4-MU substrate than GBA2 (*k*_cat_ = 24.0 ± 0.84 s^–1^ versus 0.23 ± 0.02 s^–1^, see Figure S9). Inhibition kinetics of compound **5** in GBA2 were consistent with a covalent irreversible mechanism, yielding *K*_i_ = 2.1 ± 0.9 μm and *k*_inact_ = 1.42 ± 0.72 min^–1^. For GBA1, determination of individual parameters was not feasible due to the high inhibitor concentrations required (higher than 250 μm), likely approaching solubility limits; however, a second-order rate constant (*k*_inact_/*K*_i_) of 3.14 ± 0.41 min^–1^ mm ^–1^ could be obtained. Notably, this ratio is markedly lower than that observed for GBA2 (663 ± 47 min^–1^ mm ^–1^), indicating a substantially reduced efficiency of covalent inactivation of GBA1 (Figure S10). Although the individual kinetic parameters could not be resolved for GBA1, the pronounced difference in *k*_inact_/*K*_i_ values is consistent with the MD-derived model, supporting the conclusion that selective inhibition arises from a larger propensity to form a productive, near-attack conformation in GBA2, in which the aglycon moiety plays a key role.

## Conclusion

In summary, we report the development of the first brain-penetrant, covalent inhibitor that selectively targets GBA2 *in vivo*. From a focused library of β-d-*Araf* cyclitol electrophiles, compound **5** emerges as a potent and highly selective inhibitor, validated across orthogonal platforms, including fluorogenic assays and cABPP. Importantly, this selectivity is maintained in living systems. *In cellulo* experiments demonstrated robust and selective inhibition of GBA2 over GBA1 and GBA3, with no detectable GCS inhibitory activity. Most notably, intravenous administration of compound **5** in mice resulted in clear, dose-dependent inhibition of GBA2 across tissues, including the brain, with near-complete target engagement achieved at moderate doses (0.35 or 3.5 mg·kg^–1^) 3 h post administration. GBA1 and GCS activities remained unaffected under all tested conditions, establishing an unprecedented level of selectivity *in vivo* over these enzymes. Beyond its potency and selectivity, compound **5** provides a versatile chemical biology tool to interrogate GBA2 function in complex biological systems. Its built-in click handle further expands its utility, enabling direct target engagement studies and proteomic profiling. In particular, it can be used to probe the downstream consequences of acute selective GBA2 inhibition *in cells* and *in vivo* by integrating transcriptomic profiling (RNA-seq) and lipidomic analyses to investigate how altered sphingolipid metabolism impacts inflammatory signaling pathways. Such approaches will help elucidate the relationship between GBA2 activity, lipid homeostasis and neuroinflammation. Mechanistically, kinetic analysis, together with structural studies of the bacterial GBA2 homologue *Tx*GH116 and molecular dynamics simulations based on the AlphaFold model of human GBA2, provide a coherent mechanistic rationale for the observed selectivity pointing to a preferential formation of reaction-competent binding geometries in GBA2 when compared to GBA1. These insights offer a foundation for the future design of selective covalent GBA2 inhibitors for preclinical studies.

## Supplementary Material



## Notes

The SI includes NMR spectra, experimental procedures, analytical and crystallographic data, biochemical data, whole uncropped gels and supporting figures (PDF)

## Abbreviations

ABP: activity-based probe
ABPP: activity-based protein profiling
ARCA: autosomal-recessive cerebellar ataxia
cABPP: competitive activity-based protein profiling
GBA1: lysosomal glucocerebrosidase
GBA2: nonlysosomal glucocerebrosidase
GCS: glucosylceramide synthase
GD: Gaucher disease
GlcCer: glucosylceramide
HSP: hereditary spastic paraplegia
LSD: lysosomal storage disorder
NBD: 4-nitro-2,1,3-benzoxadiazole
NPC: Niemann-Pick type C disease.
