# Supplementary material for: β‑d‑Arabinofuranose-configured Cyclitol Aziridines as Selective, Brain-penetrant Covalent GBA2 Inhibitors
Source: J Am Chem Soc. 2026 Sep 29;148(40):43072–80. doi: 10.1021/jacs.6c09754 (PMC13674267; doi:10.1021/jacs.6c09754)

Supporting information

β-D-Arabinofuranose-configured cyclitol aziridines as selective, brain-penetrant covalent GBA2 inhibitors

Rob F. Lammers,[a] Qin Su,[a] Laura Mazo,[b] Wendy A. Offen,[c] Roelof Ottenhoff,[d] Mats J. Bulterman,[a] Martijn van der Lienden,[a] Maria J. Ferraz,[a] Florian Küllmer,[a] Zahid Ilhan,[a] Hans van den Elst,[a] Ingeborg van der Made,[d] Cindy P.A.A. van Roomen,[d] Anne de Jongh,[a] Adrianus M.C.H. van den Nieuwendijk,[a] Rolf G. Boot,[a] Noam Zelcer,[d,e,f] Gideon J. Davies,[c] Carme Rovira,[b,g] Herman S. Overkleeft,[a] Johannes M.F.G. Aerts[a] and Marta Artola[a]*

[a]Departments of Medical Biochemistry and Bio-organic Synthesis, Leiden Institute of Chemistry (LIC), Leiden University, P.O. Box 9502, 2300 RA Leiden, The Netherlands.

[b]Departament de Química Inorgànica i Orgànica (Secció de Química Orgànica) and Institut de Química Teòrica i Computacional (IQTCUB), Universitat de Barcelona, Martí i Franquès 1, 08028 Barcelona, Spain.

[c]York Structural Biology Laboratory, Department of Chemistry, University of York, Heslington, York YO10 5DD, United Kingdom

[d]Department of Medical Biochemistry, Amsterdam University Medical Center, location Academic Medical Center, University of Amsterdam, Amsterdam 1105AZ, The Netherlands.

[e]Amsterdam Gastroenterology, Endocrinology, and Metabolism Institute, Amsterdam University Medical Center, Amsterdam 1105AZ, The Netherlands.

[f]Amsterdam Cardiovascular Sciences Institute, Amsterdam University Medical Center, Amsterdam 1105AZ, The Netherlands.

[g]Fundació Catalana de Recerca i Estudis Avançats (ICREA), Passeig Lluís Companys 23, 08010 Barcelona, Spain.

Table of contents

Experimental procedures **4**
Biochemistry 4
General materials 4
Generation of cells genetically modified for β-glucosidase expression 4
Cell lysis 4
4MU fluorogenic assays for apparent IC_{50} determination 4
In cellulo GCS IC_{50} measurement 5
Bligh and Dyer lipid extraction 5
HPLC analysis of NBD-labeled lipids 6
Labeling of β-glucosidases by activity-based protein profiling (ABPP) 6
β-Glucosidase inactivation visualized by competitive ABPP with SDS-PAGE 6
Plasma metabolic stability 7
In vivo mice experiments 8
Inhibition kinetics 9
Expression, purification and crystallization 10
Data collection and structure refinement 10
Molecular dynamics simulations 11
Synthetic procedures **11**
Supporting figures **24**
Figure S1. Chemical structures of compounds used. 24
Figure S2. Determination of stereochemistry of diol **20**. 25
Figure S3. Fitting curves of *in vitro* β-glucosidase IC_{50} measurement using fluorogenic substrates. 26
Table S1. Individual data points of *in vitro* apparent IC_{50} values obtained using fluorogenic substrates. 27
Figure S4. Fitting curves of quantified IC_{50} of *in vitro* cABPP. 28
Table S2. Individual data points of *in vitro* apparent IC_{50} values obtained using cABPP. 29
Figure S5. *In vitro* plasma metabolic stability of compound **5**. 29
Figure S6. Fitting curves of *in cellulo* competitive ABPP assays. 30
Table S3. Individual data points of *in cellulo* apparent IC_{50} values obtained using cABPP. 30
Figure S7. Fitting curves of *in cellulo* IC_{50} values against GCS. 31
Table S4. Individual data points of *in cellulo* apparent IC_{50} values against GCS. 31
Figure S8. Quantified enzyme activities in mice homogenates treated with compound **5**. 32
Figure S9. Michaelis Menten kinetics of GBA1 and GBA2. 33
Figure S10. Inhibition kinetics of compound **5**. 33
Figure S11. Omit electron density map (*F*o-*F*c) for aziridine and Glu441 side chain in *Tx*GH116-**5** structure. 34
Table S5. Data collection and refinement statistics for *Tx*GH116-**5** structure. 35
Figure S12. Structural overlay of human GBA2 and *Tx*GH116. 36

Figure S13. Sequence homology of human GBA2 and *Tx*GH116. 36
Figure S14. RMSD plots of protein backbone atoms along the MD simulation. 37
Figure S15. RMSF plots of protein backbone atoms along the MD simulation 37
Figure S16. Representative configurations of the two most populated clusters obtained from MD simulations of GBA1 in complex with compound **5**. 38
Figure S17. Representative configurations of the two most populated clusters obtained from MD simulations of GBA2 in complex with compound **5**. 38
Figure S18. Uncropped gels and CBB of *in vitro* cABPP. 39
Figure S19. Uncropped gels and CBB of *in cellulo* cABPP with HEK293T cells overexpressing GBA2/3. 41
Figure S20. Uncropped gels and CBB of *in cellulo* cABPP with WT HEK293T cells. 42
Figure S21. Uncropped gels and CBB of *in vivo* cABPP. 43
References **44**
NMR Spectra **47**
HRMS Spectra **96**
LC-MS Spectra (final compounds) **107**

Experimental procedures

Biochemistry

General Materials

Recombinant human GBA1 (rhGBA1, Imiglucerase) was kindly provided by Sanofi Genzyme (Genzyme Nederland, Naarden, The Netherlands). 4-Methylumbelliferyl-β-D-glucopyranoside substrates were purchased from Glycosynth (Warrington Cheshire, UK). Protein concentration was measured using the Pierce BCA assay kit (Thermo Fisher Scientific, Waltham, MA, USA). HEK293T (CRL-3216™) cells were purchased from ATCC (Manassas, VA, USA). HEK293T cells were cultured in DMEM medium (Sigma-Aldrich), supplied with 10% (v/v) FCS, 0.1% (w/v) penicillin/streptomycin and 1% (v/v) Glutamax, under 37 °C and 5% CO_2. Harvested cells (cell pellets), cell lysates and tissue homogenates not used directly were stored at –80 °C.

Generation of cells genetically modified for β-glucosidase expression

For overexpression of the different β-glucosidases, HEK293T cells with both endogenous GBA1 and GBA2 knockout (KO) were used, followed by overexpression (OE) of targeted β-glucosidases using the constructs as previously described.[1,2] For using "wildtype" HEK293T cells containing endogenous GBA1 and GBA2 to overexpress both GBA2 and GBA3, these were generated as previously described.[1,2]

Cell lysis

Cells were cultured as described above to 80-90% confluency, and were isolated, washed with Dulbecco′s phosphate buffered saline (PBS) and subsequently collected using the following method.[3] HEK293T cells were easily detached from the culture plate by trypsin treatment after which some DMEM medium was added. Cells were collected by pipetting and DMEM medium was removed by centrifuging. The cells were washed with cold PBS three times, after which they were centrifuged to remove PBS and then stored at –80 °C for further use or lysed in potassium phosphate buffer (K_2HPO_4-KH_2PO_4, 25 mM, pH 6.5, supplemented with 0.1% (v/v) Triton X-100 and protease inhibitor cocktail (EDTA-free, Roche, Basel, Switzerland)) by sonication with a Polytron PT 1300D sonicator (Kinematica, Luzern, Switzerland). After sonication, lysed mixtures were centrifuged at 10,000 x *g* for 3 min at 4 °C, and the supernatants, where the desired β-glucosidases are present, were used for the experiment.

4MU fluorogenic assays for apparent IC_{50} determination

4MU fluorogenic assays (using recombinant human rhGBA1 or lysates of HEK293T cells) were conducted in 96-well plates at 37 °C. Samples were diluted with McIlvaine buffer (150 mM) to a final volume of 25 μL, at the appropriate pH for each enzyme. Assays were performed by incubating the samples with 100 μL 4-methylumbelliferyl-β-D-glucopyranoside substrate diluted in McIlvaine buffer for a period of 30 min. Apparent IC_{50} values were determined by subtraction of the background (measured for incubations without enzyme) as previously described.[4] The substrate mixtures used for each enzyme were as follows: GBA1: 3.75 mM 4MU-β-D-glucopyranoside in McIlvaine buffer (150 mM, pH 5.2, supplemented with 0.2% (w/v) sodium taurocholate, 0.1% (v/v) Triton X-100,

0.1% (w/v) bovine serum albumin (BSA)); GBA2: 3.75 mM 4MU-β-D-glucopyranoside in McIlvaine buffer (150 mM, pH 5.8, supplemented with 0.1% (w/v) bovine serum albumin (BSA)); GBA3: 3.75 mM 4MU-β-D-glucopyranoside in McIlvaine buffer (150 mM, pH 6.0, supplemented with 0.1% (w/v) bovine serum albumin (BSA)). To determine apparent IC_{50}: For GBA1, 3.16 ng (53 fmol) of rhGBA1 (recombinant human GBA1, Imiglucerase) was prepared in 12.5 µL McIlvaine buffer (150 mM, pH 5.2, supplemented with 0.1% (v/v) Triton X-100, 0.2% (w/v) sodium taurocholate, and 0.1% (w/v) bovine serum albumin (BSA)). The enzyme was incubated with 12.5 µL of inhibitors diluted in McIlvaine buffer (150 mM, pH 5.2) at 37 °C for 30 min. For GBA2, lysates of GBA1/GBA2 KO HEK293T cells with GBA2 overexpression were prepared in 12.5 µL McIlvaine buffer (150 mM, pH 5.8), and incubated with 12.5 µL of inhibitors diluted in McIlvaine buffer (150 mM, pH 5.8) at 37 °C for 30 min. For GBA3, lysates of GBA1/GBA2 KO HEK293T cells with GBA3 overexpression were prepared in 12.5 µL McIlvaine buffer (150 mM, pH 6.0), and incubated with 12.5 µL of inhibitors diluted in McIlvaine buffer (150 mM, pH 6.0) at 37 °C for 30 min. Subsequently, the samples were incubated with 100 µL 4-methylumbelliferyl-β-D-glucopyranoside substrates dissolved in McIlvaine buffer described above for a period of 30 min. After stopping the enzyme reaction with 200 µL of 1 M NaOH-glycine (pH 10.3), 4-methylumbelliferone fluorescence was measured with a fluorimeter LS55 (Perkin Elmer, Waltham, MA, USA) with λ_{EX} 366 nm and λ_{EM} 445 nm. Apparent IC_{50} values were calculated calculated using Graphpad Prism 9.0 with the Nonlinear regression (curve fit) - [Inhibitor] *vs.* response - Variable slope (four parameters) equation.

In cellulo **GCS IC_{50} measurement**

RAW264.7 cells were seeded at a density of 0.8 x 10^6 cells / mL in RPMI-1640 medium (Sigma, with 25 mM HEPES, 5% FCS, glutamax and penicillin-streptomycin) in 12-wells plates (Greiner Bio-one) and grown until fully confluent. The cells were washed once in PBS, after which 0.5 mL of RPMI-1640 medium (with 50 mM HEPES and 300 µM conduritol B epoxide (CBE)) was added. The cells were incubated with the CBE-containing medium for 60 minutes at 37 °C and 5% CO_2 to inhibit GBA1 activity. Then, 5 µL inhibitor, at the appropriate dilution, and 0.8 nmol of C6-NBD-Cer/BSA complex were added to each well, and the cells were incubated (37 °C, 5% CO_2) for 1 hour. The incubation was terminated by three washing cycles with room temperature PBS. 120 µL milliQ-H_2O was added to each well, and the plates were stored at −20 °C or used directly. Lipid extraction was performed according to the Bligh and Dyer method (see below), and lipids were subsequently analyzed by HPLC. Relative activities of these cell lines were determined as a ratio of the area under the curve of the NBD-GlcCer peak over the total area under the curve of all NBD-lipids (NBD-Cer, NBD-GlcCer and NBD-sphingomyelin), expressed as arbitrary units.

Bligh and Dyer lipid extraction

RAW264.7 cells were harvested by scraping while kept on ice. To 100 µL homogenate, 600 µL of 2:1 methanol/chloroform (v/v) mix (Biosolve, HPLC grade) was added. The samples were shaken for 15 minutes and centrifuged for 10 minutes at maximum speed (16,000 x *g*) to remove proteins. To the supernatant, 200 µL chloroform (Biosolve, HPLC grade) and 260 µL H_2O were added. The samples were shaken for 5 minutes and centrifuged for 3

minutes at 16,000 x *g* to induce phase separation. The lower phase was collected. 400 µL chloroform was added to the upper phase, after which the samples were shaken for 5 minutes and centrifuged for 3 minutes at 16,000 x *g*. The lower phase was collected and combined with the lower phase from the previous cycle, and the lipids were dried in a heating block set to 45 °C under a stream of N_2. After fully dried, the lipids were resuspended in 100 µL methanol (Biosolve, HPLC grade), shaken for 15 minutes, and placed in an ultrasonication bath for 30 seconds to ensure all lipids were fully dissolved. The samples were centrifuged for 10 minutes at 16,000 x *g* to pellet any undissolved particles. 90 µL supernatant was transferred to MS vials with inserts and analyzed by HPLC.

HPLC analysis of NBD-labeled lipids

HPLC analysis was performed on a Thermo Fisher Ultimate 3000 Dionex UHPLC system with a reversed-phase C18 3 µm column (BDS-Hypersil 120A), which was kept at a temperature of 22 °C. Chromeleon version 7.2 was used as the operating system. Samples were separated by isocratic elution in 85:15 MeOH/H_2O for 15 minutes at a flow rate of 1 mL/min. To confirm the retention time of the measured peaks, a standards solution containing NBD-ceramide and NBD-glucosylceramide was measured prior to every run.

Labeling of β-glucosidases by activity-based protein profiling (ABPP)

β-Glucosidases were incubated with excess of fluorescent broad-spectrum β-glucosidase ABP **23** at 37 °C for 30 min. The total sample volume was 20–40 µL with 0.5–1% DMSO concentration in appropriate pH McIlvaine buffer (150 mM, at pH 6.0). After incubation with the ABP **23**, proteins were denatured by boiling the samples with 5× Laemmli buffer (50% (v/v) 1 M Tris-HCl, pH 6.8, 50% (v/v) 100% glycerol, 10% (w/v) DTT, 10% (w/v) SDS, 0.01% (w/v) bromophenol blue, diluted into 1x for use) for 5 min at 98 °C, and separated by electrophoresis on 10% SDS-PAGE gels run at 90 V for 1.0-1.5 h. Wet slab-gels were imaged using the Typhoon FLA 9500 scanner (GE Healthcare, Eindhoven, The Netherlands) or a ChemiDocTM MP Imaging System (Bio-Rad, Veenendaal, The Netherlands) at λ_{EX} 635 nm and $\lambda_{EM} \geq$ 665 nm for Cy5 fluorescence. Afterwards, wet slab-gels were stained by Coomassie brilliant blue (CBB) G250 or R250 for total protein staining and scanned with a ChemiDocTM MP Imaging System.

β-Glucosidase inactivation visualized by competitive ABPP with SDS-PAGE

In general, samples containing GBA1, GBA2 or GBA3 (see the exact constitution of these samples in the described case) were first incubated (*in vitro* or *in cellulo*) with the inhibitors for 30 min, subsequently, the fluorescent readout ABP **23** was added and incubated another 30 min with the mixture to reveal the residual active β-glucosidase enzymes. The total sample volume was 20–40 µL with 0.5–1% DMSO concentration in appropriate pH McIlvaine buffer (150 mM).

For *in vitro* β-glucosidase inactivation, cell lysates containing β-glucosidases were first incubated with inactivators *in vitro* for 30 min, after which a fluorescent readout ABP was added to reveal the residual active β-glucosidases and samples were incubated for 30 min at 37 °C. After that, proteins were denatured by boiling the samples with 5× Laemmli buffer (50% (v/v) 1 M Tris-HCl, pH 6.8, 50% (v/v) 100% glycerol, 10% (w/v) DTT, 10%

(w/v) SDS, 0.01% (w/v) bromophenol blue, diluted into 1x for use) for 5 min at 98 °C, and separated by electrophoresis on 10% SDS-PAGE gels run at 90 V for 1.0-1.5 h. Wet slab-gels were imaged using the Typhoon FLA 9500 scanner (GE Healthcare, Eindhoven, The Netherlands) or a ChemiDocTM MP Imaging System (Bio-Rad, Veenendaal, The Netherlands) at λ_{EX} 635 nm and λ_{EM} ≥ 665 nm for Cy5 fluorescence. Afterwards, wet slab-gels were stained by Coomassie brilliant blue (CBB) G250 or R250 for total protein staining and scanned with a ChemiDocTM MP Imaging.

For *in cellulo* β-glucosidase inhibition studies, intact living HEK293T cells expressing β-glucosidases that had grown to above 80% confluency were first incubated with varying concentrations of inactivators for described time at 37 °C under 5% CO_2. At indicated time points, cells were harvested and lysed as description in cell lysis. Subsequently, a fluorescent readout ABP **23** was added and samples were incubated for 30 min at 37 °C *in vitro*, followed by procedures described above for protein denaturation, SDS-PAGE and fluorescence scanning. Competitive ABPP quantified apparent IC_{50} values were determined according to the ABP-emitted fluorescence of the read out ABP quantified by ImageQuant (GE Healthcare, Chicago, IL, USA) or ImageJ and calculated with Graphpad Prism 9.0 using Analyze - Nonlinear regression (curve fit) – One phase association.

Plasma metabolic stability studies

Mouse plasma was thawed from –80 °C and used directly for the assay. An aliquot of 100 μL plasma in an Eppendorf tube was incubated in a water bath at 37 °C for 10 min. Then, 1 μM of compound (final DMSO concentration 0.25%) was added to the plasma. The Eppendorf was mixed at 1,500 rpm for 15 seconds, after which it was incubated at 37 °C with 300 rpm shaking. At 0, 5, 10, 15, 30, 60, 120 and 180 min, samples of 10 μL were taken and extracted by adding 200 μL of acetonitrile containing 30 nM internal standard (amitriptylin). Procaine hydrochloride or propantheline bromide was incubated alongside each batch of test compounds as positive control. The samples were centrifuged (14,000 x *g*, 15 min, 4 °C) to pellet to precipitated protein and the supernatant was transferred to an LC-MS tube with insert for LC-MS analyses. Reduction in inhibitor concentration was quantified by LC-MS/MS analysis by monitoring the ratio of the area under the curve (AUC) of the inhibitor at different time points over the AUC of the internal standard. For LC-MS/MS analysis, an Agilent 6475 LC/TQ coupled to an Agilent 1260 Infinity II autosampler, equipped with a C18 column (Macherey Nagel, EC 50/4.6 Nucleodur C18 Gravity, 3 μm particle size) equipped with buffers A: H_2O, B: acetonitrile (CH_3CN), C: 1% TFA in H_2O . Analytical runs were run for 8 minutes with a gradient of 0 → 90% buffer B while 10% of buffer C was used over the entire run. The first 2 minutes masses were not recorded. For MS/MS, the following parent/daughter pairs were used: **5** (m/z 282 → 55), amitryptilin (m/z 278 → 91), procaine hydrochloride (m/z 237 → 120) and propantheline bromide (m/z 368 → 181). Signal intensities were normalized to the external standard. Assay runs were invalidated if $t_{1/2}$ of the reference substrate varied more than two-fold from historical means. The maximum $t_{1/2}$ that could be reliably measured in the assay was determined by analyzing the variation in reference substrate replicates. Compounds were considered stable when the percentage remaining after 180 min exceeded 80%. Plasma stability was recorded in biological duplicates or triplicates.

***In vivo* mice experiments**

Experimental procedures were all approved by the appropriate ethics committee for animal experiments. C57BL/6J mice were obtained from Charles River and fed a commercially available lab diet (Teklad 2916; Inotiv). C57BL/6J mice were injected intravenously via tail vein with 100 µL PBS or PBS containing the inhibitor (DMSO content did not exceed 5%). After 3 h of administration, the mice were euthanised by intraperitoneal (i.p.) administration of 238 mg ketamine/kg mouse and 23.8 mg xylazine/kg mouse (in 0.9% physiological saline), and blood was collected. Then the mice were immediately perfused with PBS at 3 mL/min. Livers and other organs were rapidly excised and immediately frozen in liquid nitrogen unless stated otherwise and stored at –80 °C. Homogenates were made in 25 mM potassium phosphate buffer, pH 6.5, supplemented with 0.1% (v/v) Triton X-100 and protease inhibitor cocktail (EDTA-free, Roche, Basel, Switzerland). Protein concentrations were quantified by Thermo Fischer Pierce™ BCA protein assay and diluted with the same buffer.

cABPP in mice homogenized tissues:

Homogenized tissues were incubated with fluorescent readout ABP **23** for 30 min at 37 °C *in vitro*, followed by procedures described above for protein denaturation, SDS-PAGE and fluorescence scanning.

In vivo GBA1 activity measurements in mice homogenized tissues:

In vivo 4MU fluorogenic assays were conducted similarly to *in vitro* IC_{50} determinations as described above. First, the tissue homogenate (12.5 µg total protein in 12.5 µL buffer) was incubated with 12.5 µL McIlvaine buffer (150 mM, pH 5.2, supplemented with 0.2% (w/v) sodium taurocholate, 0.1% (v/v) Triton X-100, containing 0 or 2.0 µM GBA1 inhibitor **24**), for a period of 30 min. After 30 minutes, the samples were incubated the samples with 100 µL 4-methylumbelliferyl-β-D-glucopyranoside substrate diluted in McIlvaine buffer (150 mM, pH 5.2, supplemented with 0.2% (w/v) sodium taurocholate, 0.1% (v/v) Triton X-100 and 0.1% (w/v) bovine serum albumin (BSA)), for a period of 30 min. After stopping the enzyme reaction with 200 µL 1 M NaOH-glycine (pH 10.3), 4-methylumbelliferone fluorescence was measured with a fluorimeter LS55 (Perkin Elmer, Waltham, MA, USA) or CLARIOStar with λ_{EX} 366 nm and λ_{EM} 445 nm. GBA1 activity was quantified by subtracting background activity from samples incubated with GBA1 inhibitor **24** from samples incubated without GBA1 inhibitor **24** and division by a 1 nmol 4MU control.

In vivo GBA2 activity measurements in mice homogenized tissues:

In vivo 4MU fluorogenic assays were conducted similarly to *in vitro* IC_{50} determinations as described above. First, the tissue homogenate (50 µg total protein, 12.5 µL) was incubated with 12.5 µL McIlvaine buffer (300 mM, pH 5.8, containing 2.0 µM GBA1 inhibitor **24** and 0 or 5.0 µM GBA2 inhibitor **25**). After 30 minutes, the samples were incubated the samples with 100 µL 4-methylumbelliferyl-β-D-glucopyranoside substrate diluted in McIlvaine buffer (150 mM, pH 5.8, supplemented with 0.1% (w/v) bovine serum albumin (BSA)) for a period of 30 min. After stopping the enzyme reaction with 200 µL 1 M NaOH-glycine (pH 10.3), 4-methylumbelliferone fluorescence was measured with a fluorimeter LS55 (Perkin Elmer, Waltham, MA, USA) or CLARIOStar with λ_{EX} 366 nm and λ_{EM} 445 nm. GBA2 activity

was quantified by subtracting background activity from samples incubated with GBA2 inhibitor **25** from samples incubated without GBA2 inhibitor **25** and division by a 1 nmol 4MU control. GBA1 activity was suppressed in all samples by incubation with GBA1 inhibitor **24** to suppress background activity.

In vivo GCS activity measurements in mice homogenized tissues:

In vivo NBD substrate assays were conducted in Eppendorf tubes at 37 °C. The tissue homogenates (200 μg for brain and kidney; 100 μg for liver and spleen, 10 μL) were prepared in potassium phosphate buffer (25 mM, pH 6.5, supplemented with 0.1% (v/v) Triton X-100 and protease inhibitor cocktail (EDTA-free, Roche, Basel, Switzerland)). The master mixture used was as follows: NBD-ceramide supplemented with BSA (1 nmol, 10 μL), potassium phosphate buffer (500 mM, pH 7.5, 10 μL), UDP-glucose (100 mM in H_2O, 5 μL), $MgCl_2$ (25 mM in H_2O, 10 μL) with 250 μM conduritol B epoxide (CBE) and 50 nM of GBA2 inhibitor **26** to avoid hydrolysis of glucosylated NBD-Cer and the homogenate. The master mix was stored on ice to prevent enzymatic activity. The assay was performed by incubating the samples for 60 minutes. Then, 400 μL MeOH was added to stop the enzymatic reaction. Lipid extraction was performed according to the Bligh and Dyer method, and lipids were subsequently analyzed by HPLC. Relative activities of these cell lines were determined as a ratio of the area under the curve of the NBD-GlcCer peak over the total area under the curve of all NBD-lipids (NBD-Cer and NBD-GlcCer), expressed as arbitrary units.

Inhibition kinetics

Kinetics parameters for inhibitor **5** were determined following methods described in the literature.[5,6] Measurements were performed in a CLARIOStar Plus plate reader using λ_{EX} 366 nm and λ_{EM} 445 nm. Kinetic experiments were carried out in biological triplicates with technical duplicates. The resulting progress curves were fitted to a one-phase exponential association model to obtain k_{obs} values, which were plotted against inhibitor concentration. According to Michaelis-Menten analysis for irreversible inhibitors, these data were used to determine the K'_i , k_{inact} and their ratio k_{inact}/K'_i.[5] K_i and the ratio k_{inact}/K_i was then calculated using the equation $K'_i = K_i*(1+[S]/K_M)$.[5] The K_M and k_{cat} for each enzyme was determined by incubating the same enzyme concentration used in the kinetic experiments with a series of substrate concentrations for 10 minutes at 37 °C. Reactions were then quenched by addition of stop buffer and fluorescence was measured. The resulting data were fitted according to Michaelis-Menten equation to determine K_M. To calculate the k_{cat} of GBA2, the lysates used in the kinetic experiments, were incubated with 500 nM of ABP **23** in McIlvaine buffer (150 mM, pH 5.8) for 30 minutes and compared with labeling of rhGBA1 under the same conditions for quantification. Proteins were then denatured, separated, scanned and quantified as described above. For rhGBA1, $K_M = 0.88 \pm 0.08$ mM and $k_{cat} = 24.0 \pm 0.84$ s^{-1}. To calculate the concentration of rhGBA1, the molecular weight of 5.56×10^4 $g \cdot mol^{-1}$ was used. For hGBA2, $K_M = 0.155 \pm 0.048$ mM and $k_{cat} = 0.23 \pm 0.02$ s^{-1}. Results were processed and analyzed using GraphPad Prism 6.0.

Kinetics for GBA1 inhibition

Recombinant enzymes GBA1 (Cerezyme) was used for kinetic experiments. rhGBA1 was

diluted to 24.5 nM in McIlvaine buffer (150 mM, pH 5.2), supplemented with 0.2% (w/v) sodium taurocholate and 0.1% (v/v) triton X-100. To each well of a 96 wells plate, 25 µL of this enzyme solution was added. A series of inhibitor **5** concentrations (10 µL, in DMSO) was prepared in substrate mixture (3.75 mM β-D-4MU-Glc in McIlvaine buffer (150 mM, pH 5.2), supplemented with 0.2% (w/v) sodium taurocholate, 0.1% (v/v) Triton X-100 and 0.1% (w/v) BSA) and equilibrated at 37 °C. The *t* = 0 samples, 200 µL of stop buffer (1 M glycine/NaOH, pH 10.3) was added to the wells, followed by 100 µL of the substrate–inhibitor mixture. Reactions were initiated by addition of 100 µL of pre-warmed inhibitor–substrate mixture (37 °C) to the enzyme. At *t* = 1.0, 2.0, 3.0, 5.0 and 10.0 min, reactions were quenched by addition of 200 µL of stop buffer (1 M glycine/ NaOH, pH 10.3) to the [I + E + S] mixtures in the 96-well plate.

Kinetics for GBA2 inhibition

Pellets of HEK293T cells with overexpression (OE) GBA2 were lysed in McIlvaine buffer (150 mM, pH 5.8). The lysates were then incubated with 1 µM **27** for 30 minutes prior to the kinetic experiments. A series of inhibitor **5** concentrations (10 µL, in DMSO) was prepared in substrate mixture (3.75 mM β-D-4MU-Glc in McIlvaine buffer (150 mM, pH 5.8), supplemented with 0.1% (w/v) BSA) and equilibrated at 37 °C. To each well of a 96-well plate, 25 µL of this enzyme lysate was added. For *t* = 0 samples, 200 µL of stop buffer (1 M glycine/NaOH, pH 10.3) was added to the wells, followed by 100 µL of substrate–inhibitor mixture. Reactions were initiated by addition of 100 µL the pre-warmed inhibitor–substrate to the enzyme solution. At *t* = 1.0, 2.0, 3.0, 5.0 and 10.0 min, reactions were quenched by addition of 200 µL of stop buffer (1 M glycine/ NaOH, pH 10.3) to the [I + E + S] mixtures in the 96-well plate.

Expression, purification and crystallization

A construct consisting of *Tx*GH116Δ1-18 cloned into pET30a was transformed into *E. coli* strain BL21(DE3)GOLD, and *Tx*GH116 was expressed and purified using previously described methods,[7] except that the immobilized metal affinity chromatography step was performed on a HisTrap™ FF column charged with nickel and equilibrated with 20 mM sodium phosphate, 150 mM NaCl pH 7.4, and the protein was eluted with a gradient into 20 mM sodium phosphate, 150 mM NaCl, 0.5 M imidazole pH 7.5. *Tx*GH116 at 2 mg/mL in 20 mM Tris–HCl pH 8.0, 150 mM NaCl was crystallized by sitting drop vapour diffusion, using a drop comprised of a 1.75:1 volume ratio of protein to well solution, with the latter consisting of 22% (w/v) polyethylene glycol 3000, 200 mM ammonium sulfate, 0.1 M 2-(*N*-morpholino) ethanesulfonic acid pH 5.5. A solution of **5** at 10 mg/mL in 50% DMSO was diluted 10-fold in well solution and added to the crystallization drop in a 1:1 volume ratio, giving a final soak concentration of 0.5 mM **5**. Crystals were fished after 4 days into liquid nitrogen via a cryoprotectant comprised of the well solution components and 25% (v/v) ethylene glycol.

Data collection and structure refinement

Data were collected at beamline io3 at the Diamond Light Source (UK), processed with *DIALS*[8] and scaled using *AIMLESS*.[9] The structure was solved by molecular replacement using *Phaser*[10] with PDB entry 5BVU as the model. The structure was refined using

REFMAC5[11] interspersed with cycles of model improvement in *Coot*.[12] Crystallographic programs were run within the *CCP*4 suites using *CCP*4 Cloud.[13] Validation checks were performed using *Coot* and the wwPDB Validation server, before depositing the structure in the Protein Data Bank (PDB code: 30PV).

Molecular dynamics simulations

The initial structures of GBA1 and GBA2 were taken from PDB. For the GBA2 enzyme, the human computed structure model generated with AlphaFold (AlphaFold DB: AF_AFQ9HCG7F1) was used. The loops with the lowest model confidence (pLDDT ≤ 50) were not taken into account (from Met1 to Ser65, and from Ser899 to Glu927). For the GBA1 enzyme, an X-ray crystallographic structure (PDB ID: 6TJQ, resolution: 1.4 Å) deposited by R. J. Rowland *et al* was used,[14] which also corresponds to *Homo sapiens*. To build Michaelis complexes, compound **5** was introduced by docking using Boltz-2[15] and Protenix.[16] The protonation states of the charged amino acids (Asp, Glu, and His) were predicted using H++ software,[17] considering pH = 5.5. The corresponding acid/base residue was protonated manually (Asp677 in GBA2 and Glu235 in GBA1). Both systems were solvated using the AmberTools[18] utility tLeap, creating a cubic water box of 10 Å around the enzymes. 9 and 11 Cl^- ions were added to neutralize the charge for GBA2 and GBA1, respectively. MD simulations were carried out using the Amber suite of programs,[19] using the ff14SB[20] and TIP3P[21] force fields for the protein and water molecules. Compound **5** was parameterised using the GAFF force field.[22] The atomic partial charges used for the parameterisation were calculated at the HF/6-31G* level using Gaussian16.[23] The following simulation protocol was used. The energy was minimised in two steps (first solvent molecules, and then the entire system). Then, the system was slowly heated up until 300 K (in intervals of 100 K). The density was equilibrated in the NPT ensemble for 200 ps. Finally, three independent production runs were performed (500 ns each, 1.5 µs total) for both systems. The timestep of the production simulation was 2 fs, using the SHAKE algorithm.

Chemistry

General information

All anhydrous solvents were dried with 4 Å molecular sieves (MeOH was dried with 3 Å molecular sieves). Unless stated otherwise, solvents were removed under reduced pressure at 40 °C. Reactions were monitored by TLC analysis using Merck 25 DC aluminum foil 60 F_{254} with detection by UV (254 nm) or by spraying with a solution of $(NH_4)_6Mo_7O_{24}\cdot 4H_2O$ (25 g/L) and $(NH_4)_4Ce(SO_4)_4\cdot 2H_2O$ in 10% sulfuric acid or a solution of $KMnO_4$ (20 g/L) and K_2CO_3 (10 g/L) in water followed by charring at approximately 150 °C. Column chromatography was performed manually using either Baker or Screening Device silica gel 60 (0.04-0.063 mm) or a Biotage Isolera™ flash purification system using silica gel cartridges (Screening Device SilicaSep HP, particle size 15-40 µm, 60 Å) in the indicated solvents. High resolution mass spectra were recorded with Q-Exactive HF Orbitrap (Thermo Scientific) equipped with an electrospray ion source (ESI), injection of 2 µL of a 1 µM solution via Ultimate 3000 nano UPLC (Dionex) system, with an external calibration (Thermo Scientific). Source voltage of 3.5 kV, capillary temperature 275 °C, no

sheath gas, Resolution = 240.000 at m/z = 400. Mass range m/z = 160 – 2,000 or until a maximum of 6,000. Eluens used: MeCN:H_2O (1:1 v/v) supplemented with 0.1% formic acid. LC-MS analysis was performed on a LCQ Fleet (Thermo Scientific) ion-trap spectrometer (ESI+) coupled to a Vanquish HPLC system (Thermo Scientific) equipped with a C18 column (Macherey Nagel, EC 50/4.6 Nucleodur C18 Gravity, 3 μm particle size) equipped with buffers A: H_2O, B: acetonitrile (MeCN), C: 1% TFA. ^{1}H-NMR and ^{13}C-NMR spectra were recorded on Bruker AV-400, Bruker AV-500 or Bruker AV-850. Chemical shifts were given in ppm (δ) relative to TMS (0 ppm, ^{1}H-NMR), $CDCl_3$ (77.16 ppm, ^{13}C-NMR), MeOD-d_4 (3.31 ppm, ^{1}H-NMR and 49.00 ppm, ^{13}C-NMR) or D_2O (4.79 ppm, ^{1}H-NMR). Coupling constants are given in Hz. ^{13}C-NMR spectra are Attached Proton Test (APT) spectra with phase inverted (180 degrees). The following abbreviations are used to describe peak patterns when appropriate: s (singlet), d (doublet), t (triplet), q (quartet), quint (quintet), m (multiplet), h (hextet) and br (broad). 2D NMR experiments (HSQC, COSY) were carried out to assign protons and carbons of the new structures and numbering and assignation follows the general numbering shown in compound **3**. The following abbreviations were used for signal assignation: Ar (aromatic), C_q (quaternary carbon), Ts (*p*-toluenesulfonyl), Bz (benzoyl), Bn (benzyl), PMB (*p*-methoxybenzyl), TBS (*tert*-butyldimethylsilyl), TES (triethylsilyl).

Methyl 3,4-di-*O*-benzoyl-2,6-di-*O*-tosyl-α-D-glucopyranoside (12)

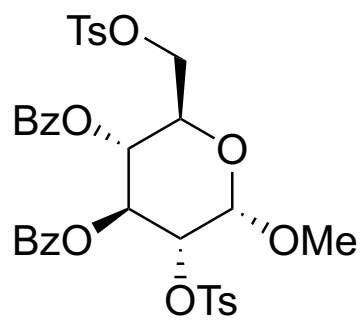

Orthogonally protected glucose intermediate **12** was synthesised according to literature procedure.[24,25] A flask under argon atmosphere was charged with methyl-α-d-glucopyranose (25.1 g, 129 mmol, 1.0 eq.) and anhydrous pyridine (500 mL). Tosyl chloride (50.6 g, 265 mmol, 2.1 eq.) was added and the mixture was stirred at room temperature for 24 hours. Then, benzoyl chloride (50 mL, 430 mmol, 3.3 eq.) was added and the mixture was stirred for 24 hours. The mixture was poured in ice and filtered. The solid residue was recrystallized from methanol to obtain the product as a white solid in quantitative yield (93.5 g, 129 mmol). Characterisation data are in agreement with the literature.[25] ^{1}H-NMR ($CDCl_3$, 400 MHz): δ 7.77 – 7.73 (m, 2H, 2xCH_{Ar} Bz), 7.70 (d, J = 8.4 Hz, 2H, 2xCH_{Ar} Ts), 7.63 – 7.59 (m, 2H, 2xCH_{Ar} Bz), 7.57 (d, J = 8.4 Hz, 2H, 2xCH_{Ar} Ts), 7.48 (td, J = 7.4, 1.4 Hz, 2H, 2xCH_{Ar} Bz), 7.34 – 7.25 (m, 4H, 2xCH_{Ar} Bz), 7.20 (d, J = 7.7 Hz, 2H, 2xCH Ts), 6.93 (d, J = 8.5 Hz, 2H, 2xCH_{Ar} Ts), 5.82 (t, J = 9.7 Hz, 1H, CH–3), 5.28 (t, J = 9.7 Hz, 1H, CH–4), 5.02 (d, J = 3.6 Hz, 1H, CH–1), 4.53 (dd, J = 10.0, 3.6 Hz, 1H, CH–2), 4.21 – 4.14 (m, 2H, CH–5, CH–6a), 4.06 (dd, J = 11.5, 6.0 Hz, 1H, CH–6b), 3.46 (s, 3H, CH_3–O), 2.33 (s, 3H, CH_3 Ts), 2.20 (s, 3H, CH_3 Ts). ^{13}C-NMR ($CDCl_3$, 101 MHz): δ 164.98, 164.97 (2xCO Bz), 145.1, 145.0 (2xCSO_2 Ts), 133.7, 133.2 (2xCH_{Ar}), 132.7, 132.3 (2x<u>C</u>CH_3 Ts), 129.9, 129.83, 129.81 (4xCH_{Ar}), 128.8 ($C_{q,Ar}$ Bz), 128.5 (CH_{Ar}), 128.4 ($C_{q,Ar}$), 128.2, 128.1, 127.7 (3xCH_{Ar}), 97.8 (C–1), 76.4 (C–2), 69.5 (C–3), 68.8 (C–4), 67.8 (C–6), 67.4 (C–5), 56.3 (CH_3O), 21.7 (2xCH_3 Ts). HRMS: calcd. for $[C_{35}H_{34}O_{12}S_2 + NH_4]^+$ 728.1830, found 728.1828.

3,4-Di-*O*-benzoyl-6-deoxy-6-iodo-2-*O*-tosyl-α-D-glucopyranoside (13)

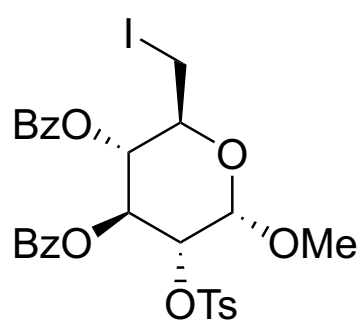

Iodine intermediate **13** was synthesised according to literature procedure.[24,25] A flask under argon atmosphere was charged with tosylate **12** (25.5 g, 35.9 mmol, 1.0 eq.) and acetic anhydride (60 mL). Sodium iodide (6.56 g, 43.7 mmol, 1.2 eq.) was added and the mixture

was refluxed for 4 hours. After cooling to room temperature, the mixture was filtered over Celite. The residue was dissolved in dichloromethane, washed with aqueous sodium thiosulphate, water, dried with $MgSO_4$, filtered, and concentrated *in vacuo*. The solid residue was recrystallized from methanol to yield the product as a white solid in 94% yield (22.5 g, 33.7 mmol). Characterisation data are in agreement with the literature.[25] ^{1}H-NMR ($CDCl_3$, 400 MHz): δ 7.86 (dd, *J* = 8.4, 1.4 Hz, 2H, 2xCH$_{Ar}$ Bz), 7.64 (dd, *J* = 8.4, 1.3 Hz, 2H, 2xCH$_{Ar}$ Bz), 7.60 (d, *J* = 8.4 Hz, 2H, 2xCH$_{Ar}$ Ts), 7.49 (q, *J* = 7.3 Hz, 2H, 2xCH$_{Ar}$ Bz), 7.34 (dd, *J* = 8.2, 7.3 Hz, 2H, 2xCH$_{Ar}$ Bz), 7.28 (dd, *J* = 8.7, 7.8 Hz, 2H, 2xCH$_{Ar}$ Bz), 6.95 (d, *J* = 7.9 Hz, 2H, 2xCH$_{Ar}$ Ts), 5.89 (t, *J* = 9.7 Hz, 1H, CH–3), 5.20 (t, *J* = 9.6 Hz, 1H, CH–4), 5.09 (d, *J* = 3.7 Hz, 1H, CH–1), 4.60 (dd, *J* = 10.1, 3.7 Hz, 1H, CH–2), 3.99 (ddd, *J* = 9.5, 8.2, 2.6 Hz, 1H, CH–5), 3.57 (s, 3H, CH_3O), 3.34 (dd, *J* = 11.0, 2.6 Hz, 1H, CH–6a), 3.19 (dd, *J* = 11.0, 8.4 Hz, 1H, CH–6b), 2.21 (s, 3H, CH_3 Ts). ^{13}C-NMR ($CDCl_3$, 101 MHz): δ 165.4, 165.0 (2xCO Bz), 145.0 (CSO_2 Ts), 133.8, 133.2 (2xCH$_{Ar}$), 132.9 (<u>C</u>CH_3 Ts), 130.0, 129.9 (3xCH$_{Ar}$), 128.9 (C$_{q,Ar}$ Bz), 128.6 (CH$_{Ar}$), 128.5 (C$_{q,Ar}$ Bz), 128.2, 127.7 (2xCH$_{Ar}$), 97.9 (C–1), 76.8 (C–2), 72.7 (C–4), 69.2 (C–3), 69.1 (C–5), 56.4 (CH_3O), 21.7 (CH_3 Ts), 3.7 (C–6). HRMS: calcd. for $[C_{28}H_{27}O_9SI + Na]^+$ 689.0313, found 689.0311.

(1*R*,5*R*)-6-Exo,7-endo-6,7-Bis(benzoyloxy)-*N*-benzyl-8-exo-8-toluene-*p*-sulfonyloxy-3-oxa-2-azabicyclo[3.3.0]octane (14)

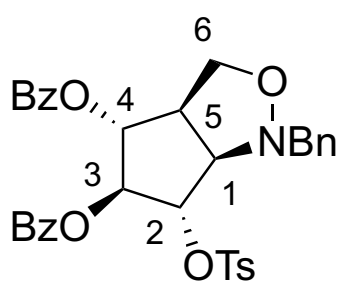

A flask under argon atmosphere was charged with iodide **13** (30.0 g, 45.0 mmol, 1.0 eq.) and 96% EtOH (900 mL). Zn dust (30.0 g, 459 mmol, 10 eq.) was added and the mixture was refluxed for 3 hours. The mixture was filtered through Celite and concentrated *in vacuo*. The resulting oil was dissolved in pyridine (240 mL) and 96% EtOH (60 mL) and put under argon atmosphere. *N*-benzylhydroxylamine hydrochloride (10.0 g, 62.7 mmol, 1.4 eq.) was added and the mixture was stirred at 50 °C for 3 hours. The mixture was then concentrated *in vacuo*. The resulting oil was recrystallized from methanol and rinsed with cold methanol to obtain the product as a white solid in 70% yield (19.2 g, 31.3 mmol). Characterisation data are in agreement with the literature.[26] ^{1}H-NMR ($CDCl_3$, 400 MHz): δ 7.98 – 7.95 (m, 2H, 2xCH$_{Ar}$ Bz), 7.88 (dd, *J* = 8.4, 1.4 Hz, 2H, 2xCH$_{Ar}$ Bz), 7.73 (d, *J* = 8.3 Hz, 2H, 2xCH$_{Ar}$ Ts), 7.59 – 7.51 (m, 2H, 2xCH$_{Ar}$ Bz), 7.44 – 7.37 (m, 4H, 4xCH$_{Ar}$ Bz), 7.36 – 7.27 (m, 5H, 5xCH$_{Ar}$ Bn), 7.07 (d, *J* = 7.9 Hz, 2H, 2xCH$_{Ar}$ Ts), 5.91 (t, *J* = 8.2 Hz, 1H, CH–3), 5.18 (dd, *J* = 8.1, 6.2 Hz, 1H, CH–4), 5.09 (dd, *J* = 8.3, 5.5 Hz, 1H, CH–2), 4.29 – 4.17 (m, 2H, CH_2–6), 3.95 (d, *J* = 13.5 Hz, 1H, C<u>H</u>H Bn), 3.90 – 3.80 (m, 2H, CH–1, CH<u>H</u> Bn), 3.25 (dtd, *J* = 10.0, 6.5, 3.7 Hz, 1H, CH–5), 2.20 (s, 3H, CH_3 Ts). ^{13}C-NMR ($CDCl_3$, 101 MHz): δ 166.2, 165.0 (2xCO Bz), 145.0 (CSO_2 Ts), 136.7 (C$_{q,Ar}$), 133.7, 133.49 (2xCH$_{Ar}$), 133.45 (C$_{q,Ar}$), 130.1, 130.0, 129.9 (3xCH$_{Ar}$), 129.10, 129.08 (2xC$_{q,Ar}$ Bz), 128.9, 128.7, 128.6, 128.5, 128.0, 127.7 (6xCH$_{Ar}$), 84.1 (C–2), 79.2 (C–4), 77.4 (C–3), 70.9 (C–1), 70.2 (C–6), 59.4 (CH_2 Bn), 50.3 (C–5), 21.7 (CH_3 Ts). HRMS: calcd. for $[C_{34}H_{31}NO_8S + H]^+$ 614.1843, found 614.1843.

***N*-Benzyl 2,3-*O*-dibenzoyl β-D-*Araf* cyclitol aziridine (15)**

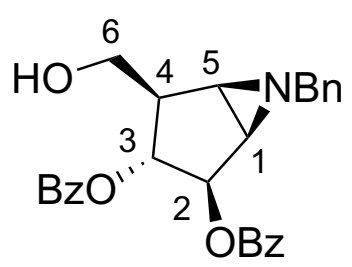

To a flask containing compound **14** (2.95 g, 4.80 mmol, 1.0 eq.) and acetic acid (300 mL), activated zinc powder (13.0 g, 195 mmol, 40 eq.) was added. The greyish mixture was stirred in a preheated oil bath at 35 °C for 2 h. The mixture was allowed to cool to room temperature, filtered over celite which was rinsed with MeOH (2 × 100 mL). The solution was concentrated under

reduced pressure and redissolved in MeOH. Et_3N was added until pH ≈ 7 was reached after which the mixture was stirred for two days at room temperature. The mixture was concentrated under reduced pressure, redissolved in CH_2Cl_2, washed with water, dried with $MgSO_4$, filtered and concentrated *in vacuo*. The crude mixture was purified by silica gel column chromatography (30 → 60% EtOAc in *n*-pentane) to obtain the product as a colourless solid in 80% yield (1.70 g, 3.83 mmol). Characterisation data are in agreement with the literature.[26] ^{1}H-NMR (400 MHz, $CDCl_3$): δ 8.06 – 7.99 (m, 4H, 4xCH_{Ar} Bz), 7.60 – 7.51 (m, 2H, 2xCH_{Ar} Bz), 7.46 – 7.39 (m, 4H, 4xCH_{Ar} Bz), 7.37 – 7.32 (m, 2H, 2xCH_{Ar} Bn), 7.16 – 7.11 (m, 3H, 3xCH_{Ar} Bn), 5.59 (dd, *J* = 6.1, 3.0 Hz, 1H, CH–2), 5.45 (dd, *J* = 7.3, 6.1 Hz, 1H, CH–3), 3.98 (dd, *J* = 11.1, 5.4 Hz, 1H, CH–6a), 3.86 (dd, *J* = 11.2, 6.2 Hz, 1H, CH–6b), 3.58 (d, *J* = 13.6 Hz, 1H, C<u>H</u>H Bn), 3.27 (d, *J* = 13.7 Hz, 1H, CH<u>H</u> Bn), 2.66 (dd, J = 5.0, 3.1 Hz, 1H, CH–1), 2.49 (dd, J = 5.0, 3.0 Hz, 1H, CH–5), 2.47 – 2.41 (m, 1H, CH–4). ^{13}C- NMR (101 MHz, $CDCl_3$): δ 166.7, 166.6 (2xCO Bz), 138.6 ($C_{q,Ar}$), 133.4, 133.3 (2xCH_{Ar} Bz), 130.03, 129.96 (2xCH_{Ar} Bz), 129.8, 129.7 (2x$C_{q,Ar}$), 128.52, 128.46, 128.4, 127.7, 127.2 (5xCH_{Ar}), 80.0 (C–2), 62.6 (C–6), 61.4 (CH_2 Bn), 46.4 (C–4), 43.1 (C–5), 42.6 (C–1). HRMS: calculated for $[C_{27}H_{25}NO_5 + H]^+$ 444.1806, found 444.1798. **Note**: The ^{13}C-NMR signal of C–3 overlaps with the $CDCl_3$ triplet and is therefore unassigned.

2,3-Di-*O*-benzoyl D-*Araf* cyclopentene (16)

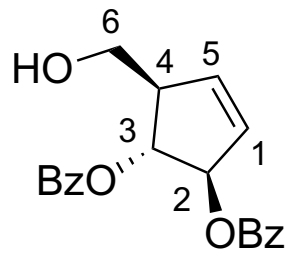

To a solution of aziridine **15** (793 mg, 1.78 mmol, 1.0 eq.) in CH_2Cl_2 (20 mL) was added $NaHCO_3$ (2.24 g, 26.7 mmol, 15 eq.) and *m*-CPBA (1.23 g, 7.15 mmol, 4.0 eq.). The mixture was stirred for 4 days at room temperature. The reaction was diluted with water and extracted three times with CH_2Cl_2. The organic phases were dried with $MgSO_4$, filtered and concentrated *in vacuo*. The crude mixture was purified by silica gel column chromatography (5 → 50% EtOAc in *n*-pentane) to obtain the product as a colourless solid in 80% yield (480 mg, 1.42 mmol). Characterisation data are in agreement with the literature.[27] ^{1}H-NMR (400 MHz, $CDCl_3$): δ 8.11 – 8.02 (m, 4H, 4xCH_{Ar} Bz), 7.62 – 7.54 (m, 2H, 2xCH_{Ar} Bz), 7.49 – 7.41 (m, 4H, 4xCH_{Ar} Bz), 6.17 (ddt, *J* = 2.9, 2.0, 0.9 Hz, 1H, CH–2), 6.05 (dt, *J* = 5.9, 2.0 Hz, 1H, CH–1), 6.02 – 5.99 (m, 1H, CH–5), 5.47 (t, *J* = 2.9 Hz, 1H, CH–3), 3.90 (dd, *J* = 11.1, 4.3 Hz, 1H, CH–6a), 3.77 (dd, *J* = 11.1, 7.5 Hz, 1H, CH–6b), 3.01 (dttd, *J* = 5.8, 4.7, 2.3, 1.3 Hz, 1H, CH–4). ^{13}C-NMR (101 MHz, $CDCl_3$): δ 167.3, 166.1 (2xCO), 135.9 (C–5), 133.7, 133.4, 130.0, 129.9 (4xCH_{Ar}), 129.8 ($C_{q,Ar}$), 129.7 (C–1), 129.5 ($C_{q,Ar}$), 128.63, 128.56 (2xCH_{Ar}), 84.1 (C–2), 81.9 (C–3), 64.3 (C–6), 55.2 (C–4). HRMS: calculated for $[C_{20}H_{18}O_5 + Na]^+$ 361.1046, found 361.1046.

6-*O*-*Tert*-butyldimethylsilyl D-*Araf* cyclopentene (17)

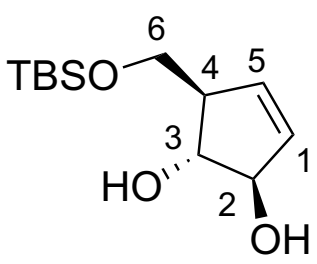

To a flask under N_2 atmosphere charged with alcohol **16** (700 mg, 2.07 mmol, 1.0 eq.) and dry CH_2Cl_2 (30 mL) was added imidazole (280 mg, 4.14 mmol, 2.0 eq.). The solution was cooled to 0 °C and TBSCl (410 mg, 2.72 mmol, 1.2 eq.) was added. The solution was allowed to warm to room temperature and stirred for 16 h. The mixture was diluted with CH_2Cl_2 and washed consecutively with sat. aq. solution of NH_4Cl (2x) and water. The organic phase was dried with Na_2SO_4, filtered, and concentrated *in vacuo*. The crude material was redissolved in a mixture of dry MeOH and CH_2Cl_2 (30 mL, 2:1) under N_2 atmosphere. Solid NaOMe (319 mg, 5.90 mmol, 3.0 eq.) was added and the solution was stirred for 16 h. The mixture was diluted with CH_2Cl_2 and washed consecutively with sat. aq. solution of $NaHCO_3$ and water.

The organic phase was dried with Na_2SO_4, filtered, and concentrated *in vacuo*. The crude mixture was purified by silica gel column chromatography (20 → 70% EtOAc in *n*-pentane) to obtain cycloalkene **17** as a colourless solid in 72% yield (370 mg, 1.51 mmol). ^{1}H-NMR (500 MHz, MeOD-d_4): δ 5.80 (dt, J = 6.1, 1.7 Hz, 1H, CH–5), 5.71 (dt, J = 6.1, 2.0 Hz, 1H, CH–1), 4.50 (dq, J = 4.5, 1.5 Hz, 1H, CH–2), 3.81 (dd, J = 5.2, 4.2 Hz, 1H, CH–3), 3.76 (dd, J = 9.9, 5.2 Hz, 1H, CH–6a), 3.61 (dd, J = 10.0, 6.5 Hz, 1H, CH–6b), 2.56 (dddd, J = 8.4, 6.7, 4.1, 2.5 Hz, 1H, CH–4), 0.91 (s, 9H, $C(CH_3)_3$ TBS), 0.08 (s, 6H, 2xCH_3 TBS). ^{13}C-NMR (126 MHz, MeOD-d_4): δ 134.0 (C–5), 133.7 (C–1), 84.2 (C–2), 82.8 (C–3), 65.5 (C–6), 55.9 (C–4), 26.4 (3xCH_3 TBS), 19.2 ($\underline{C}CH_3$ TBS), –5.24, –5.27 (2xCH_3 TBS). HRMS: calculated for $[C_{12}H_{24}O_3Si + Na]^+$ 267.1387, found 267.1384.

6-*O*-*Tert*-butyldimethylsilyl β-D-*Araf* cyclitol epoxide (18)

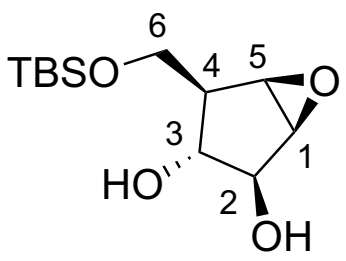

A flask under N_2 atmosphere was charged with cyclopentene **17** (185 mg, 0.757 mmol, 1.0 eq.), dry CH_2Cl_2 (40 mL) and solid $NaHCO_3$ (191 mg, 2.27 mmol, 3.0 eq.). To the stirred suspension, solid *m*-CPBA (261 mg, 1.51 mmol, 2.0 eq.) was added at room temperature. After 6 h the mixture was diluted with aq. sat. $NaHCO_3$ and water before it was extracted three times with CH_2Cl_2. The combined organic phases were dried with Na_2SO_4, filtered and concentrated *in vacuo*. The crude mixture was purified by silica gel column chromatography (70 → 100% Et_2O in *n*-pentane) to obtain epoxide **18** as a colourless solid in 87% yield (171 mg, 0.657 mmol). ^{1}H NMR (400 MHz, $CDCl_3$): δ 4.10 (ddd, J = 7.3, 5.8, 1.5 Hz, 1H, CH–2), 3.93 (dd, J = 9.5, 7.1 Hz, 1H, CH–6a), 3.85 (dd, J = 9.5, 8.6 Hz, 1H, CH–6b), 3.54 (dd, J = 3.1, 1.5 Hz, 1H, CH–1), 3.49 (td, J = 7.5, 6.1, 2.3 Hz, 1H, CH–3), 3.44 (dd, J = 3.1, 1.3 Hz, 1H, CH–5), 2.46 (d, J = 2.7 Hz, 1H, OH–3), 2.42 (d, J = 7.4 Hz, 1H, OH–2), 2.12 (dtd, J = 8.5, 7.2, 1.3 Hz, 1H, CH–4), 0.90 (s, 9H, $C(CH_3)_3$ TBS), 0.09 (s, 6H, 2xCH_3 TBS). ^{13}C NMR (101 MHz, $CDCl_3$): δ 79.5 (C–2), 78.2 (C–3), 63.3 (C–6), 56.5 (C–1), 54.1 (C–5), 47.9 (C–4), 26.0 (3xCH_3 TBS), 18.4 ($\underline{C}CH_3$ TBS), –5.4 (2xCH_3 TBS). HRMS: calculated for $[C_{12}H_{24}O_4Si + Na]^+$ 283.1336, found 283.1338.

β-D-*Araf* cyclitol epoxide (1)

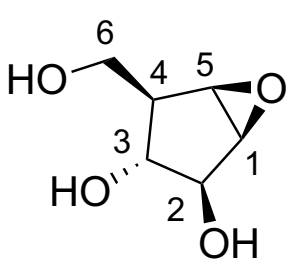

A flask under argon atmosphere containing compound **18** (10.0 mg, 38.4 μmol, 1.0 eq.), dry THF (0.5 mL) and solid TBAF on silica (1.5 mmol/g, 51 mg, 76.8 μmol, 2.0 eq.) was stirred for 8 h. The reaction mixture was evaporated to dryness. The crude mixture was purified by silica gel column chromatography (0 → 15% MeOH in CH_2Cl_2) to obtain epoxide **1** as a colourless solid in 73% yield (4.1 mg, 28.1 μmol). Characterisation data are in agreement with the reported enantiomer.[28] ^{1}H NMR (500 MHz, MeOD-d_4): δ 3.94 (dd, J = 6.1, 1.6 Hz, 1H, CH–2), 3.81 (dd, J = 10.6, 4.7 Hz, 1H, CH–6a), 3.67 (t, J = 10.1 Hz, 1H, CH–6b), 3.57 – 3.54 (m, 1H, CH–5), 3.50 – 3.44 (m, 1H, CH–1), 3.18 (dd, J = 7.6, 6.1 Hz, 1H, CH–3), 2.01 – 1.93 (m, 1H, CH–4). ^{13}C NMR (126 MHz, MeOD-d_4): δ 80.3 (C–2), 76.0 (C–3), 61.7 (C–6), 57.6 (C–1), 54.9 (C–5), 49.5 (C–4). HRMS: calculated for $[C_6H_{10}O_4 + Na]^+$ 169.0471, found 169.0473.

3,4,6-Tri-*O*-(4-methoxybenzyl) α-D-*Araf* cyclitol diol (19)

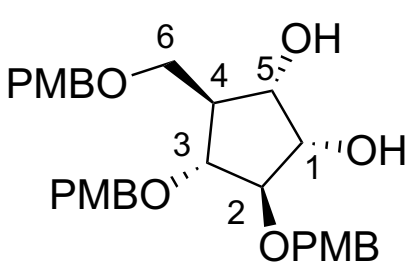

A flask under argon atmosphere was charged with cyclopentene **16** (510 mg, 1.51 mmol, 1 eq.) and anhydrous methanol (10 mL). Anhydrous NaOMe (319 mg, 5.91 mmol, 4 eq.) was added and the

mixture was stirred overnight at room temperature. The mixture was quenched by dropwise addition of 1 m aqueous HCl until pH ~ 7.5, concentrated *in vacuo* and co-evaporated with toluene. The mixture was dissolved in anhydrous DMF (10 mL) and anhydrous THF (2 mL), cooled to 0 °C and put under argon atmosphere. NaH (60% in mineral oil, 355 mg, 8.87 mmol, 6 eq.) was added and the mixture was stirred at 0 °C. After 30 minutes, PMBCl (1.2 mL, 8.89 mmol, 6 eq.) and TBAI (55.0 mg, 0.148 mmol, 10 mol%) were added and the mixture was stirred overnight at room temperature. The mixture was then diluted with CH_2Cl_2 and washed with aqueous NH_4Cl. The aqueous layer was extracted with CH_2Cl_2. The combined organic layers were dried with $MgSO_4$, filtered and concentrated *in vacuo*. The product was purified by silica gel column chromatography (0 → 60% Et_2O in *n*-pentane). The resulting oil was dissolved in 6:1 acetone/water (6:1 mL) and put under argon atmosphere. NMO (346 mg, 2.96 mmol, 2 eq.) and OsO_4 (0.2 mL of 2% aqueous solution, 4.0 mg, 16.0 µmol, 1 mol%) were added and the mixture was stirred overnight at room temperature. The reaction was quenched by dropwise addition of aqueous $Na_2S_2O_3$, as well as a dropwise addition of aqueous $NaHCO_3$. The mixture was then washed with $Na_2S_2O_3$, $NaHCO_3$, water and extracted with CH_2Cl_2 three times. The combined organic layer was dried with $MgSO_4$, filtered and concentrated *in vacuo*. The crude mixture was purified by flash column chromatography (0 → 100% EtOAc in *n*-pentane) to obtain the product in 64% yield (503 mg, 0.960 mmol). ^{1}H-NMR (400 MHz, $CDCl_3$): δ 7.31 – 7.11 (m, 6H, 6xCH_{Ar}), 6.90 – 6.76 (m, 6H, 6xCH_{Ar}), 4.63 – 4.33 (m, 6H, 3xCH_2 PMB), 3.97 – 3.86 (m, 3H, CH–1, CH–2, CH–5), 3.85 – 3.74 (m, 9H, 3xCH_3 PMB), 3.66 – 3.54 (m, 2H, CH–3, CH–6a), 3.44 (dd, *J* = 9.2, 7.1 Hz, 1H, CH–6b), 3.23 (br s, 1H, OH), 3.09 (br s, 1H, OH), 2.30 (qd, *J* = 7.2, 4.8 Hz, 1H, CH–4). ^{13}C NMR (101 MHz, $CDCl_3$): δ 159.31, 159.27, 159.2 (3x$C_{q,Ar}$O PMB), 130.3, 130.2, 130.1 (3x$C_{q,Ar}$ PMB), 129.8, 129.6, 129.4, 113.86, 113.85, 113.8 (6xCH_{Ar}), 88.4 (C–2), 82.0 (C–3), 74.8, 73.3 (C–1, C–5), 73.0, 71.7, 71.4 (3xCH_2 PMB), 70.2 (C–6), 55.32, 55.30 (3xCH_3 PMB), 48.5 (C–4). HRMS: calculated for $[C_{30}H_{36}O_8 + Na]^+$ 547.2302, found 547.2297.

3,4,6-Tri-*O*-(4-methoxybenzyl) β-D-*Araf* cyclitol diol (20)

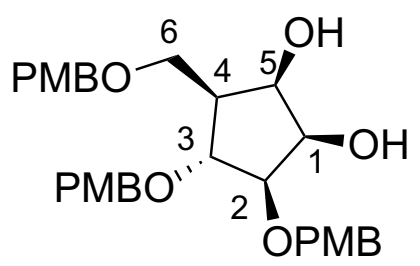

A flask was charged with diol **19** (100 mg, 191 µmol, 1 eq.) and co-evaporated with toluene. The flask was put under argon atmosphere and charged with anhydrous CH_2Cl_2 (2 mL) and anhydrous pyridine (0.15 mL, 1.86 mmol, 10 eq.) and cooled to –10 °C. Triflic anhydride (130 µL, 774 µmol, 4 eq.) was added dropwise and the mixture was stirred at –10 °C. After 1.5 hours, cesium acetate (368 mg, 1.92 mmol, 10 eq.) and anhydrous DMF (2 mL) were added at –10 °C and the mixture was stirred overnight at room temperature. The mixture was then diluted with ethyl acetate and washed with aqueous $NaHCO_3$ and brine. The aqueous layers were combined and extracted with ethyl acetate. The combined organic layer was dried with $MgSO_4$, filtered and concentrated *in vacuo*. The resulting oil was dissolved in methanol (2 mL) and put under argon atmosphere. Methanolic NaOMe was added dropwise until pH > 10 and the mixture was stirred at room temperature for 30 minutes. The mixture was then washed with aqueous $NaHCO_3$. The aqueous layer was extracted with CH_2Cl_2 three times. The combined organic layer was dried with $MgSO_4$, filtered and concentrated *in vacuo*. The crude product was purified by silica gel column chromatography (0 → 100% EtOAc in *n*-pentane) to obtain the product in 48% yield (48.0

mg, 91.5 µmol). ^{1}H-NMR (400 MHz, $CDCl_3$): δ 7.27 – 7.14 (m, 6H, 6xCH_{Ar}), 6.90 – 6.82 (m, 6H, 6xCH_{Ar}), 4.60 (d, *J* = 11.3 Hz, 1H, $C\underline{H}H$ PMB), 4.52 (d, *J* = 11.3 Hz, 1H, $CH\underline{H}$ PMB), 4.45 (d, *J* = 11.0 Hz, 3H, CH_2 PMB, $C\underline{H}H$ PMB), 4.38 (d, *J* = 11.3 Hz, 1H, $CH\underline{H}$ PMB), 4.21 (td, *J* = 6.5, 4.2 Hz, 1H, CH–5), 4.09 (ddd, *J* = 8.7, 5.6, 4.3 Hz, 1H, CH–1), 3.94 (dd, *J* = 6.9, 4.3 Hz, 1H, CH–3), 3.83 (dd, *J* = 5.4, 4.4 Hz, 1H, CH–2), 3.80 (d, *J* = 2.7 Hz, 9H, 3xCH_3 PMB), 3.70 – 3.61 (m, 2H, CH–6a, CH–6b), 3.01 (d, *J* = 8.6 Hz, 1H, OH–1), 2.87 (d, *J* = 6.6 Hz, 1H, OH–6), 2.24 – 2.10 (m, 1H, CH–4). ^{13}C-NMR (101 MHz, $CDCl_3$): δ 159.6, 159.5, 159.4 (3x$C_{q,Ar}$O PMB), 130.4, 129.92 (3x$C_{q,Ar}$ PMB), 129.85, 129.6, 129.5, 114.02, 114.00, 113.9 (6xCH_{Ar}), 83.7 (C–3), 83.2 (C–2), 73.2 (CH_2 PMB), 72.8 (C–5), 72.4 (CH_2 PMB), 72.1 (C–1), 72.0 (CH_2 PMB), 67.9 (C–6), 55.43, 55.41 (3xCH_3 PMB), 46.6 (C–4). HRMS: calculated for $[C_{30}H_{36}O_8 + Na]^+$ 547.2302, found 547.2305.

3,4,6-Tri-*O*-(4-methoxybenzyl) β-D-*Araf* cyclitol cyclosulfate (21)

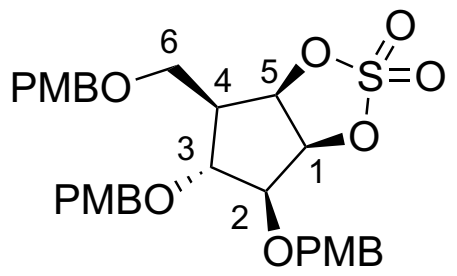

A flask under argon atmosphere was charged with diol **20** (28.0 mg, 53.4 µmol, 1 eq.) and anhydrous CH_2Cl_2 (0.75 mL). Triethylamine (744 µL, 5.33 mmol, 100 eq.) was added and the mixture was cooled to –65 °C. SO_2Cl_2 (17.3 µL, 213 µmol, 4 eq.) was added dropwise over a period of 5 minutes. The mixture was stirred at –65 °C for 4 hours, before the addition of more anhydrous CH_2Cl_2 (1 mL) and SO_2Cl_2 (17.3 µL, 213 µmol, 4 eq.). The mixture was then stirred overnight at –65 °C. The mixture was diluted with anhydrous CH_2Cl_2 (10 mL) and quenched with 10 drops of water at –65 °C. The mixture was washed with aqueous $NaHCO_3$ and extracted with CH_2Cl_2 twice. The combined organic layer was dried with $MgSO_4$, filtered and concentrated *in vacuo*. The crude product was purified by silica gel column chromatography (0 → 100% Et_2O in *n*-pentane) to obtain the product in 67% yield (21.0 mg, 35.8 µmol). ^{1}H-NMR (400 MHz, $CDCl_3$): δ 7.32 – 7.27 (m, 2H, 2xCH_{Ar}), 7.24 – 7.19 (m, 2H, 2xCH_{Ar}), 7.18 – 7.13 (m, 2H, 2xCH_{Ar}), 6.93 – 6.81 (m, 6H, 6xCH_{Ar}), 5.24 (t, *J* = 5.9 Hz, 1H, CH–5), 5.04 (t, *J* = 5.7 Hz, 1H, CH–1), 4.68 (dd, *J* = 14.2, 11.2 Hz, 2H, 2x$C\underline{H}H$ PMB), 4.49 (dd, *J* = 15.3, 11.3 Hz, 2H, 2x$CH\underline{H}$ PMB), 4.45 – 4.36 (m, 2H, CH_2 PMB), 3.95 (dd, *J* = 10.7, 8.1 Hz, 1H, CH–3), 3.85 – 3.82 (m, 1H, CH–2), 3.82 – 3.79 (m, 9H, 3xCH_3 PMB), 3.62 (dd, *J* = 9.2, 4.8 Hz, 1H, CH–6a), 3.55 (t, *J* = 9.6 Hz, 1H, CH–6b), 2.30 (ddt, *J* = 15.6, 10.5, 5.1 Hz, 1H, CH–4). ^{13}C-NMR (101 MHz, $CDCl_3$): δ 159.8, 159.53, 159.45 (3x$C_{q,Ar}$O PMB), 129.9, 129.7, 129.5 (6xCH_{Ar}), 128.9 ($C_{q,Ar}$), 114.2, 113.98, 113.96 (6xCH_{Ar}), 82.2 (C–2), 81.2 (C–5), 80.0 (C–3), 78.8 (C–1), 73.30, 73.25, 72.5 (3xCH_2 PMB), 65.7 (C–6), 55.44, 55.42 (3xCH_3 PMB), 44.8 (C–4). HRMS: calculated for $[C_{30}H_{34}O_{10}S + NH_4]^+$ 604.2211, found 604.2207.

β-D-*Araf* cyclitol cyclic sulfate (2)

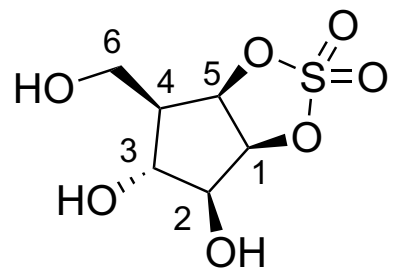

A flask under argon atmosphere was charged with compound **21** (11.7 mg, 19.9 µmol) and methanol (1 mL). $Pd(OH)_2$/C (20 wt%, 6.0 mg) was added, after which argon gas was passed through the solution for 10 minutes. H_2 gas was passed through the solution, and the mixture was stirred for 3 hours at room temperature. argon gas was flushed through the solution, and the mixture was filtered through Celite, covered by a Whatman filter. The crude product was purified by neutralized silica gel chromatography (0 → 6% MeOH in CH_2Cl_2) to afford **2** in 40% yield (1.8 mg, 7.96 µmol). ^{1}H-NMR (850 MHz, MeOD-d_4): δ 5.35 (t, *J* = 6.0 Hz, 1H, CH–5), 5.17 (t, *J* = 5.7 Hz, 1H, CH–1), 3.89 (dd, *J* = 9.1, 5.4 Hz, 1H, CH–2), 3.86 (dd, *J* = 10.9, 4.7 Hz, 1H, CH–6a), 3.81 (dd, *J* = 11.0, 9.1 Hz, 1H, CH–3), 3.76 (t, *J* = 10.6 Hz, 1H, CH–6b),

2.15 (tdd, *J* = 10.7, 6.0, 4.7 Hz, 1H, CH–4). ^{13}C-NMR (214 MHz, MeOD-d_4): δ 82.9 (C–1), 82.7 (C–5), 77.2 (C–2), 74.9 (C–3), 59.0 (C–6), 49.0 (C–4). HRMS: not found.

***N*-Benzyl β-D-*Araf* cyclitol aziridine (22)**

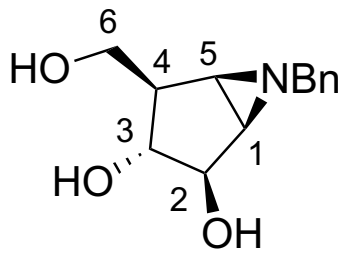

A flask under argon atmosphere was charged with compound **14** (503 mg, 0.819 mmol, 1.0 eq.), and Zinc powder (1.08 g, 16.5 mmol, 40 eq.) and acetic acid (10 mL) were added. The mixture was put in a preheated oil bath at 35 ^{0}C. After 1 hour, the mixture was filtered over Celite and rinsed with methanol. The solvents were evaporated under reduced pressure and co-evaporated with toluene. The solid residue was dissolved in methanol (10 mL) and methanolic NaOMe was added until pH ~ 10 and stirred for 3 hours. Acetic acid was added until pH ~ 7 and the solvent was evaporated under reduced pressure followed by purification of the crude product by neutralized silica gel chromatography (10% MeOH in CH_2Cl_2) to obtain the product as a white solid in quantitative yield (201 mg, 0.819 mmol). Characterisation data are in agreement with the literature.[26] ^{1}H-NMR (D_2O, 400 MHz): δ 7.45 – 7.28 (m, 5H, CH_{Ar}), 4.09 (dd, *J* = 6.5, 2.8 Hz, 1H, CH–2), 3.71 (dd, *J* = 10.7, 4.9 Hz, 1H, CH–6a), 3.60 (dd, *J* = 10.7, 9.5 Hz, 1H, CH–6b), 3.47 (d, *J* = 13.8 Hz, 1H, C<u>H</u>H Bn), 3.35 (d, *J* = 13.9 Hz, 1H, CH<u>H</u> Bn), 3.25 (dd, *J* = 8.2, 6.5 Hz, 1H, CH–3), 2.60 – 2.51 (m, 2H, CH–1, CH–5), 2.08 (dtd, *J* = 10.6, 4.7, 2.5 Hz, 1H, CH–4). ^{13}C-NMR (D_2O, 101 MHz): δ 138.2 ($C_{q,Ar}$), 128.6, 128.0, 127.5 (3xCH_{Ar}), 78.1 (C–2), 75.6 (C–3), 60.9 (C–6), 60.5 (CH_2 Bn), 46.9 (C–4), 45.0 (C–1), 42.4 (C–5). HRMS: calcd. for $[C_{13}H_{17}NO_3 + H]^+$ 236.1281, found 236.1282.

β-D-*Araf* cyclitol aziridine (3)

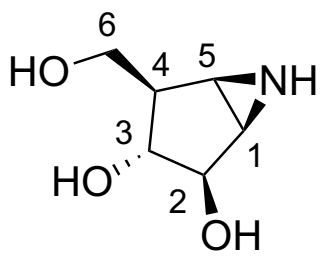

To a solution of compound **22** (289 mg, 1.23 mmol) in water (24 mL) under argon atmosphere was added palladium hydroxide on carbon 20% (288 mg, 2 mmol) and the mixture was hydrogenated at atmospheric pressure with a hydrogen balloon and room temperature for 1 hour. The mixture was filtered through Celite and concentrated to dryness followed by purification of the crude product by neutralized silica gel chromatography (5 → 10% MeOH in CH_2Cl_2), to obtain the product as a white solid in 46% yield (82 mg, 0.56 mmol). Characterisation data are in agreement with the literature.[26] ^{1}H-NMR (D_2O, 400 MHz): δ 4.04 (d, *J* = 6.4 Hz, 1H, CH–2), 3.73 (dd, *J* = 11.0, 4.8 Hz, 1H, CH–6a), 3.63 (t, *J* = 10.1 Hz, 1H, CH–6b), 3.16 (t, *J* = 7.3 Hz, 1H, CH–3), 2.66 (s, 2H, CH–1, CH–5), 2.03 (td, *J* = 8.8, 4.7 Hz, 1H, CH–4). ^{13}C-NMR (D_2O, 101 MHz): δ 78.0 (C–2), 74.7 (C–3), 60.8 (C–6), 46.2 (C–4), 35.3, 32.6 (C–1, C–5). HRMS: calcd. for $[C_6H_{11}NO_3 + H]^+$ 146.0812, found 146.0815.

***N*-Butyl β-D-*Araf* cyclitol aziridine (4)**

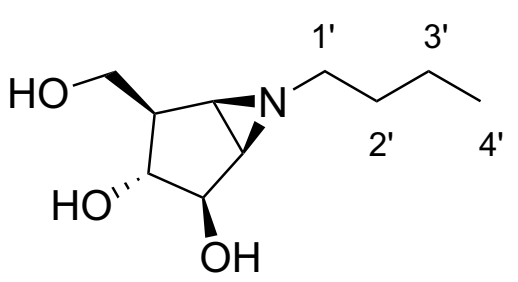

To a flask under argon atmosphere charged with aziridine **3** (11.0 mg, 75.8 µmol, 1.0 eq.) was added a solution of 1-iodobutane in anhydrous DMF (0.5 mL, 0.15 mmol, 0.30 M, 2 eq.) and potassium carbonate (32.0 mg, 232 µmol, 3 eq.). The mixture was heated to 80 ^{0}C and stirred overnight. The mixture was then concentrated *in vacuo* and the product was purified by silica gel chromatography (5 → 10% MeOH in CH_2Cl_2) and reversed-phase column chromatography (1 → 5% CH_3CN in H_2O) to obtain the product as a colourless oil in 23% yield (3.55 mg, 17.6 µmol). ^{1}H-NMR (MeOD-d_4, 500 MHz): δ 3.88 (*J* = 6.3, 3.0 Hz, 1H, CH–2), 3.76 (dd, *J* = 10.2, 4.5 Hz, 1H, CH–6a), 3.66 (t, *J* = 9.9 Hz, 1H, CH–6b), 3.18 (dd,

J = 8.0, 6.3 Hz, 1H, CH–3), 2.34 – 2.25 (m, 1H, CH–1'a), 2.14 (dd, J = 5.2, 2.8 Hz, 1H, CH–5), 2.10 (dd, J = 5.2, 3.0 Hz, 1H, CH–1), 2.04 – 1.95 (m, 1H, CH–1'b), 1.95 – 1.87 (m, 1H, CH–4), 1.54 (quint, J = 6.8 Hz, 2H, CH_2–2'), 1.45 – 1.27 (m, 2H, CH_2–3'), 0.92 (t, J = 7.4 Hz, 2H, CH_3–4'). ^{13}C-NMR (MeOD-d_4, 126 MHz): δ 80.3 (C–2), 77.4 (C–3), 62.7 (C–6), 59.5 (C–1'), 46.2 (C–1), 43.0 (C–5), 32.8 (C–2'), 21.5 (C–3'), 14.4 (C–4'). LC-MS: Rt = 0.67 (10 → 90% CH_3CN, 10 min). HRMS: calcd. For [$C_{10}H_{19}NO_3$ + H]$^+$ 202.1438, found 202.1436. **Note**: The ^{13}C-NMR signal of C–4 overlaps with the MeOD-d_4 multiplet and is therefore unassigned.

(10-Iododec-1-yn-1-yl)trimethylsilane (28)

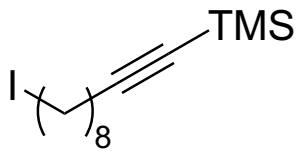

A flask under argon atmosphere was charged with trimethylsilylacetylene (70 µL, 0.50 mmol, 1 eq.) and dry THF (1.8 mL). The mixture was cooled to –78 °C, after which n-butyllithium (2.5 m solution in hexanes, 0.20 mL, 0.50 mmol, 1 eq.) was added. The mixture was allowed to warm up to 0 °C, after which it was stirred for 30 minutes. Then, the mixture was added carefully to a solution of 1,8-diiodooctane (183 mg, 0.50 mmol, 1 eq.) in a mixture of dry THF (1.0 mL) and dry HMPA (0.5 mL) under argon atmosphere at 0 °C. The solution was stirred for 30 minutes, after it was carefully quenched with water. The aqueous layer was extracted three times with Et_2O, after which the combined organic layers were washed with water and brine and dried with $MgSO_4$, filtered and concentrated. The product was purified by silica gel chromatography (100% n-pentane) to afford the product as a colourless oil in 26% yield (44.5 mg, 0.132 mmol). ^{1}H-NMR ($CDCl_3$, 400 MHz): δ 3.19 (t, J = 7.0 Hz, 2H, CH_2I), 2.21 (t, J = 7.1 Hz, 2H, CH_2C alkyne), 1.82 (quint, J = 7.1 Hz, 2H, CH_2), 1.51 (quint, J = 6.9 Hz, 2H, CH_2), 1.42 – 1.27 (m, 8H, 4xCH_2), 0.15 (s, 9H, TMS). ^{13}C-NMR ($CDCl_3$, 126 MHz): δ 107.8, 84.5 (2xC_q alkyne), 33.6, 30.6 (2xCH_2), 29.8 (C_q TMS), 29.0, 28.8, 28.7, 28.5 (4xCH_2), 20.0 ($\underline{C}H_2C$ alkyne), 7.4 (CH_2I), 0.3 (CH_3 TMS).

***N*-[Dec-9-yn-1-yl]-β-D-*Araf* cyclitol aziridine (5)**

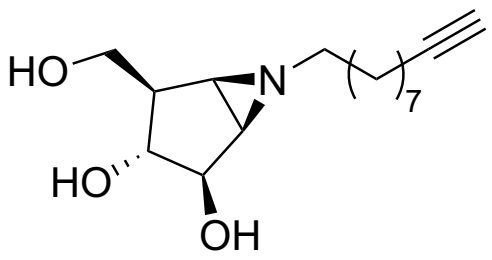

A flask was charged with aziridine **3** (5.2 mg, 36 µmol, 1.0 eq.), iodide **28** (28.0 mg, 83.3 µmol, 2.3 eq.) and anhydrous DMF (0.5 mL). Potassium carbonate (15.7 mg, 0.114 mmol, 3.2 eq.) was added and the mixture was heated to 80 ^{0}C and stirred overnight. The mixture was then quenched by MeOH and concentrated to dryness and the product was purified by neutralized silica gel chromatography (0 → 10% MeOH in CH_2Cl_2) to afford the product as a colourless oil in 40% yield (4.0 mg, 14 µmol). ^{1}H-NMR (MeOD-d_4, 500 MHz): δ 3.88 (dd, J = 6.3, 3.0 Hz, 1H, CH–2), 3.76 (dd, J = 10.2, 4.5 Hz, 1H, CH–6a), 3.66 (t, J = 9.9 Hz, 1H, CH–6b), 3.18 (dd, J = 7.9, 6.2 Hz, 1H, CH–3), 2.33 – 2.24 (m, 1H, C$\underline{H}$HN), 2.19 – 2.13 (m, 4H, CH–5, CH_2C≡C, HC≡C), 2.10 (dd, J = 5.2, 3.0 Hz, 1H, CH–1), 2.02 – 1.96 (m, 1H, CH$\underline{H}$N), 1.91 (dddd, J = 12.4, 7.5, 4.4, 2.8 Hz, 1H, CH–4), 1.60 – 1.46 (m, 4H, 2xCH_2), 1.44 – 1.23 (m, 8H, 4xCH_2). ^{13}C-NMR (MeOD-d_4, 126 MHz): δ 85.1 (C_q alkyne), 80.3 (C–2), 77.5 (C–3), 69.3 (CH alkyne), 62.7 (C–6), 59.8 (CH_2N), 46.2 (C–1), 43.0 (C–5), 30.7, 30.6, 30.1, 29.8, 29.7, 28.4, 19.0 (7xCH_2). Rt = 3.85 (10 → 90% CH_3CN, 10 min). HRMS: calcd. for [$C_{16}H_{27}O_3N$ + H]$^+$ 282.2064, found 282.2064. **Note**: The ^{13}C-NMR signal of C–4 overlaps with the MeOD-d_4 multiplet and is therefore unassigned.

***N*-Azidooctyl β-D-*Araf* cyclitol aziridine (6)**

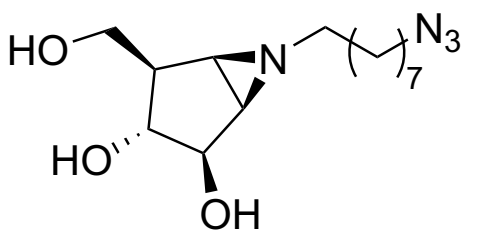

A flask was charged with aziridine **3** (82 mg, 0.56 mmol, 1.0 eq.) and anhydrous DMF (2.5 mL). 1-Azido-8-iodooctane[6] (321 mg, 1.14 mmol, 2.0 eq.) and potassium carbonate (235 mg, 1.70 mmol, 3.0 eq.) were added and the mixture was heated to 80 ^{0}C and stirred overnight. The mixture was then concentrated to dryness and the product was purified by neutralized silica gel chromatography (0 → 10% MeOH in CH_2Cl_2) to obtain the product as a fluffy powder in 31% yield (51.7 mg, 0.173 mmol). ^{1}H-NMR (MeOD-d_4, 400 MHz): δ 3.90 (dd, J = 6.3, 3.0 Hz, 1H, CH–2), 3.76 (dd, J = 10.2, 4.5 Hz, 1H, CH–6a), 3.66 (t, J = 10.0 Hz, 1H, CH–6b), 3.28 (t, J = 6.8 Hz, 2H, CH_2N_3), 3.22 – 3.14 (m, 1H, CH–3), 2.30 (dt, J = 11.7, 7.6 Hz, 1H, $C\underline{H}HN$), 2.16 (dd, J = 5.3, 2.7 Hz, 1H, CH–5), 2.12 (dd, J = 5.3, 3.0 Hz, 1H, CH–1), 1.99 (dt, J = 11.7, 7.3 Hz, 1H, $CH\underline{H}N$), 1.92 (ddd, J = 9.8, 7.4, 4.2 Hz, 1H, CH–4), 1.63 – 1.52 (m, 4H, $C\mathit{H}_2CH_2N$, $C\mathit{H}_2CH_2N_3$), 1.46 – 1.22 (m, 8H, 4xCH_2). ^{13}C-NMR (MeOD-d_4, 101 MHz): δ 80.2 (C–2) , 77.3 (C–3), 62.6 (C–6), 59.7 (CH_2N), 52.4 (CH_2N_3), 49.1 (C–4), 46.2 (C–1), 43.0 (C–5), 30.53, 30.49, 30.2, 29.9, 28.2, 27.7 (6xCH_2). HRMS: calcd. for $[C_{14}H_{26}N_4O_3 + H]^+$ 299.2078, found 299.2078.

1-(((5-Iodopentyl)oxy)methyl)adamantane (29)

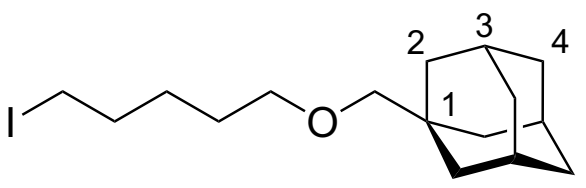

A solution of iodine (6.34 g, 25.0 mmol, 1.5 eq.) in 50 mL THF was added to a solution of 5-(adamantan-1-yl-methoxy)-1-pentanol[29] (4.2 g, 16.7 mmol 1.0 eq.), triphenylphosphine (6.78 g, 25.8 mmol, 1.5 eq.), and imidazole (1.83 g, 26.9 mmol, 1.6 eq.) in 150 mL dry THF. The mixture was refluxed for 2 hours, after which it was allowed to cool to room temperature and diluted with EtOAc and washed with 1 m $Na_2S_2O_3$ twice. The combined aqueous layers were extracted with EtOAc. The combined organic layers were washed with brine, dried with Na_2SO_4, filtered and concentrated. The product was purified by silica gel chromatography (2 → 6% EtOAc in petroleum ether) to afford the product as a colourless oil in 87% yield (5.25 g, 14.5 mmol). Characterisation data are in agreement with the literature.[30] ^{1}H-NMR ($CDCl_3$, 400 MHz): δ 3.38 (t, J = 6.3 Hz, 2H, CH_2O), 3.20 (t, J = 7.0 Hz, 2H, CH_2I), 2.95 (s, 2H, CH_2O), 1.98 – 1.93 (m, 3H, 3xCH–3), 1.86 (quint, J = 7.1 Hz, 2H, CH_2), 1.75 – 1.62 (m, 6H, 3xCH_2–2 or 3xCH_2–4), 1.61 – 1.55 (m, 2H, CH_2), 1.55 – 1.51 (m, 6H, 3xCH_2–2 or 3xCH_2–4), 1.50 – 1.43 (m, 2H, CH_2). ^{13}C-NMR ($CDCl_3$, 101 MHz): δ 82.1 (CH_2O), 71.3 (CH_2O), 39.9, 37.4 (C–2, C–4), 34.2 (C–1), 33.5, 28.6 (2xCH_2), 28.4 (C–3), 27.4 (CH_2), 7.3 (CH_2I).

***N*-[5-(Adamantan-1-yl-methoxy)-pentyl]-β-D-*Araf* cyclitol aziridine (7)**

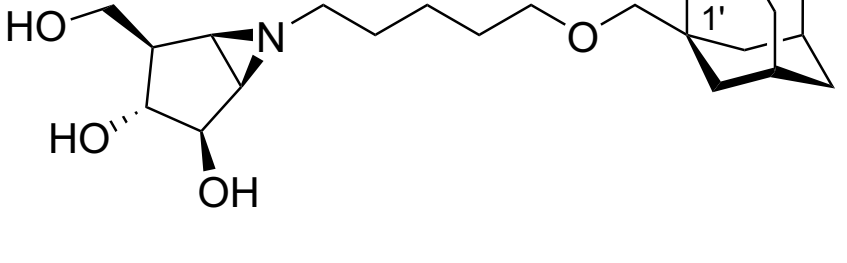

A flask under argon atmosphere was charged with aziridine **3** (129 mg, 0.889 mmol, 1.0 eq.) and 4 mL anhydrous DMF. To this solution, iodide **29** (648 mg, 1.79 mmol, 2.0 eq.) and potassium carbonate (372 mg, 2.67 mmol, 3.0 eq.) were added and the mixture was heated to 80 ^{0}C and stirred overnight. The mixture was then concentrated to dryness and the product was purified by neutralized silica gel chromatography (0 → 8% MeOH in CH_2Cl_2) to obtain the product as a colourless oil in 53% yield (180 mg, 0.475 mmol). ^{1}H-NMR (MeOD-d_4, 400 MHz): δ 3.89 (dd, J = 6.2, 3.0 Hz, 1H, CH–2), 3.76 (dd, J = 10.3, 4.5 Hz, 1H, CH–6a), 3.66 (t, J = 9.9 Hz, 1H, CH–6b), 3.38 (t, J = 6.4 Hz, 2H, CH_2O), 3.18 (dd, J = 8.0, 6.3 Hz, 1H, CH–3), 2.97 (s, 2H, CH_2O), 2.32 (dt, J = 11.7, 7.3 Hz, 1H, $C\underline{H}HN$), 2.17 (dd, J = 5.1, 2.8 Hz, 1H, CH–5), 2.12 (dd, J = 5.2,

3.0 Hz, 1H, CH–1), 2.05 (m, 5H, CHHN, 3xCH–3', CH–4), 1.80 – 1.64 (m, 6H, 3xCH_2–2' or 3xCH_2–4'), 2.62 – 1.51 (m, 10H, 2xCH_2, 3xCH_2–2' or 3xCH_2–4'), 1.40 (quint, *J* = 7.1 Hz, 2H, CH_2). ^{13}C-NMR (MeOD-d_4, 101 MHz): δ 83.0 (CH_2O), 80.2 (C–2), 77.4 (C–3), 72.5 (CH_2O), 62.7 (C–6), 59.7 (CH_2N), 49.1 (C–4), 46.2 (C–1), 43.0 (C–5), 40.8 , 38.3 (C–2', C–4'), 35.2 (C–1'), 30.6, 30.4 (2xCH_2), 29.7 (C–3'), 25.0 (CH_2). Rt = 5.16 (10 → 90% CH_3CN, 10 min). HRMS: calcd. for $[C_{22}H_{37}NO_4 + H]^+$ 380.2795, found 380.2794.

4-(((5-Iodopentyl)oxy)methyl)-1,1'-biphenyl (30)

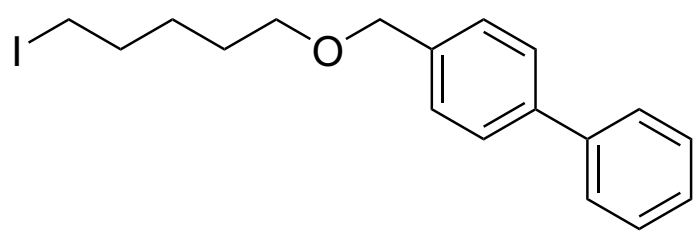

A solution of iodine (4.72 g, 18.6 mmol, 1.6 eq.) in 70 mL THF was added to a solution of 5-([1,1'-biphenyl]-4-ylmethoxy)pentane-1-ol[31] (3.25 g, 12.0 mmol, 1 eq.), triphenylphosphine (4.9 g, 18.7 mmol, 1.6 eq.), and imidazole (1.48 g, 21.8 mmol, 1.8 eq.) in 80 mL THF. The mixture was refluxed for 2 hours, after which it was allowed to cool to room temperature and concentrated *in vacuo*. The product was purified by silica gel chromatography (2 → 5% EtOAc in petroleum ether) to afford the product as a yellow oil in 83% yield (3.80 g, 10.0 mmol). ^{1}H-NMR ($CDCl_3$, 400 MHz): δ 7.60 – 7.53 (m, 4H, 4xCH_{Ar}), 7.45 – 7.37 (m, 4H, 4xCH_{Ar}), 7.35 – 7.29 (m, CH_{Ar}), 4.52 (s, 2H, CH_2O), 3.48 (t, *J* = 6.4 Hz, 2H, CH_2O), 3.17 (t, *J* = 7.0 Hz, 2H, CH_2I), 1.88 – 1.77 (m, 2H, CH_2), 1.69 – 1.58 (m, 2H, CH_2), 1.54 – 1.43 (m, 2H, CH_2). ^{13}C-NMR ($CDCl_3$, 101 MHz): δ 141.0, 140.5, 137.6 (3x$C_{q,Ar}$), 128.8, 128.2, 127.3, 127.21, 127.15 (5xCH_{Ar}), 72.7 (CH_2O), 70.1 (CH_2O), 33.4, 28.8, 27.3 (3xCH_2), 7.1 (CH_2I).

***N*-[5-(Biphenyl-4-yl-methoxy)-pentyl]-β-D-*Araf* cyclitol aziridine (8)**

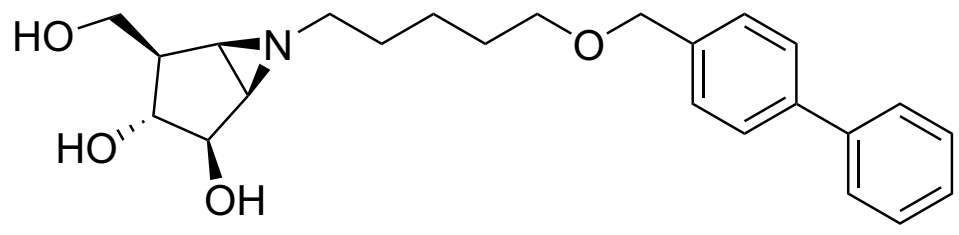

A flask under argon atmosphere was charged with aziridine **3** (117 mg, 0.809 mmol, 1.0 eq.) and 3.5 mL anhydrous DMF. To this solution, iodide **30** (627 mg, 1.65 mmol, 2.0 eq.) and potassium carbonate (336 mg, 2.43 mmol, 3.0 eq.) were added and the mixture was heated to 80 ^{0}C and stirred overnight. The mixture was then concentrated to dryness and the product was purified by neutralized silica gel chromatography (0 → 8% MeOH in CH_2Cl_2) to obtain the product as a colourless oil in 25% yield (80.4 mg, 0.202 mmol). ^{1}H-NMR (MeOD-d_4, 400 MHz): δ 7.64 – 7.55 (m, 4H, 4xCH_{Ar}), 7.46 – 7.37 (m, 4H, 4xCH_{Ar}), 7.32 (t, *J* = 7.3 Hz, 1H, CH_{Ar}), 4.53 (s, 2H, CH_2O), 3.93 (dd, *J* = 6.3, 3.0 Hz, 1H, CH–2), 3.77 (dd, *J* = 10.3, 4.4 Hz, 1H CH–6a), 3.67 (t, *J* = 9.8 Hz, 1H, CH–6b), 3.52 (t, *J* = 6.4 Hz, 2H, CH_2O), 3.22 (dd, *J* = 8.1, 6.3 Hz, 1H, CH–3), 2.43 – 2.18 (m, 3H, CHHN, CH–1, CH–5), 2.14 – 2.02 (m, 1H, CHHN), 2.00 – 1.90 (m, 1H, CH–4), 1.70 – 1.55 (m, 4H, 2xCH_2), 1.51 – 1.37 (m, 2H, CH_2). ^{13}C-NMR (MeOD-d_4, 101 MHz): δ 142.1, 141.8, 138.9 (3x$C_{q,Ar}$), 129.9, 129.4, 128.3, 127.94, 127.92 (5xCH_{Ar}), 78.0 (C–2), 77.0 (C–3), 73.5, 71.3 (2xCH_2O), 62.4 (C–6), 59.1 (CH_2N), 46.6, 43.6 (C–1, C–5), 30.6, 30.1, 24.9 (3xCH_2). Rt = 4.83 (10 → 90% CH_3CN, 10 min). HRMS: calcd. for $[C_{24}H_{31}NO_4 + H]^+$ 398.2326, found 398.2326. **Note**: The ^{13}C-NMR signal of C–4 overlaps with the MeOD-d_4 multiplet and is therefore unassigned.

***N*-[((3-(Adamantyl-1-yl-methoxy)-propyl)-1*H*-1,2,3-triazol-1-yl)octyl]-β-D-*Araf* cyclitol aziridine (9)**

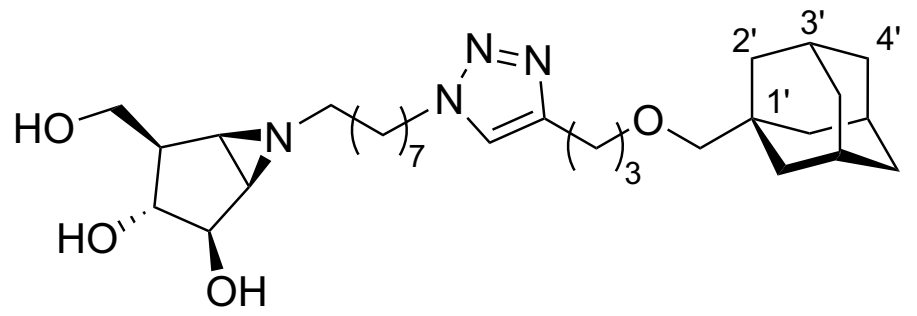

A solution of 5-(adamantan-1-yl-methoxy)-pent-1-yne[29] (30.6 mg, 0.132 mmol, 2.1 eq.) in DMF (2.2 mL) was added to a flask under argon atmosphere charged with azide **7** (18.8 mg, 63 µmol, 1.0 eq.). $CuSO_4$ (126 µL of 100 mm solution in H_2O, 20 mol%) and sodium ascorbate (189 µL of 100 mm solution in H_2O, 30 mol%) were mixed and added, after which the solution was stirred overnight at room temperature. The volatiles were removed under reduced pressure, and the product was purified by neutralized silica gel chromatography (0 → 8% MeOH in CH_2Cl_2) to obtain the product as a colourless oil in 51% yield (17.1 mg, 32.2 µmol). ^{1}H-NMR (MeOD-d_4, 400 MHz): δ 7.74 (s, 1H, CH_{Ar}), 4.36 (t, J = 7.0 Hz, 2H, CH_2N triazole), 3.92 (dd, J = 6.3, 3.0 Hz, 1H, CH–2), 3.77 (dd, J = 10.2, 4.5 Hz, 1H, CH–6a), 3.66 (t, J = 9.9 Hz, 1H, CH–6b), 3.41 (t, J = 6.1 Hz, 2H, CH_2O), 3.20 (dd, J = 8.0, 6.2 Hz, 1H, CH–3), 2.97 (s, 2H, CH_2O), 2.80 – 2.74 (m, 2H, CH_2C triazole), 2.39 – 2.29 (m, 1H, C$\underline{H}$HN aziridine), 2.29 – 2.14 (m, 2H, CH–1, CH–5), 2.10 – 1.99 (m, 1H, CH$\underline{H}$N aziridine), 1.99 – 1.84 (m, 8H, CH–5, 3xCH–3’, 2xCH_2), 1.81 – 1.64 (m, 6H, 3xCH_2–2’ or 3xCH_2–4’), 1.62 – 1.48 (m, 8H, 3xCH_2–2’ or 3xCH_2–4’, CH_2), 1.43 – 1.24 (m, 8H, 4xCH_2). ^{13}C-NMR (MeOD-d_4, 101 MHz): δ 148.8 ($C_{q,Ar}$), 123.2 (CH_{Ar}), 83.1 (CH_2O), 80.1 (C–2), 77.1 (C–3), 71.5 (CH_2O), 62.5 (C–6), 59.3 (CH_2N aziridine), 51.2 (CH_2N triazole), 49.1 (C–4), 46.5 (C–1), 43.5 (C–5), 40.8, 38.3 (C–2’, C–4’), 35.2 (C–1’), 31.3, 30.5, 30.4, 30.3, 30.0 (5xCH_2), 29.8 (C–3’), 28.2, 27.4 (2xCH_2), 23.1 ($\underline{C}H_2C$ triazole). Rt = 5.82 (10 → 90% CH_3CN, 10 min). HRMS: calcd. for $[C_{30}H_{50}O_4N_4 + H]^+$ 531.3905, found 531.3906.

(5-([1,1'-Biphenyl]-4-ylmethoxy)pent-1-yn-1-yl)triethylsilane (31)

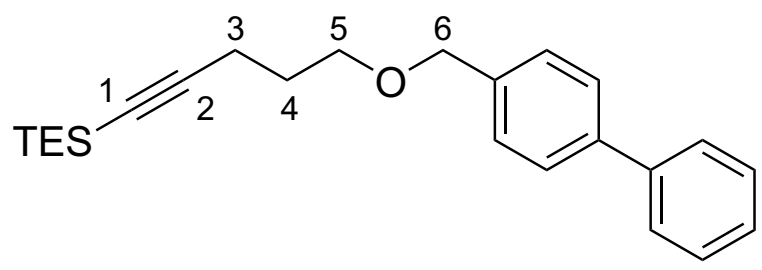

A flask under argon atmosphere was charged with 5-(triethylsilyl)pent-4-yn-1-yl triflate[29] (723 mg, 2.19 mmol, 1.0 eq.) and anhydrous CH_2Cl_2 (40 mL). To the solution, biphenyl-4-methanol (2.02 g, 10.9 mmol, 5.0 eq.) and potassium carbonate (1.52 g, 10.9 mmol, 5.0 eq.) were added. The mixture was refluxed for 3 days, after which the solids were removed by filtration and the filtrate was concentrated *in vacuo*. The residue was purified by silica gel chromatography (0 → 10% EtOAc in toluene) to obtain the product as a yellow oil in 92% yield (731 mg, 2.00 mmol). ^{1}H-NMR ($CDCl_3$, 400 MHz): δ 7.61 – 7.55 (m, 4H, 4xCH_{Ar}), 7.46 – 7.38 (m, 4H, 4xCH_{Ar}), 7.34 (t, J = 7.3 Hz, 1H, CH_{Ar}), 4.55 (s, 2H, CH_2–6), 3.62 (t, J = 6.2 Hz, 2H, CH_2–5), 2.39 (t, J = 7.0 Hz, 2H, CH_2–3), 1.85 (quint, J = 6.7 Hz, 2H, CH_2–4), 0.97 (t, J = 7.9 Hz, 9H, 3xCH_3 TES), 0.56 (q, J = 8.0 Hz, 6H, 3xCH_2 TES). ^{13}C-NMR ($CDCl_3$, 101 MHz): δ 141.1, 140.7, 137.7 (3x$C_{q,Ar}$), 128.9, 128.2, 127.38, 127.30, 127.24 (5xCH_{Ar}), 108.0 (C–1), 82.1 (C–2), 72.9 (C–6), 69.0 (C–5), 29.1 (C–4), 16.9 (C–3), 7.6 (CH_3 TES), 4.67 (CH_2 TES). HRMS: calcd. for $[C_{24}H_{32}OSi + H]^+$ 365.2295, found 365.2298.

4-((Pent-4-yn-1-yloxy)methyl)-1,1'-biphenyl (32)

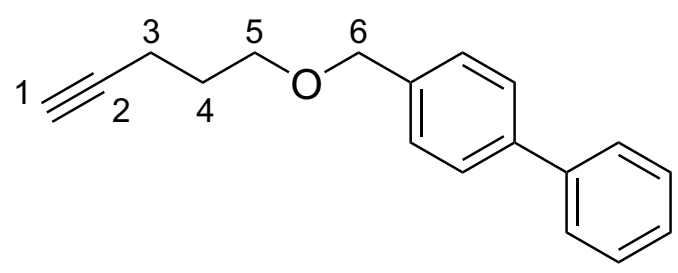

A flask under argon atmosphere was charged with compound **31** (716 mg, 1.96 mmol, 1.0 eq.) and anhydrous THF (20 mL). To the solution, TBAF (4.0 mL, 4.0 mmol, 1.0 m solution in THF, 2.0 eq.) was added. The mixture was stirred for 1 hour at room temperature, after which the reaction was quenched with 30 mL aqueous NH_4Cl. The product was extracted three times with Et_2O, and the combined

organic layers were washed with brine, dried with $MgSO_4$, filtered, concentrated and purified by silica gel chromatography (0 → 2% Et_2O in *n*-pentane) to obtain the product as a yellow oil in 85% yield (417 mg, 1.67 mmol). Characterisation data are in agreement with the literature.[29,32] ^{1}H-NMR ($CDCl_3$, 400 MHz): δ 7.61 – 7.52 (m, 4H, 4xCH_{Ar}), 7.44 – 7.37 (m, 4H, 4xCH_{Ar}), 7.35 – 7.29 (m, 1H, CH_{Ar}), 4.53 (s, 2H, CH_2–6), 3.58 (t, *J* = 6.2 Hz, 2H, CH_2–5), 2.32 (td, *J* = 7.1, 2.7 Hz, 2H, CH_2–3), 1.93 (t, *J* = 2.7 Hz, 1H, CH–1), 1.83 (quint, *J* = 6.5 Hz, 2H, CH_2–4). ^{13}C-NMR ($CDCl_3$, 101 MHz): δ 141.0, 140.6, 137.6 (3x$C_{q,Ar}$), 128.8, 128.2, 127.3, 127.22, 127.16 (5xCH_{Ar}), 84.0 (C–2), 72.8 (C–6), 68.8 (C–5), 68.6 (C–1), 28.8 (C–4), 15.4 (C–3). HRMS: calcd. for $[C_{18}H_{18}O + H]^+$ 251.1430, found 251.1432.

***N*-[((3-(Biphenyl-4-yl-methoxy)-propyl)-1*H*-1,2,3-triazol-1-yl)octyl]-β-d-*Araf* cyclitol aziridine (10)**

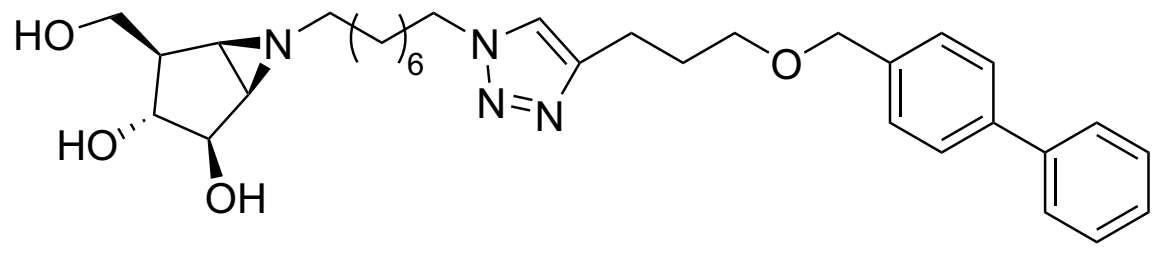

A solution of alkyne **32** (27.2 mg, 0.109 mmol, 2.0 eq.) in DMF (1.8 mL) was added to a flask under argon atmosphere charged with azide **7** (15.7 mg, 53 μmol, 1.0 eq.). $CuSO_4$ (126 μL of 100 mm solution in H_2O, 20 mol%) and sodium ascorbate (189 μL of 100 mm solution in H_2O, 30 mol%) were mixed and added, after which the solution was stirred overnight at room temperature. The volatiles were removed under reduced pressure, and the product was purified by neutralized silica gel chromatography (0 → 8% MeOH in CH_2Cl_2) to obtain the product as a colourless oil in 40% yield (11.6 mg, 21.1 μmol). ^{1}H-NMR (MeOD-*d*$_4$, 400 MHz): δ 7.64 – 7.58 (m, 5H, CH_{Ar} triazole, 4xCH_{Ar} biphenyl), 7.46 – 7.38 (m, 4H, 4xCH_{Ar} biphenyl), 7.36 – 7.31 (m, 1H, CH_{Ar} biphenyl), 4.54 (s, 2H, CH_2O), 4.29 (t, *J* = 7.1 Hz, 2H, CH_2N triazole), 3.91 (dd, *J* = 6.3, 3.0 Hz, 1H, CH–2), 3.77 (dd, *J* = 10.2, 4.5 Hz, 1H, CH–6a), 3.65 (t, *J* = 9.9 Hz, 1H, CH–6b), 3.53 (t, *J* = 6.2 Hz, 2H, CH_2O), 3.20 (dd, *J* = 8.0, 6.3 Hz, 1H, CH–3), 2.80 (t, *J* = 7.5 Hz, 2H, CH_2C triazole), 2.31 (dt, *J* = 12.6, 7.6 Hz, 1H, C<u>H</u>HN aziridine), 2.26 – 2.16 (m, 2H, CH–1, CH–5), 2.08 – 1.89 (m, 4H, CH–4, CH<u>H</u>N aziridine, CH_2), 1.82 (quint, *J* = 7.2 Hz, 2H, CH_2), 1.60 – 1.48 (m, 2H, CH_2), 1.39 – 1.17 (m, 8H, 4xCH_2). ^{13}C-NMR (MeOD-*d*$_4$, 101 MHz): δ 148.5 ($C_{q,Ar}$ triazole), 142.1, 141.8, 138.9 (3x$C_{q,Ar}$ biphenyl), 129.9, 129.5, 128.4, 127.94, 127.92 (5xCH_{Ar}), 123.3 (CH_{Ar} triazole), 80.1 (C–2), 77.1 (C–3), 73.5, 70.0 (2xCH_2O), 62.5 (C–6), 59.3 (CH_2N aziridine), 51.2 (CH_2N triazole), 49.1 (C–4), 46.5 (C–1), 43.5 (C–5), 31.2, 30.5, 30.4, 30.3, 29.9, 28.1, 27.4 (7xCH_2), 23.0 (<u>C</u>H_2C triazole). Rt = 5.28 (10 → 90% CH_3CN, 10 min). HRMS: calcd. for $[C_{32}H_{44}O_4N_4 + H]^+$ 549.3435, found 549.3436.

Supporting figures

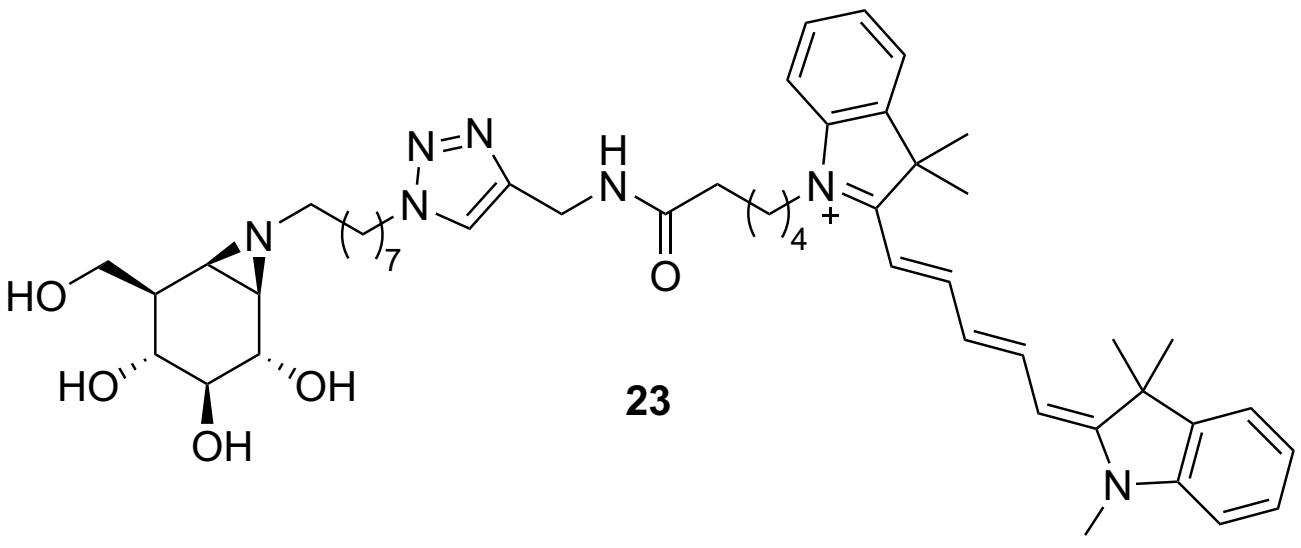

23

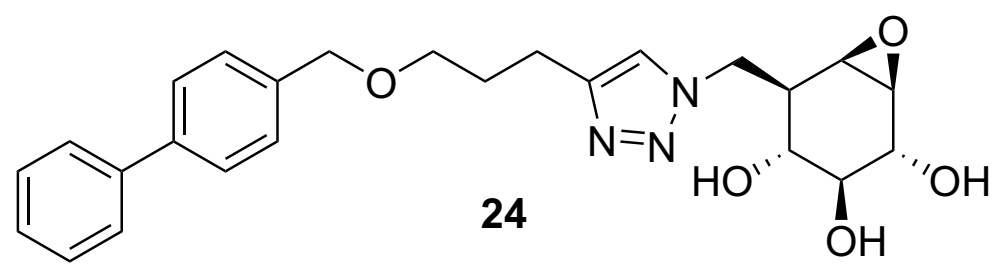

24

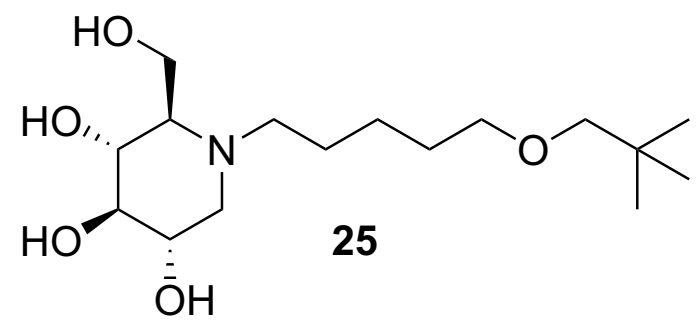

25

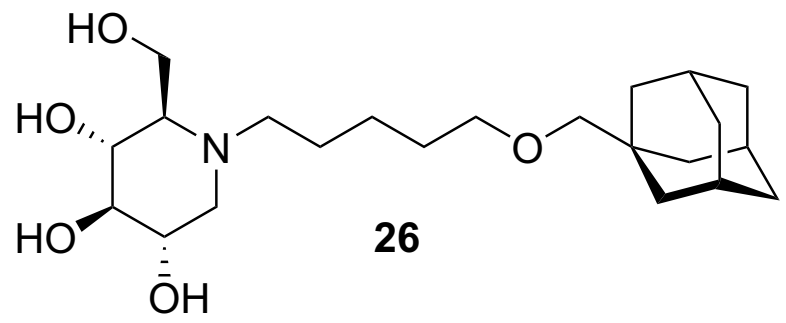

26

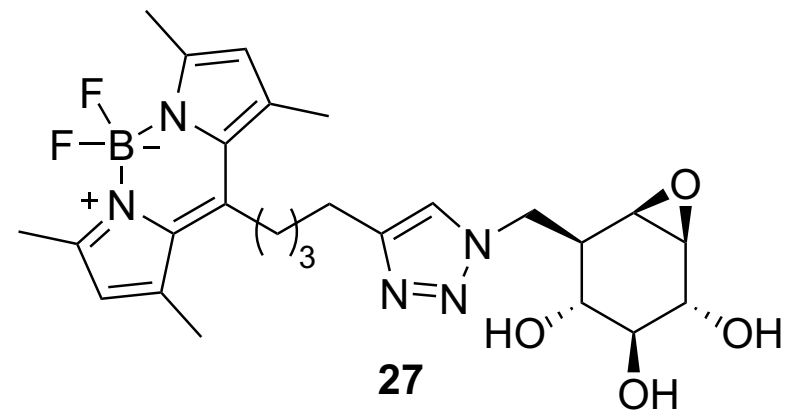

27

Figure S1. Chemical structures of compounds used.

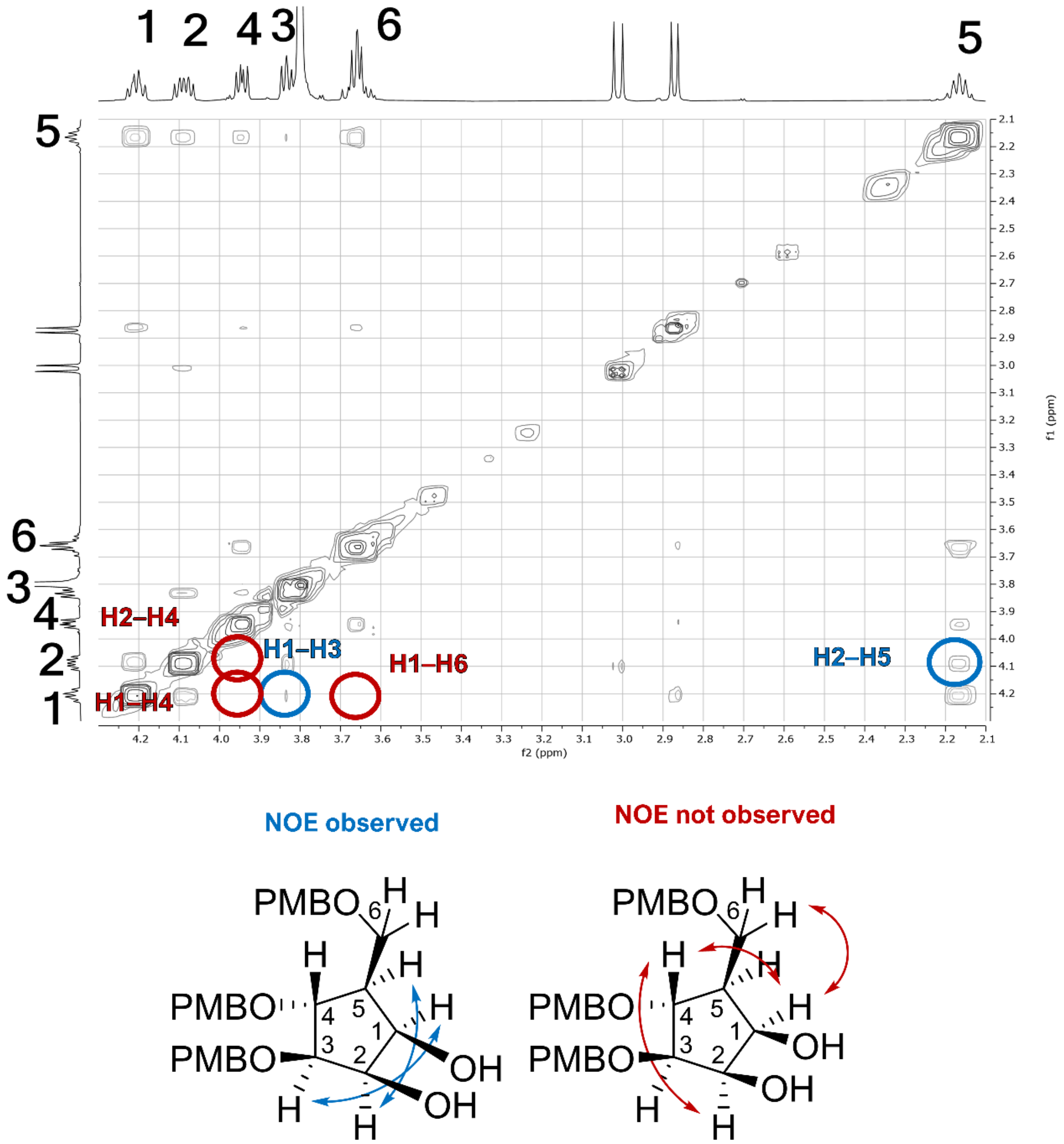

Figure S2. Determination of stereochemistry of diol **20** by NOESY spectroscopy.

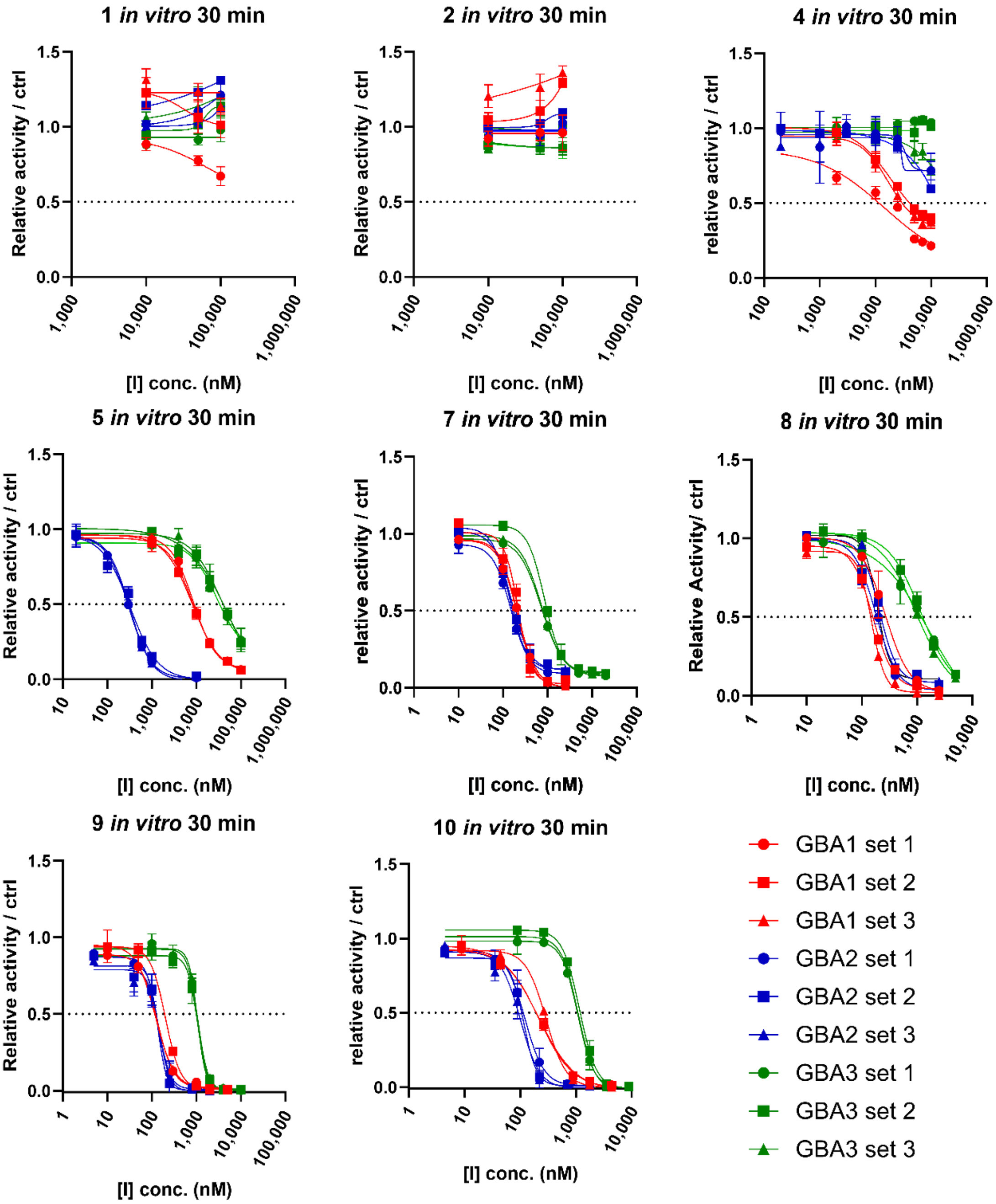

Figure S3. Fitting curves of *in vitro* β-glucosidase IC_{50} measurement using fluorogenic substrates.

Table S1. Individual data points of *in vitro* apparent IC_{50} values obtained using fluorogenic substrates.

Apparent IC_{50} (nM)	GBA2			rhGBA1			GBA3		
Set	1	2	3	1	2	3	1	2	3
**1**	>100,000	>100,000	>100,000	>100,000	>100,000	>100,000	>100,000	>100,000	>100,000
**2**	>100,000	>100,000	>100,000	>100,000	>100,000	>100,000	>100,000	>100,000	>100,000
**4**	>100,000	>100,000	>100,000	14,369	18,872	17,226	>100,000	>100,000	>100,000
**5**	306	328	322	8,688	8,159	8,564	28,893	78,999	35,786
**7**	149	155	141	212	216	209	723	846	724
**8**	181	207	191	265	159	151	1,670	1,166	980
**9**	138	138	145	133	203	121	1,080	1,065	1,058
**10**	121	115	101	218	217	291	1,086	1,162	1,102

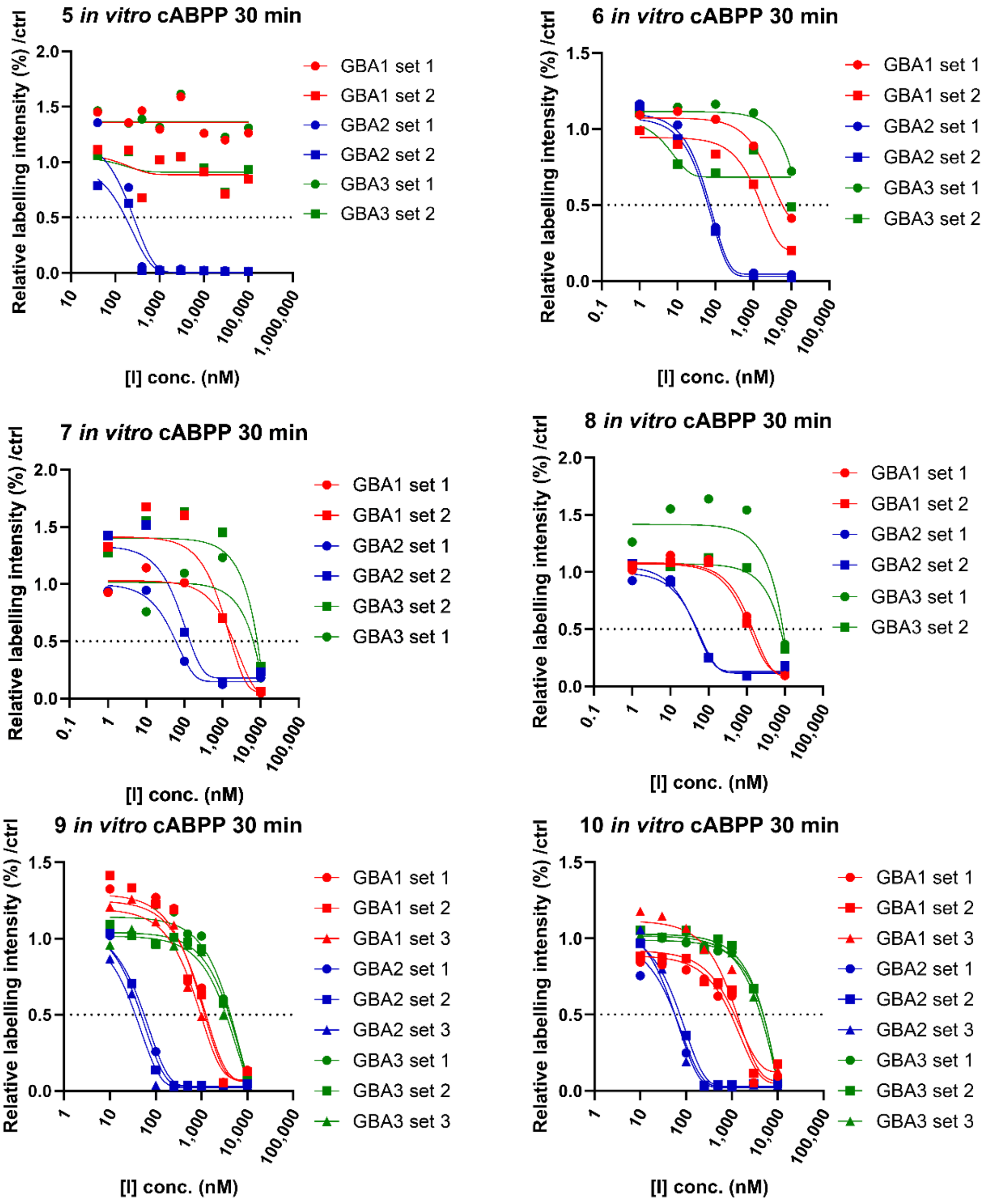

Figure S4. Fitting curves of quantified IC_{50} of *in vitro* labeling of β-glucosidases for 30 minutes with broad-spectrum β-glucosidase ABP **23** after 30 minutes incubation with the inhibitor. HEK293T cells GBA1 endogeneous and GBA2/GBA3 OE lysate was used as the enzyme source. Raw data can be found in **Figure S16**.

Table S2. Individual data points of *in vitro* apparent IC_{50} values obtained using cABPP.

Apparent IC_{50} (nM)	GBA2			rhGBA1			GBA3		
Set	1	2	3	1	2	3	1	2	3
**5**	196	172		>100,000	>100,000		>100,000	>100,000	
**6**	58	55		2,277	1,235		>10,000	>10,000	
**7**	47	76		1,808	1,054		>5,000	>5,000	
**8**	42	36		1,140	928		>5,000	>5,000	
**9**	49	41	33	869	788	693	4,596	5,104	3,892
**10**	56	65	48	1,070	1,061	1,028	21,790	15,384	7,531

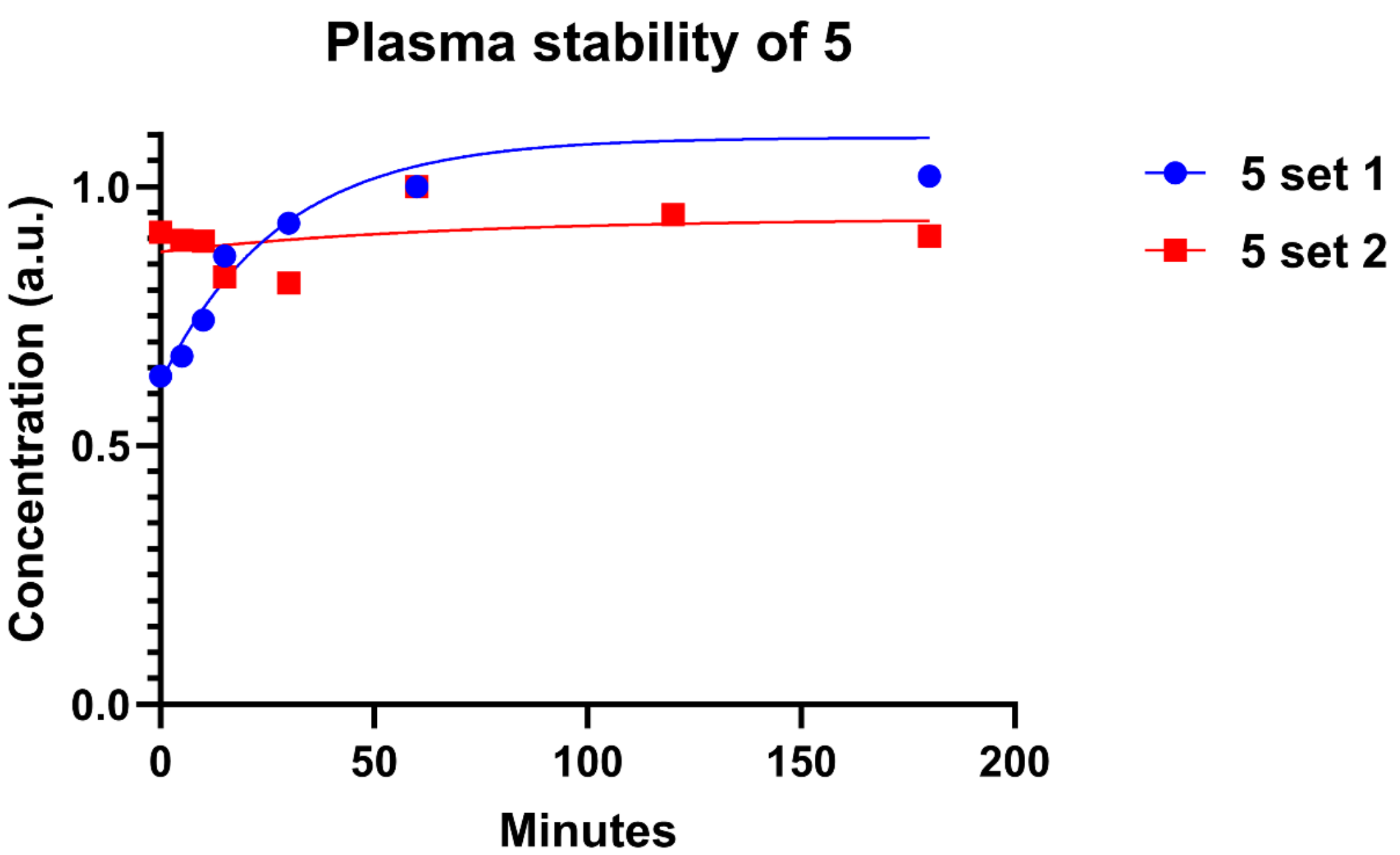

Figure S5. *In vitro* plasma metabolic stability of compound **5**.

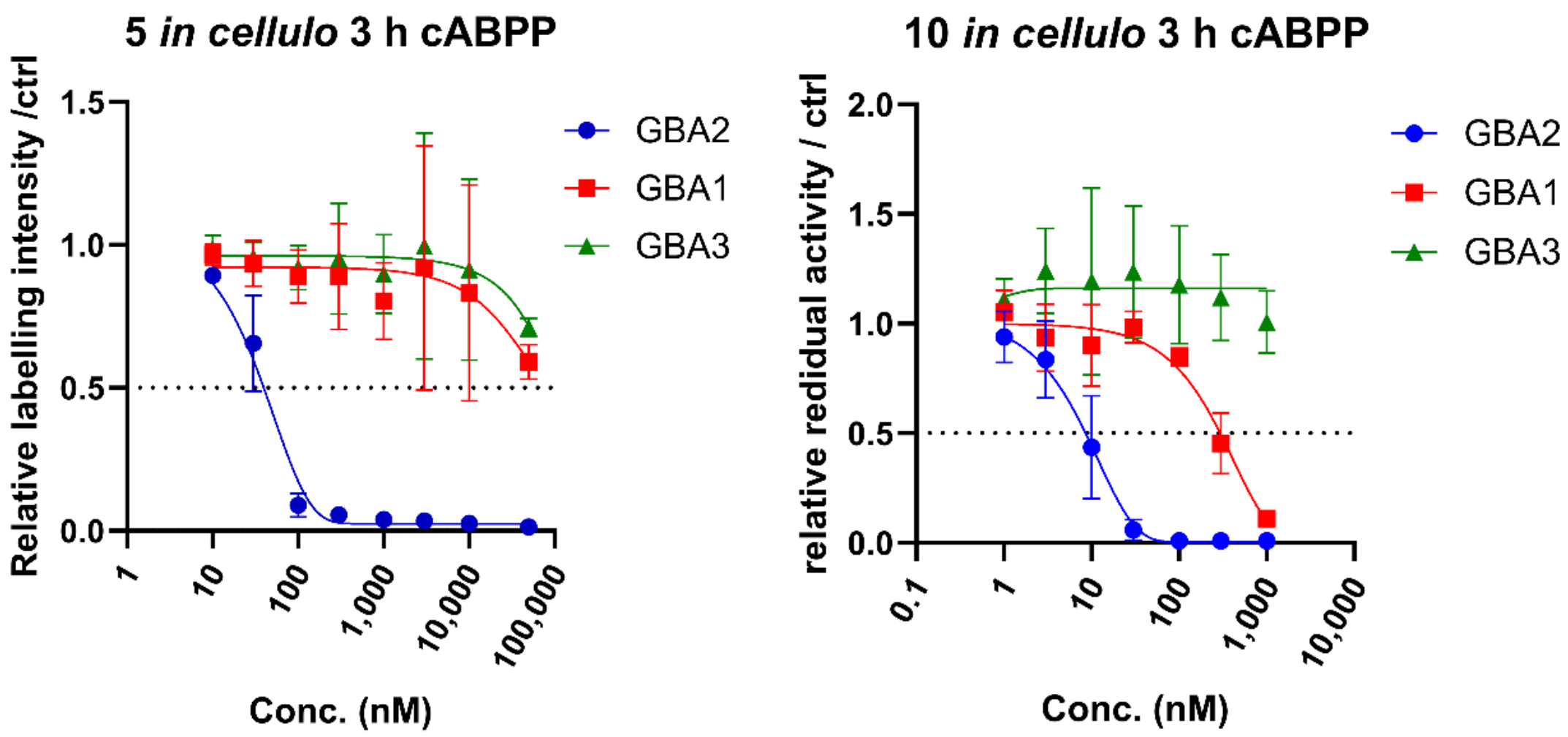

Figure S6. Fitting curves of *in cellulo* competitive ABPP assays using intact living HEK293T cells containing endogenous GBA1 and overexpressed GBA2/GBA3. Raw data can be found in **Figure S17**.

Table S3. Individual data points of *in cellulo* apparent IC_{50} values obtained using cABPP.

Apparent IC_{50} (nM)	GBA2			rhGBA1			GBA3		
Set	1	2	3	1	2	3	1	2	3
**5**	29	39	39	>50,000	>50,000	>50,000	>50,000	>50,000	>50,000
**10**	11	10	4	381	216	326	>1,000	>1,000	>1,000

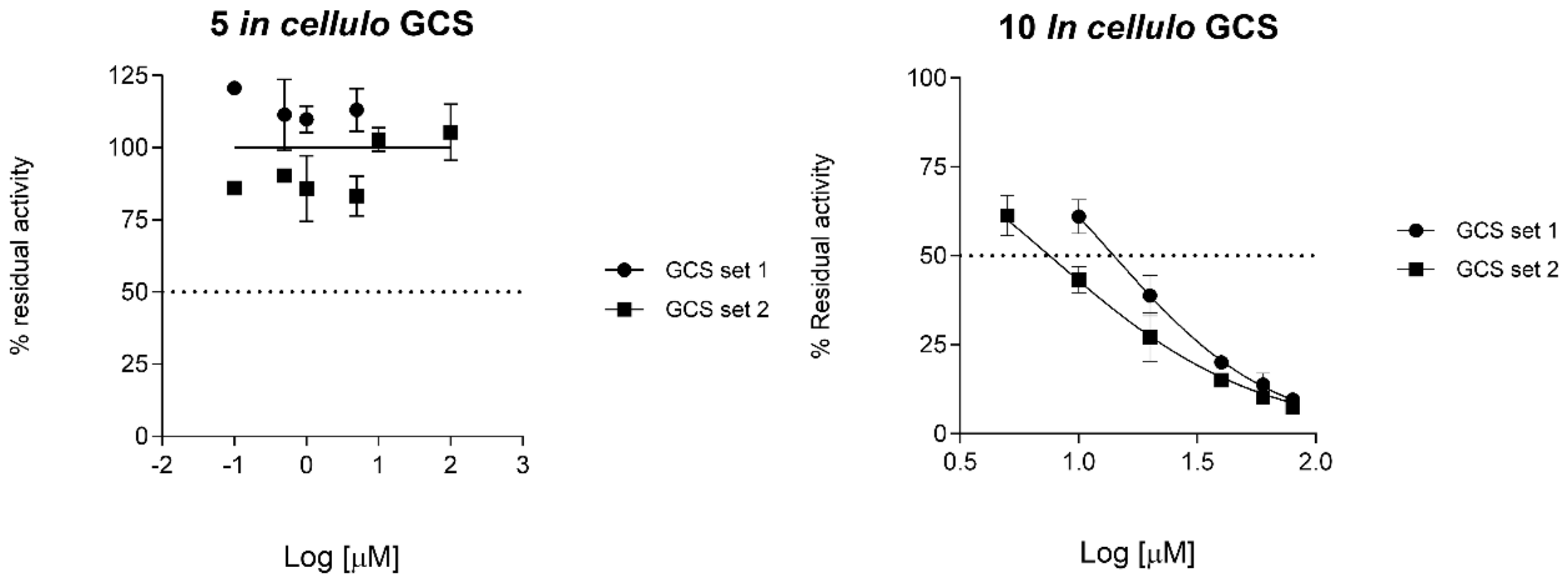

Figure S7. Fitting curves of *in cellulo* IC_{50} values of compounds **5** and **10** against GCS in RAW264.7 cells.

Table S4. Individual data points of *in cellulo* IC_{50} values against GCS.

IC_{50} value (μM)	Set 1	Set 2	Average
**5**	>100,000	>100,000	>100,000
**10**	12.4	7.5	10.0

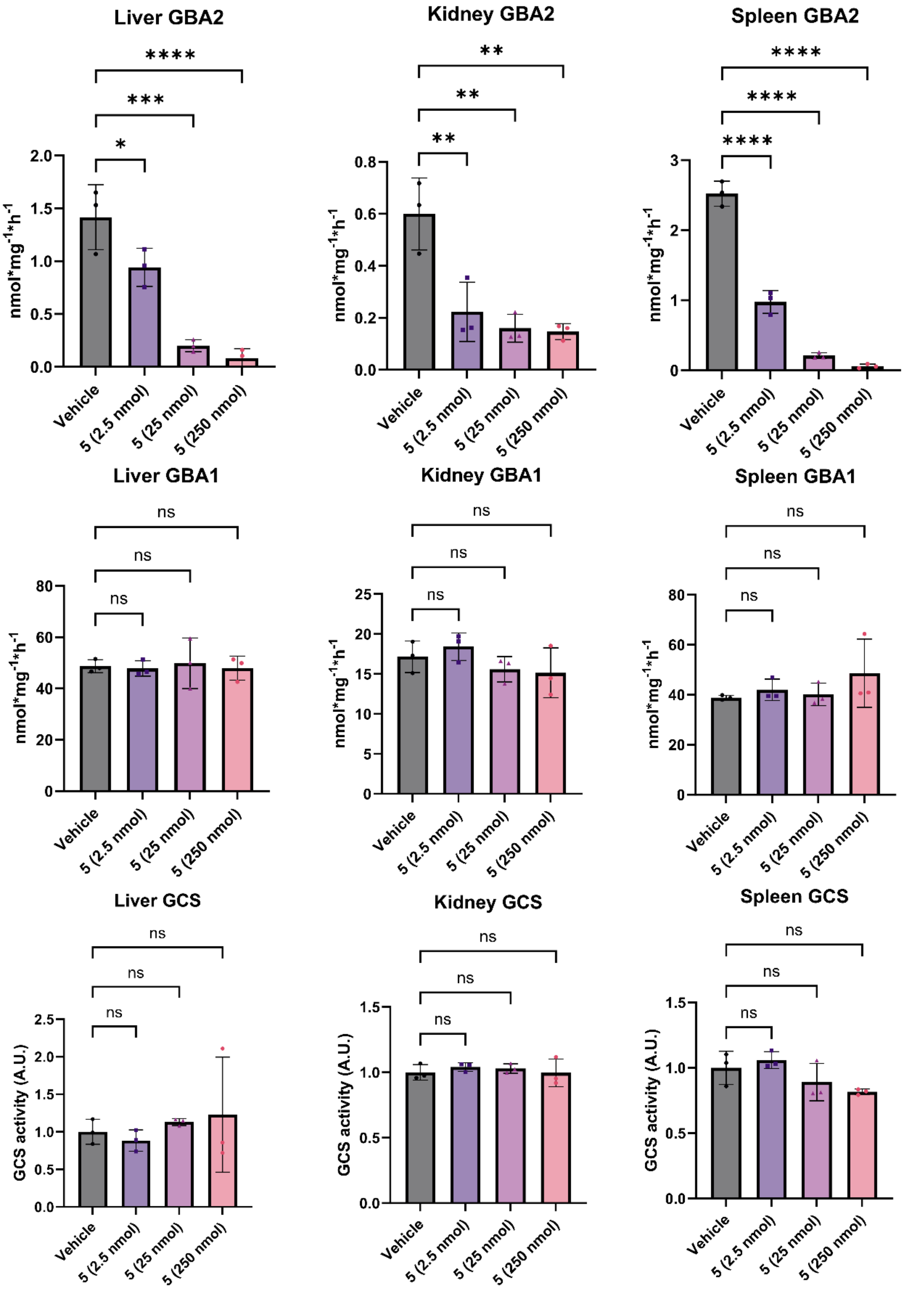

Figure S8. Quantified GBA2 and GBA1 enzyme activities using 4MU-based fluorogenic assays and quantified GCS enzyme activities using NBD-based fluorescence assays, in mouse tissue homogenates after treatment *N*-alkylated aziridine **5**. Mice were treated intravenously with 0, 2.5, 25, or 250 nmol of compound **5** and killed after 3 h.

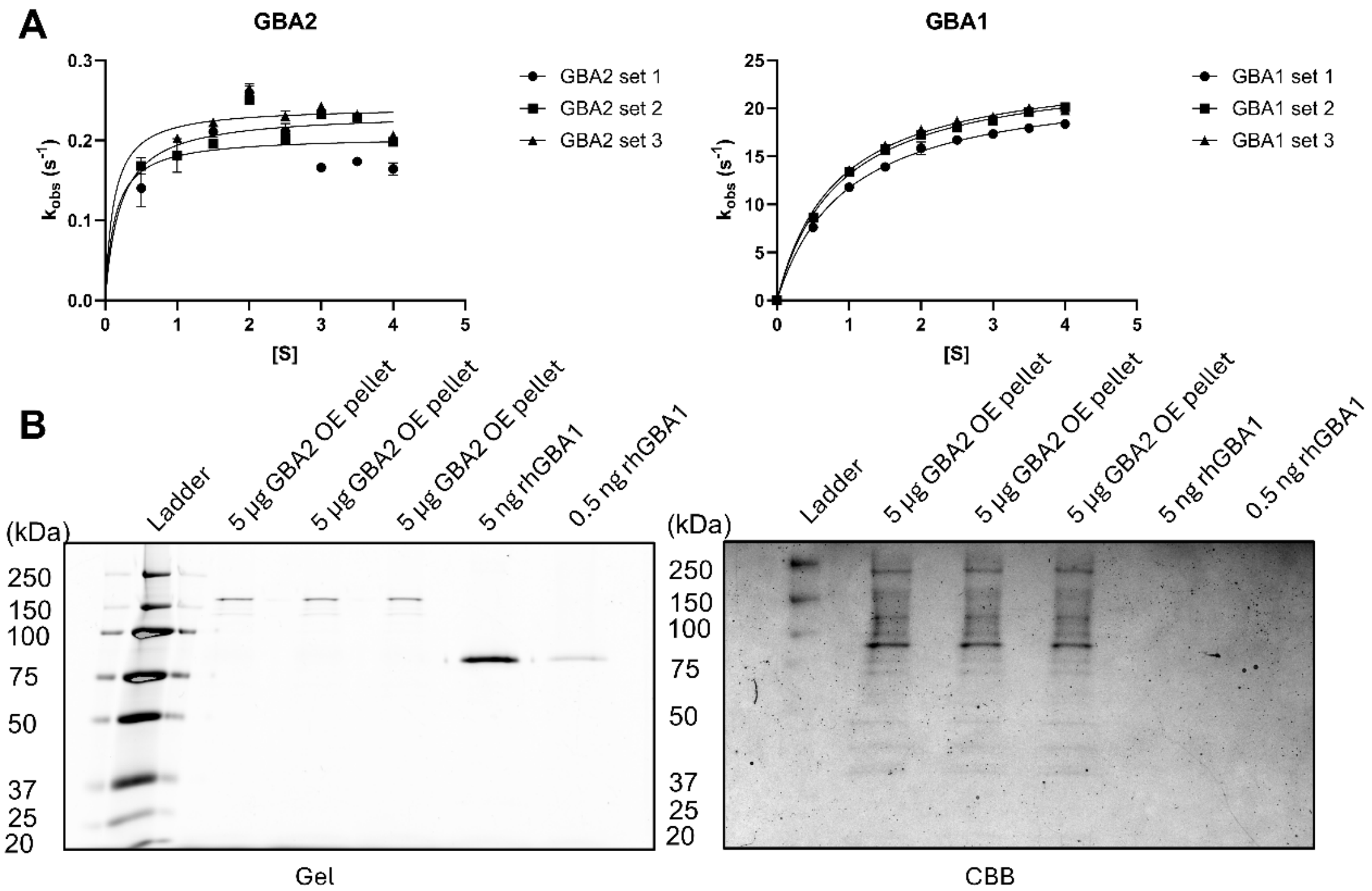

Figure S9. Michaelis Menten kinetics of GBA1 and GBA2 using fluorogenic substrates. For quantification of GBA2 enzyme amounts, equivalent labeling stoichiometry between both enzymes is assumed.

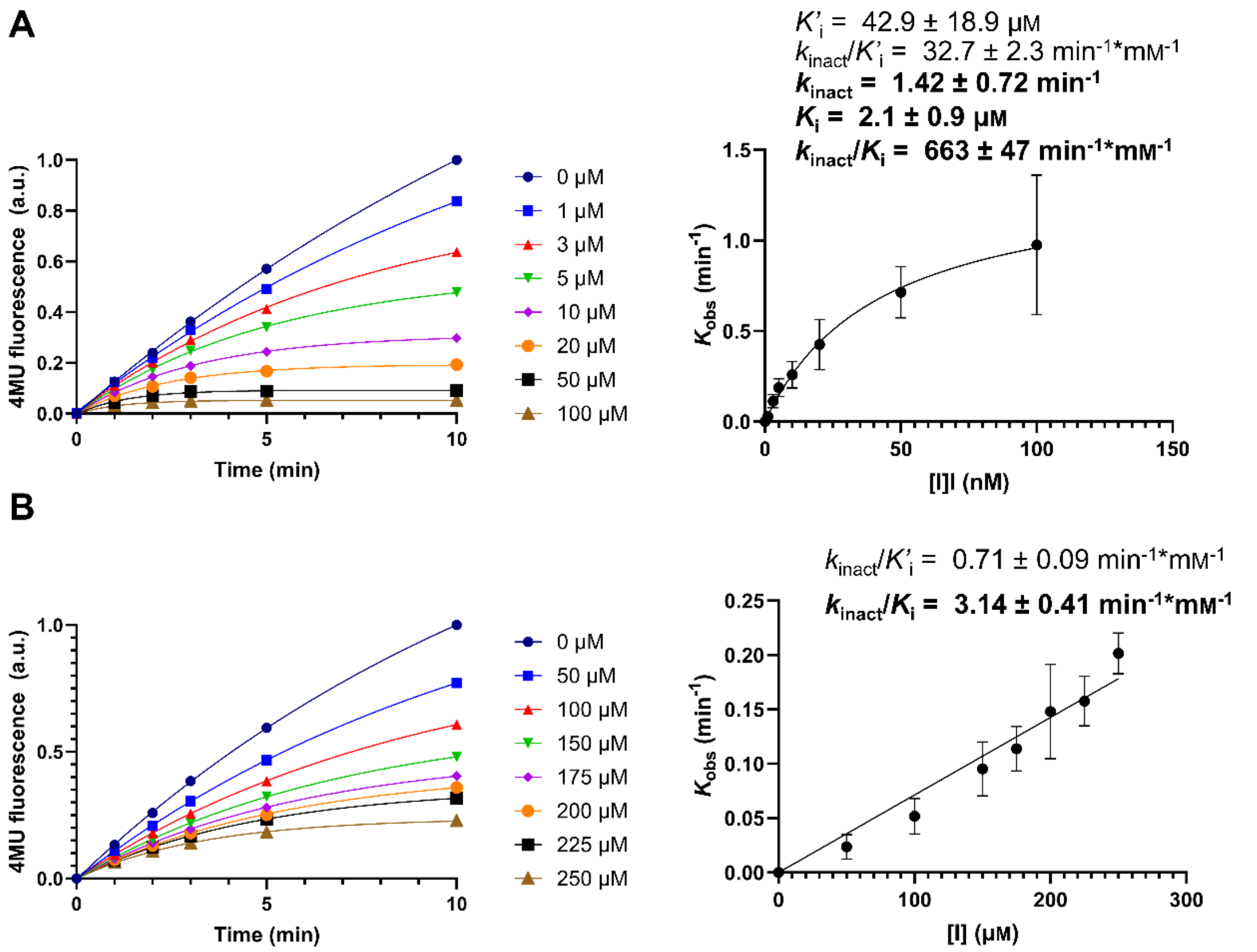

Figure S10. Determination of inhibition kinetics of compound **5** using fluorogenic substrates. The left panels are representative experiments. The right panels show the average, standard deviations and fitting curves for three independent experiments. **A**) Inhibition kinetics of GBA2. **B**) Inhibition kinetics of GBA1.

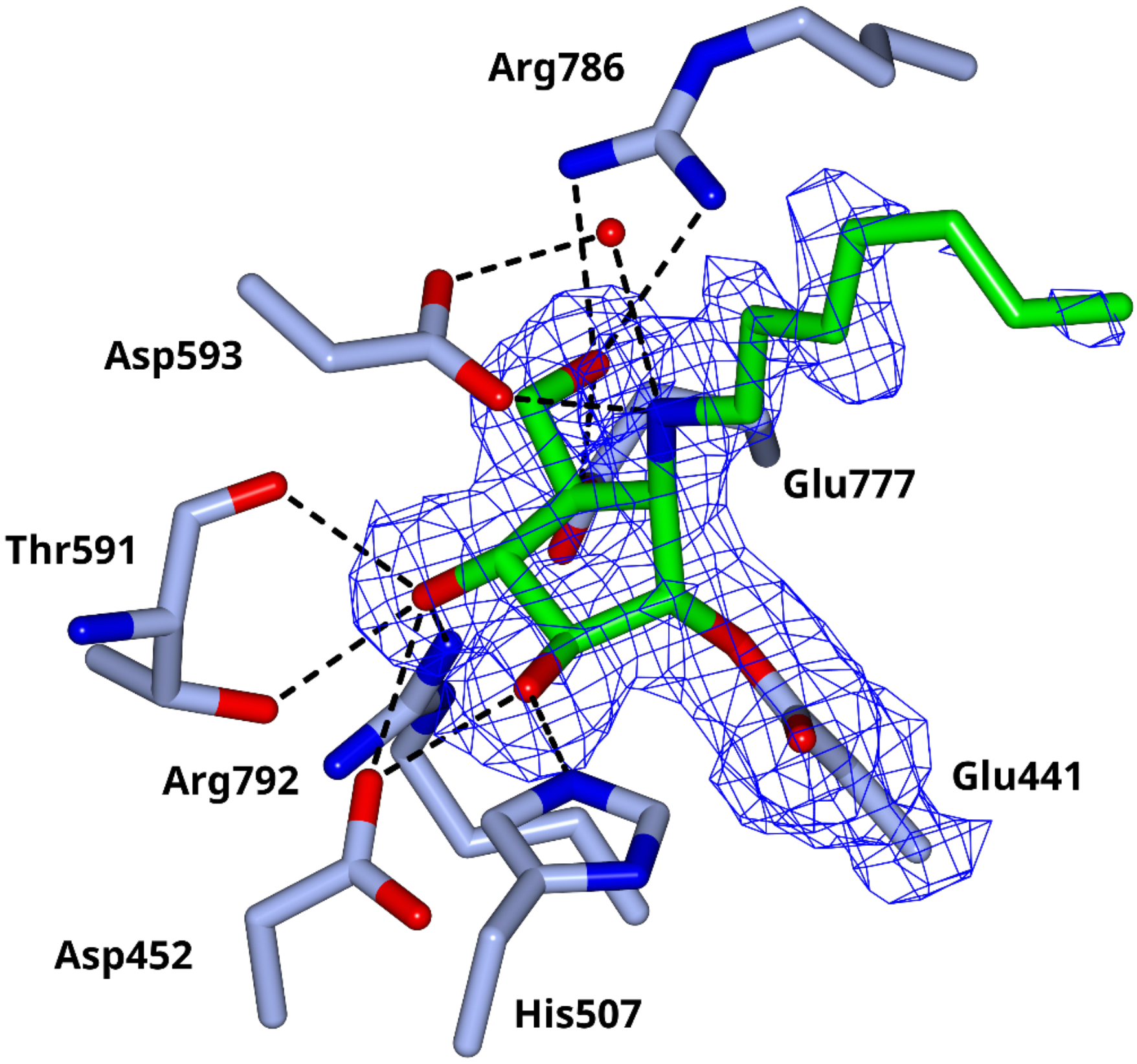

Figure S11. Omit electron density map (*F*o-*F*c) for the aziridine and Glu441 side chain in the *Tx*GH116- **5** structure, contoured at 2.5 rmsd. Carbon atoms are shown in green for the ligand, and ice blue for the neighbouring amino acids. Hydrogen bonds are shown as dashed black lines. The figure was made using *CCP*4*mg*.[33]

Table S5. Data collection and refinement statistics for *Tx*GH116-**5** structure.

**Data collection**	
Space group	*P21 21 2*
Cell dimensions	
a, *b*, *c* (Å)	177.7, 53.9, 83.2
α, β, γ (°)	90.0, 90.0, 90.0
Resolution (Å)*	53.92-1.75 (1.78-1.75)
R_{merge}	0.167 (1.925)
R_{pim}	0.069 (0.801)
$CC_{1/2}$	0.996 (0.633)
I / σ*I*	10.2 (1.3)
Completeness (%)	100.0 (100.0)
Redundancy	12.9 (13.2)
Unique reflections	81586
**Refinement**	
No. reflections	81291
R_{work} / R_{free}	0.18/0.21
No. atoms	
Protein	6221
Ligand/ion**	17 **5**/108 EDO/15 SO4/1 Ca
Water	388
B-factors	
Protein	25.8
Ligand/ion**	25.1 **5**/31.3 EDO/42.2 SO4/40.8 Ca
Water	33.1
R.m.s deviations	
Bond lengths (Å)	0.011
Bond angles (°)	1.875
Ramachandran plot residues	
In most favorable regions (%)	95.9
In allowed regions (%)	3.9
PDB code	30PV

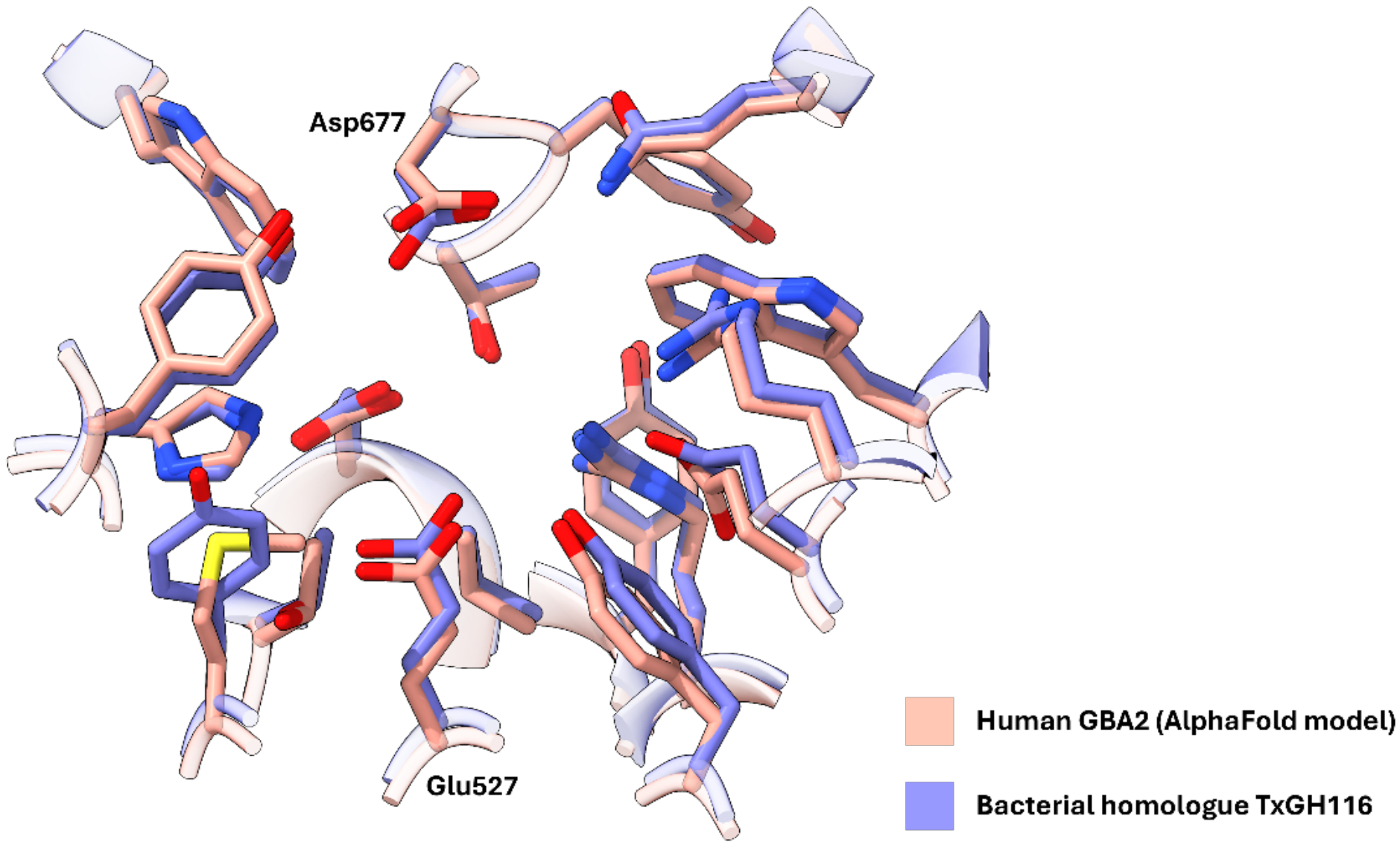

Figure S12. Structural overlay of human GBA2 (Alphafold model) and *Tx*GH116 (crystal structure; PDB code: 8R1M).

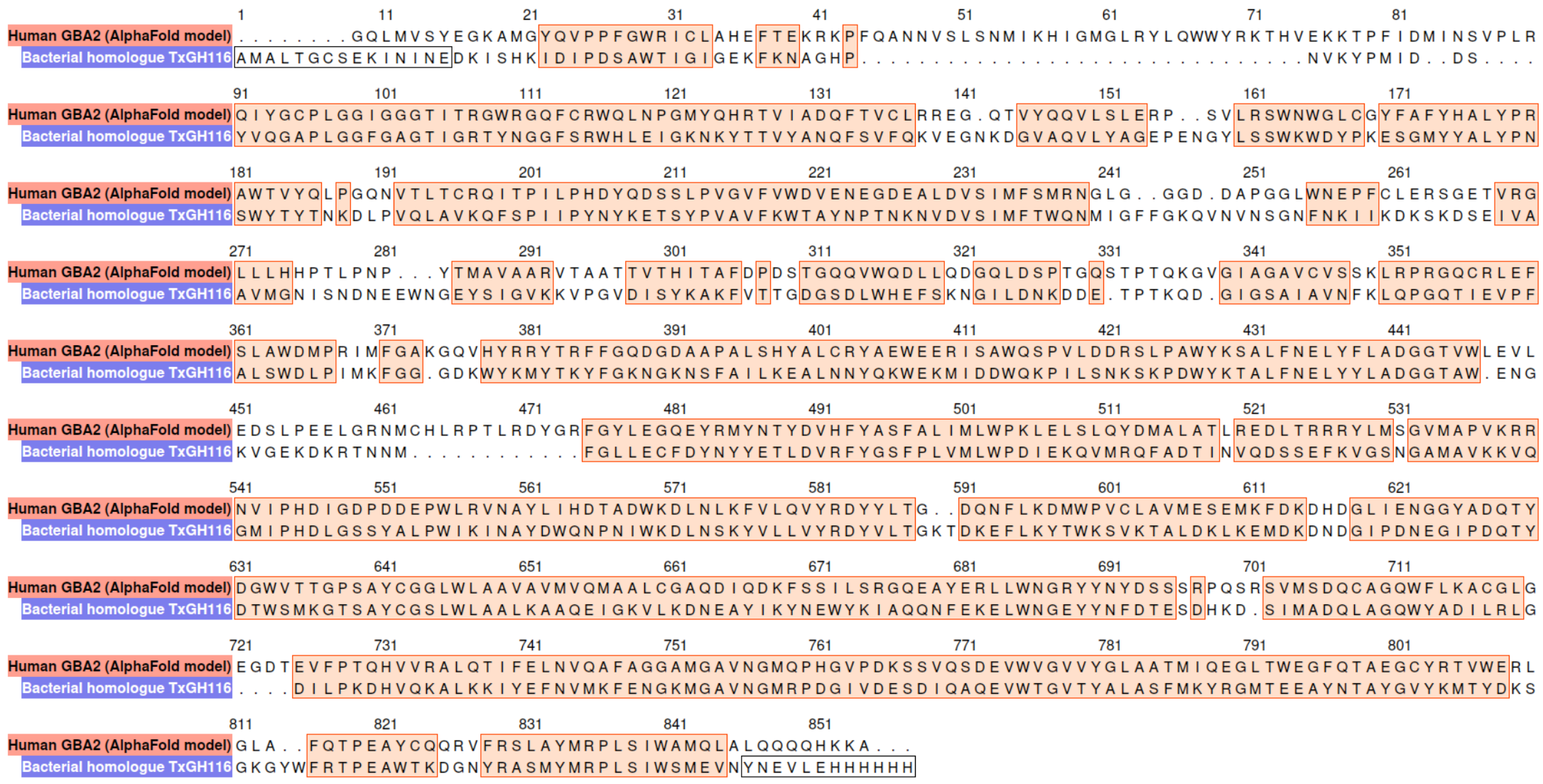

Figure S13. Sequence homology of human GBA2 (Alphafold model) and *Tx*GH116.

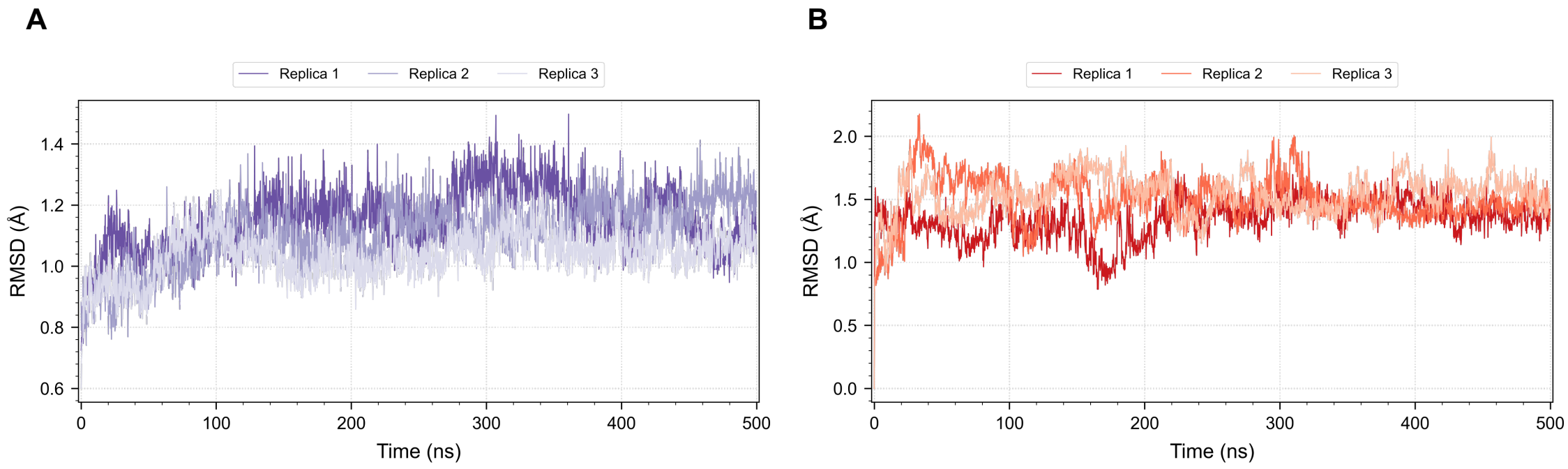

Figure S14. Root-mean-square deviation (RMSD) plots of the protein backbone atoms along the MD simulation, for systems (**A**) GBA1 and (**B**) GBA2 in complex with compound **5**. Each graph shows data from the three replicas.

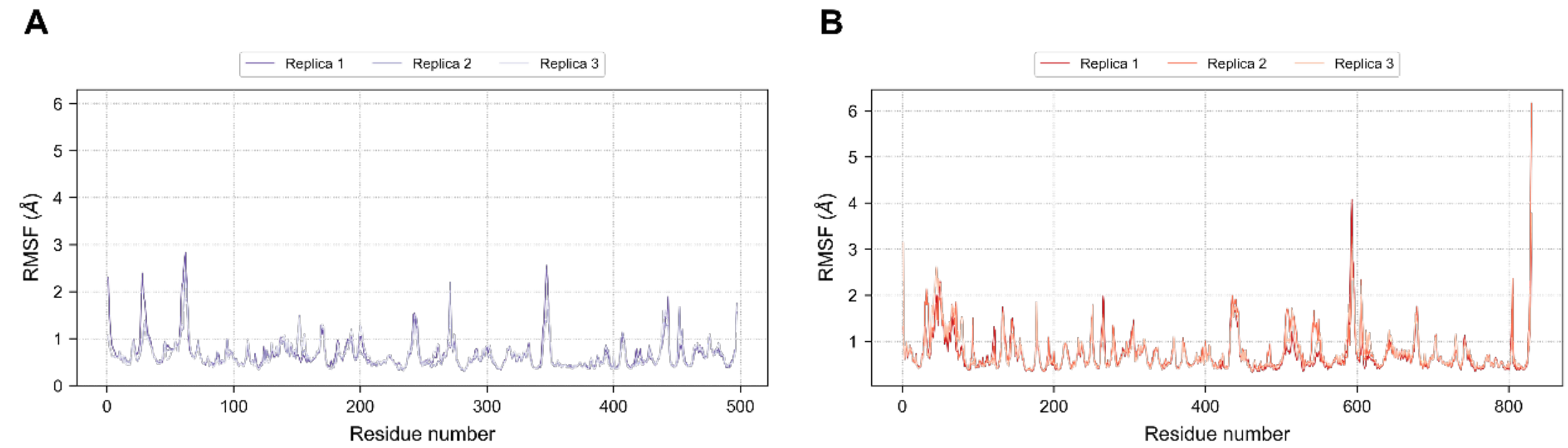

Figure S15. Root-mean-square fluctuation (RMSF) plots of the protein backbone atoms along the MD simulation, for systems (**A**) GBA1 and (**B**) GBA2 in complex with compound **5**. Each graph shows data from the three replicas.

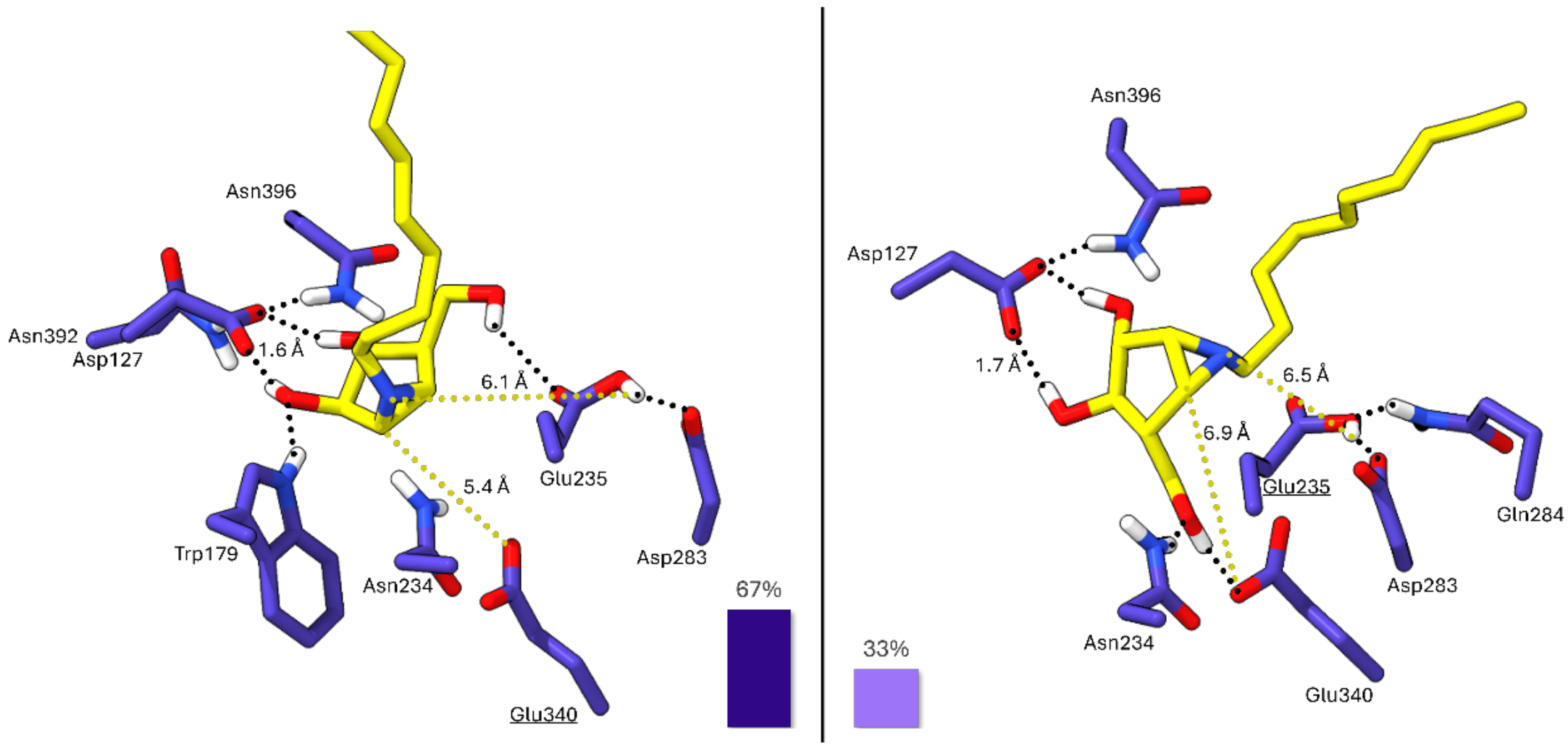

Figure S16. Representative configurations of the two most populated clusters obtained from MD simulations of GBA1 in complex with compound **5**. Hydrogen bonds are shown as black dotted lines, whereas distances between atoms involved in bond formation or bond cleavage during the labeling reaction are shown as yellow dotted lines.

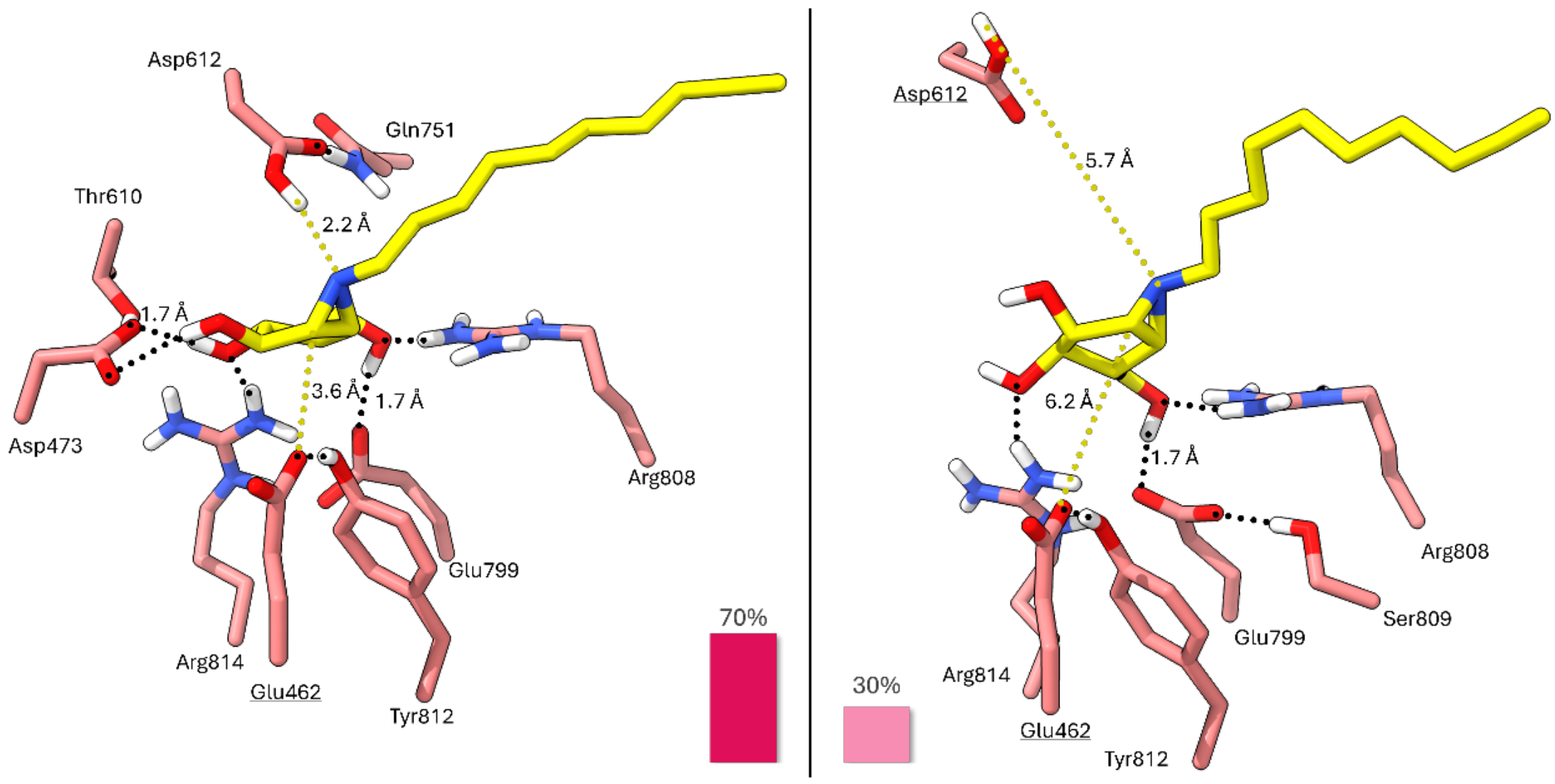

Figure S17. Representative configurations of the two most populated clusters obtained from MD simulations of GBA2 in complex with compound **5**. Hydrogen bonds are shown as black dotted lines, whereas distances between atoms involved in bond formation or bond cleavage during the labeling reaction are shown as yellow dotted lines.

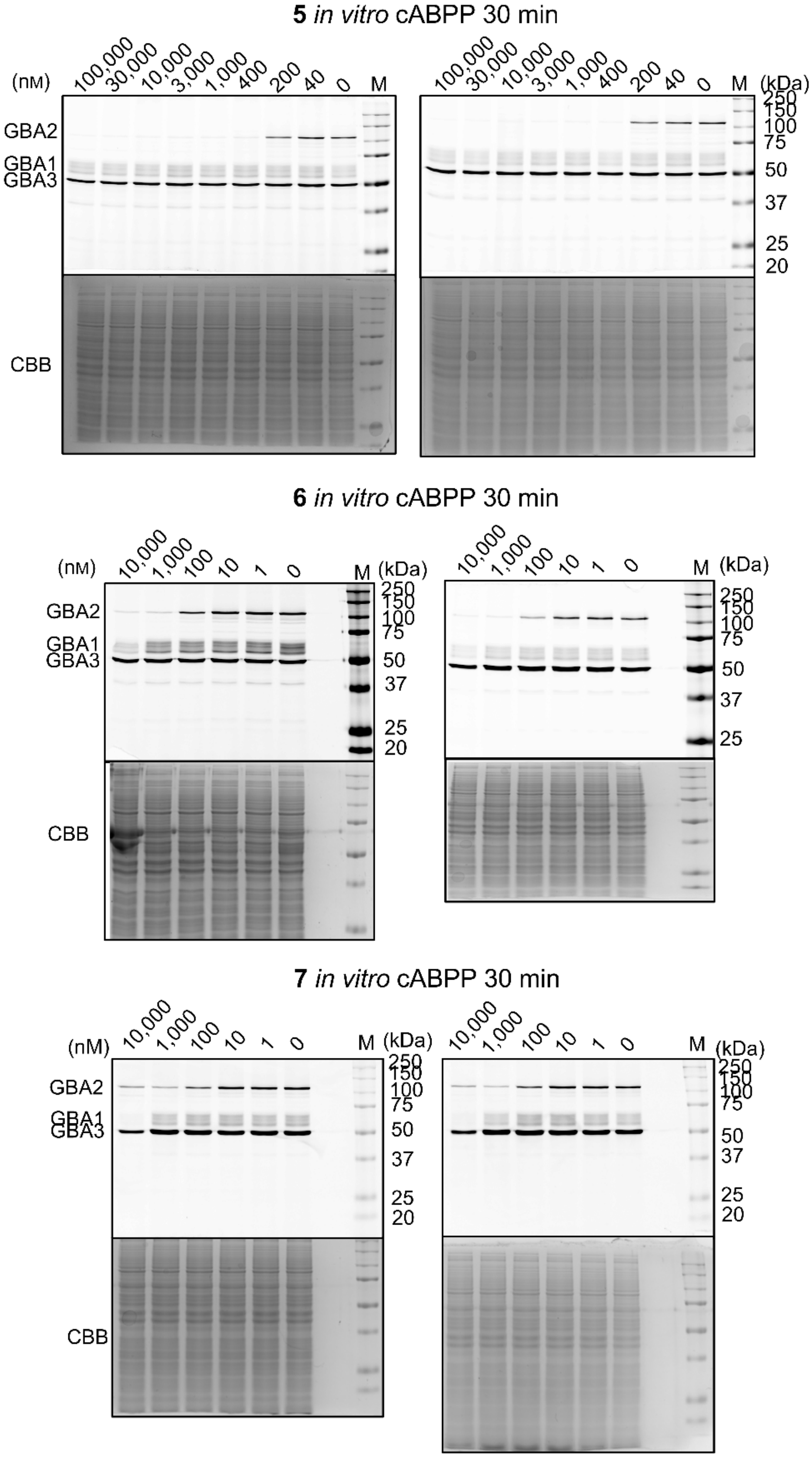

Figure S18. (1/2) Uncropped gels and Coomassie brilliant blue (CBB) staining of *in vitro* labeling of β-glucosidases for 30 minutes with broad-spectrum β-glucosidase ABP **23** after 30 minutes incubation with the inhibitor. HEK293T cells GBA1 endogeneous and GBA2/GBA3 OE lysate was used as the enzyme source.

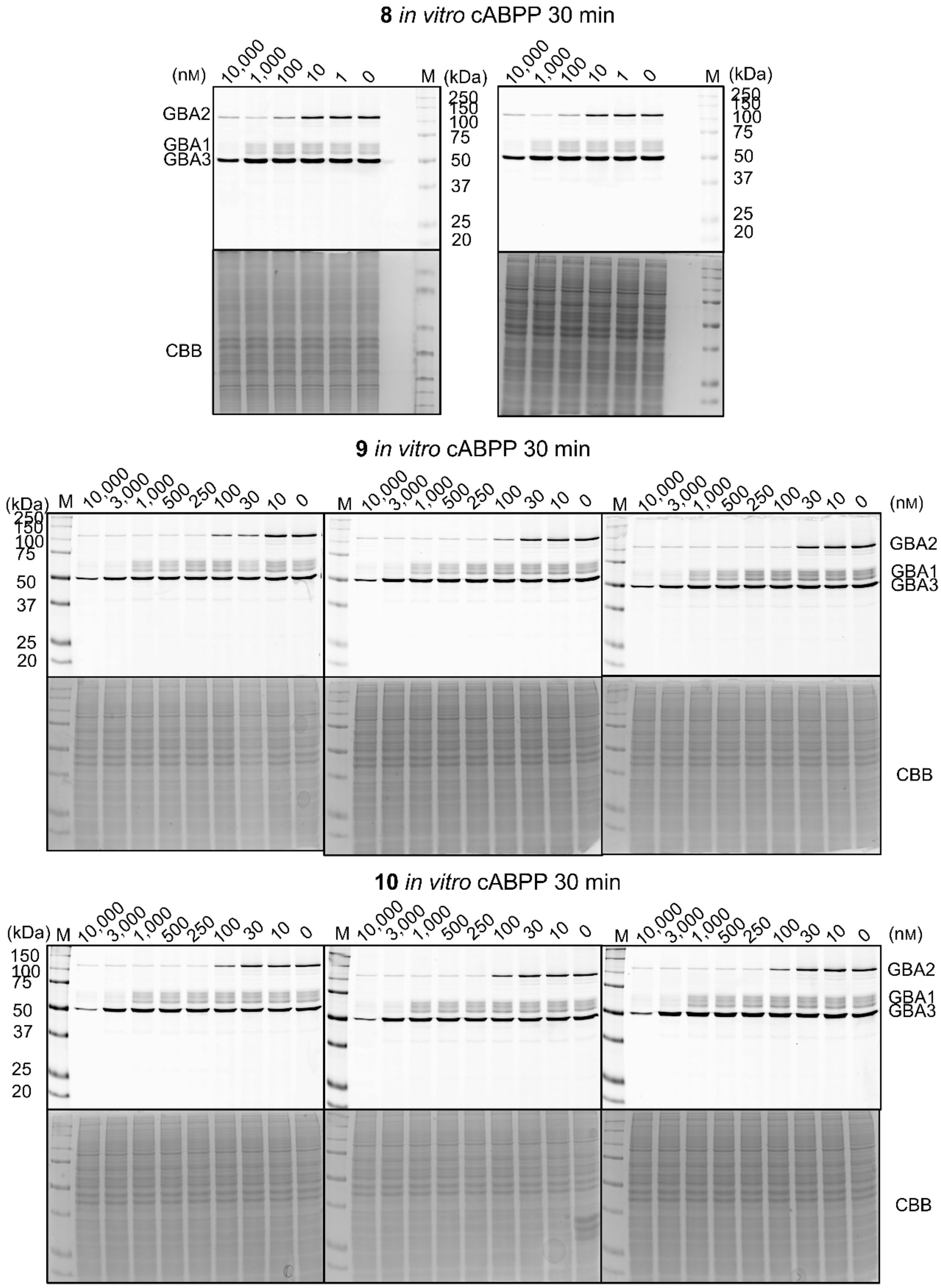

Figure S18. (2/2) Uncropped gels and Coomassie brilliant blue (CBB) staining of *in vitro* labeling of β-glucosidases for 30 minutes with broad-spectrum β-glucosidase ABP **23** after 30 minutes incubation with the inhibitor. HEK293T cells GBA1 endogeneous and GBA2/GBA3 OE lysate was used as the enzyme source.

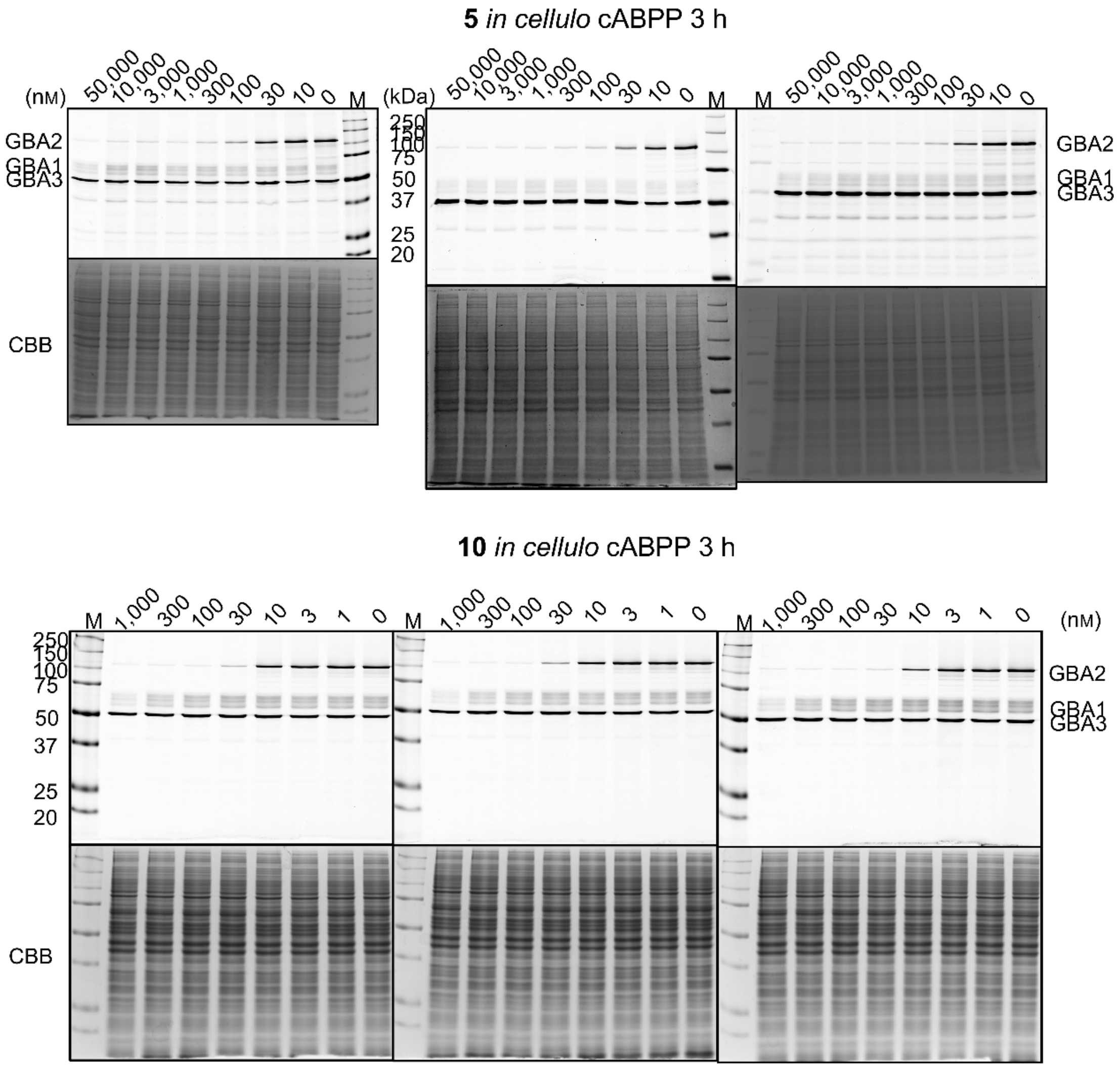

Figure S19. Uncropped gels and Coomassie brilliant blue (CBB) staining of *in cellulo* competitive ABPP assays using intact living HEK293T cells containing endogenous GBA1 and overexpressed GBA2/GBA3.

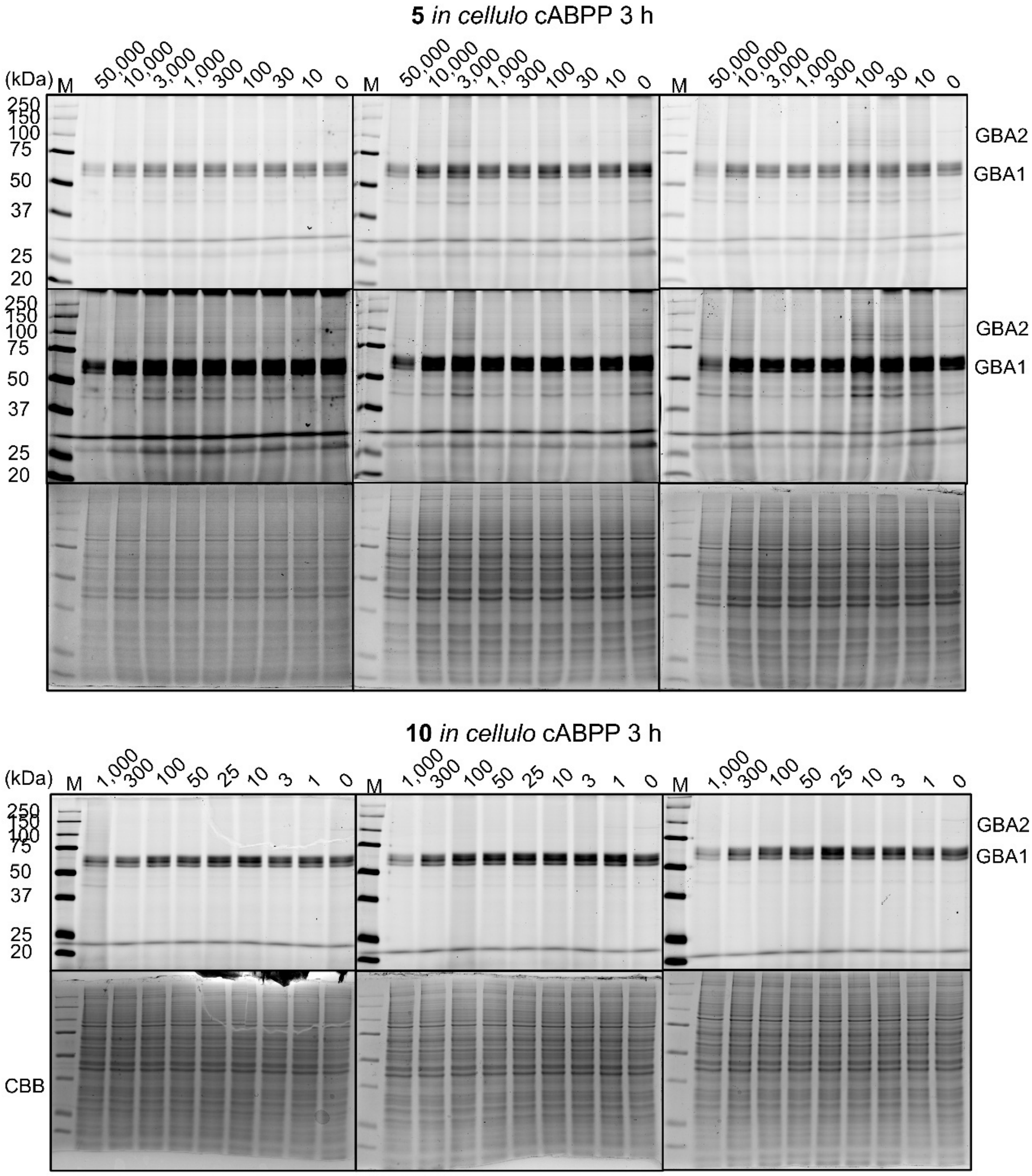

Figure S20. Uncropped gels and Coomassie brilliant blue (CBB) staining of *in cellulo* competitive ABPP assays using intact living wild-type HEK293T cells containing endogenous GBA1 and GBA2. For compound **5**, the lower panel is shown with enhanced contrast to make the GBA2 band more visible.

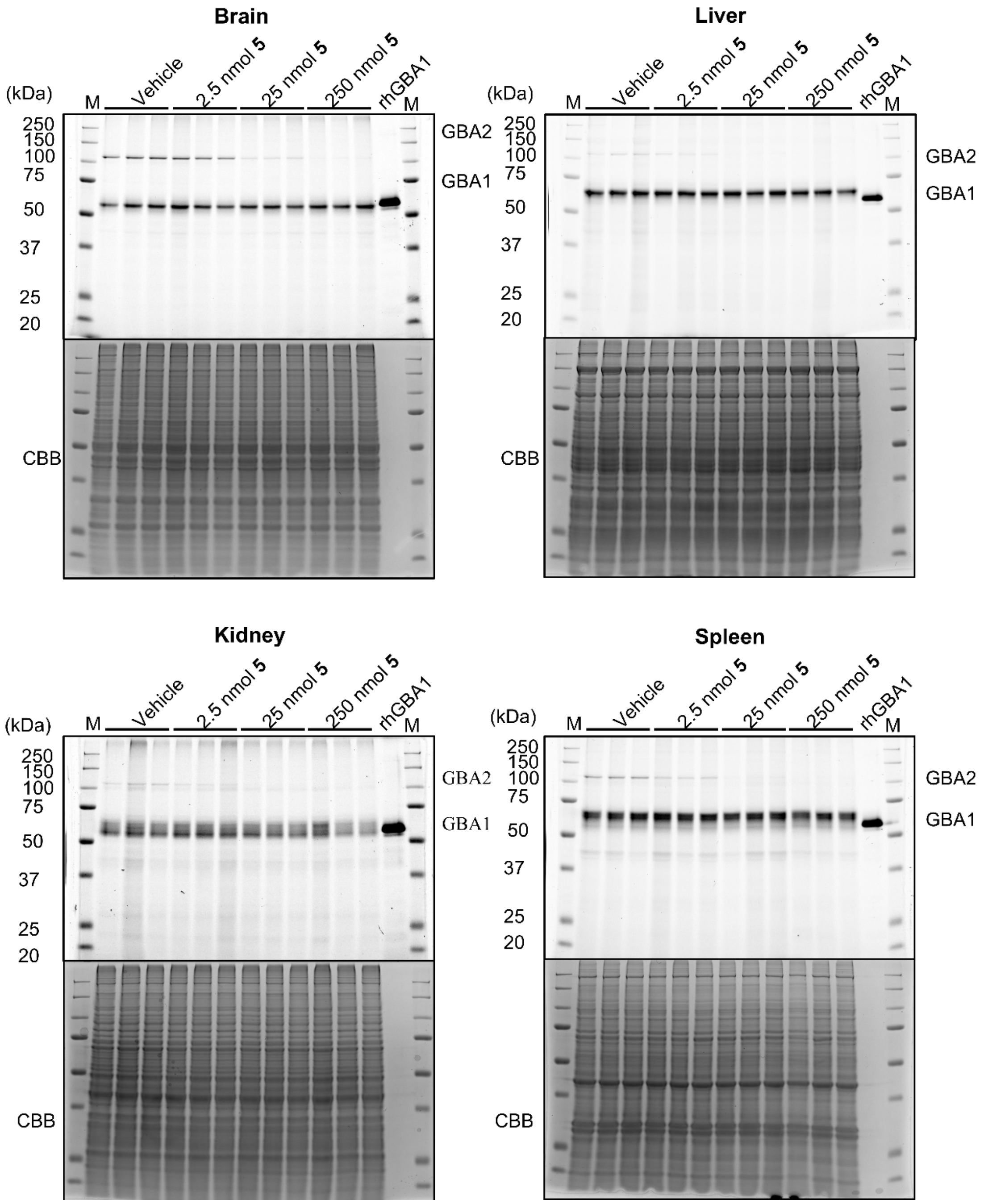

Figure S21. Uncropped gels and Coomassie brilliant blue (CBB) staining of *in vivo* competitive ABPP assays in murine tissues.

References

(1) Marques, A. A.; Mirzaian, M.; Akiyama, H.; Wisse, P.; Ferraz, M. J.; Gaspar, P.; Ghauharali-van der Vlugt, K.; Meijer, R.; Giraldo, P.; Alfonso, P.; Irún, P.; Dahl, M.; Karlsson, S.; Pavlova, E. V.; Cox, T. M.; Scheij, S.; Verhoek, M.; Ottenhoff, R.; van Roomen, C. A. A.; Pannu, N. S.; van Eijk, M.; Dekker, N.; Boot, R. G.; Overkleeft, H. S.; Blommaart, E.; Hirabayashi, Y.; Aerts, J. M. Glucosylated cholesterol in mammalian cells and tissues: Formation and degradation by multiple cellular β-glucosidases. *J. Lipid Res.* **2016**, *57*, 451-463.

(2) Su, Q.; Schröder, S. P.; Lelieveld, L. T.; Ferraz, M. J.; Verhoek, M.; Boot, R. G.; Overkleeft, H. S.; Aerts, J. M. F. G.; Artola, M.; Kuo, C.-L. Xylose-configured cyclophellitols as selective inhibitors for glucocerebrosidase. *ChemBioChem* **2021**, *22*, 3090-3098.

(3) Kuo, C.-L.; van Meel, E.; Kytidou, K.; Kallemeijn, W. W.; Witte, M.; Overkleeft, H. S.; Artola, M. E.; Aerts, J. M. Activity-based probes for glycosidases: Profiling and other applications. In *Methods enzymol.*, Vol. 598; 2018; pp 217-235.

(4) Kuo, C.-L.; Kallemeijn, W. W.; Lelieveld, L. T.; Mirzaian, M.; Zoutendijk, I.; Vardi, A.; Futerman, A. H.; Meijer, A. H.; Spaink, H. P.; Overkleeft, H. S.; Aerts, J. M. F. G.; Artola, M. In vivo inactivation of glycosidases by conduritol b epoxide and cyclophellitol as revealed by activity-based protein profiling. *FEBS J.* **2019**, *286*, 584-600.

(5) Wu, L.; Jiang, J.; Jin, Y.; Kallemeijn, W. W.; Kuo, C.-L.; Artola, M.; Dai, W.; van Elk, C.; van Eijk, M.; van der Marel, G. A.; Codée, J. D. C.; Florea, B. I.; Aerts, J. M. F. G.; Overkleeft, H. S.; Davies, G. J. Activity-based probes for functional interrogation of retaining β-glucuronidases. *Nature Chemical Biology* **2017**, *13*, 867-873.

(6) Schröder, S. P.; van de Sande, J. W.; Kallemeijn, W. W.; Kuo, C.-L.; Artola, M.; van Rooden, E. J.; Jiang, J.; Beenakker, T. J. M.; Florea, B. I.; Offen, W. A.; Davies, G. J.; Minnaard, A. J.; Aerts, J. M. F. G.; Codée, J. D. C.; van der Marel, G. A.; Overkleeft, H. S. Towards broad spectrum activity-based glycosidase probes: Synthesis and evaluation of deoxygenated cyclophellitol aziridines. *Chem. Commun.* **2017**, *53*, 12528-12531.

(7) Charoenwattanasatien, R.; Pengthaisong, S.; Breen, I.; Mutoh, R.; Sansenya, S.; Hua, Y.; Tankrathok, A.; Wu, L.; Songsiririthigul, C.; Tanaka, H.; Williams, S. J.; Davies, G. J.; Kurisu, G.; Cairns, J. R. K. Bacterial β-glucosidase reveals the structural and functional basis of genetic defects in human glucocerebrosidase 2 (GBA2). *ACS Chem. Biol.* **2016**, *11*, 1891-1900.

(8) Waterman, D. G.; Winter, G.; Gildea, R. J.; Parkhurst, J. M.; Brewster, A. S.; Sauter, N. K.; Evans, G. Diffraction-geometry refinement in the DIALS framework. *Acta Crystallogr. D* **2016**, *72*, 558-575.

(9) Evans, P. R.; Murshudov, G. N. How good are my data and what is the resolution? *Acta Crystallogr. D* **2013**, *69*, 1204-1214.

(10) McCoy, A. J.; Grosse-Kunstleve, R. W.; Adams, P. D.; Winn, M. D.; Storoni, L. C.; Read, R. J. Phaser crystallographic software. *J. Appl. Crystallogr.* **2007**, *40*, 658-674.

(11) Murshudov, G. N.; Skubak, P.; Lebedev, A. A.; Pannu, N. S.; Steiner, R. A.; Nicholls, R. A.; Winn, M. D.; Long, F.; Vagin, A. A. REFMAC5 for the refinement of macromolecular crystal structures. *Acta Crystallogr. D* **2011**, *67*, 355-367.

(12) Emsley, P.; Lohkamp, B.; Scott, W. G.; Cowtan, K. Features and development of Coot. *Acta Crystallogr. D* **2010**, *66*, 486-501.

(13) Krissinel, E.; Lebedev, A. A.; Uski, V.; Ballard, C. B.; Keegan, R. M.; Kovalevskiy, O.; Nicholls, R. A.; Pannu, N. S.; Skubak, P.; Berrisford, J.; Fando, M.; Lohkamp, B.; Wojdyr, M.; Simpkin, A. J.;

Thomas, J. M. H.; Oliver, C.; Vonrhein, C.; Chojnowski, G.; Basle, A.; Purkiss, A.; Isupov, M. N.; McNicholas, S.; Lowe, E.; Trivino, J.; Cowtan, K.; Agirre, J.; Rigden, D. J.; Uson, I.; Lamzin, V.; Tews, I.; Bricogne, G.; Leslie, A. G. W.; Brown, D. G. CCP4 cloud for structure determination and project management in macromolecular crystallography. *Acta Crystallogr. D* **2022**, *78*, 1079-1089.
(14) Rowland, R. J.; Wu, L.; Liu, F.; Davies, G. J. A baculoviral system for the production of human [beta]-glucocerebrosidase enables atomic resolution analysis. *Acta Crystallogr. D* **2020**, *76*, 565-580.
(15) Passaro, S.; Corso, G.; Wohlwend, J.; Reveiz, M.; Thaler, S.; Somnath, V. R.; Getz, N.; Portnoi, T.; Roy, J.; Stark, H.; Kwabi-Addo, D.; Beaini, D.; Jaakkola, T.; Barzilay, R. Boltz-2: Towards accurate and efficient binding affinity prediction. *bioRxiv* **2025**, 2025.2006.2014.659707.
(16) ByteDance, A. M. L. A. I. S. T.; Chen, X.; Zhang, Y.; Lu, C.; Ma, W.; Guan, J.; Gong, C.; Yang, J.; Zhang, H.; Zhang, K.; Wu, S.; Zhou, K.; Yang, Y.; Liu, Z.; Wang, L.; Shi, B.; Shi, S.; Xiao, W. Protenix - advancing structure prediction through a comprehensive AlphaFold3 reproduction. *bioRxiv* **2025**, 2025.2001.2008.631967.
(17) Anandakrishnan, R.; Aguilar, B.; Onufriev, A. V. H++ 3.0: Automating pK prediction and the preparation of biomolecular structures for atomistic molecular modeling and simulations. *Nucleic Acids Res.* **2012**, *40*, W537-W541.
(18) Case, D. A.; Aktulga, H. M.; Belfon, K.; Cerutti, D. S.; Cisneros, G. A.; Cruzeiro, V. W. D.; Forouzesh, N.; Giese, T. J.; Götz, A. W.; Gohlke, H.; Izadi, S.; Kasavajhala, K.; Kaymak, M. C.; King, E.; Kurtzman, T.; Lee, T.-S.; Li, P.; Liu, J.; Luchko, T.; Luo, R.; Manathunga, M.; Machado, M. R.; Nguyen, H. M.; O'Hearn, K. A.; Onufriev, A. V.; Pan, F.; Pantano, S.; Qi, R.; Rahnamoun, A.; Risheh, A.; Schott-Verdugo, S.; Shajan, A.; Swails, J.; Wang, J.; Wei, H.; Wu, X.; Wu, Y.; Zhang, S.; Zhao, S.; Zhu, Q.; Cheatham, T. E., III; Roe, D. R.; Roitberg, A.; Simmerling, C.; York, D. M.; Nagan, M. C.; Merz, K. M., Jr. AmberTools. *J. Chem. Inf. Model.* **2023**, *63*, 6183-6191.
(19) D.A. Case, H. M. A., K. Belfon, I.Y. Ben-Shalom, J.T. Berryman, S.R. Brozell, F.S. Carvahol, D.S. Cerutti, T.E. Cheatham, III, G.A. Cisneros, V.W.D. Cruzeiro, T.A. Darden, N. Forouzesh, M. Ghazimirsaeed, G. Giambaşu, T. Giese, M.K. Gilson, H. Gohlke, A.W. Goetz, J. Harris, Z. Huang, S. Izadi, S.A. Izmailov, K. Kasavajhala, M.C. Kaymak, I. Kolossvary, A. Kovalenko, T. Kurtzman, T.S. Lee, P. Li, Z. Li, C. Lin, J. Liu, T. Luchko, R. Luo, M. Machado, M. Manathunga, K.M. Merz, Y. Miao, O. Mikhailovskii, G. Monard, H. Nguyen, K.A. O'Hearn, A. Onufriev, F. Pan, S. Pantano, A. Rahnamoun, D.R. Roe, A. Roitberg, C. Sagui, S. Schott-Verdugo, A. Shajan, J. Shen, C.L. Simmerling, N.R. Skrynnikov, J. Smith, J. Swails, R.C. Walker, J. Wang, J. Wang, X. Wu, Y. Wu, Y. Xiong, Y. Xue, D.M. York, C. Zhao, Q. Zhu, P.A. Kollman. *Amber 2025*; University of California, San Francisco., 2025.
(20) Maier, J. A.; Martinez, C.; Kasavajhala, K.; Wickstrom, L.; Hauser, K. E.; Simmerling, C. ff14SB: Improving the accuracy of protein side chain and backbone parameters from ff99sb. *J. Chem. Theory Comput.* **2015**, *11*, 3696-3713.
(21) Jorgensen, W. L.; Chandrasekhar, J.; Madura, J. D.; Impey, R. W.; Klein, M. L. Comparison of simple potential functions for simulating liquid water. *J. Chem. Phys.* **1983**, *79*, 926-935.
(22) Wang, J.; Wolf, R. M.; Caldwell, J. W.; Kollman, P. A.; Case, D. A. Development and testing of a general amber force field. *J. Comput. Chem.* **2004**, *25*, 1157-1174.
(23) Frisch, M. J.; Trucks, G. W.; Schlegel, H. B.; Scuseria, G. E.; Robb, M. A.; Cheeseman, J. R.; Scalmani, G.; Barone, V.; Petersson, G. A.; Nakatsuji, H.; Li, X.; Caricato, M.; Marenich, A. V.; Bloino, J.; Janesko, B. G.; Gomperts, R.; Mennucci, B.; Hratchian, H. P.; Ortiz, J. V.; Izmaylov, A. F.; Sonnenberg, J. L.; Williams; Ding, F.; Lipparini, F.; Egidi, F.; Goings, J.; Peng, B.; Petrone, A.; Henderson, T.; Ranasinghe, D.; Zakrzewski, V. G.; Gao, J.; Rega, N.; Zheng, G.; Liang, W.; Hada, M.;

Ehara, M.; Toyota, K.; Fukuda, R.; Hasegawa, J.; Ishida, M.; Nakajima, T.; Honda, Y.; Kitao, O.; Nakai, H.; Vreven, T.; Throssell, K.; Montgomery Jr., J. A.; Peralta, J. E.; Ogliaro, F.; Bearpark, M. J.; Heyd, J. J.; Brothers, E. N.; Kudin, K. N.; Staroverov, V. N.; Keith, T. A.; Kobayashi, R.; Normand, J.; Raghavachari, K.; Rendell, A. P.; Burant, J. C.; Iyengar, S. S.; Tomasi, J.; Cossi, M.; Millam, J. M.; Klene, M.; Adamo, C.; Cammi, R.; Ochterski, J. W.; Martin, R. L.; Morokuma, K.; Farkas, O.; Foresman, J. B.; Fox, D. J. *Gaussian 16 rev. C.01*; Wallingford, CT, 2016.

(24) Ferrier, R. J. Unsaturated carbohydrates. Part 21. A carbocyclic ring closure of a hex-5-enopyranoside derivative. *J. Chem. Soc., Perkin Trans. 1* **1979**, 1455-1458.

(25) Bauder, C. A convenient synthesis of orthogonally protected 2-deoxystreptamine (2-dos) as an aminocyclitol scaffold for the development of novel aminoglycoside antibiotic derivatives against bacterial resistance. *Org. Biomol. Chem.* **2008**, *6*, 2952-2960.

(26) Lopez, O. L.; Fernández-Bolaños, J. G.; Lillelund, V. H.; Bols, M. Aziridines as a structural motif to conformational restriction of azasugars. *Org. Biomol. Chem.* **2003**, *1*, 478-482.

(27) Ferrier, R. J.; Prasit, P.; Gainsford, G. J. Functionalised carbocycles from carbohydrates. Part 3. The synthesis of the epoxy lactone prostaglandin intermediate via an isoxazolidine derivative. X-ray crystal structure of (1R,5R)-6-exo,7-endo-dibenzoyloxy-8-exo-iodo-3-oxo-2-oxabicyclo[3.3.0]octane. *J. Chem. Soc., Perkin Trans. 1* **1983**, 1629-1634.

(28) McGregor, N. G. S.; Coines, J.; Borlandelli, V.; Amaki, S.; Artola, M.; Nin-Hill, A.; Linzel, D.; Yamada, C.; Arakawa, T.; Ishiwata, A.; Ito, Y.; van der Marel, G. A.; Codée, J. D. C.; Fushinobu, S.; Overkleeft, H. S.; Rovira, C.; Davies, G. J. Cysteine nucleophiles in glycosidase catalysis: Application of a covalent β-l-arabinofuranosidase inhibitor. *Angew. Chem. Int. Ed.* **2021**, *60*, 5754-5758.

(29) Wennekes, T.; van den Berg, R. J. B. H. N.; Donker, W.; van der Marel, G. A.; Strijland, A.; Aerts, J. M. F. G.; Overkleeft, H. S. Development of adamantan-1-yl-methoxy-functionalized 1-deoxynojirimycin derivatives as selective inhibitors of glucosylceramide metabolism in man. *J. Org. Chem.* **2007**, *72*, 1088-1097.

(30) De Fenza, M.; D'Alonzo, D.; Esposito, A.; Munari, S.; Loberto, N.; Santangelo, A.; Lampronti, I.; Tamanini, A.; Rossi, A.; Ranucci, S.; De Fino, I.; Bragonzi, A.; Aureli, M.; Bassi, R.; Tironi, M.; Lippi, G.; Gambari, R.; Cabrini, G.; Palumbo, G.; Dechecchi, M. C.; Guaragna, A. Exploring the effect of chirality on the therapeutic potential of N-alkyl-deoxyiminosugars: Anti-inflammatory response to pseudomonas aeruginosa infections for application in cf lung disease. *Eur. J. Med. Chem.* **2019**, *175*, 63-71.

(31) Ghisaidoobe, A. T.; van den Berg, R. J. B. H. N.; Butt, S. S.; Strijland, A.; Donker-Koopman, W. E.; Scheij, S.; van den Nieuwendijk, A. M. C. H.; Koomen, G.-J.; van Loevezijn, A.; Leemhuis, M.; Wennekes, T.; van der Stelt, M.; van der Marel, G. A.; van Boeckel, C. A. A.; Aerts, J. M. F. G.; Overkleeft, H. S. Identification and development of biphenyl substituted iminosugars as improved dual glucosylceramide synthase/neutral glucosylceramidase inhibitors. *J. Med. Chem.* **2014**, *57*, 9096-9104.

(32) Artola, M.; Kuo, C.-L.; Lelieveld, L. T.; Rowland, R. J.; van der Marel, G. A.; Codée, J. D. C.; Boot, R. G.; Davies, G. J.; Aerts, J. M. F. G.; Overkleeft, H. S. Functionalized cyclophellitols are selective glucocerebrosidase inhibitors and induce a bona fide neuropathic Gaucher model in zebrafish. *J. Am. Chem. Soc.* **2019**, *141*, 4214-4218.

(33) McNicholas, S.; Potterton, E.; Wilson, K. S.; Noble, M. E. M. Presenting your structures: The CCP4mg molecular-graphics software. *Acta Crystallogr. D* **2011**, *67*, 386-394.

NMR Spectra

^{1}H-NMR spectrum of compound 12

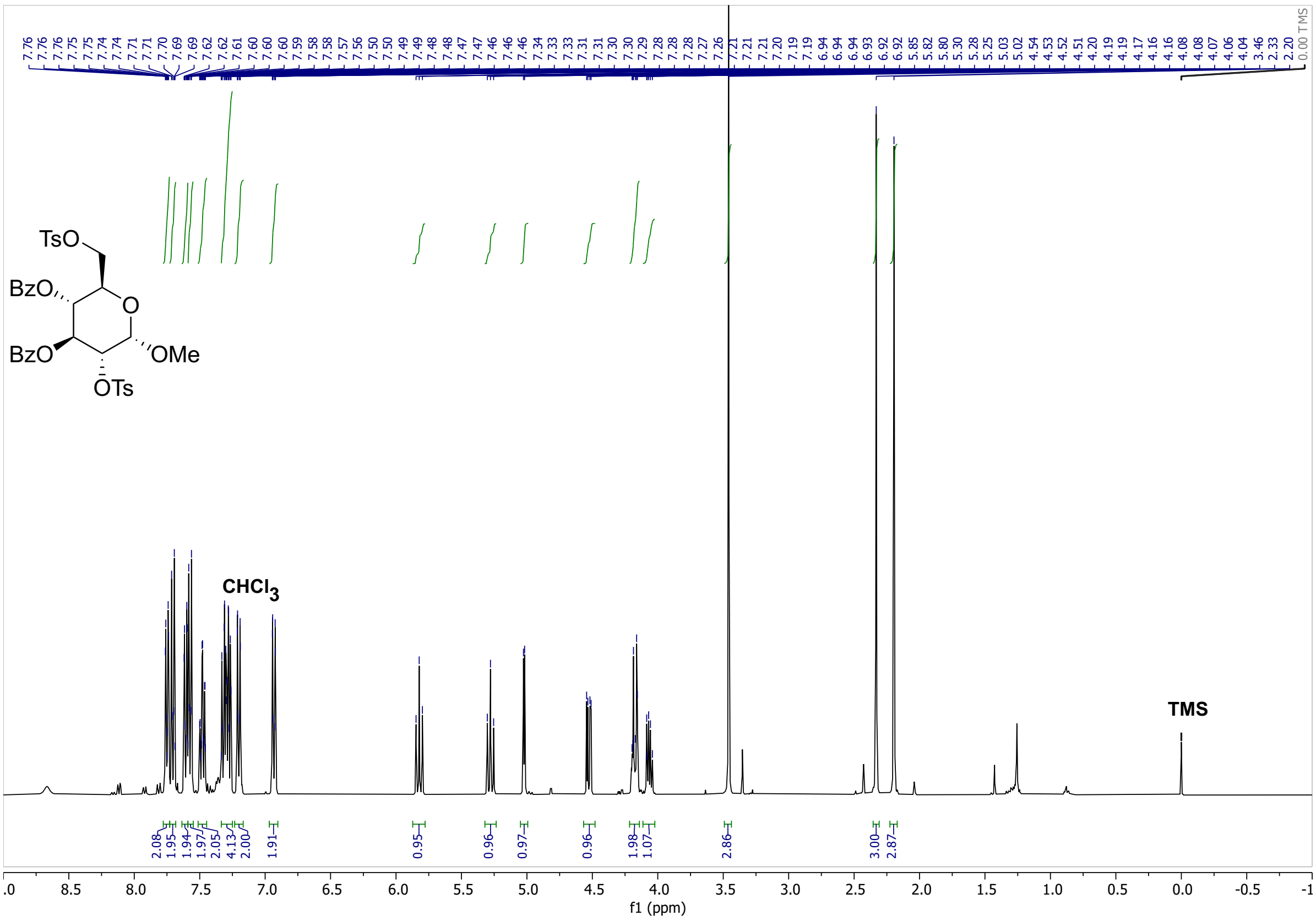

APT-NMR spectrum of compound 12

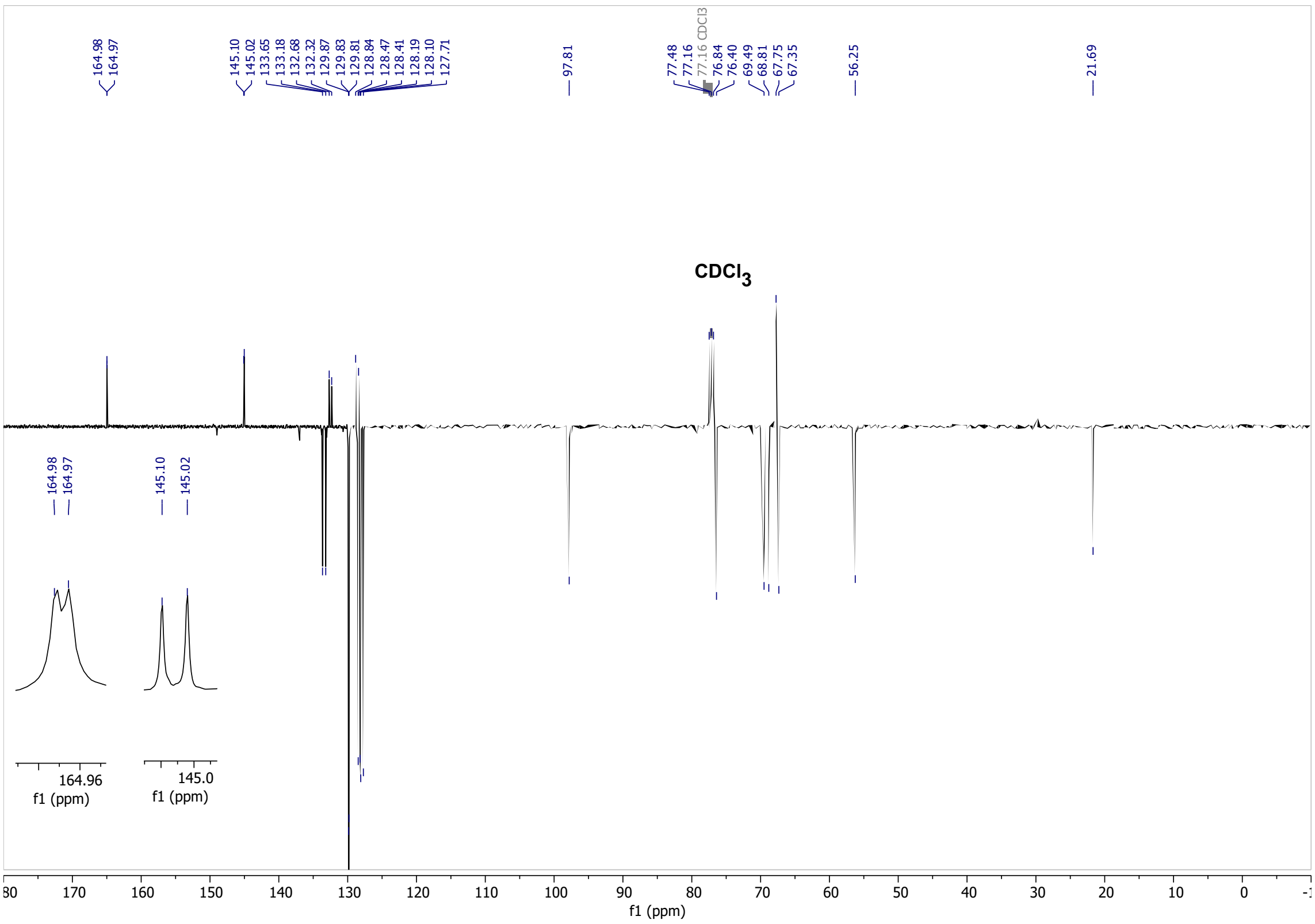

¹H-NMR spectrum of compound 13

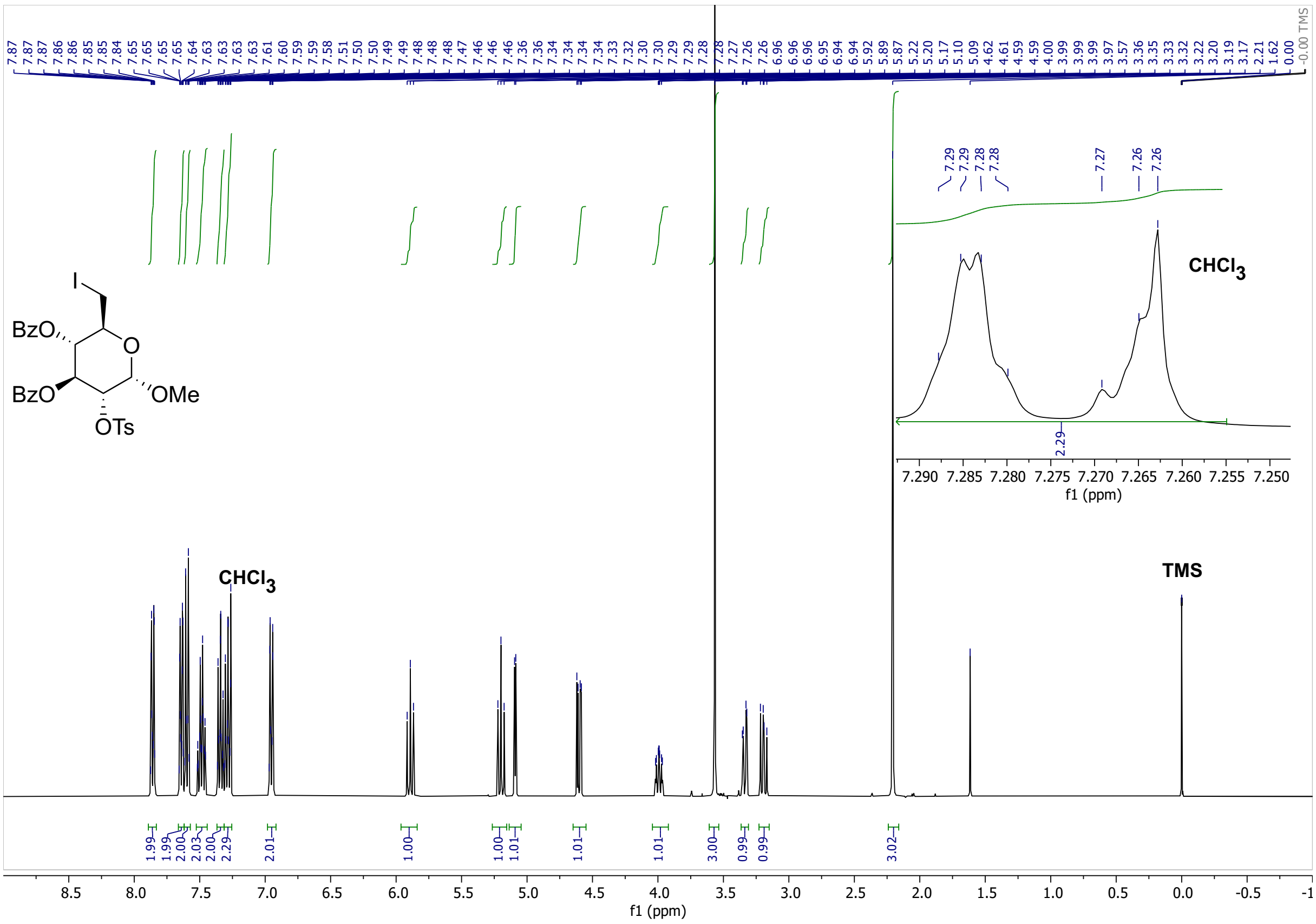

APT-NMR spectrum of compound 13

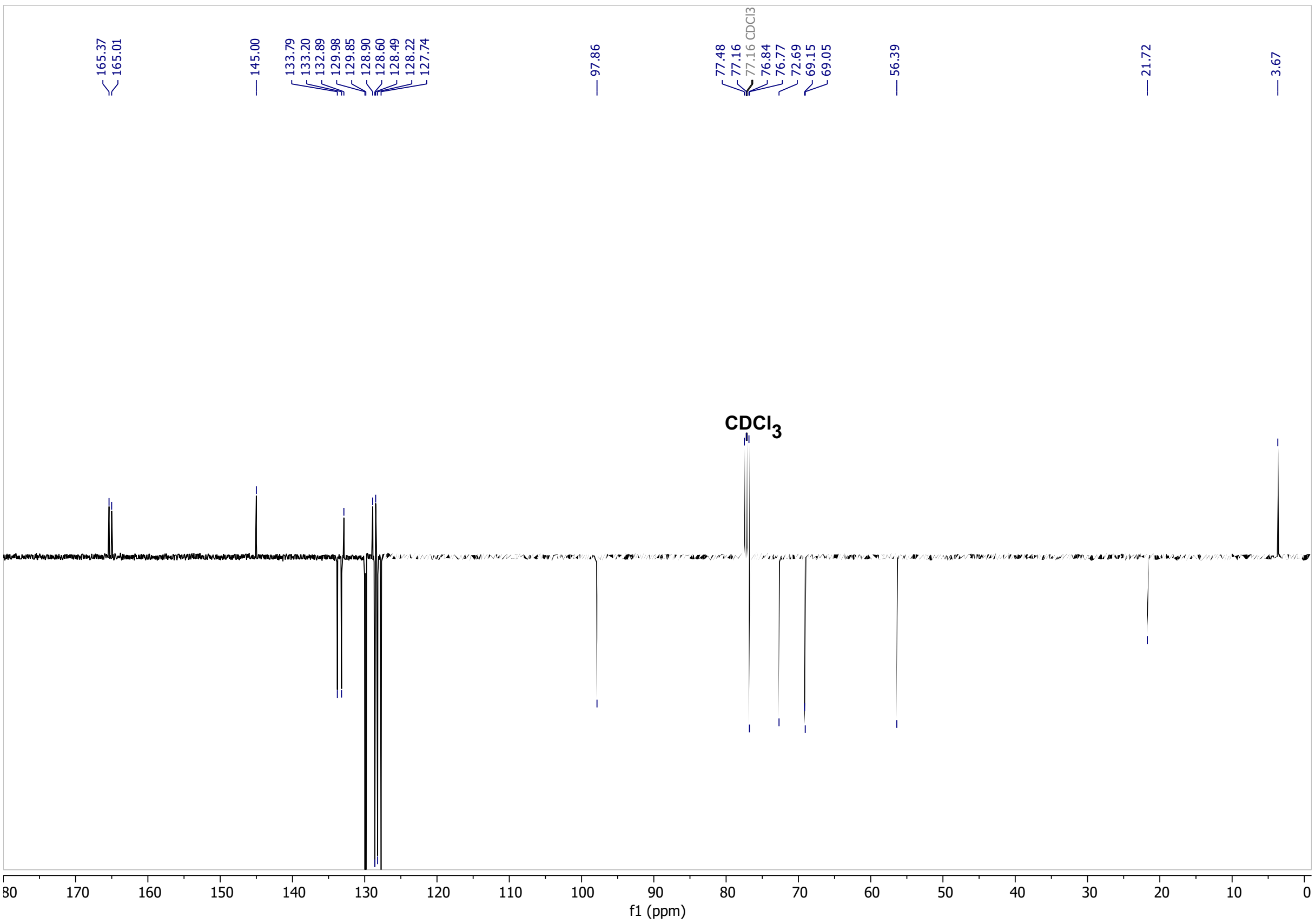

¹H-NMR spectrum of compound 14

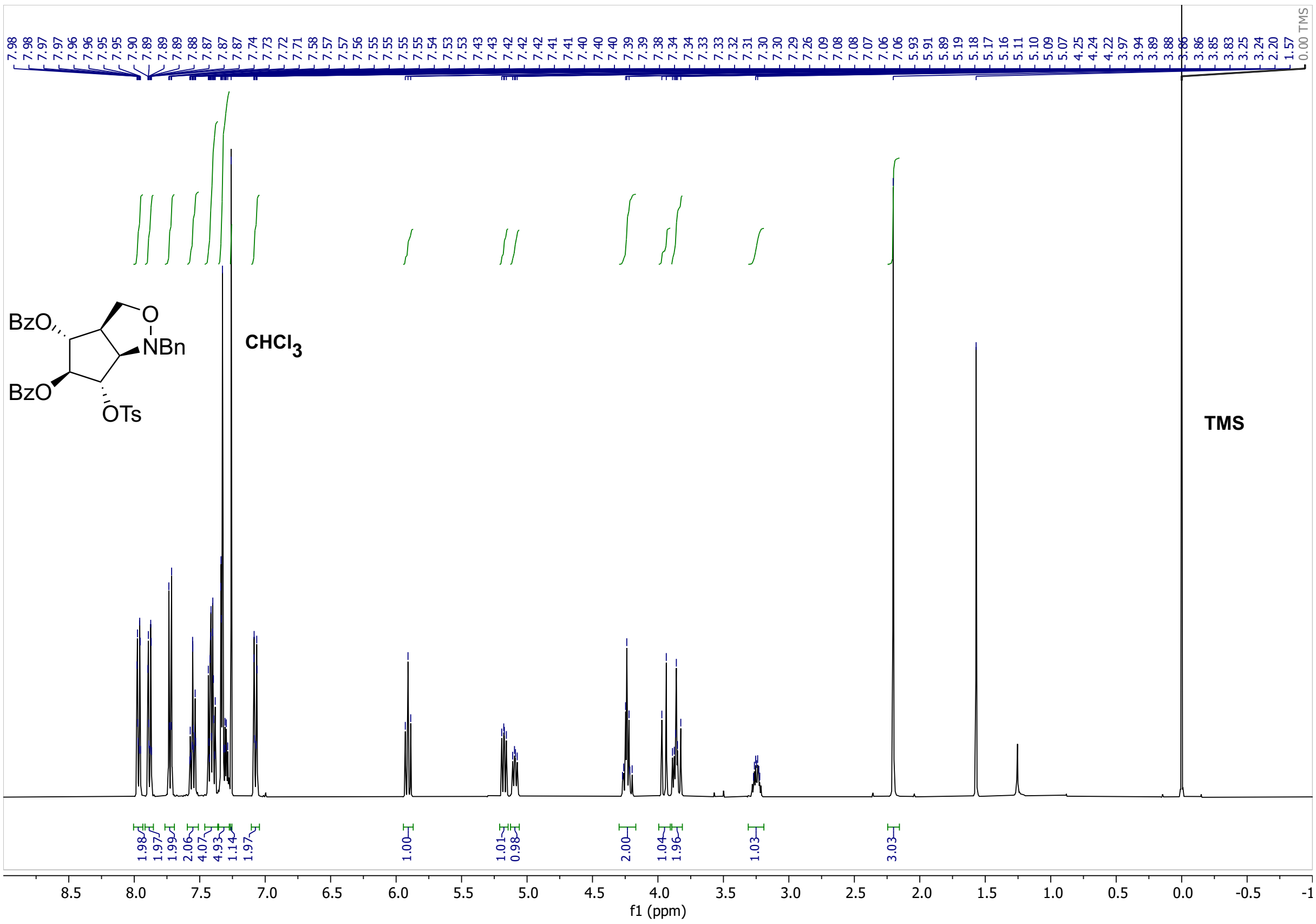

APT-NMR spectrum of compound 14

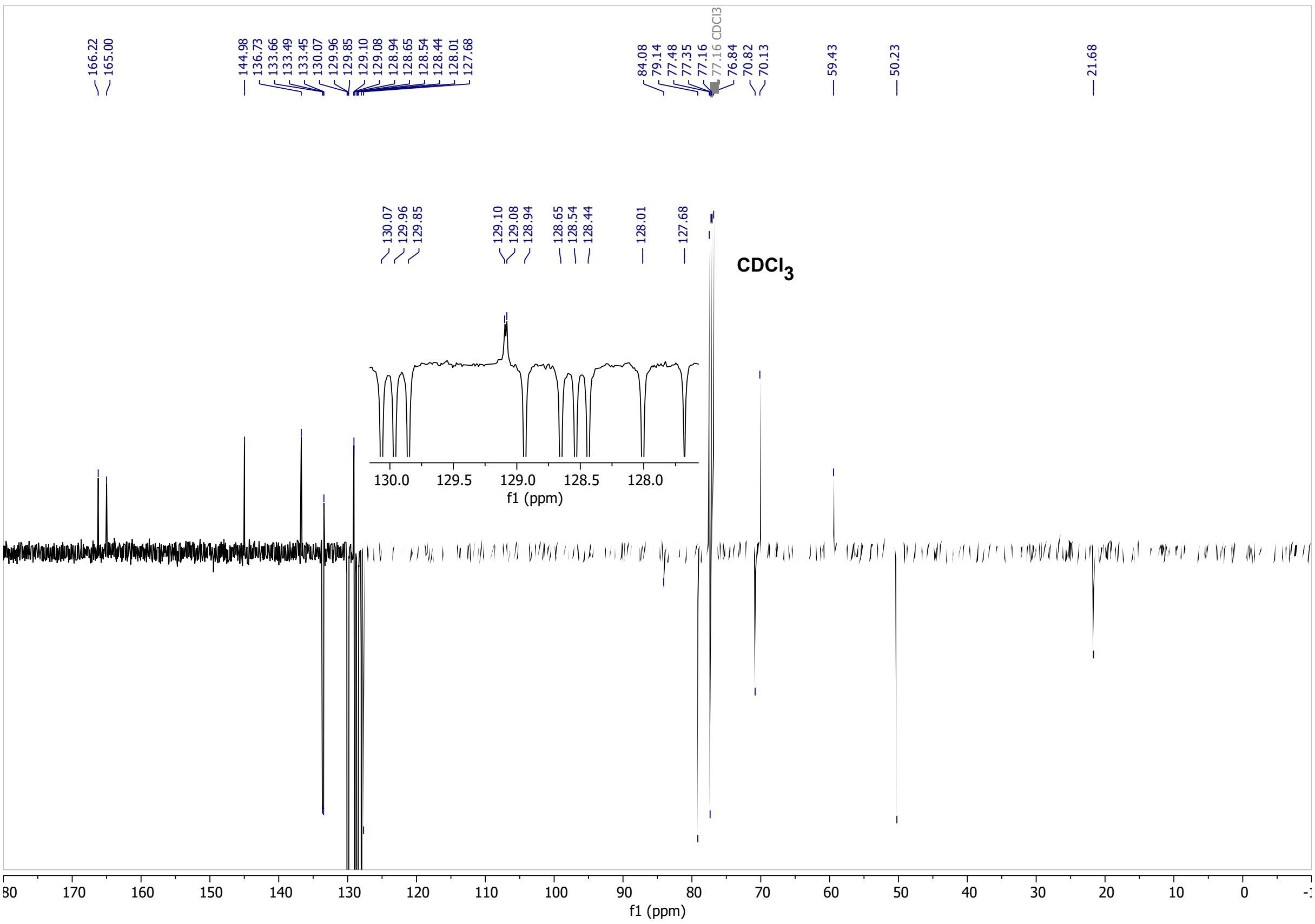

1H-NMR spectrum of compound 15

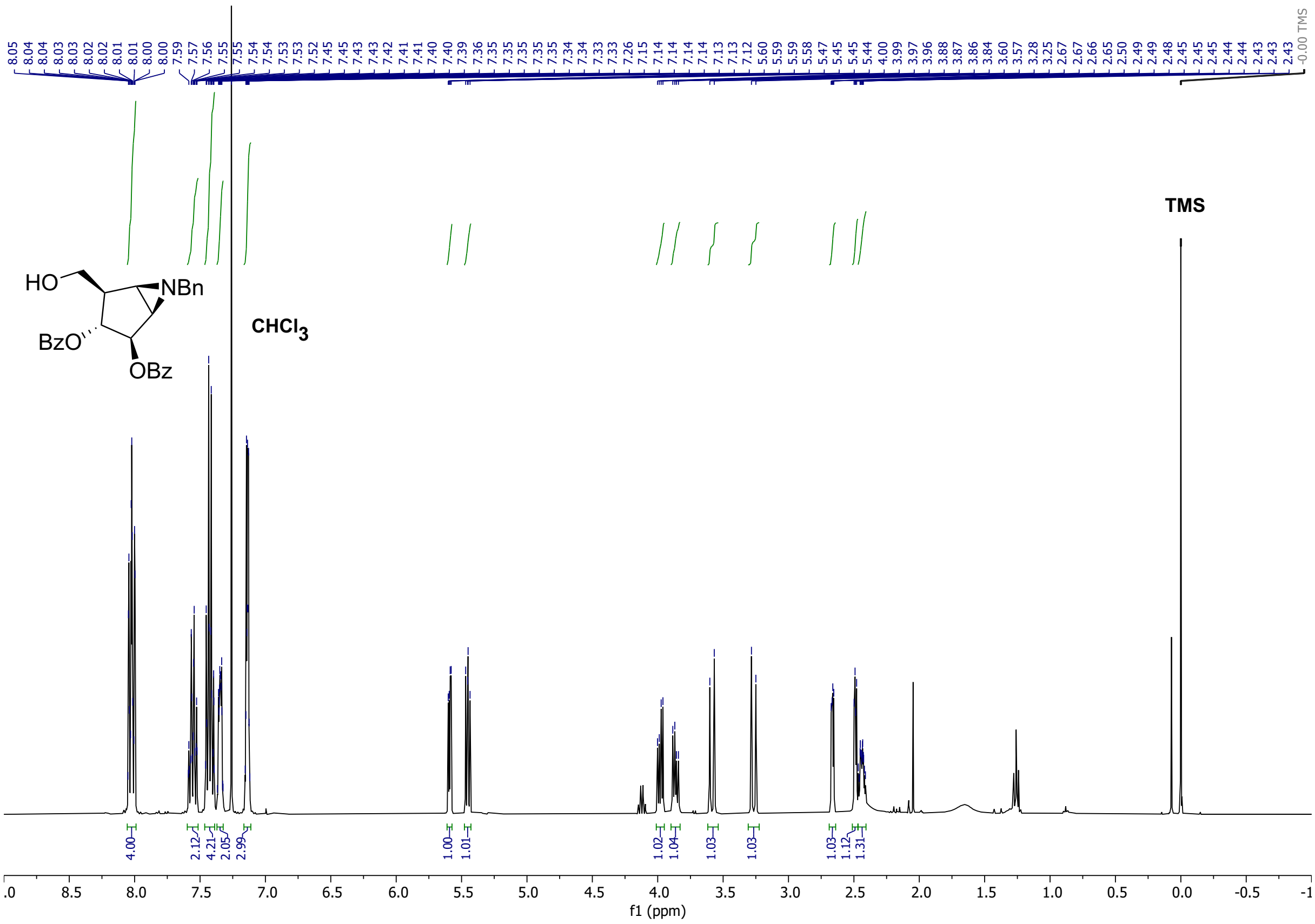

APT-NMR spectrum of compound 15

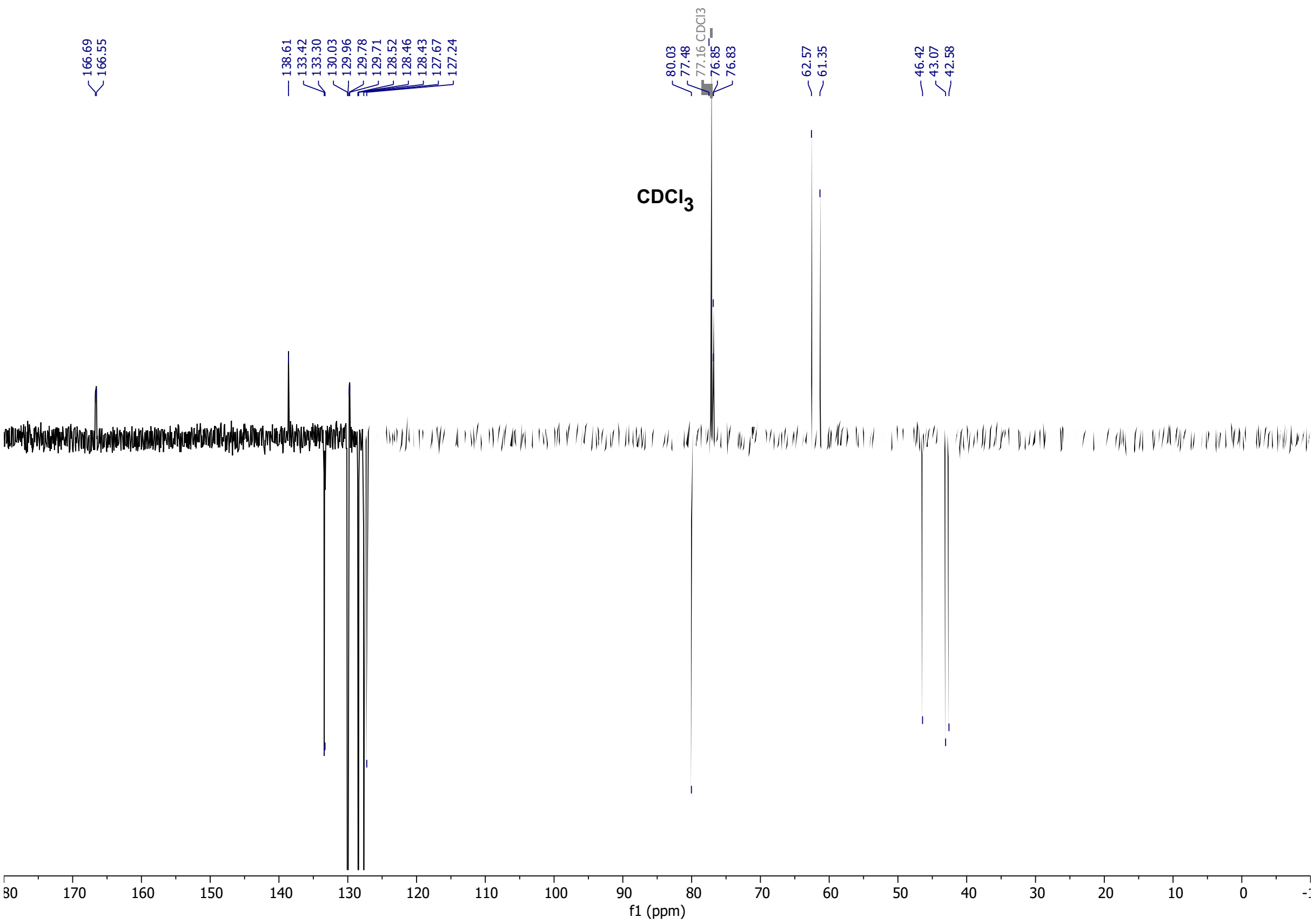

^{1}H-NMR spectrum of compound 15

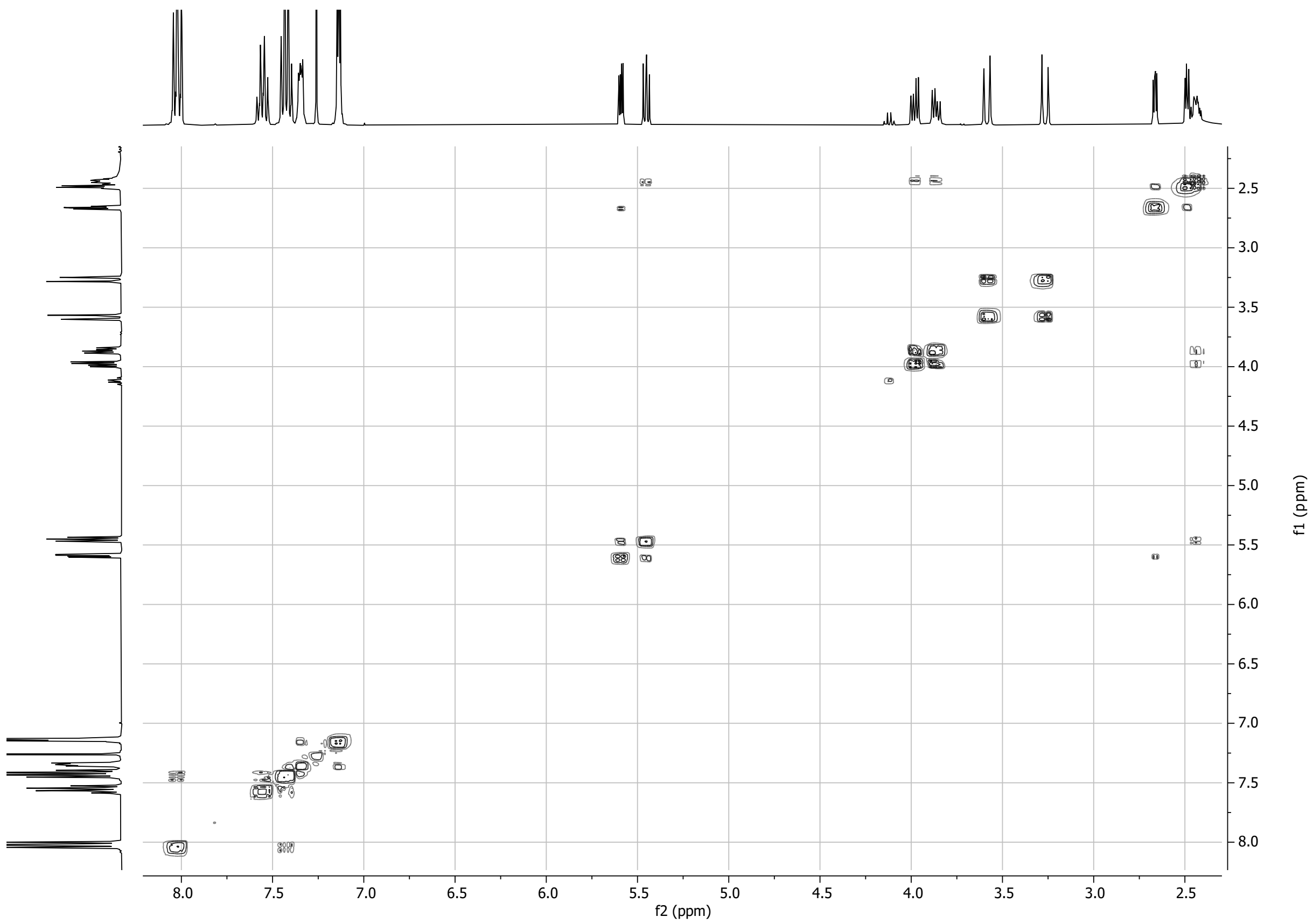

APT-NMR spectrum of compound 15

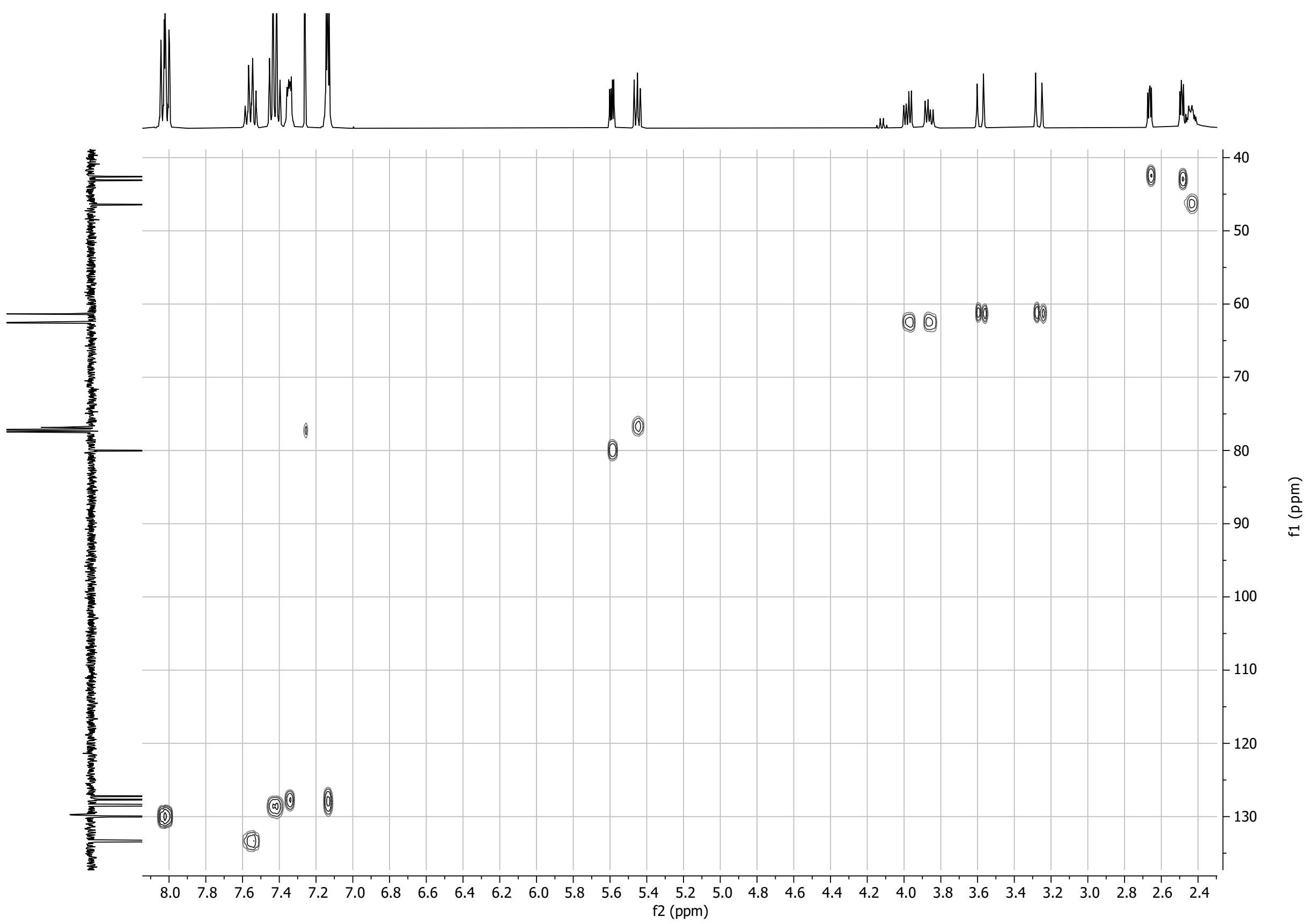

^{1}H-NMR spectrum of compound 16

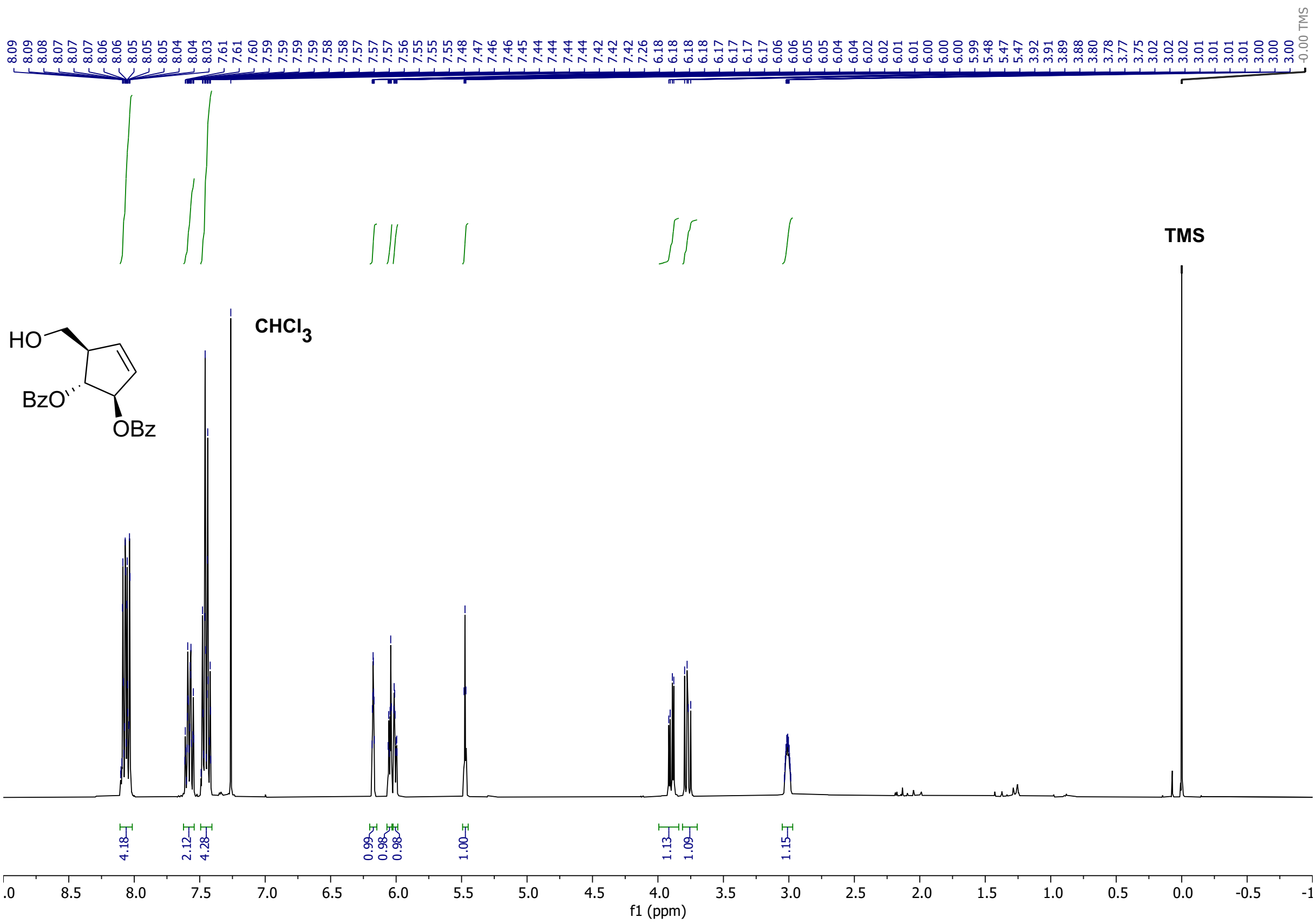

APT-NMR spectrum of compound 16

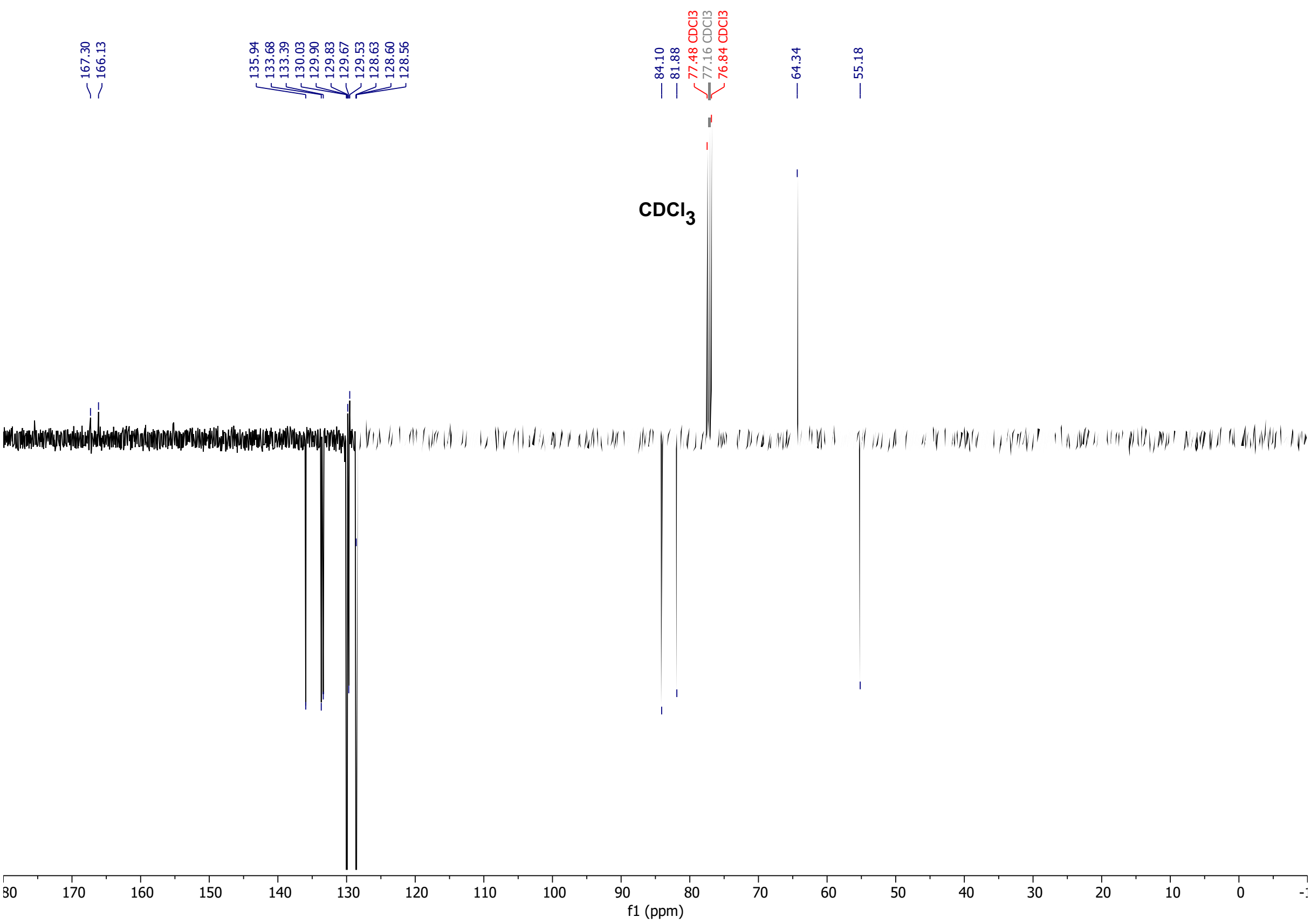

COSY-NMR spectrum of compound 16

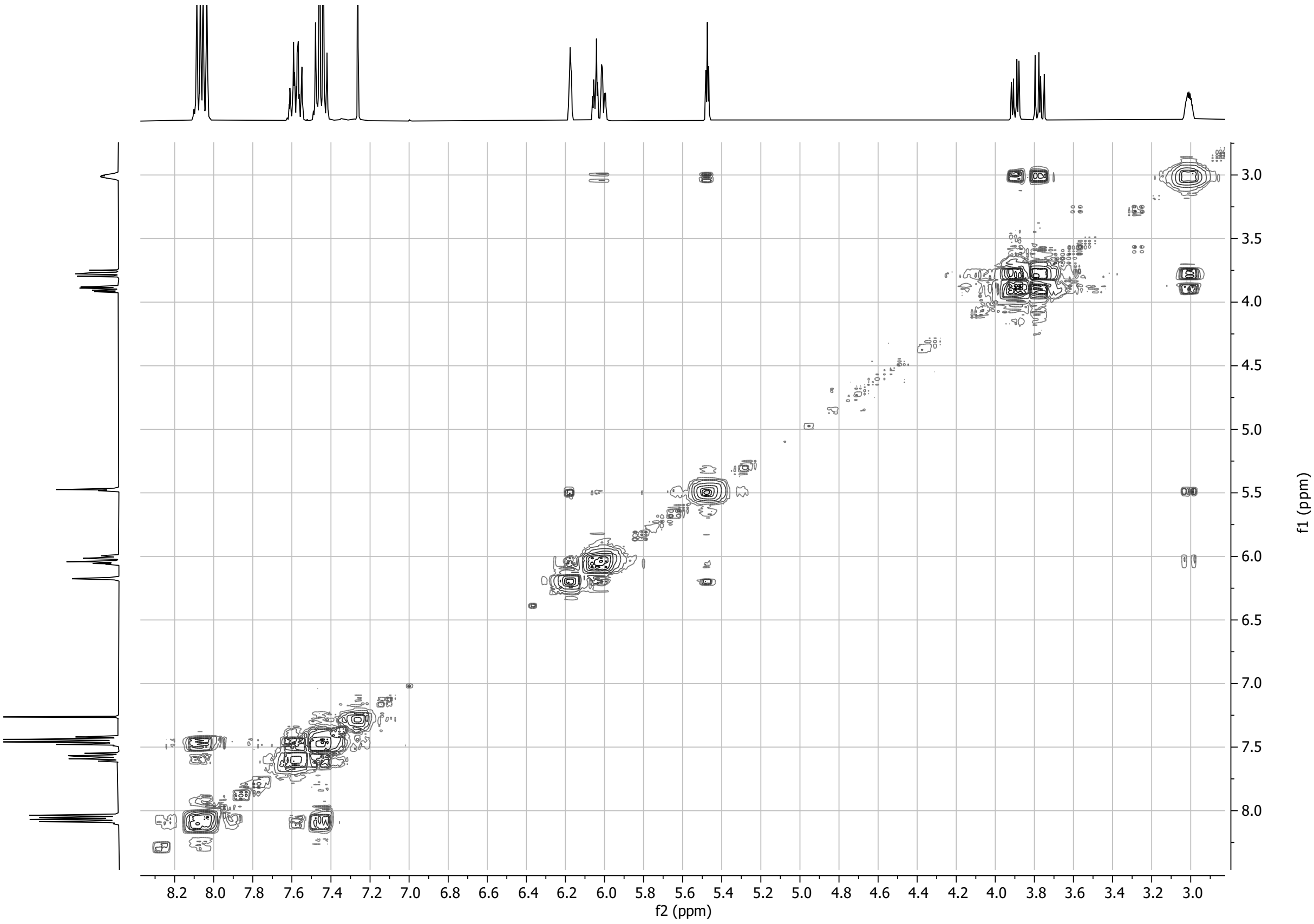

HSQC-NMR spectrum of compound 16

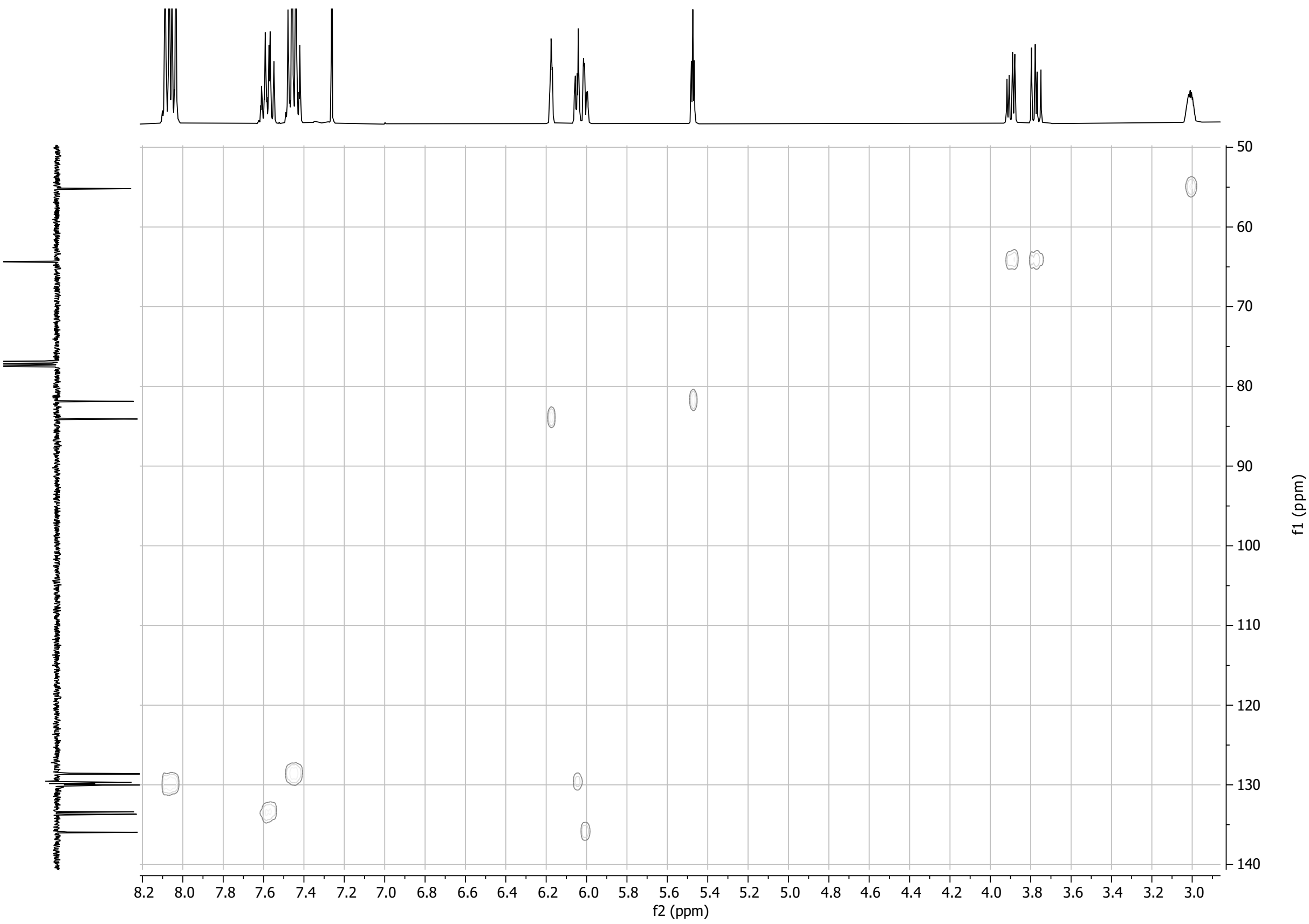

¹H-NMR spectrum of compound 17

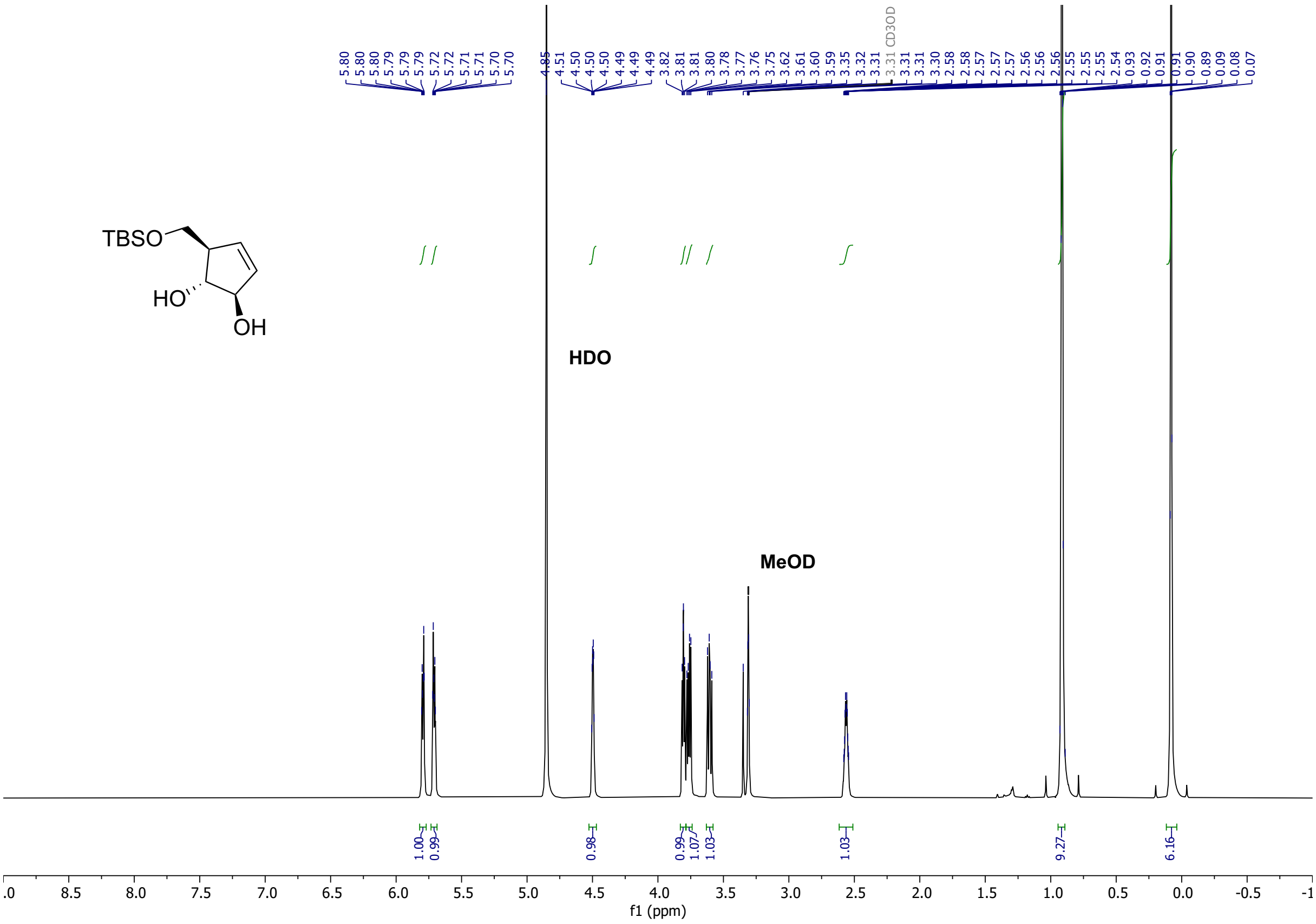

APT-NMR spectrum of compound 17

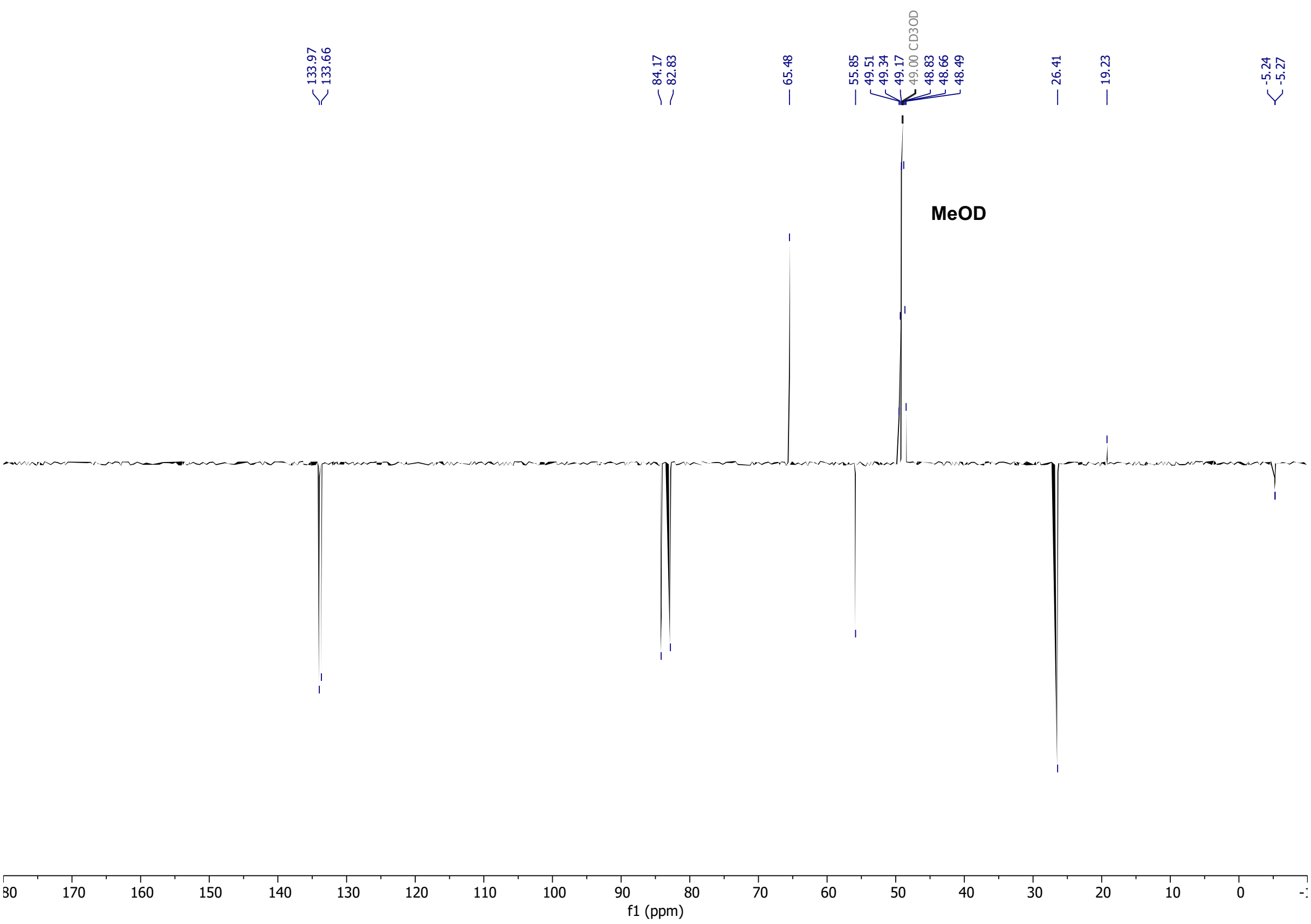

COSY-NMR spectrum of compound 17

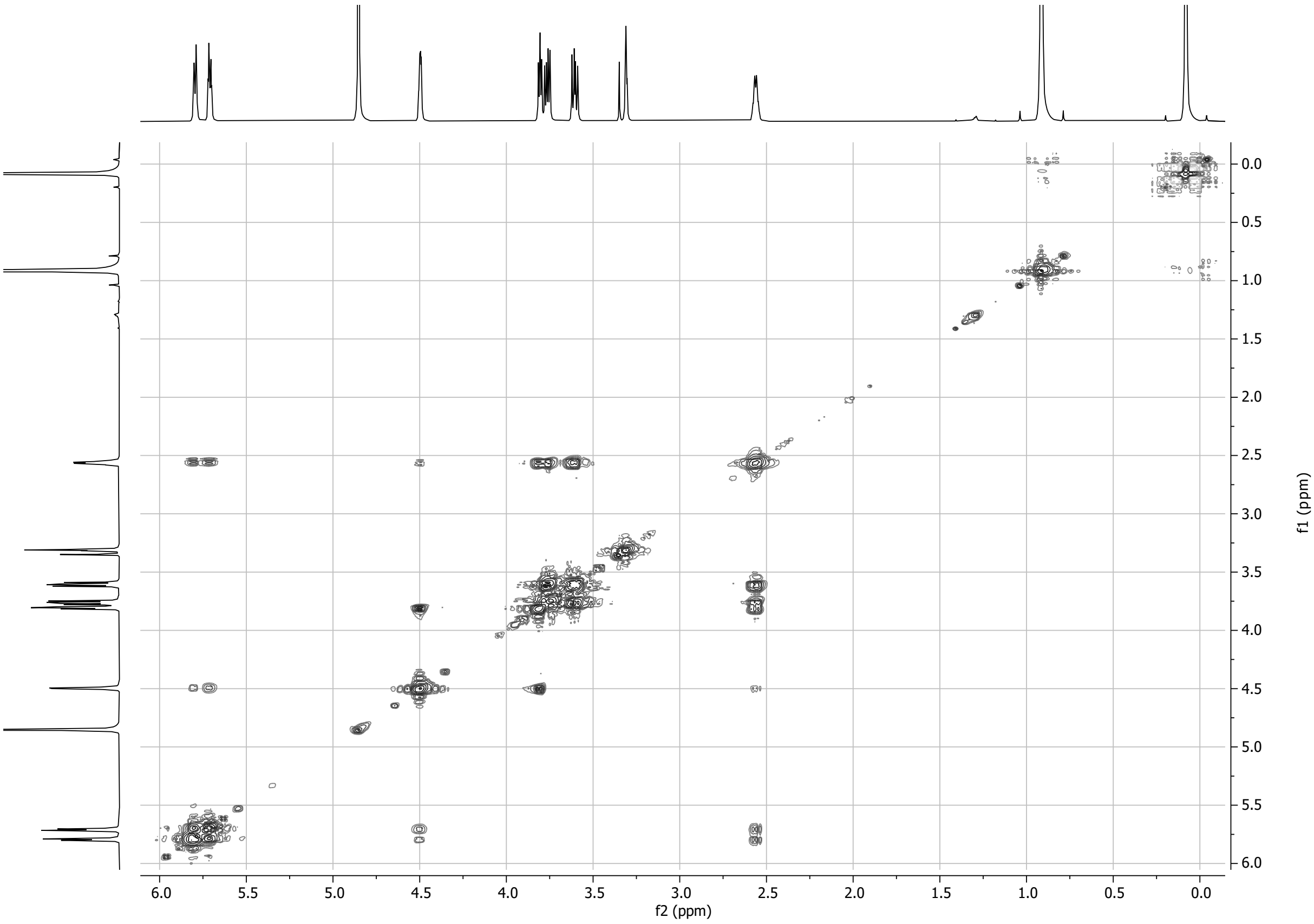

HSQC-NMR spectrum of compound 17

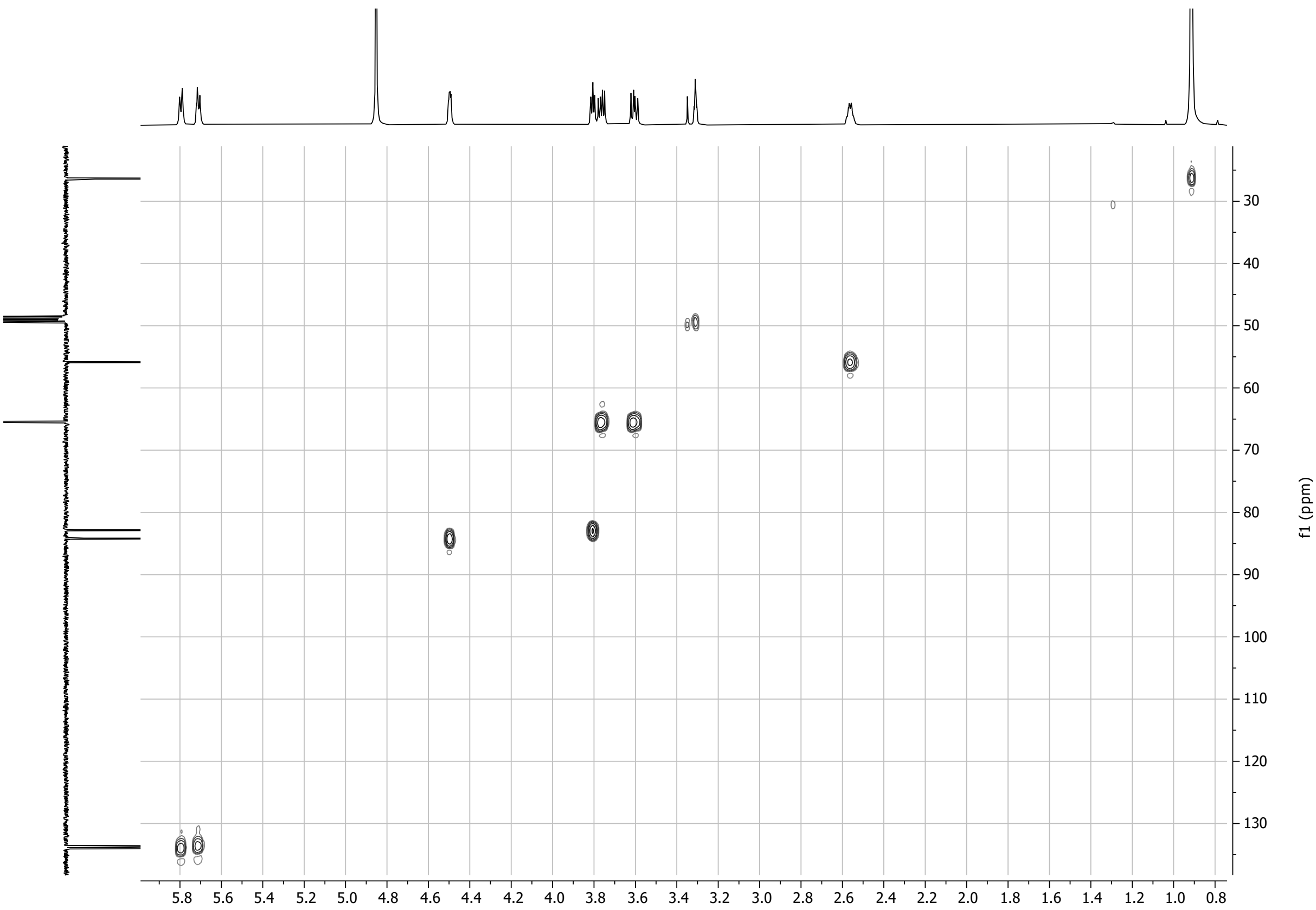

1H-NMR spectrum of compound 18

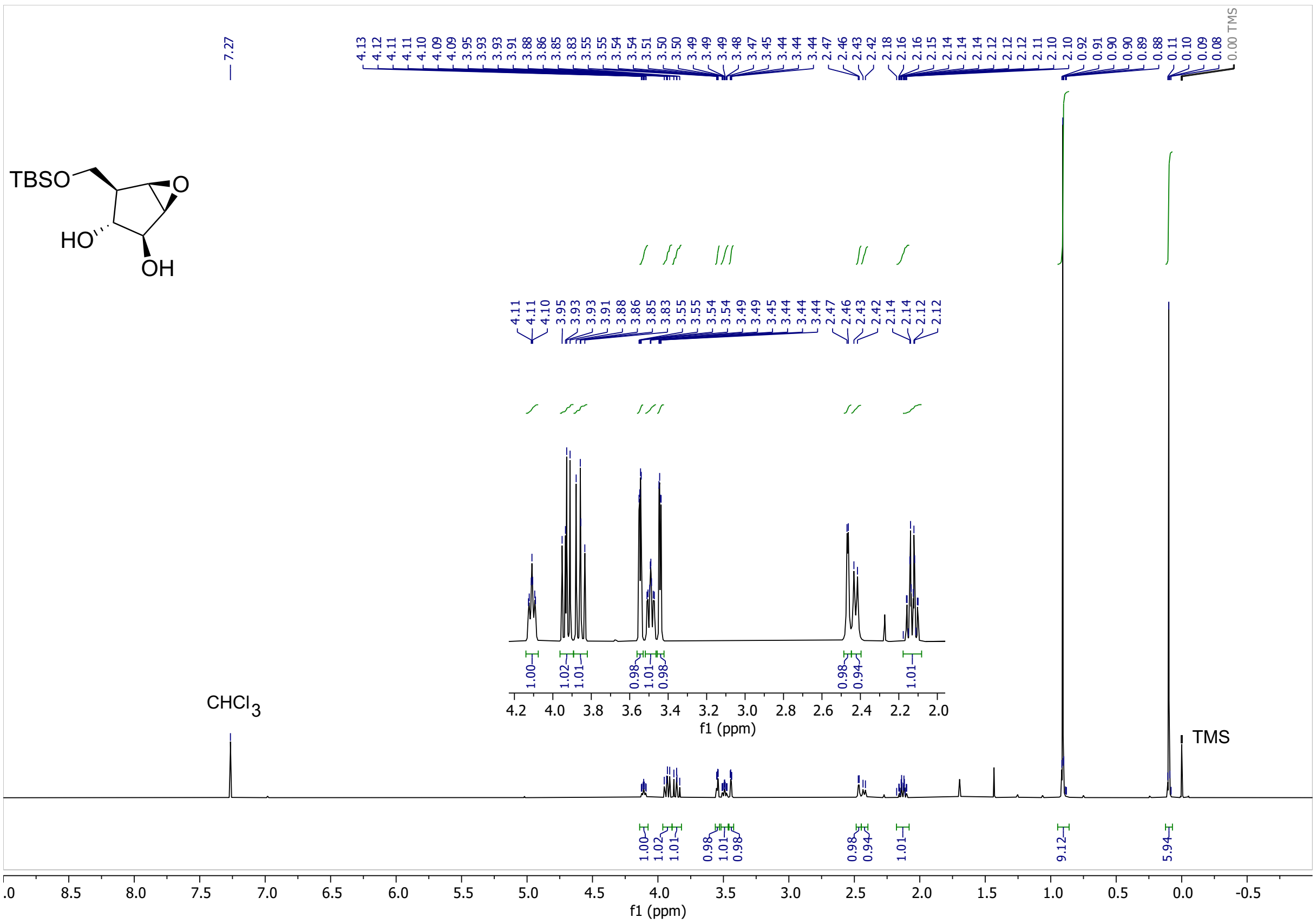

APT-NMR spectrum of compound 18

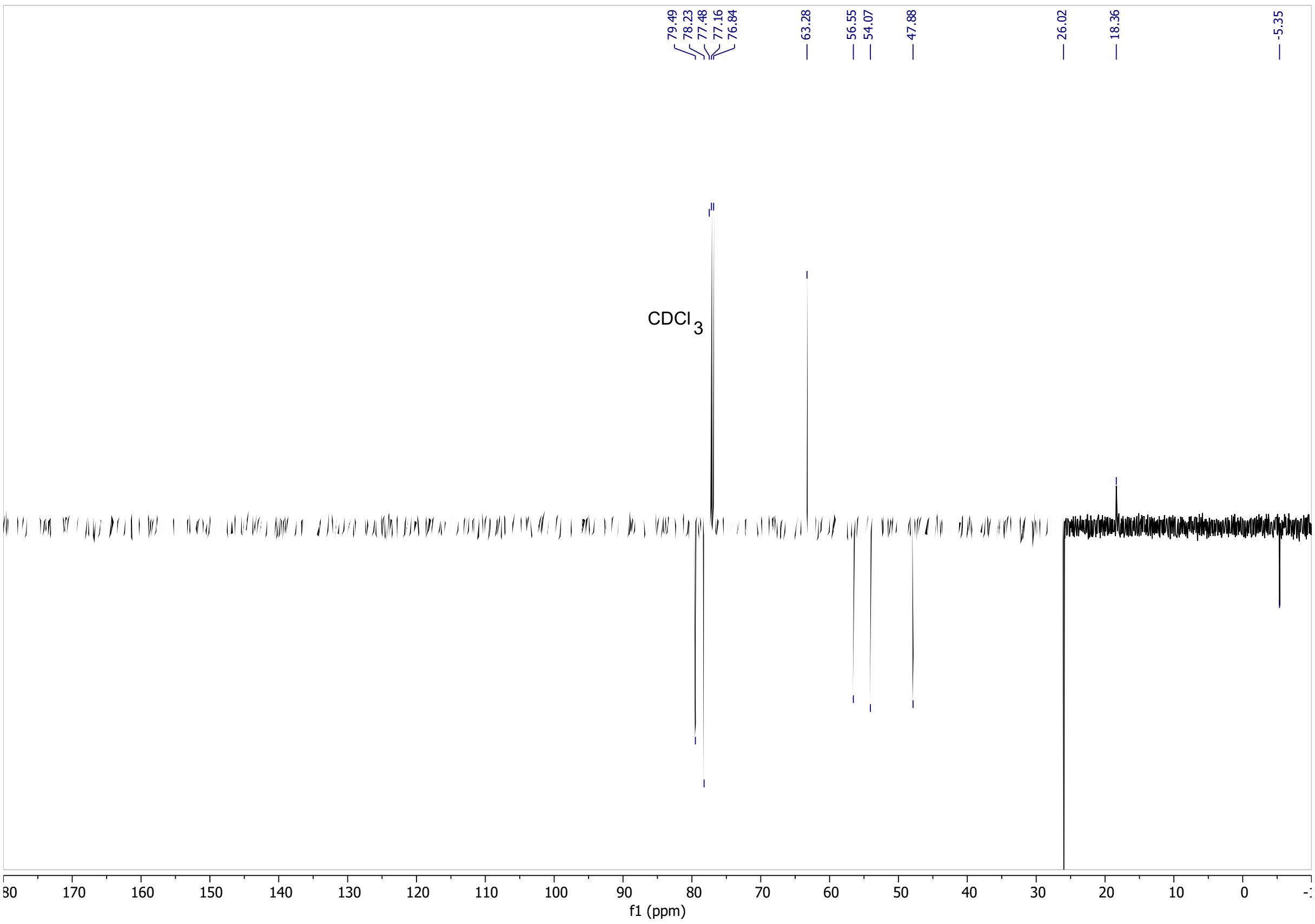

COSY-NMR spectrum of compound 18

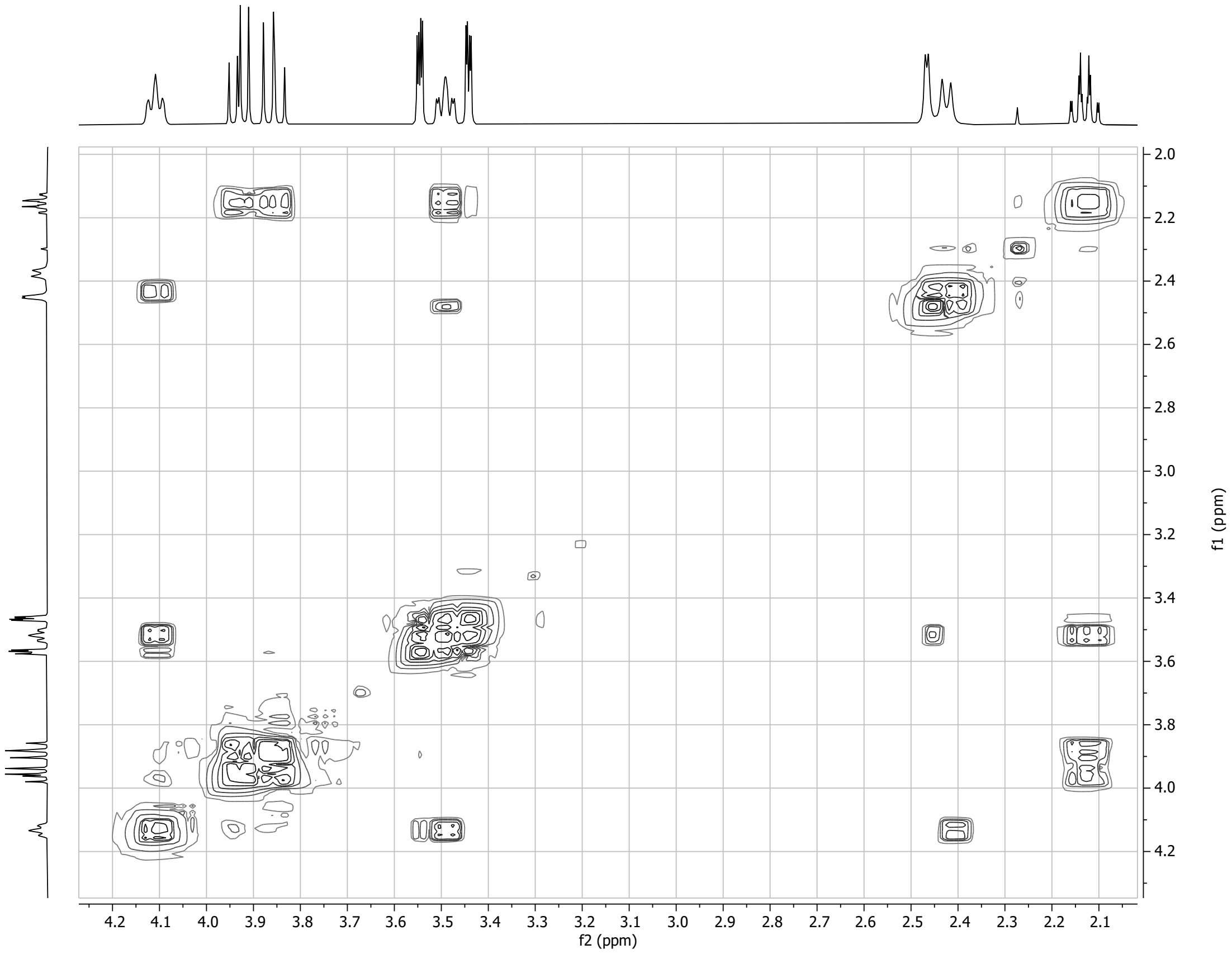

HSQC-NMR spectrum of compound 18

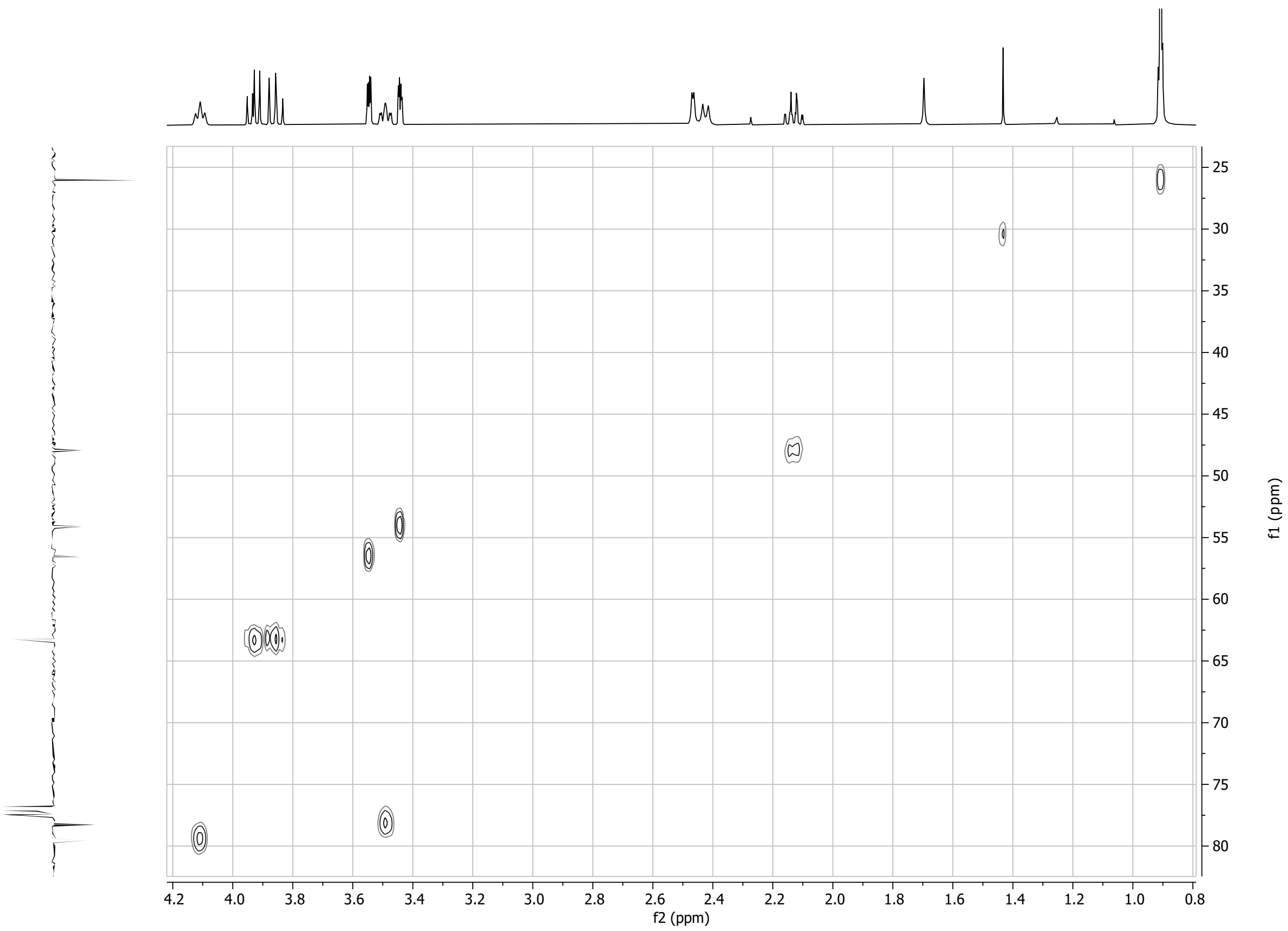

NOESY-NMR spectrum of compound 18

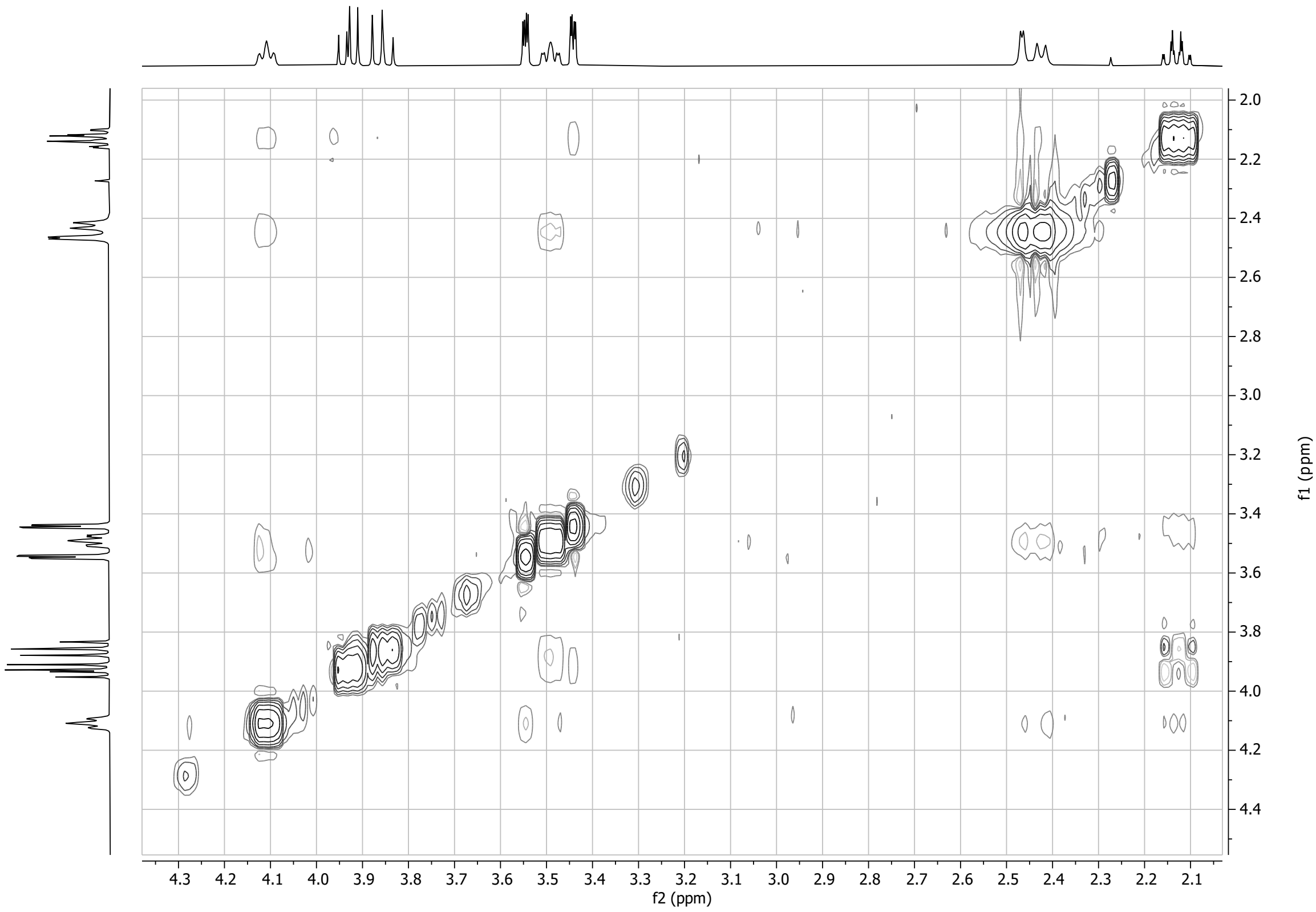

^{1}H-NMR spectrum of compound 1

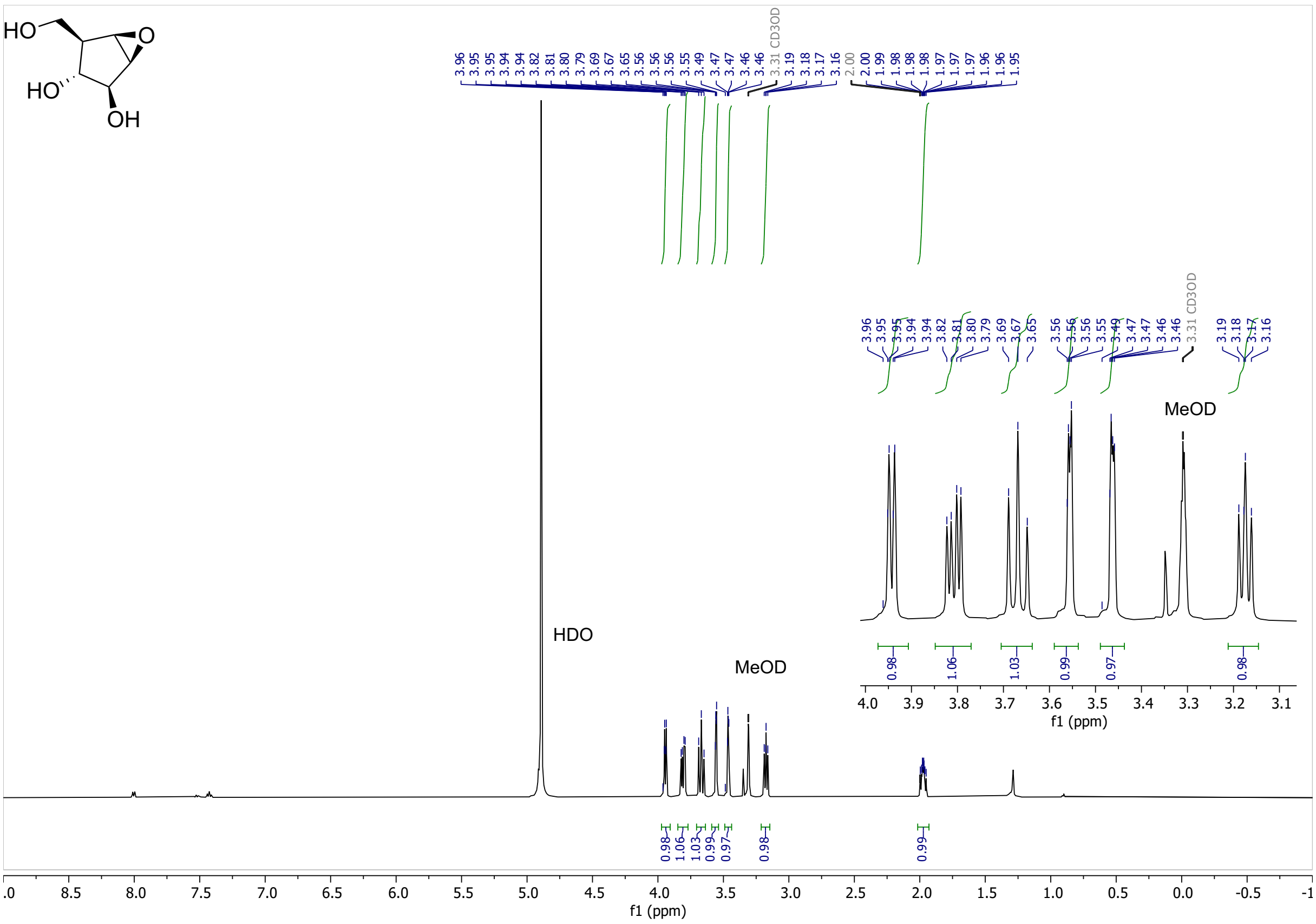

APT-NMR spectrum of compound 1

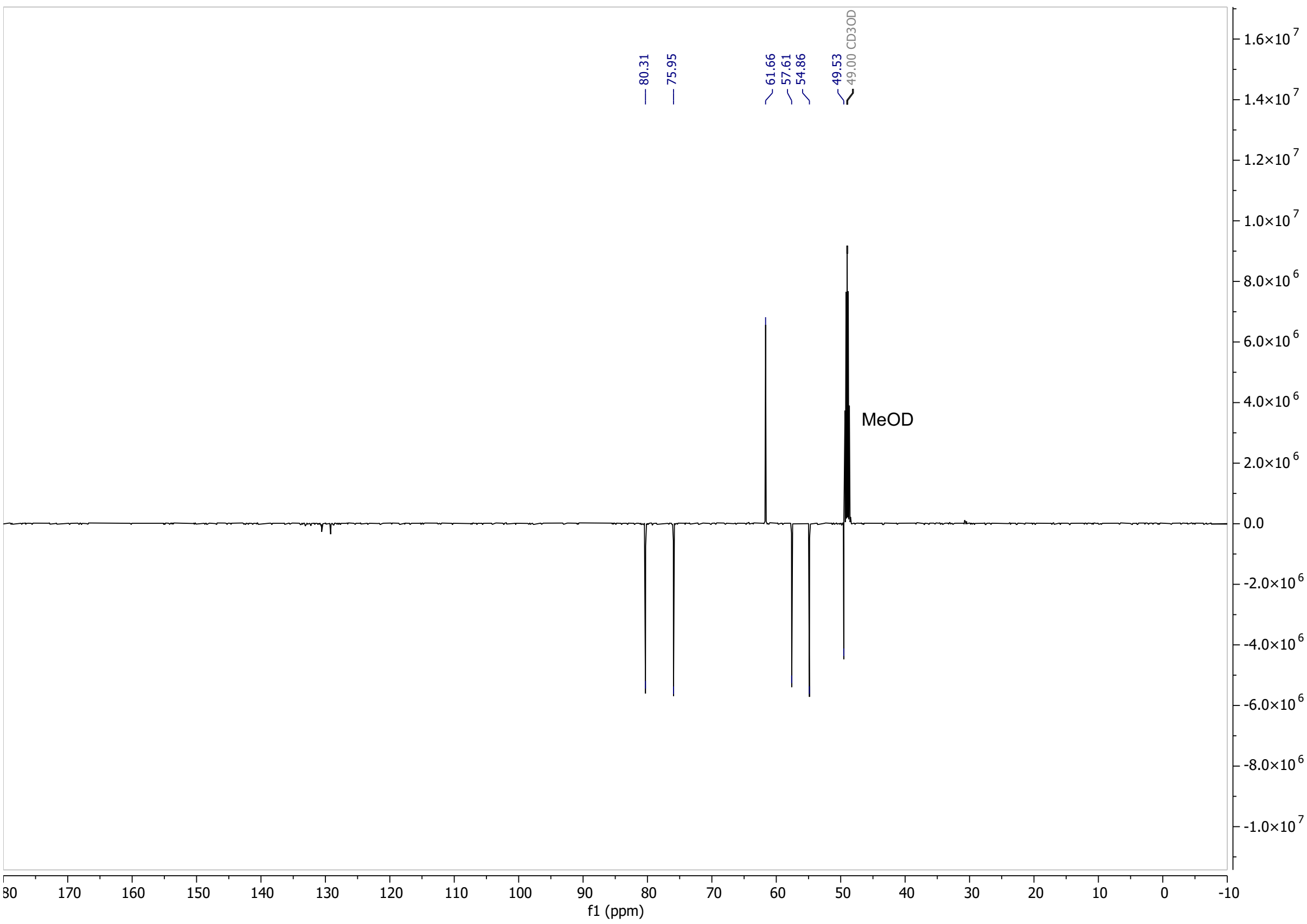

COSY-NMR spectrum of compound 1

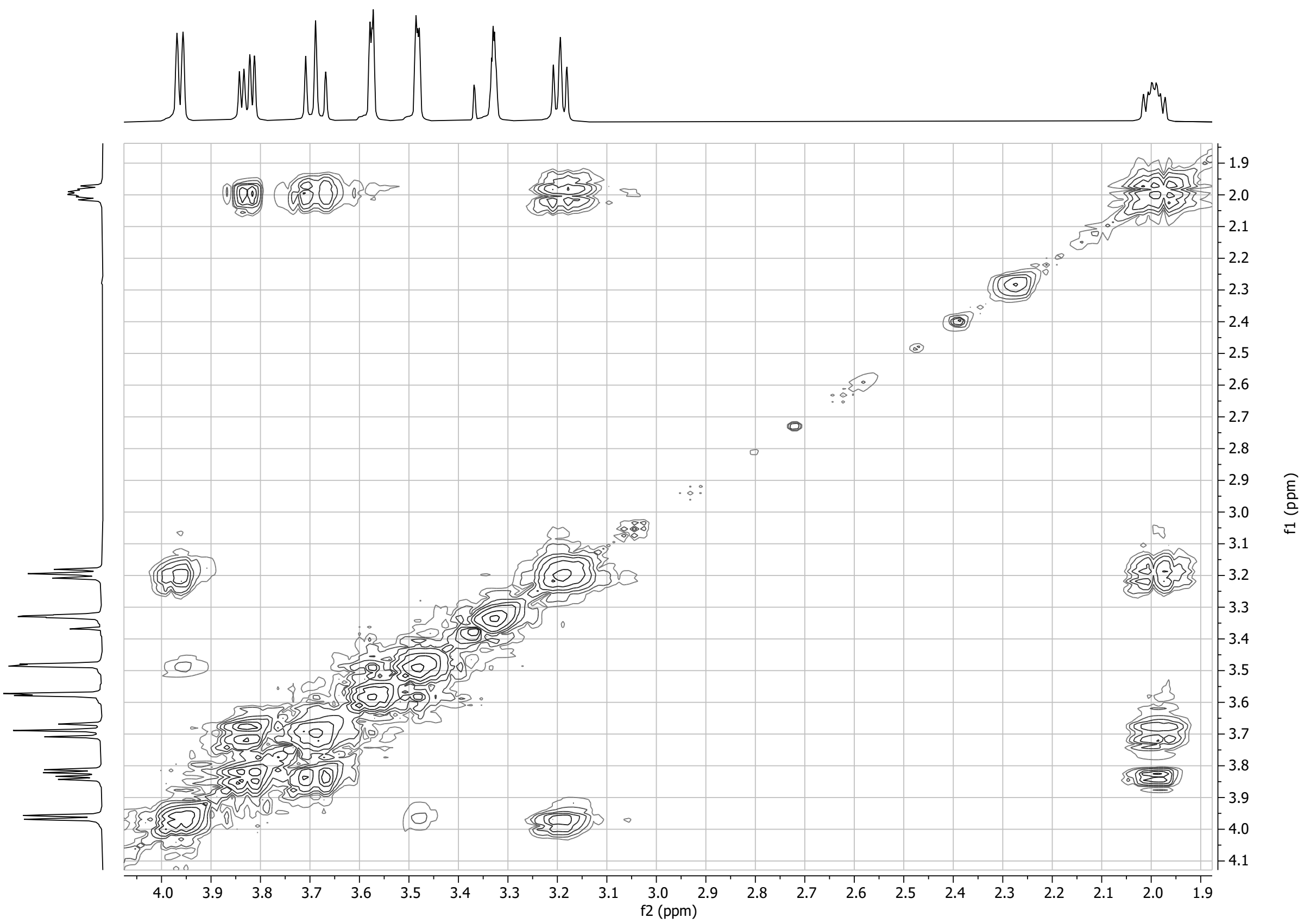

HSQC-NMR spectrum of compound 1

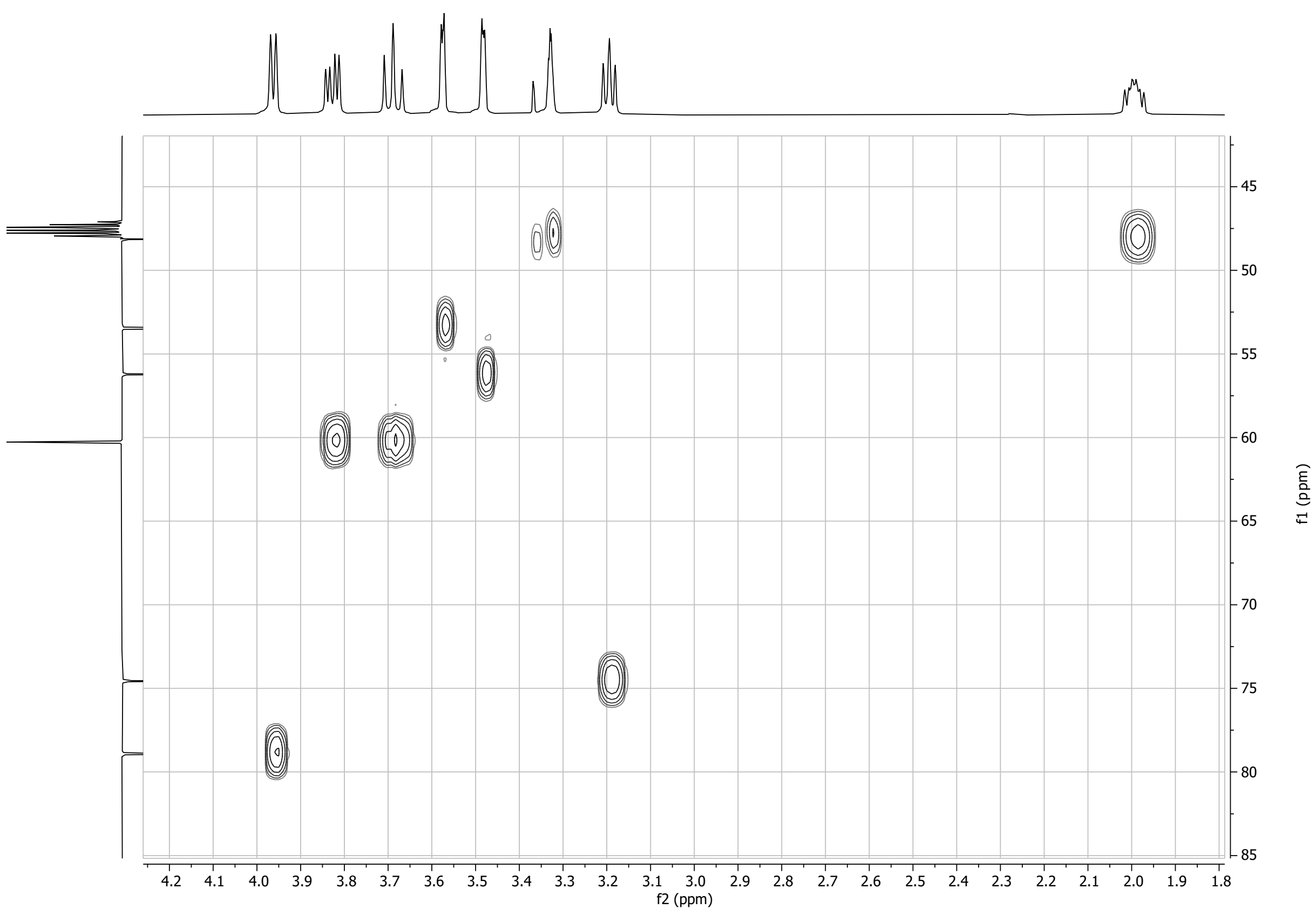

^{1}H-NMR spectrum of compound 19

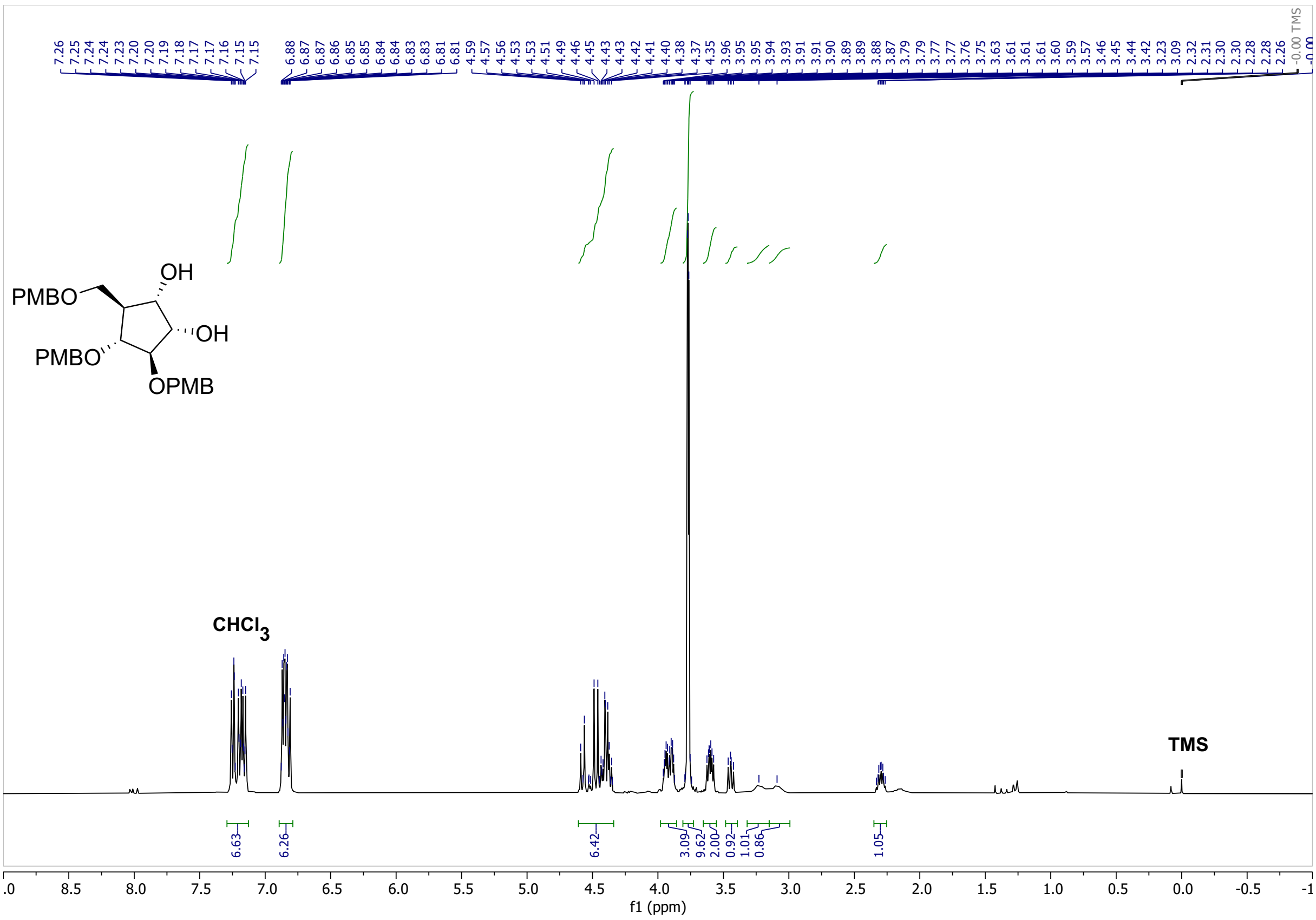

APT-NMR spectrum of compound 19

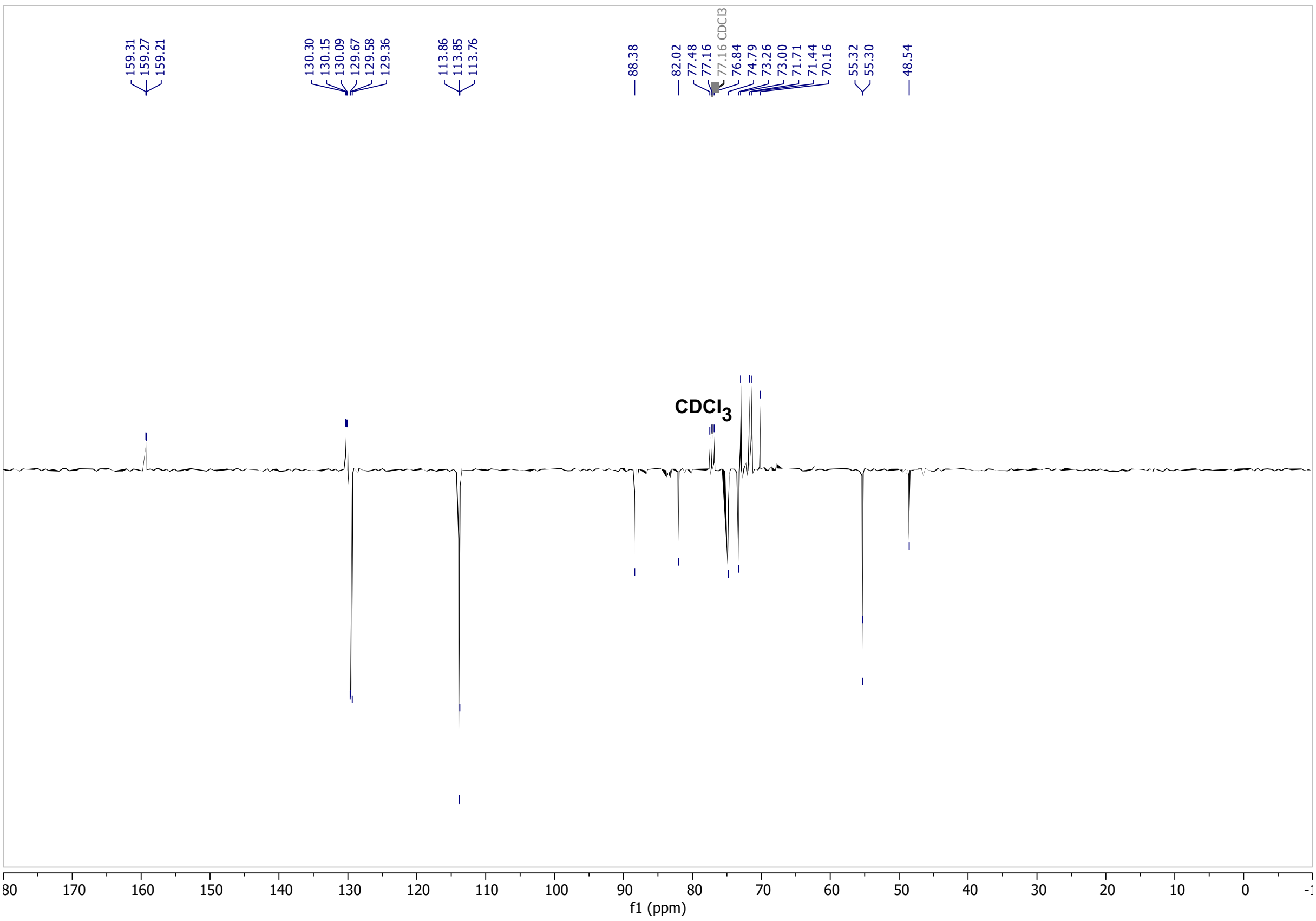

COSY-NMR spectrum of compound 19

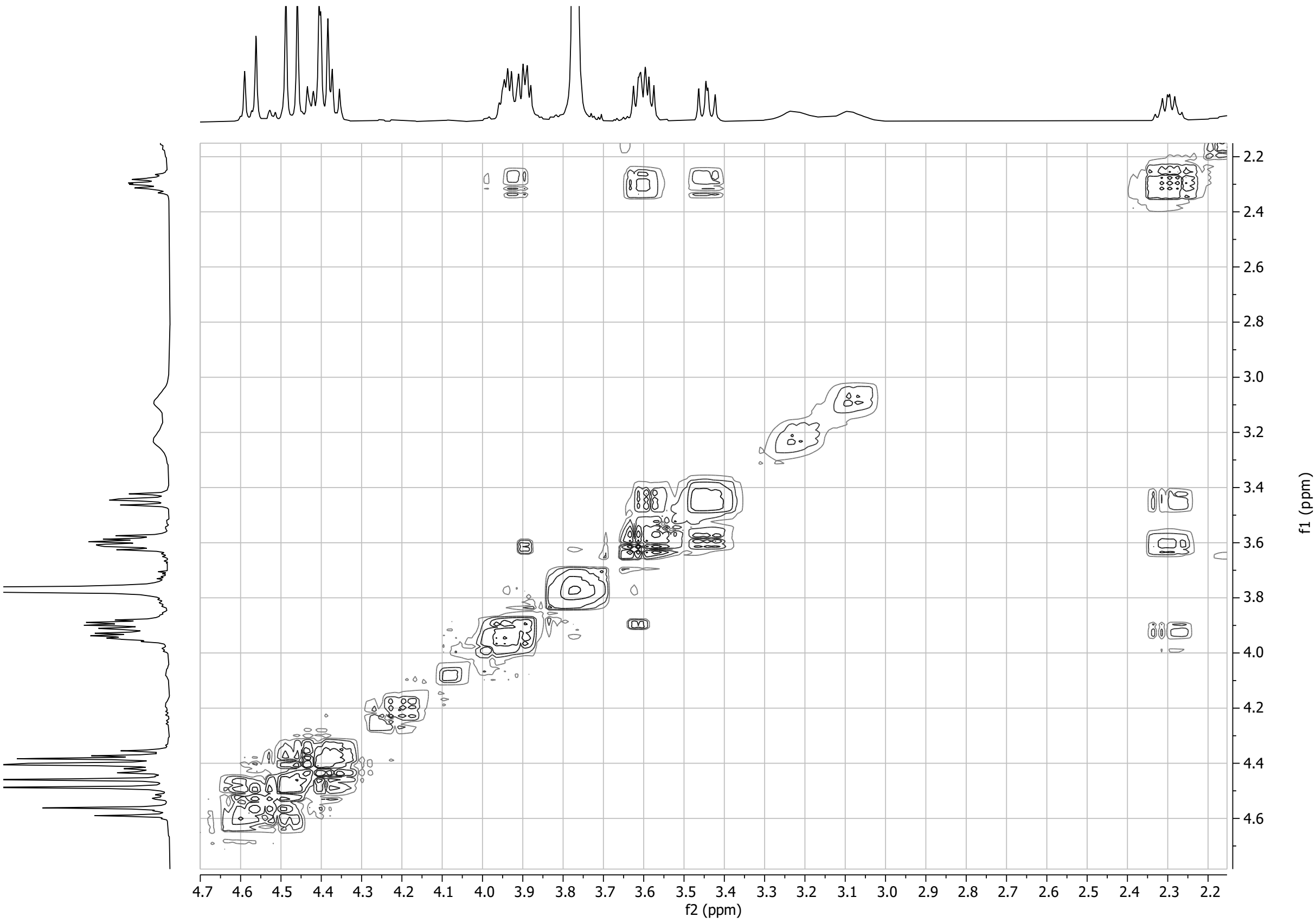

HSQC-NMR spectrum of compound 19

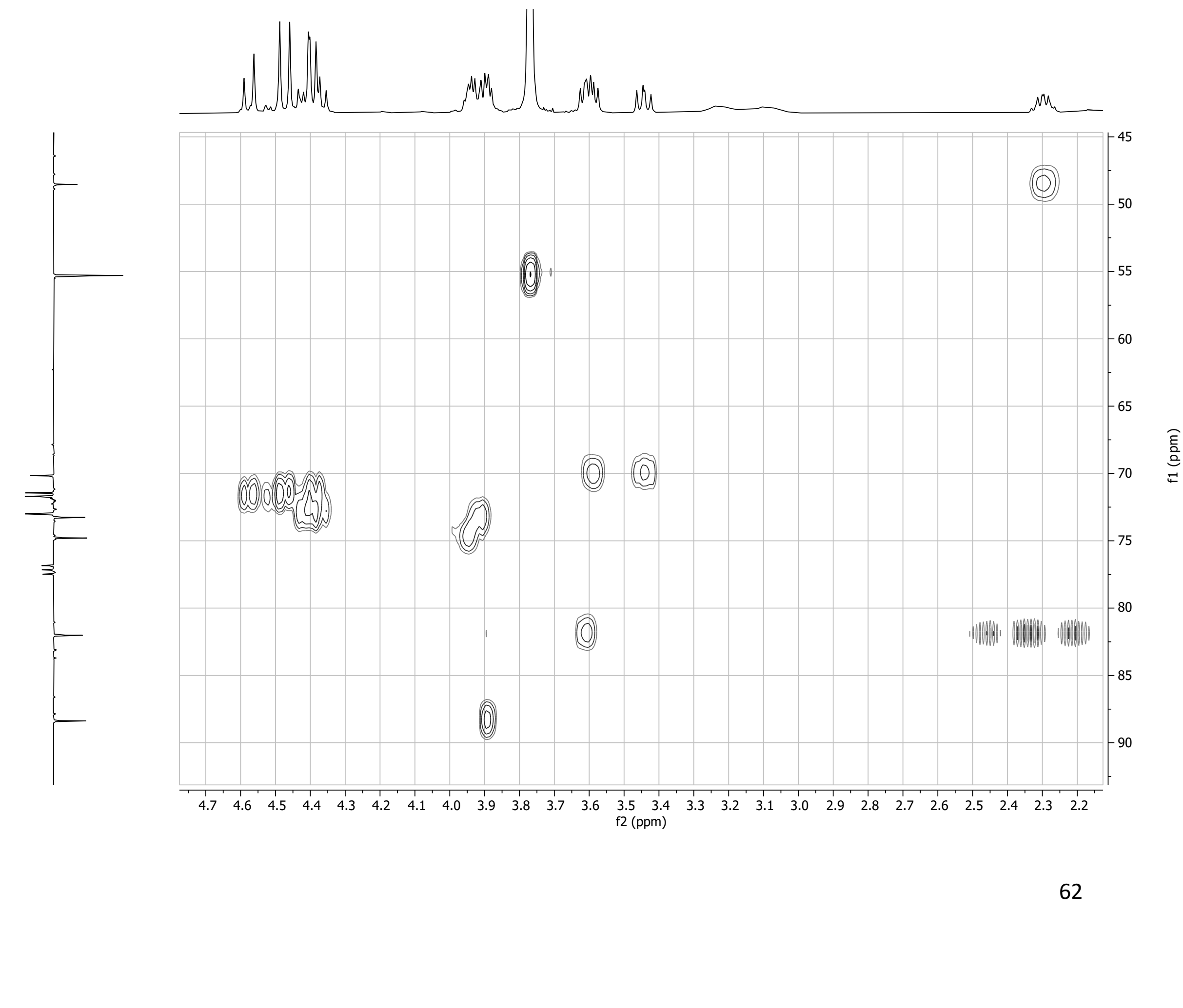

¹H-NMR spectrum of compound 20

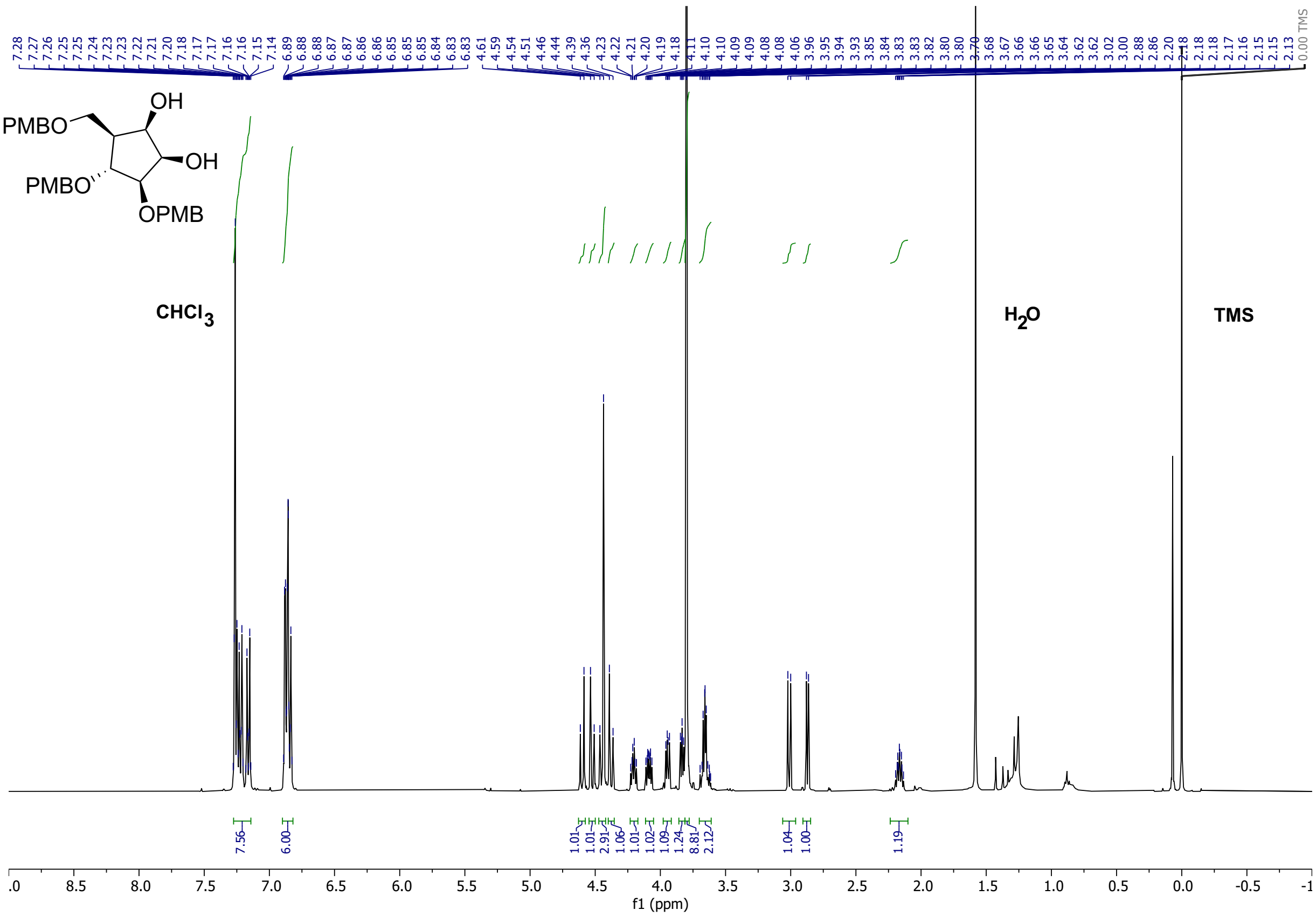

APT-NMR spectrum of compound 20

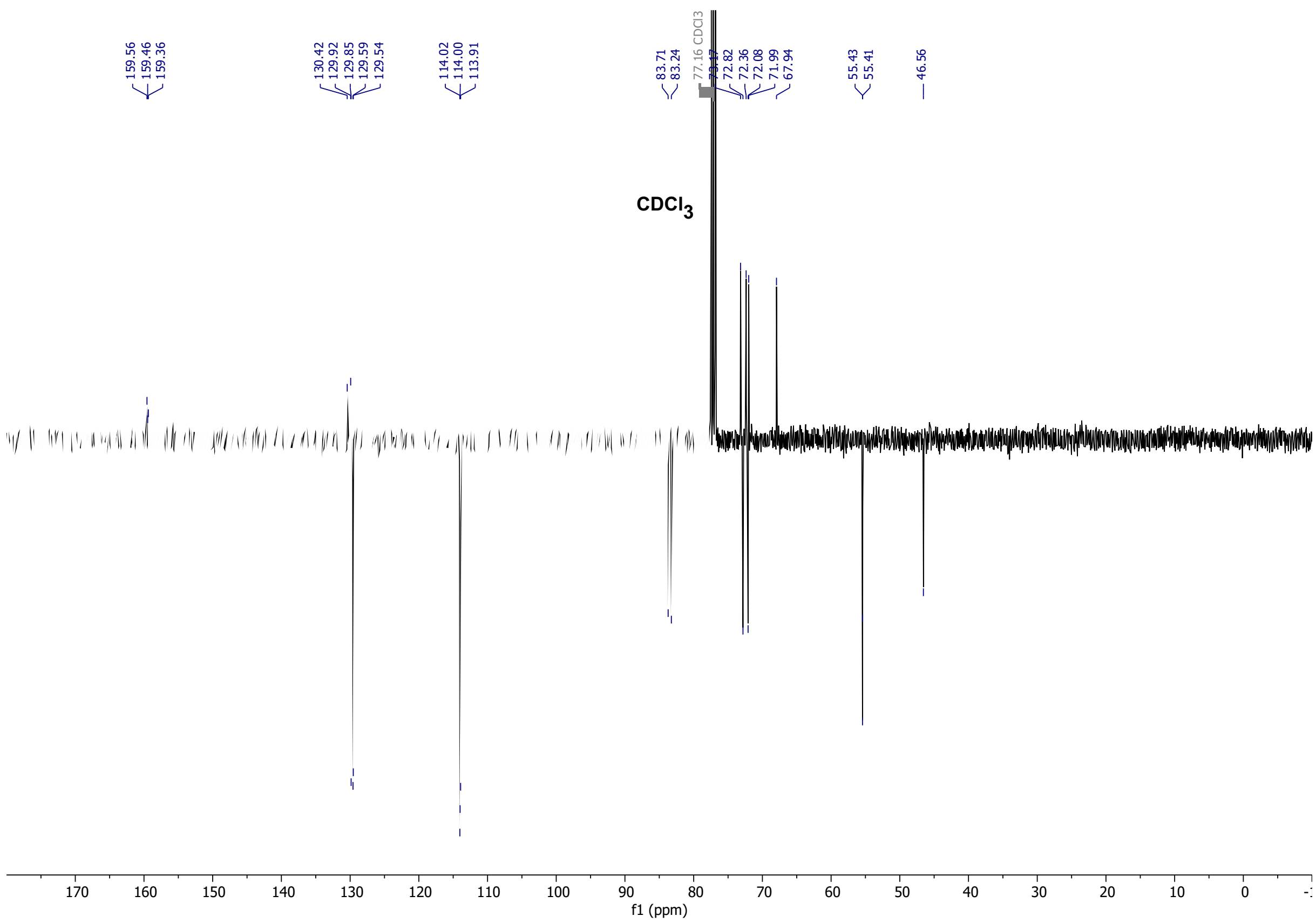

COSY-NMR spectrum of compound 20

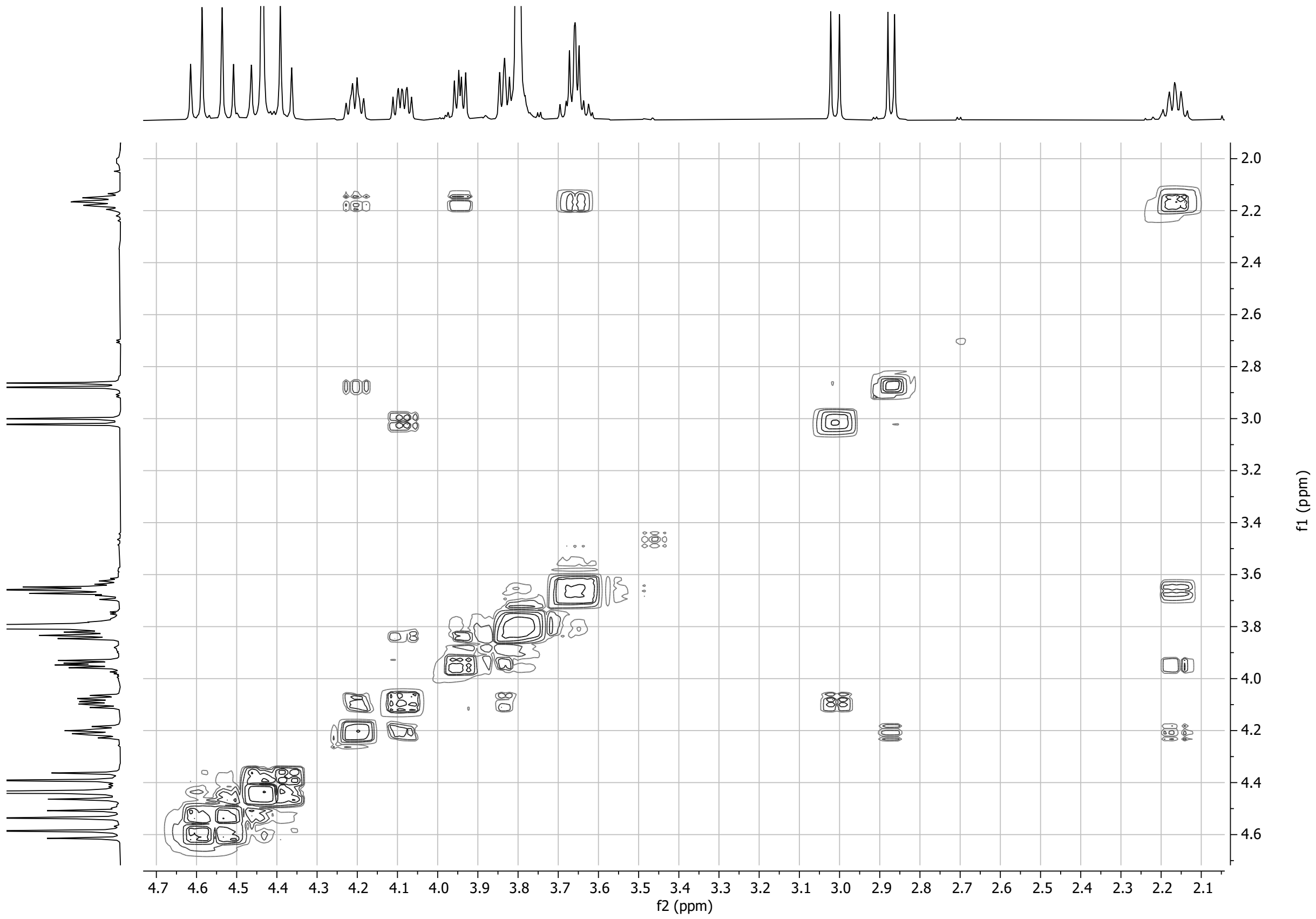

HSQC-NMR spectrum of compound 20

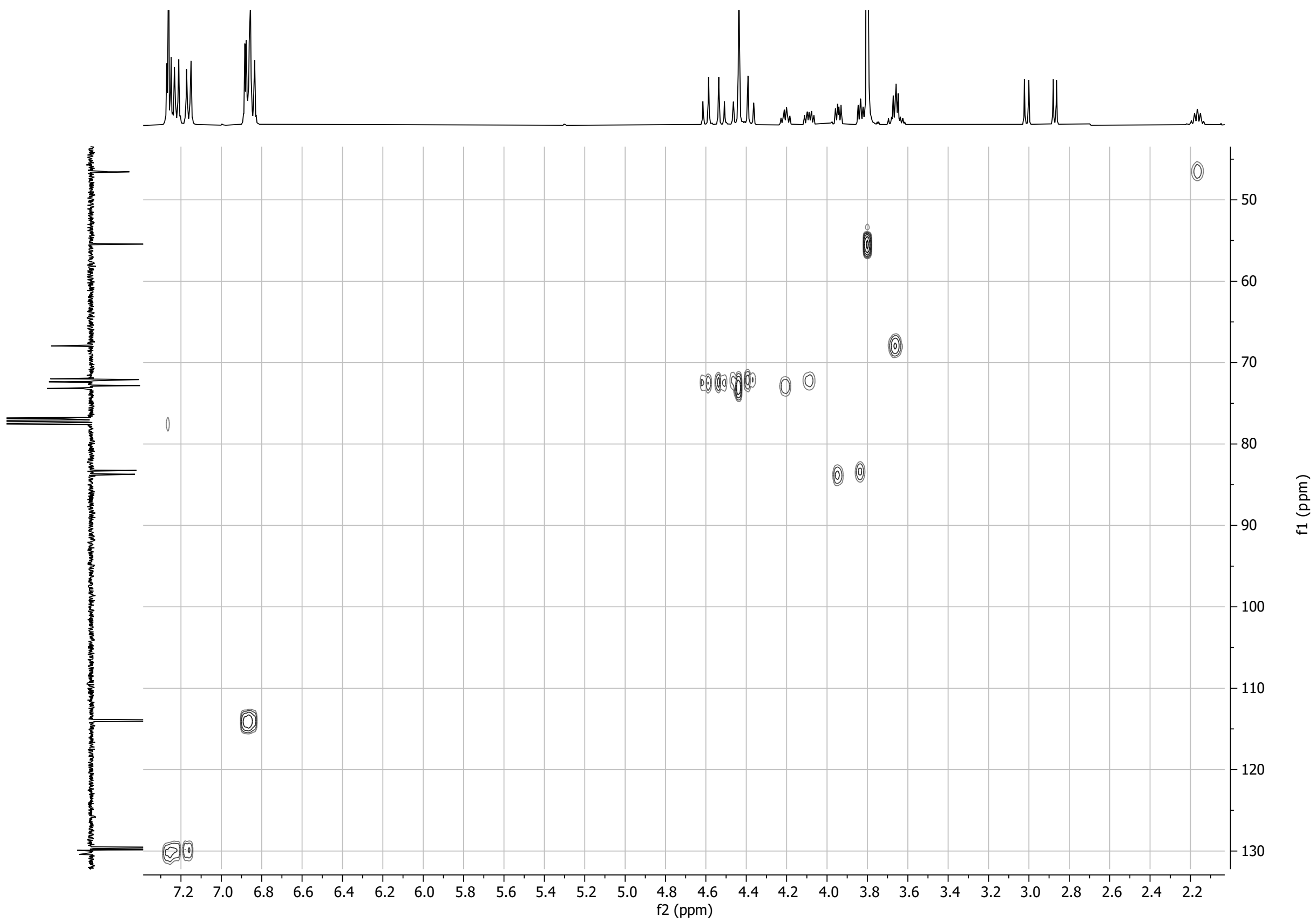

¹H-NMR spectrum of compound 21

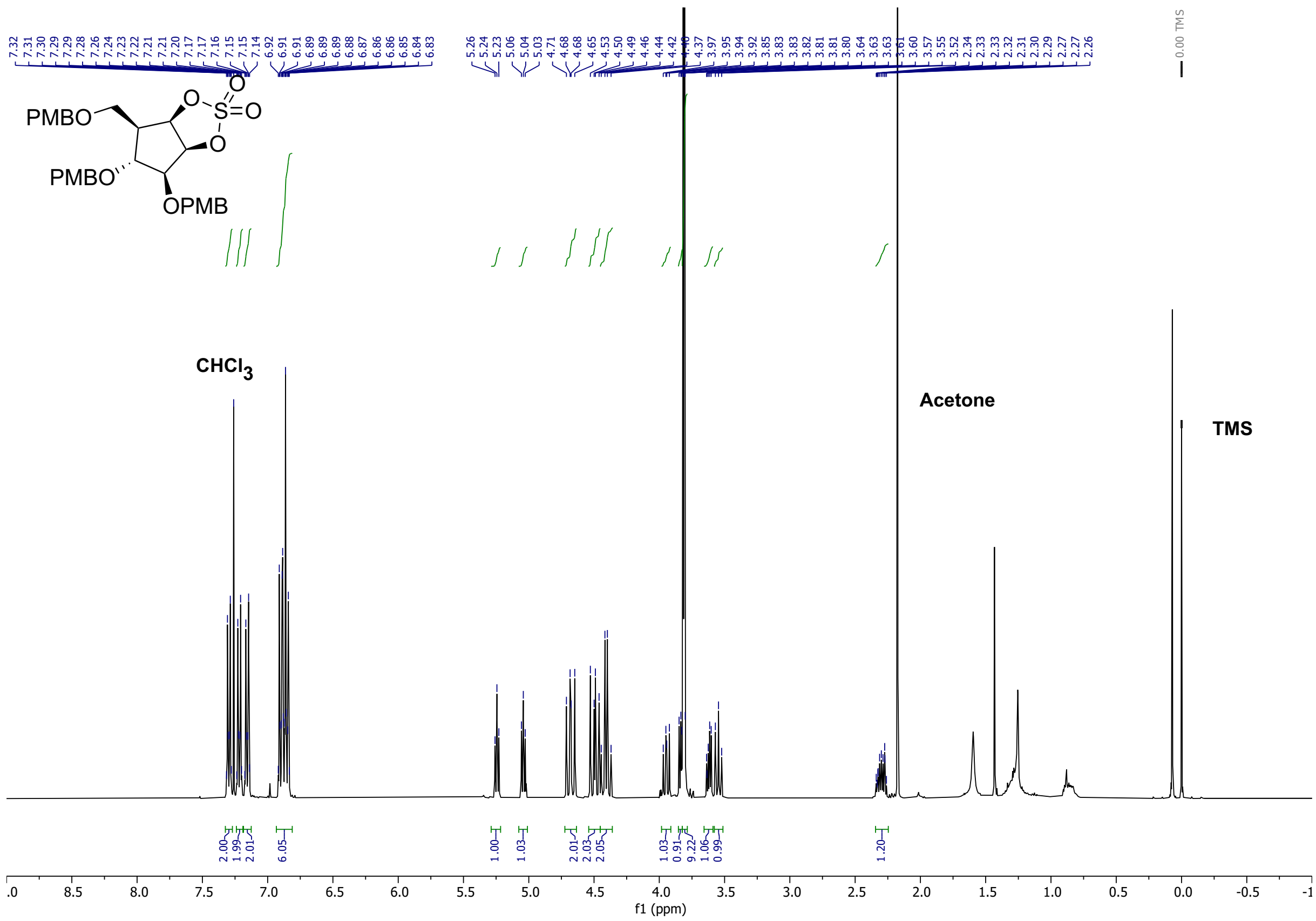

APT-NMR spectrum of compound 21

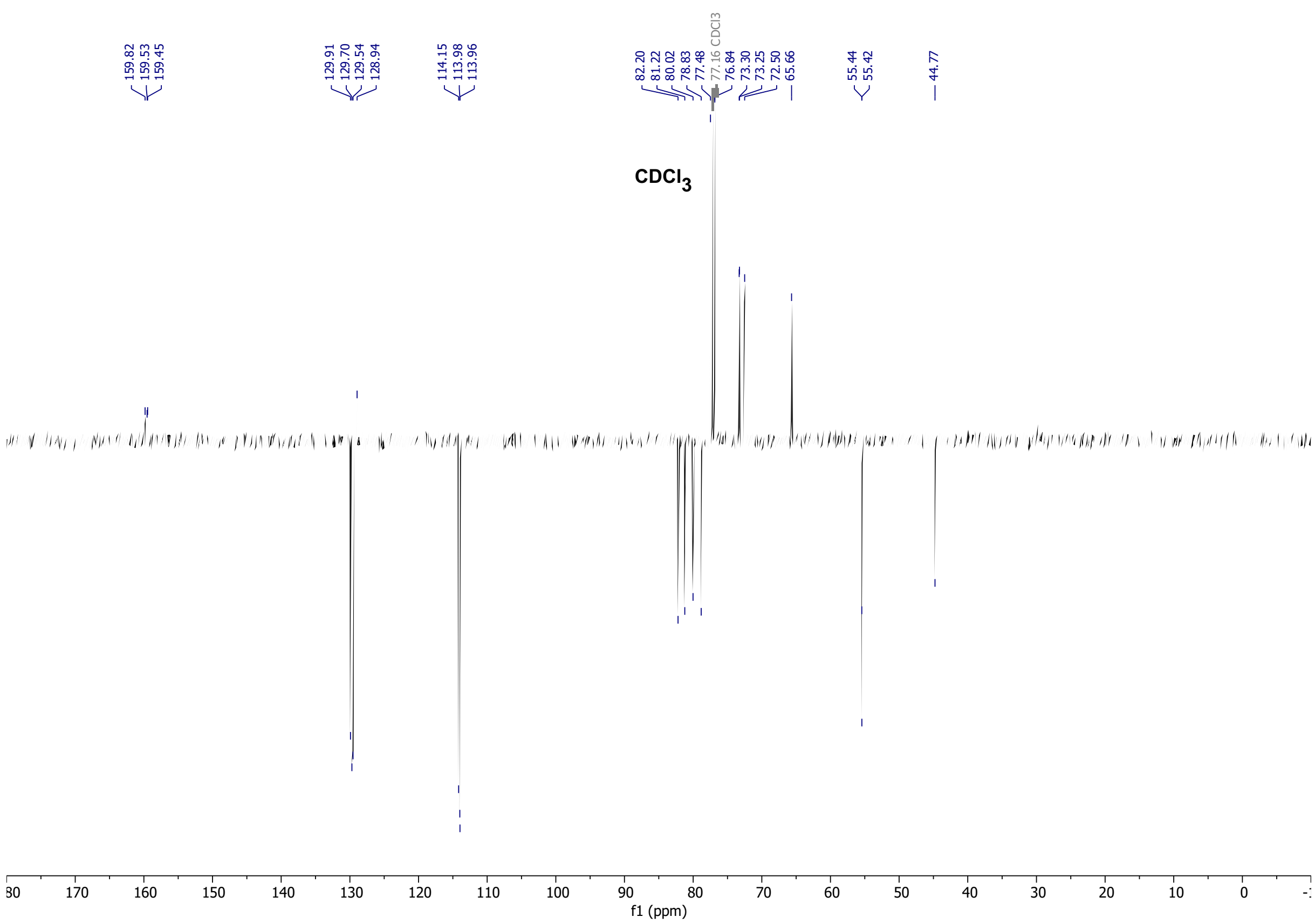

COSY-NMR spectrum of compound 21

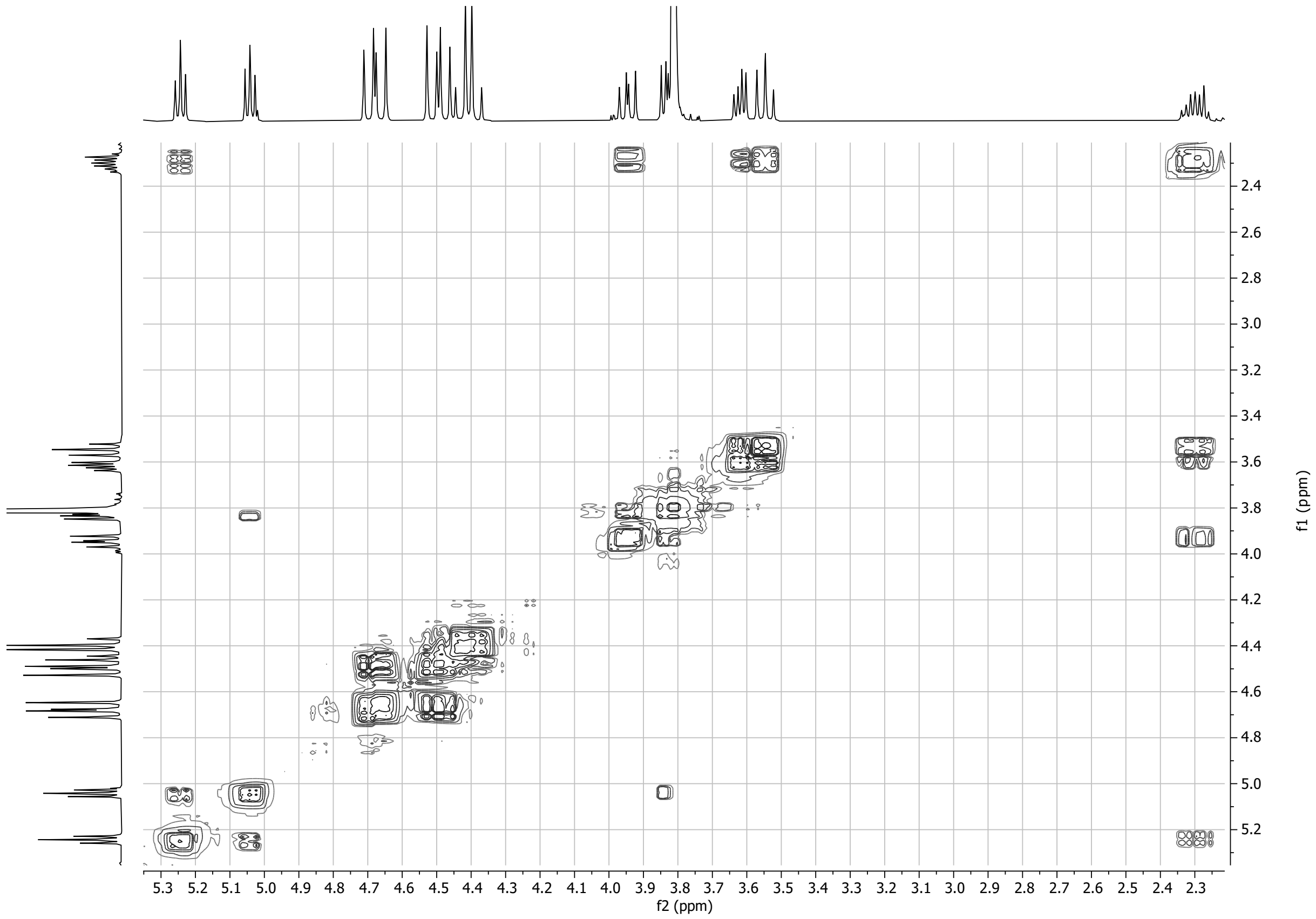

HSQC-NMR spectrum of compound 21

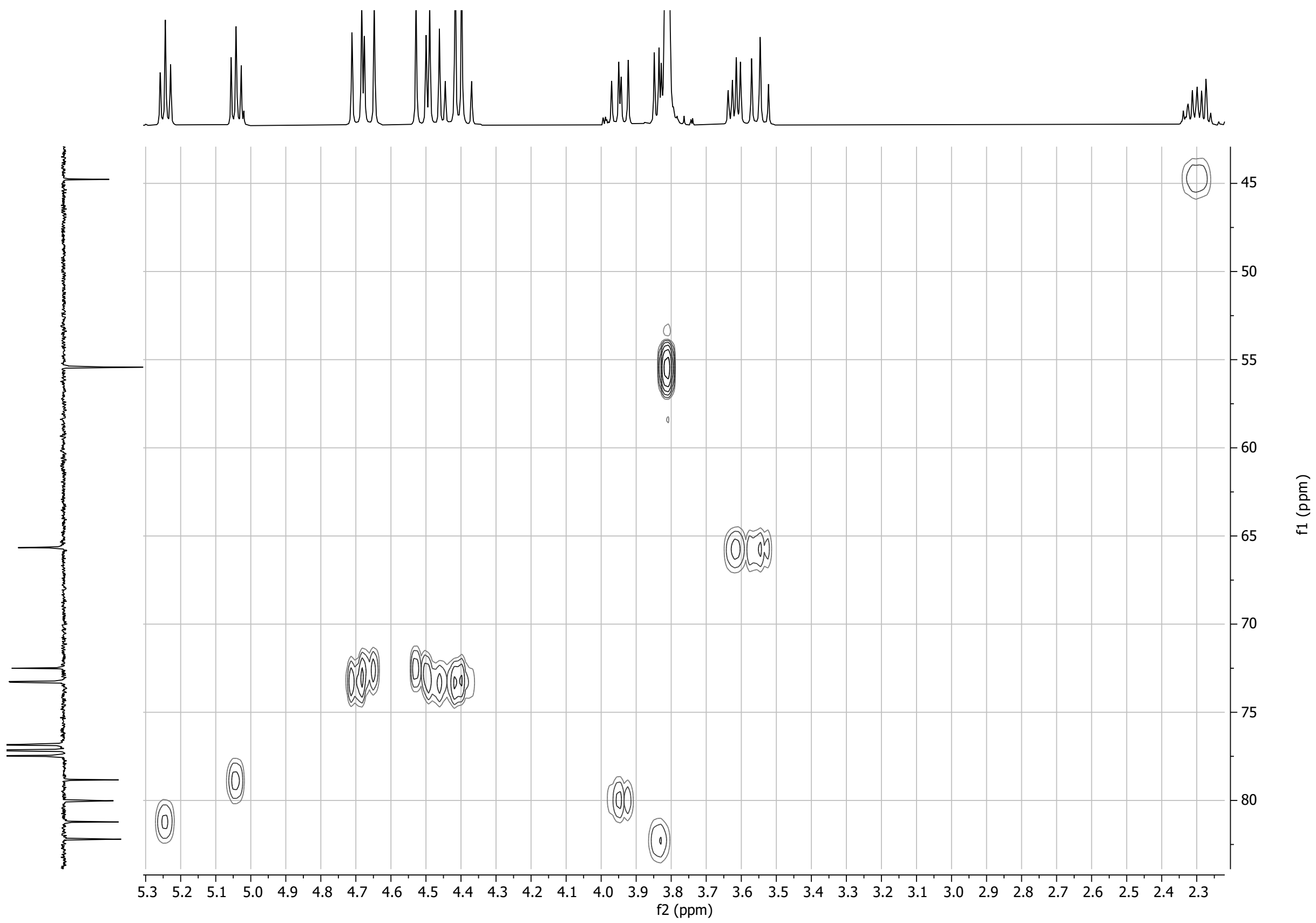

^{1}H-NMR spectrum of compound 2

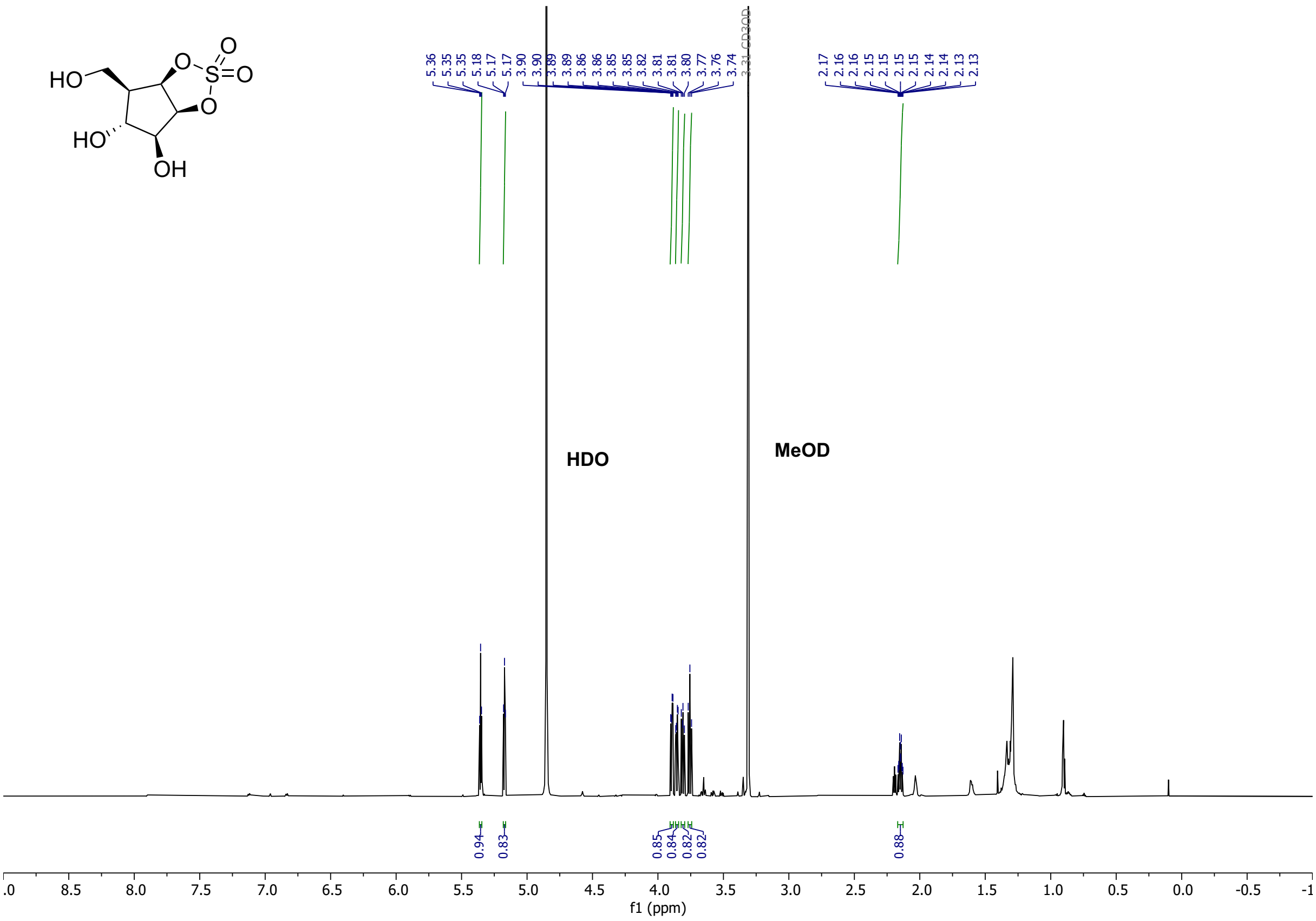

APT-NMR spectrum of compound 2

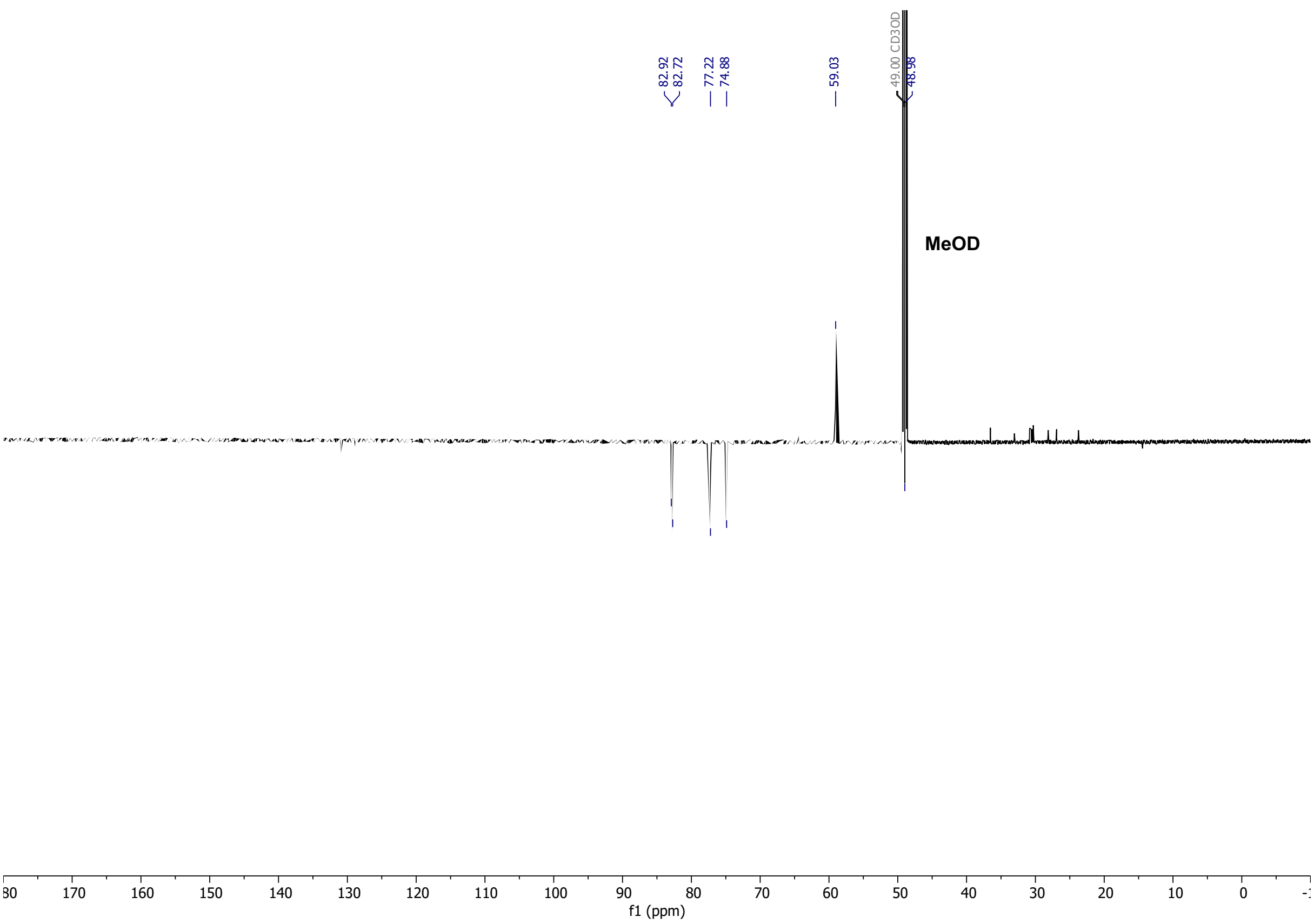

COSY-NMR spectrum of compound 2

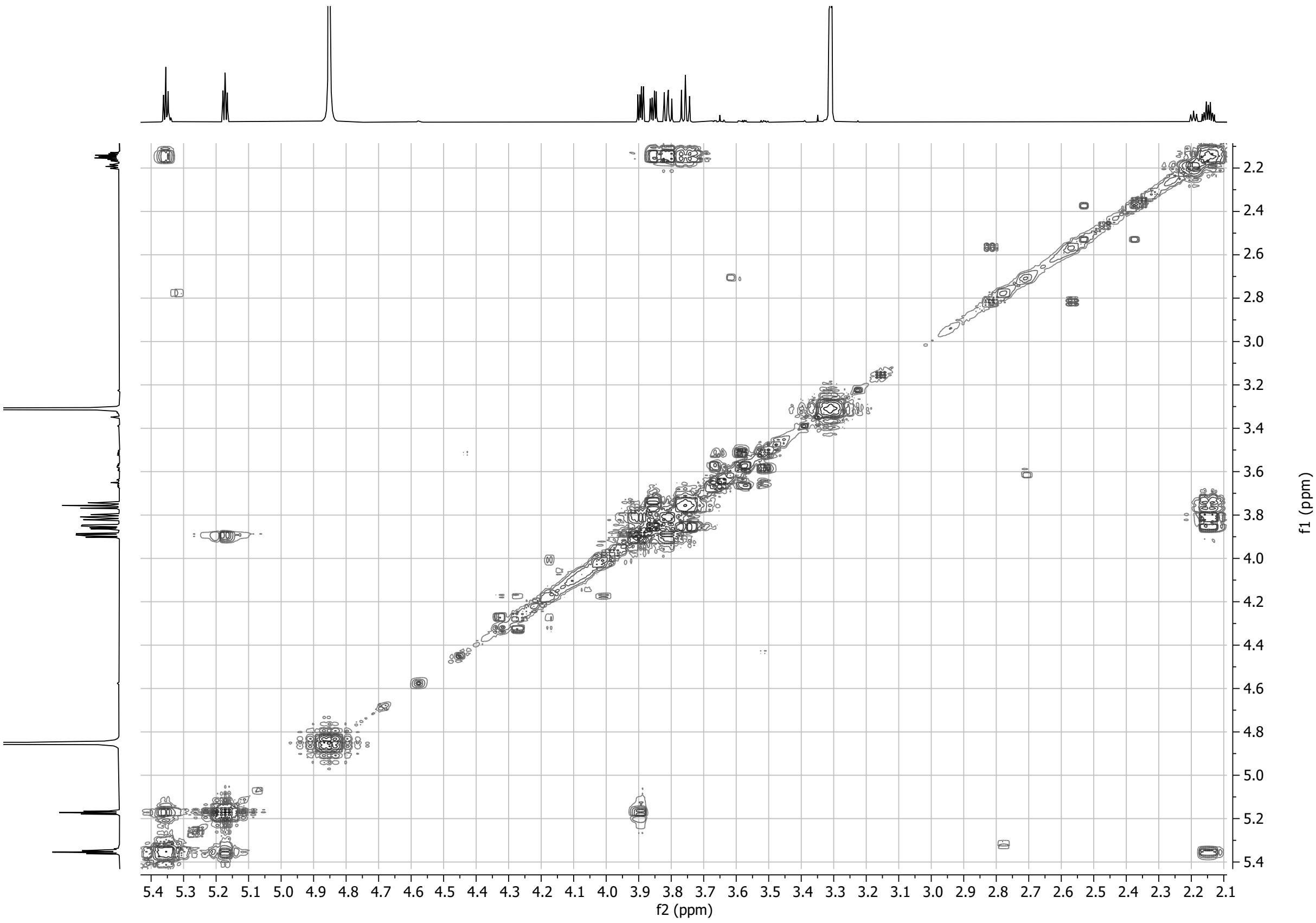

HSQC-NMR spectrum of compound 2

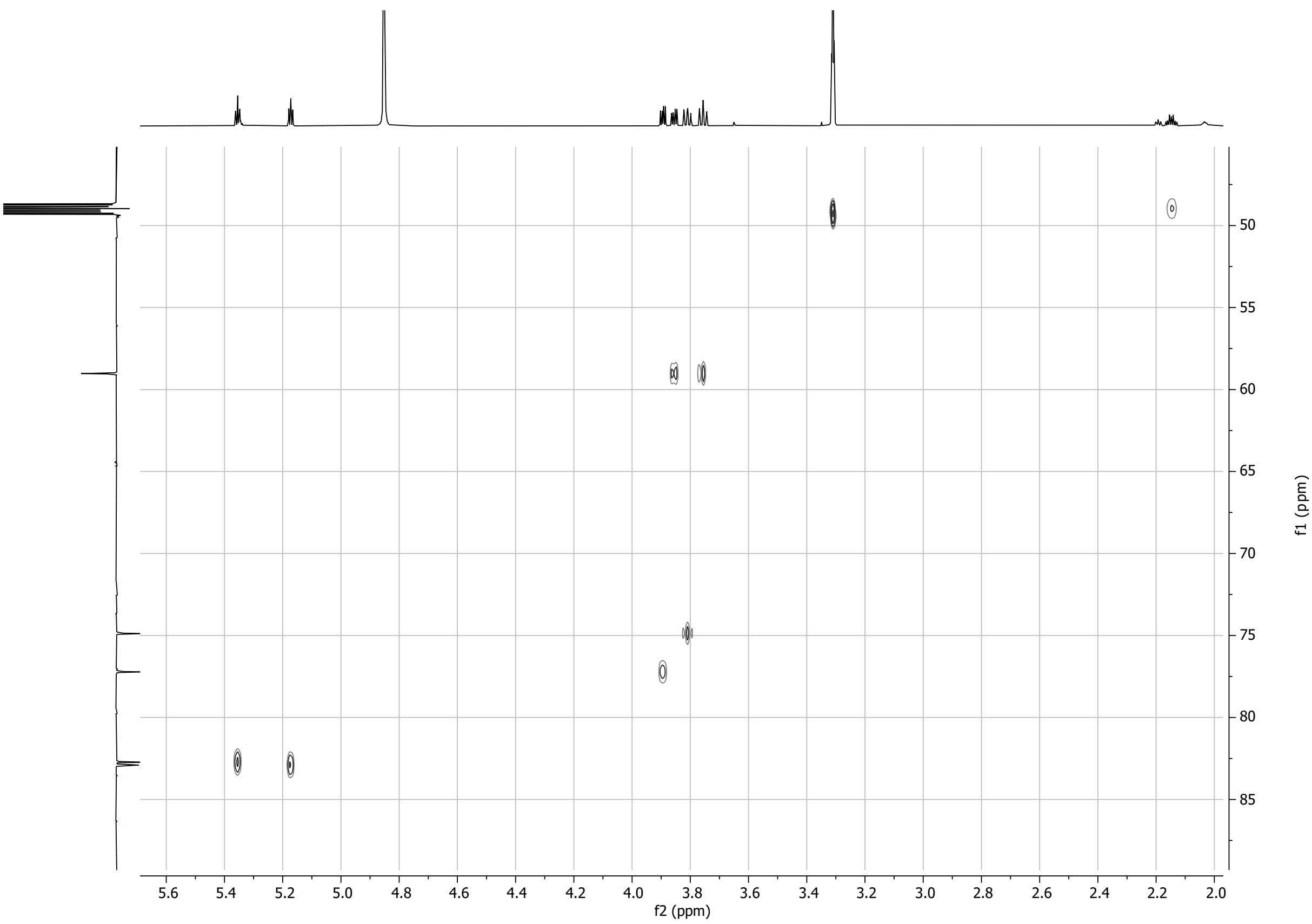

^{1}H-NMR spectrum of compound 22

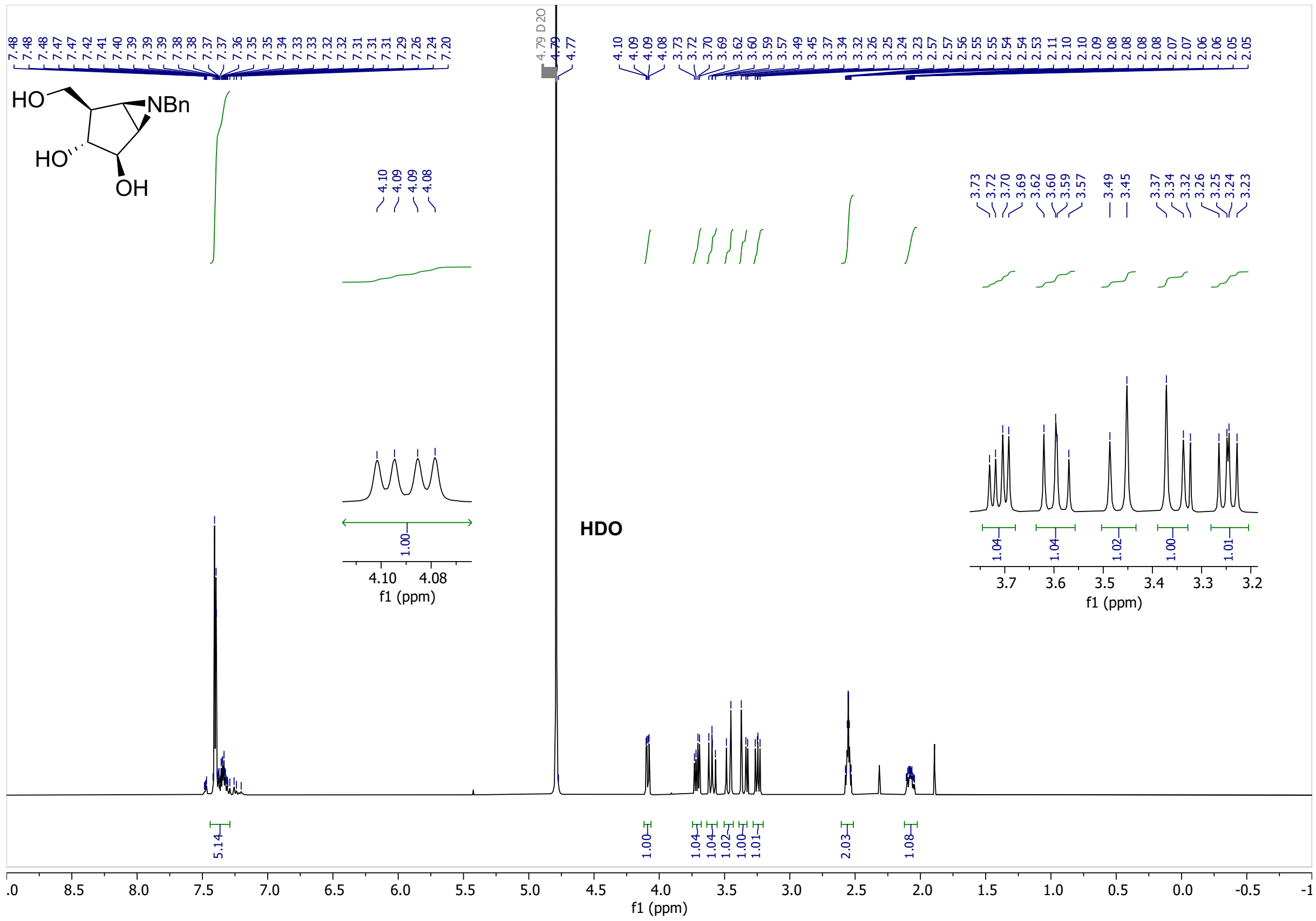

APT-NMR spectrum of compound 22

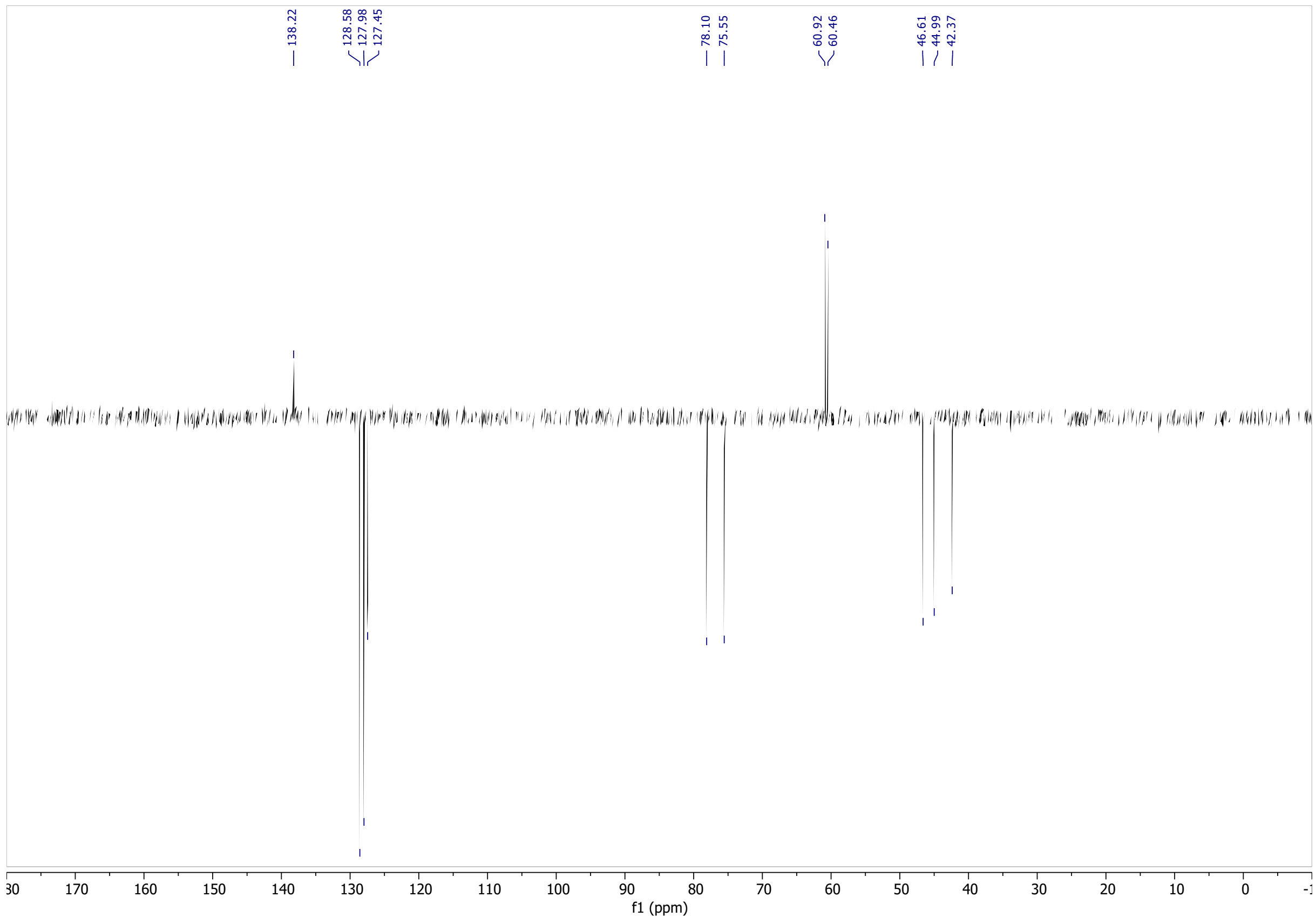

^{1}H-NMR spectrum of compound 3

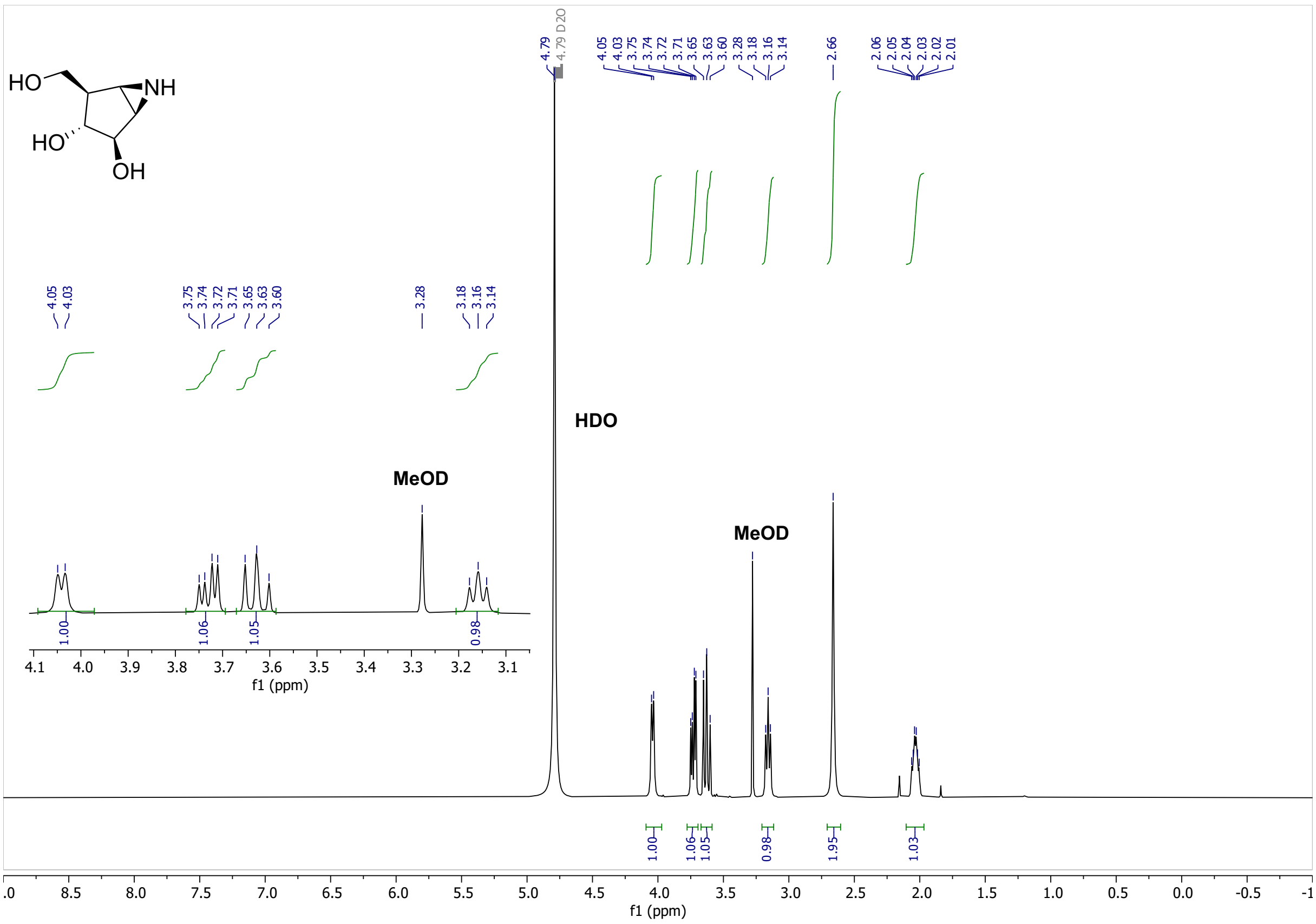

APT-NMR spectrum of compound 3

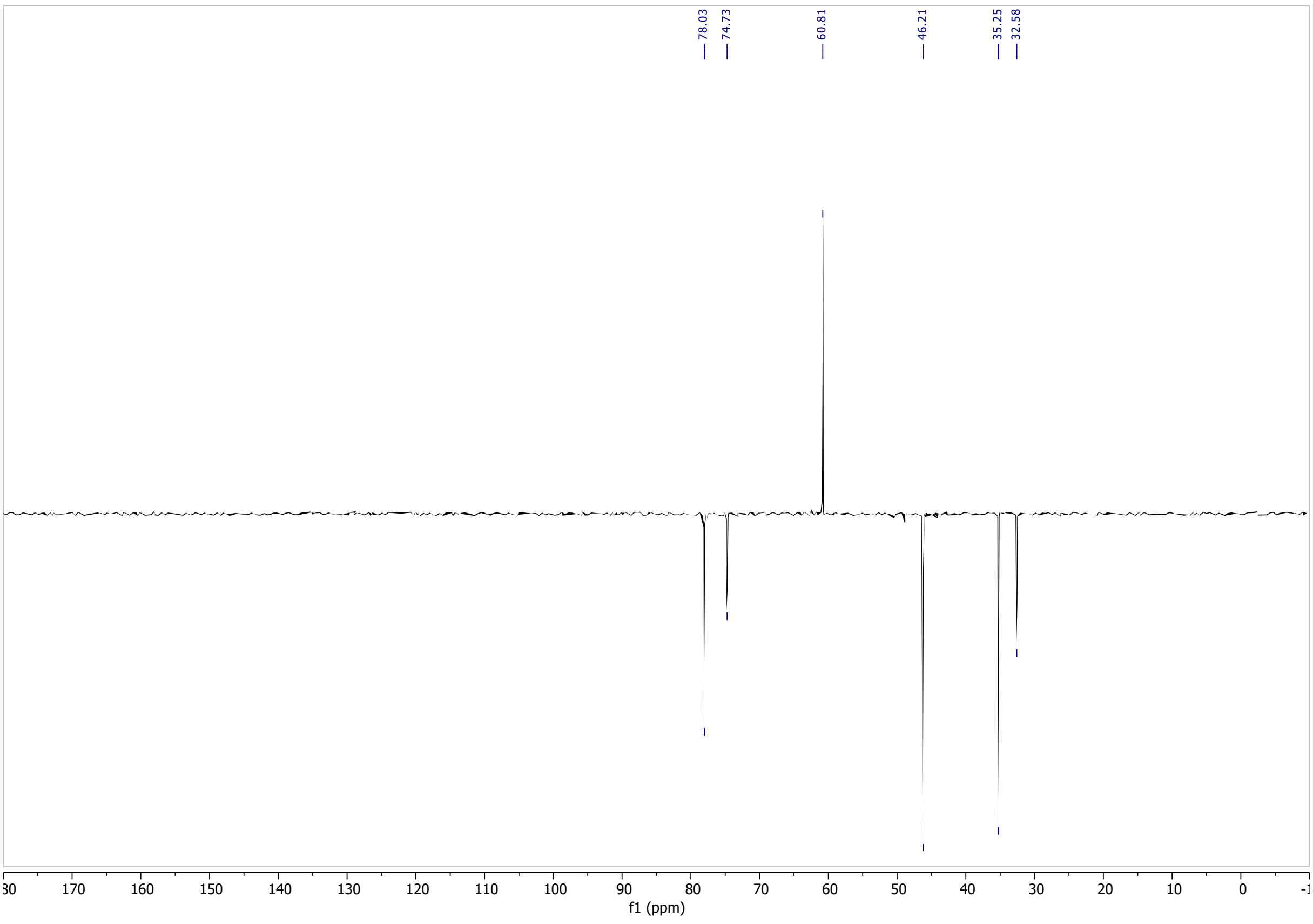

1H-NMR spectrum of compound 4

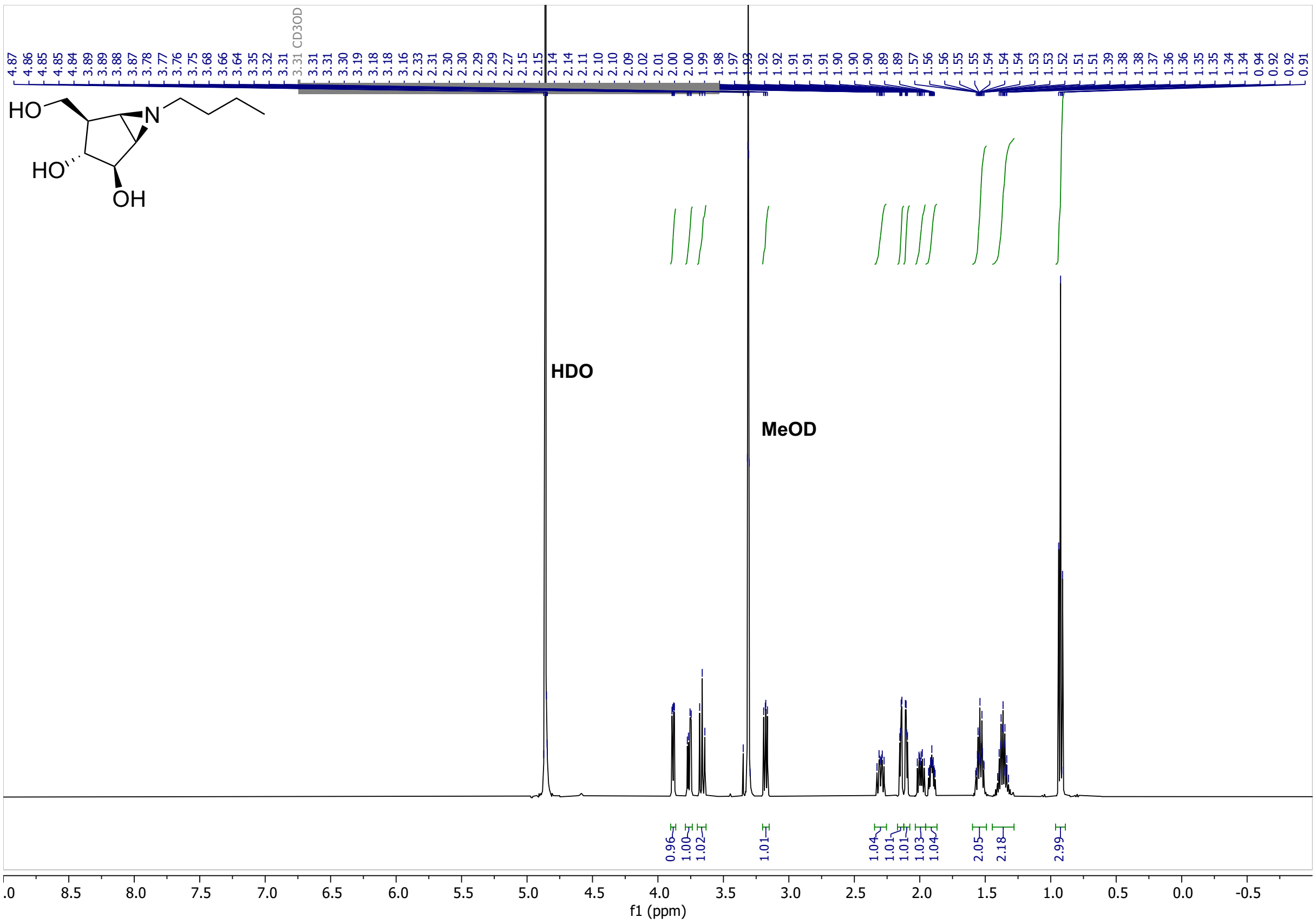

APT-NMR spectrum of compound 4

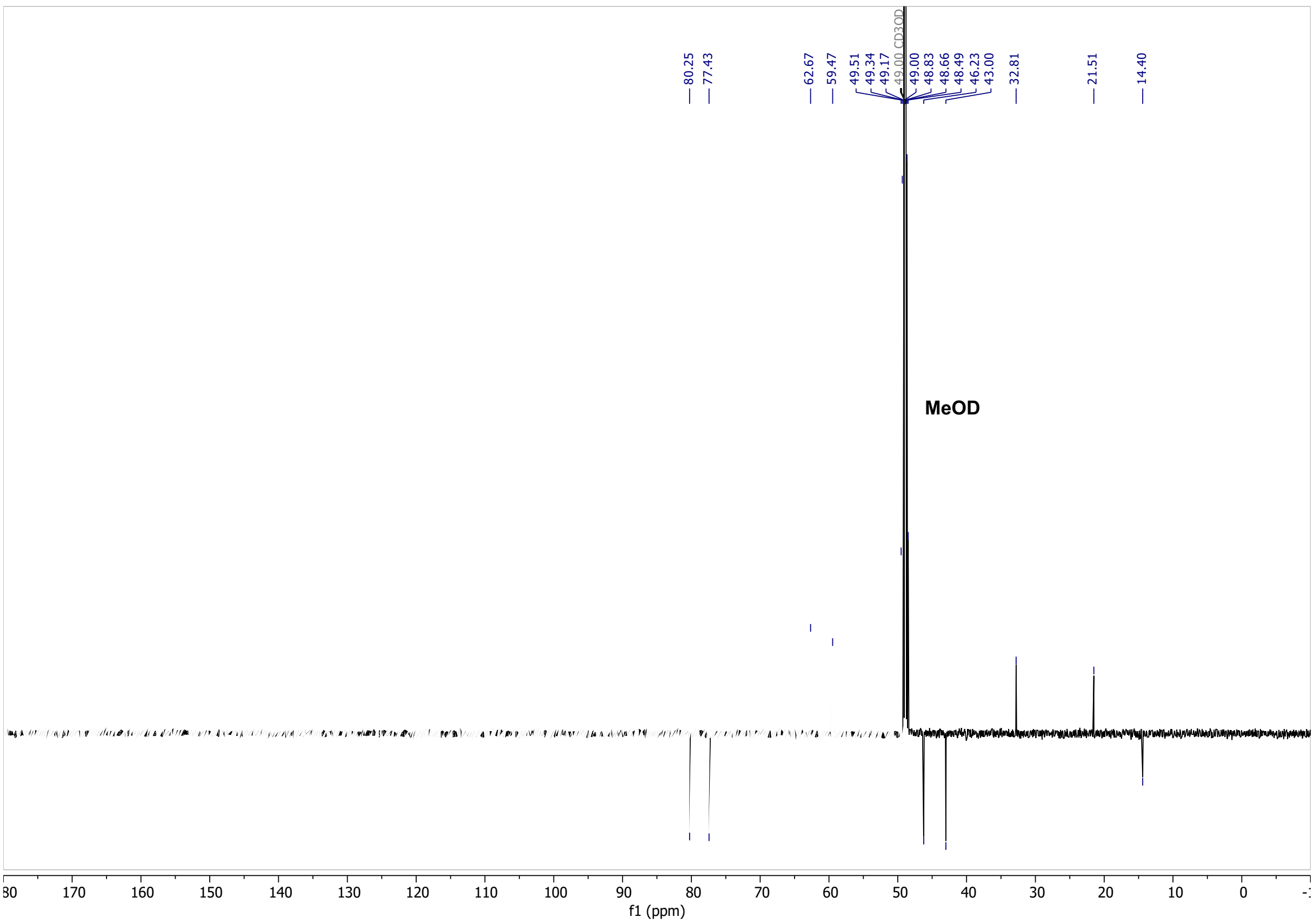

COSY-NMR spectrum of compound 4

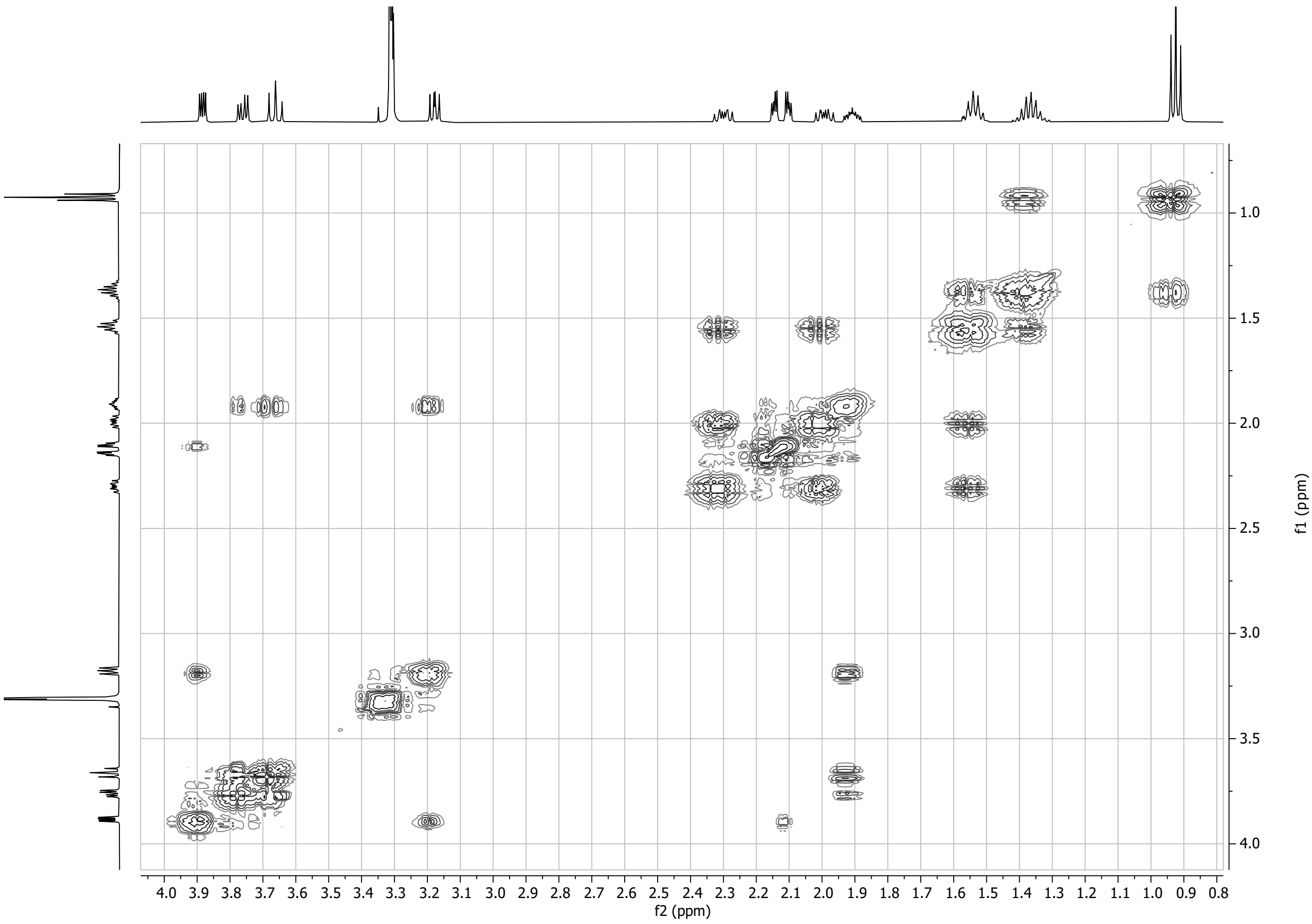

HSQC-NMR spectrum of compound 4

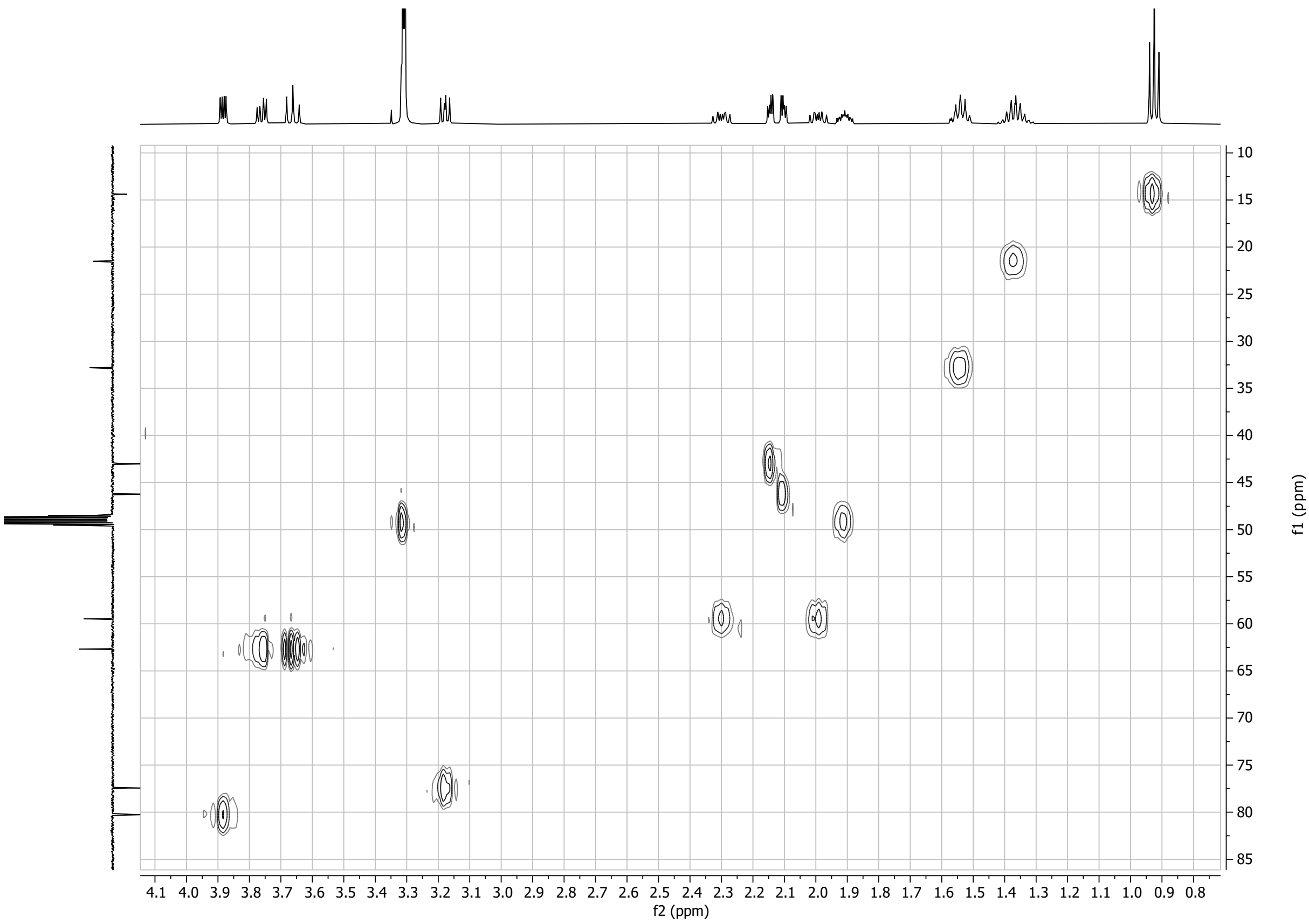

¹H-NMR spectrum of compound 28

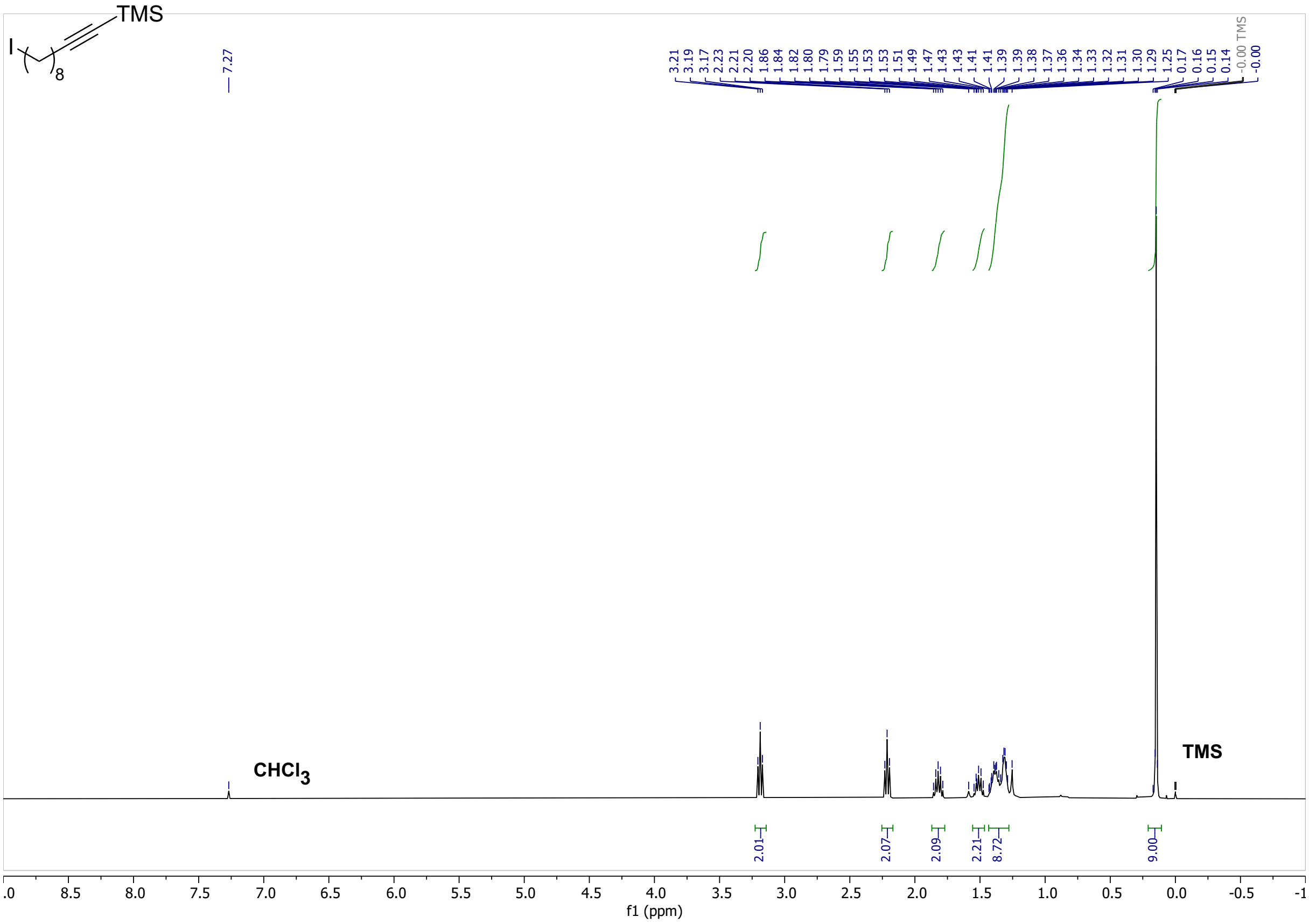

APT-NMR spectrum of compound 28

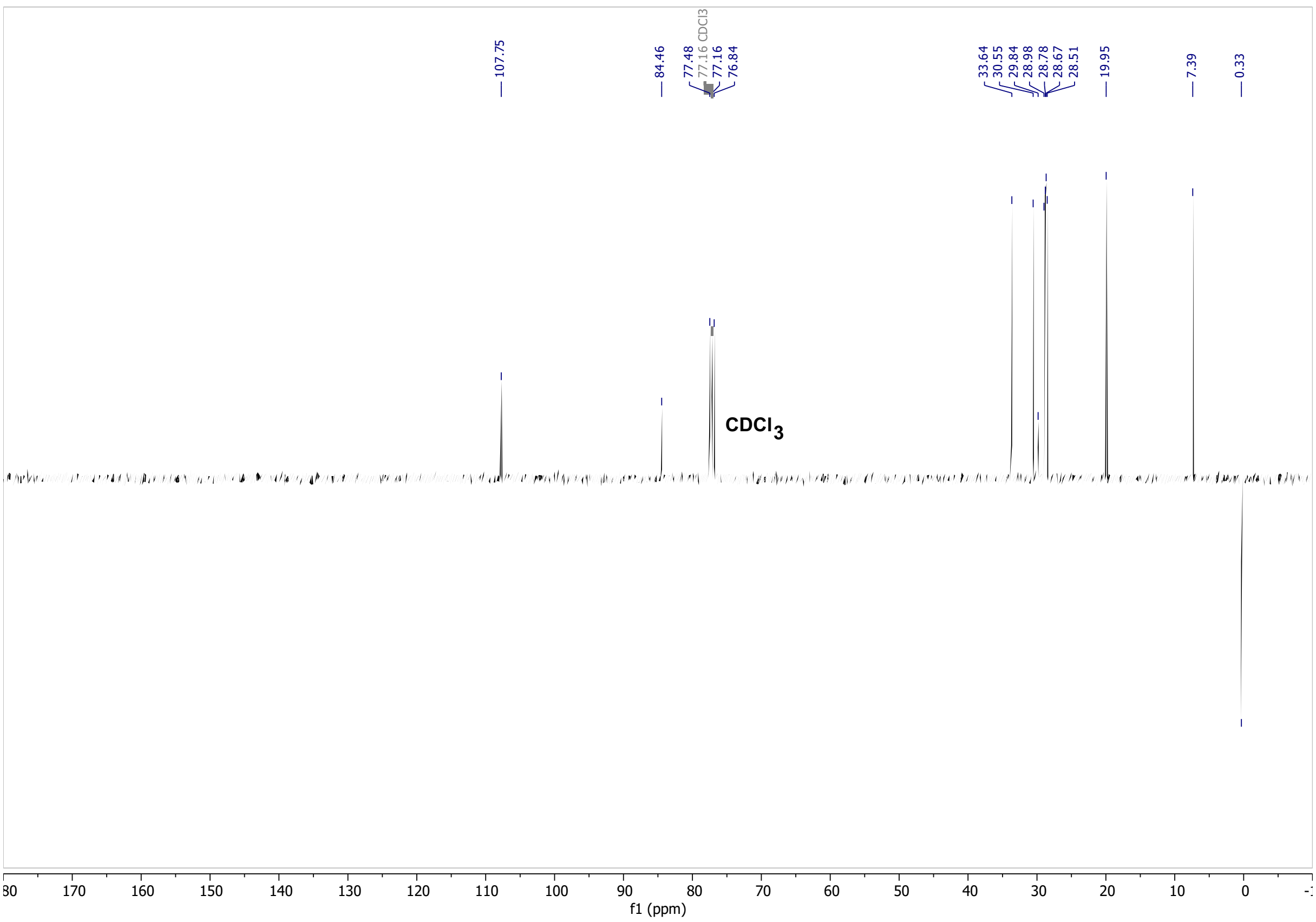

COSY-NMR spectrum of compound 28

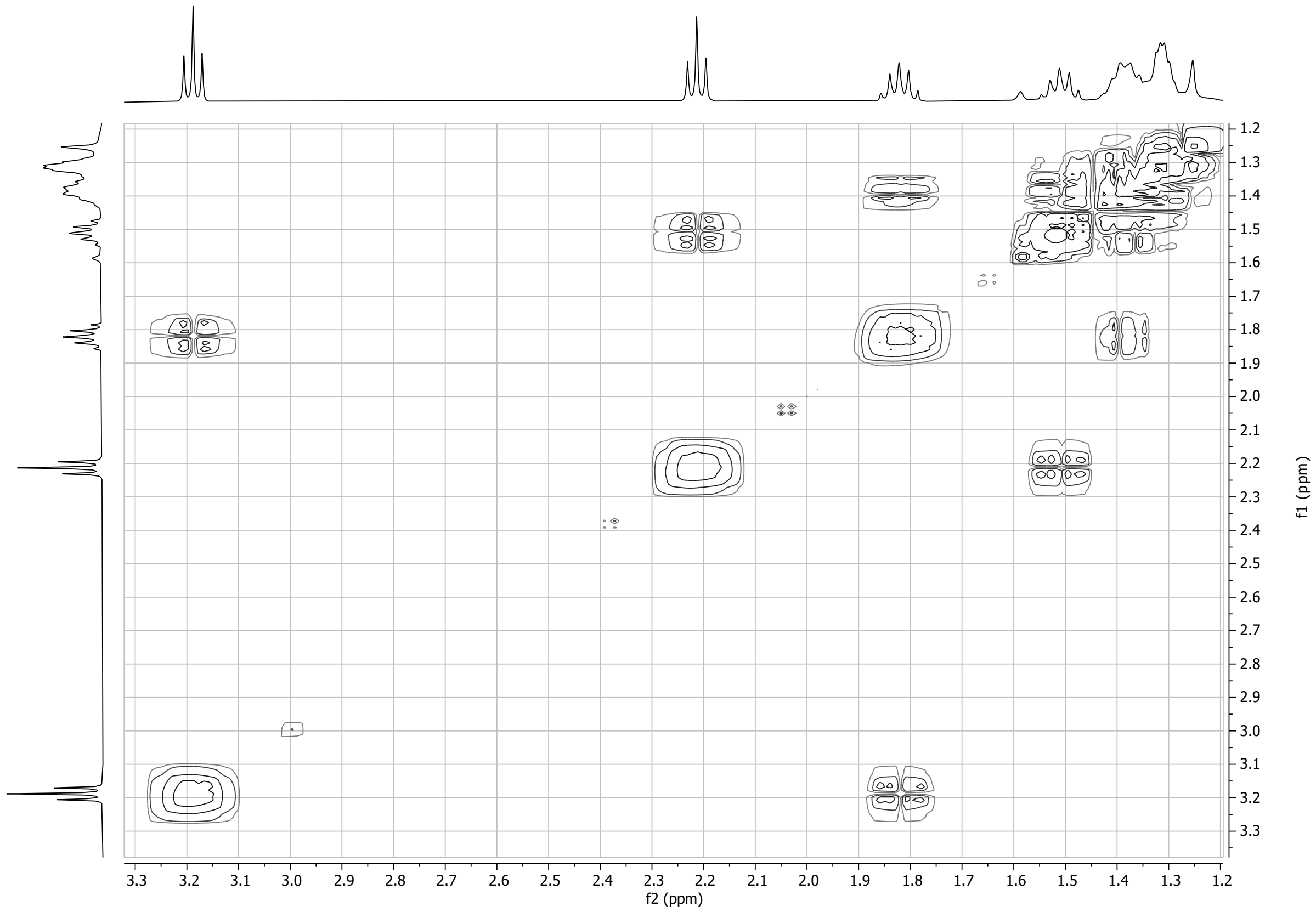

HSQC-NMR spectrum of compound 28

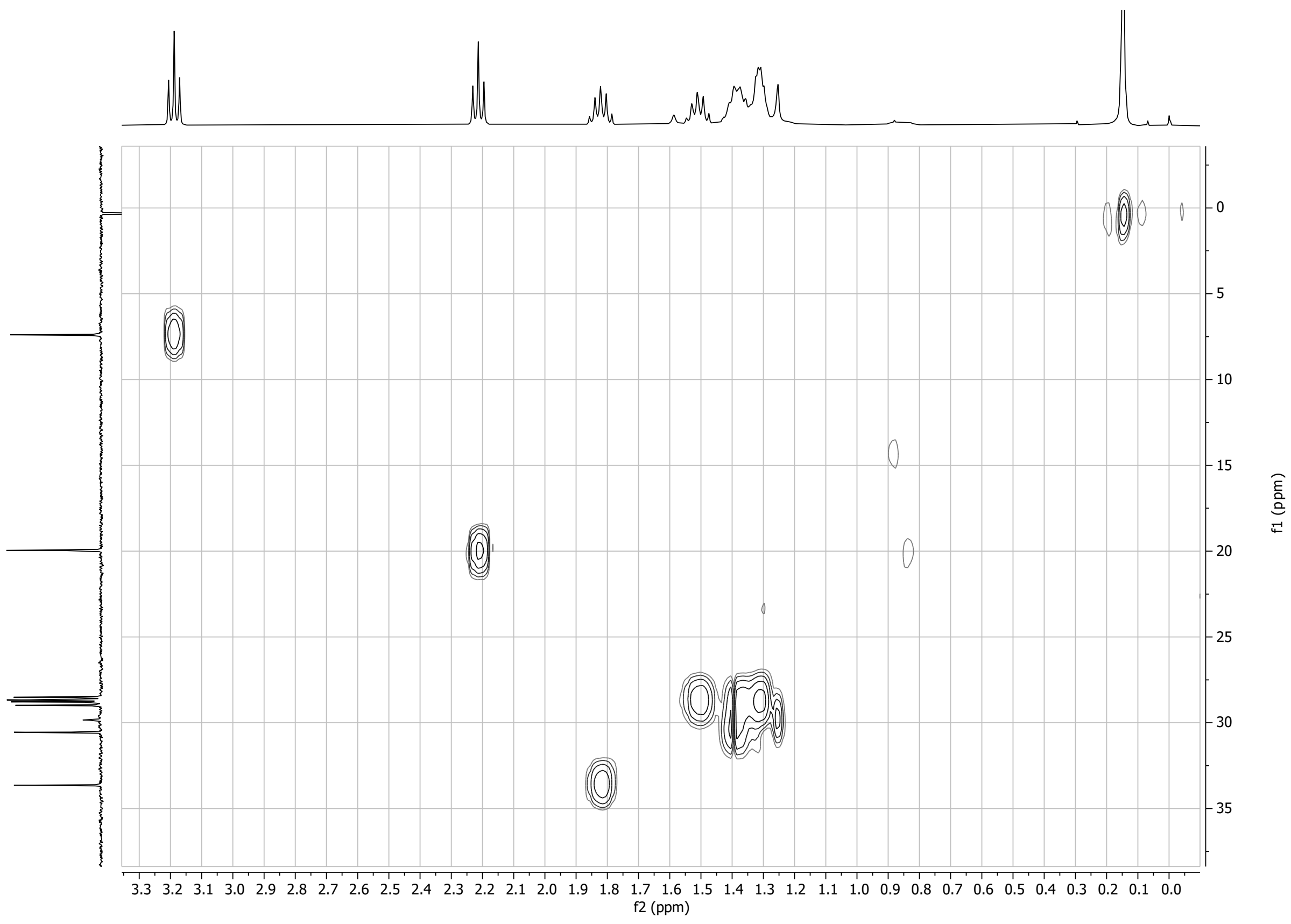

¹H-NMR spectrum of compound 5 (Batch 1)

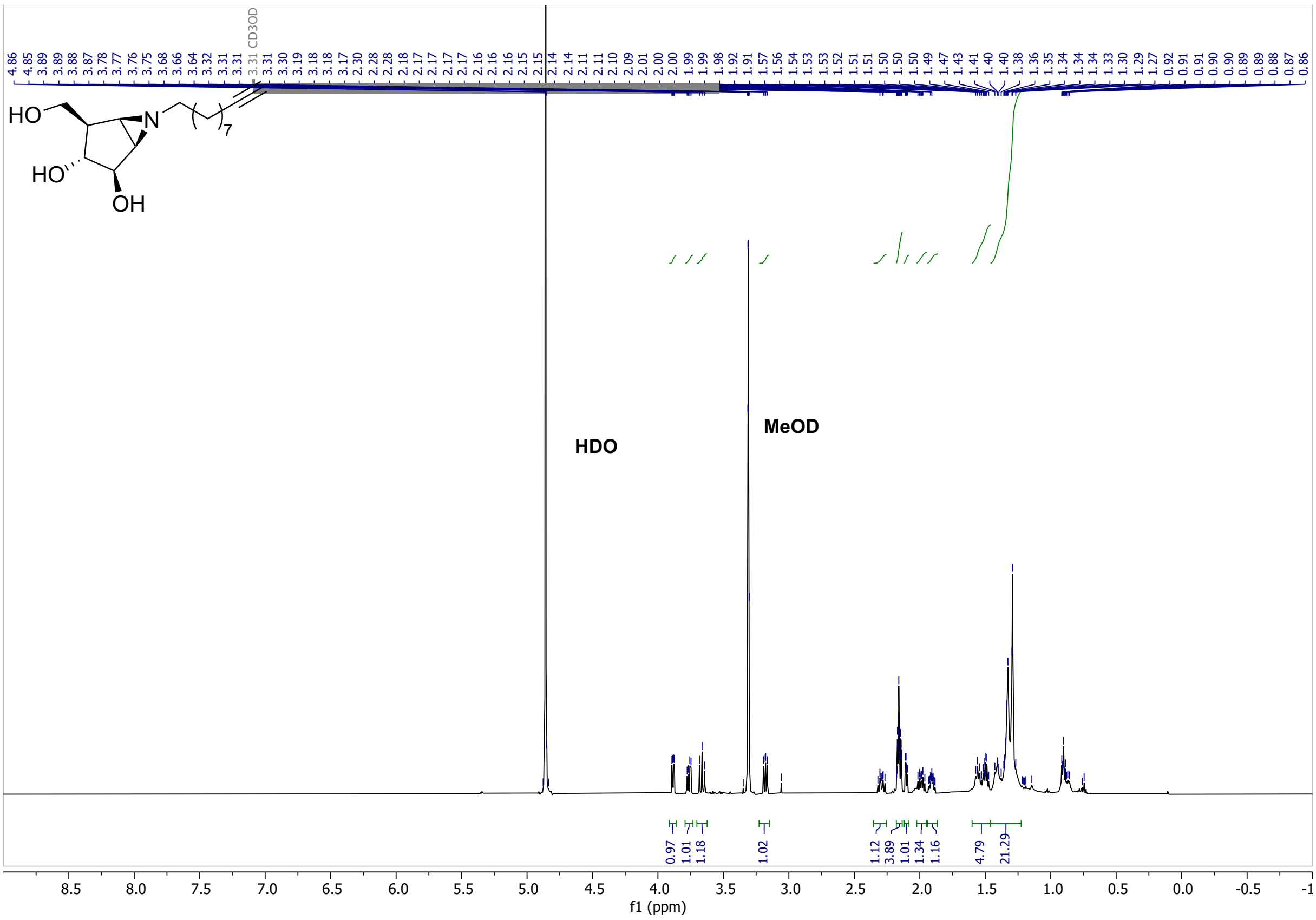

APT-NMR spectrum of compound 5 (Batch 1)

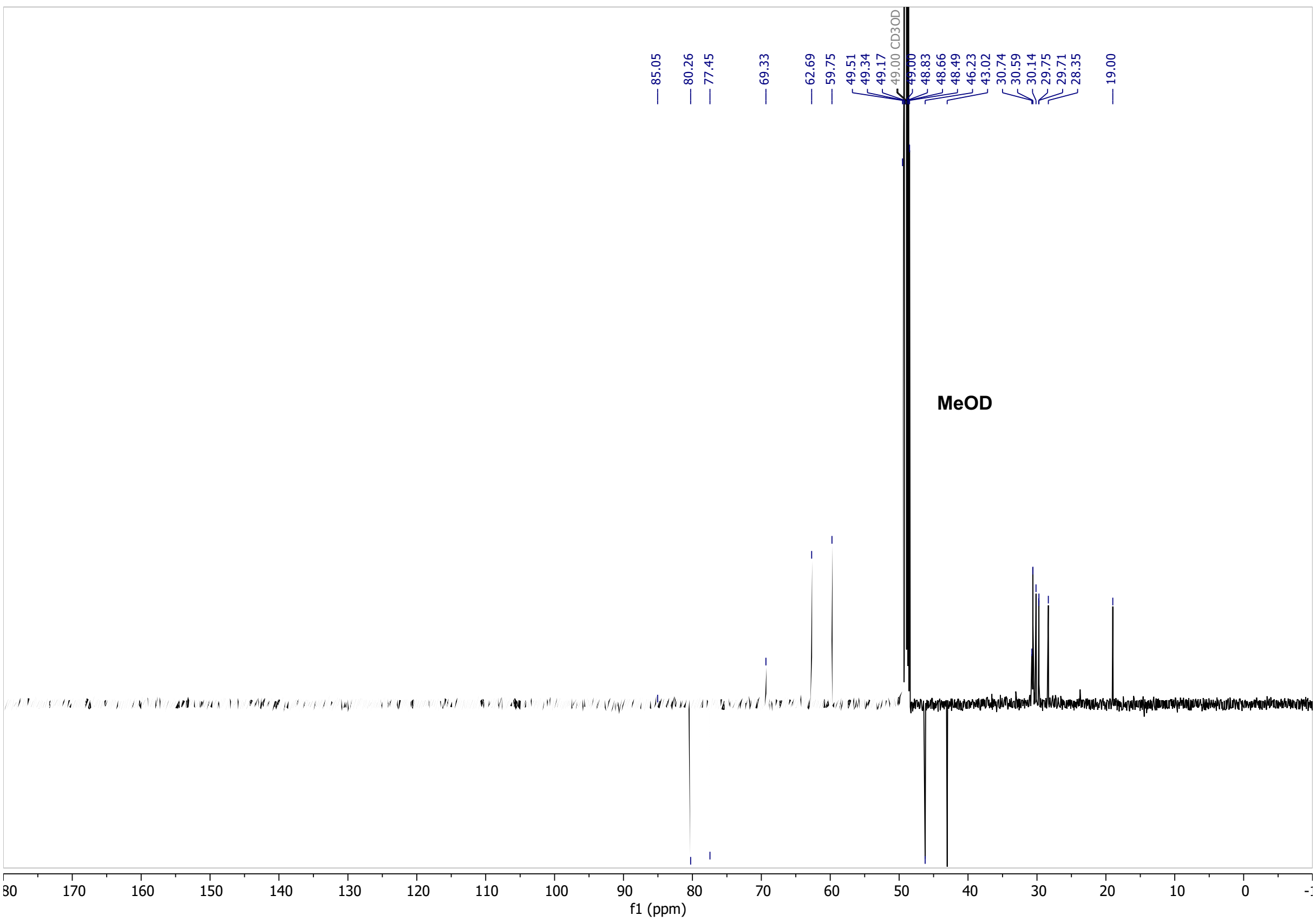

COSY-NMR spectrum of compound 5 (Batch 1)

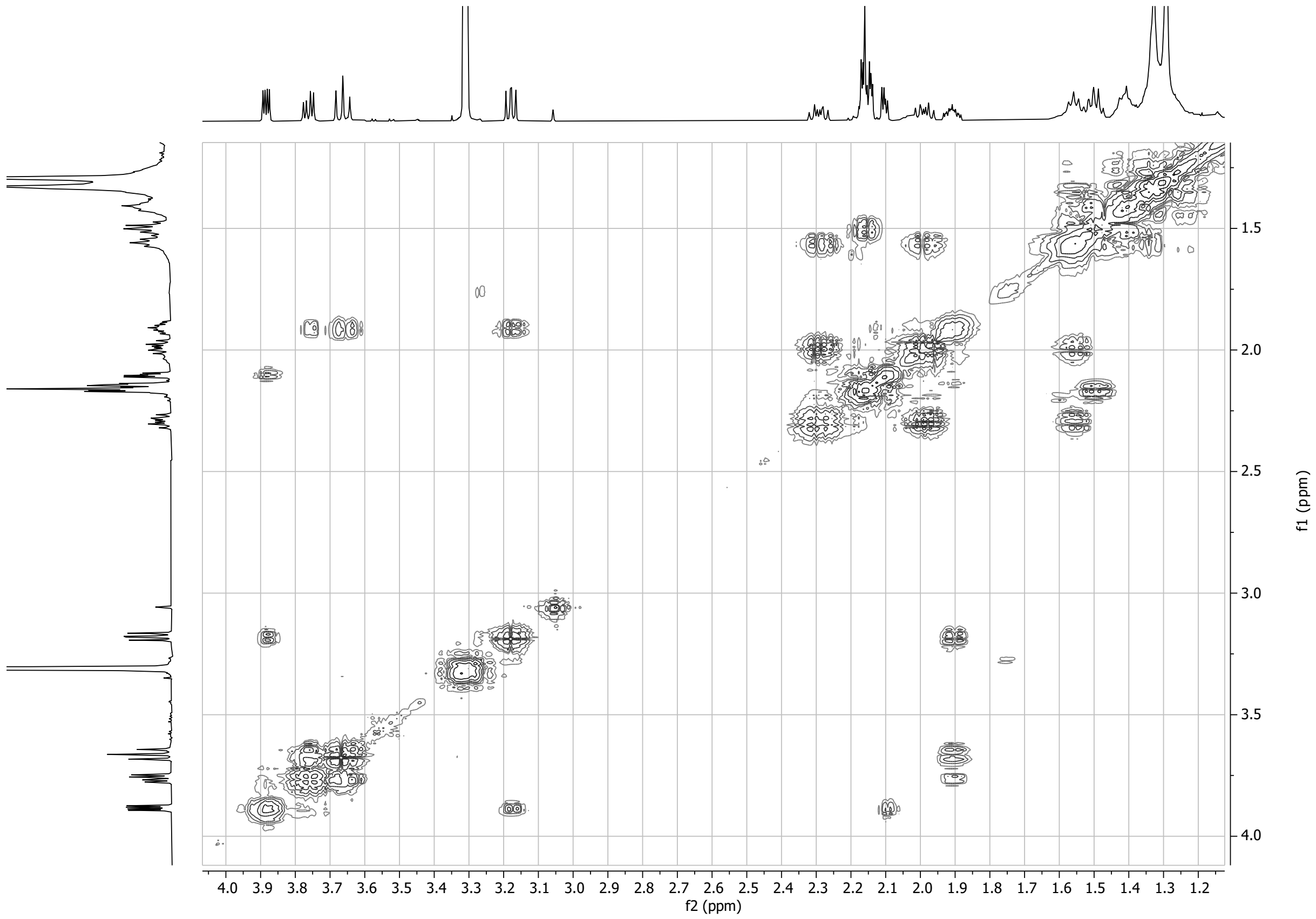

HSQC-NMR spectrum of compound 5 (Batch 1)

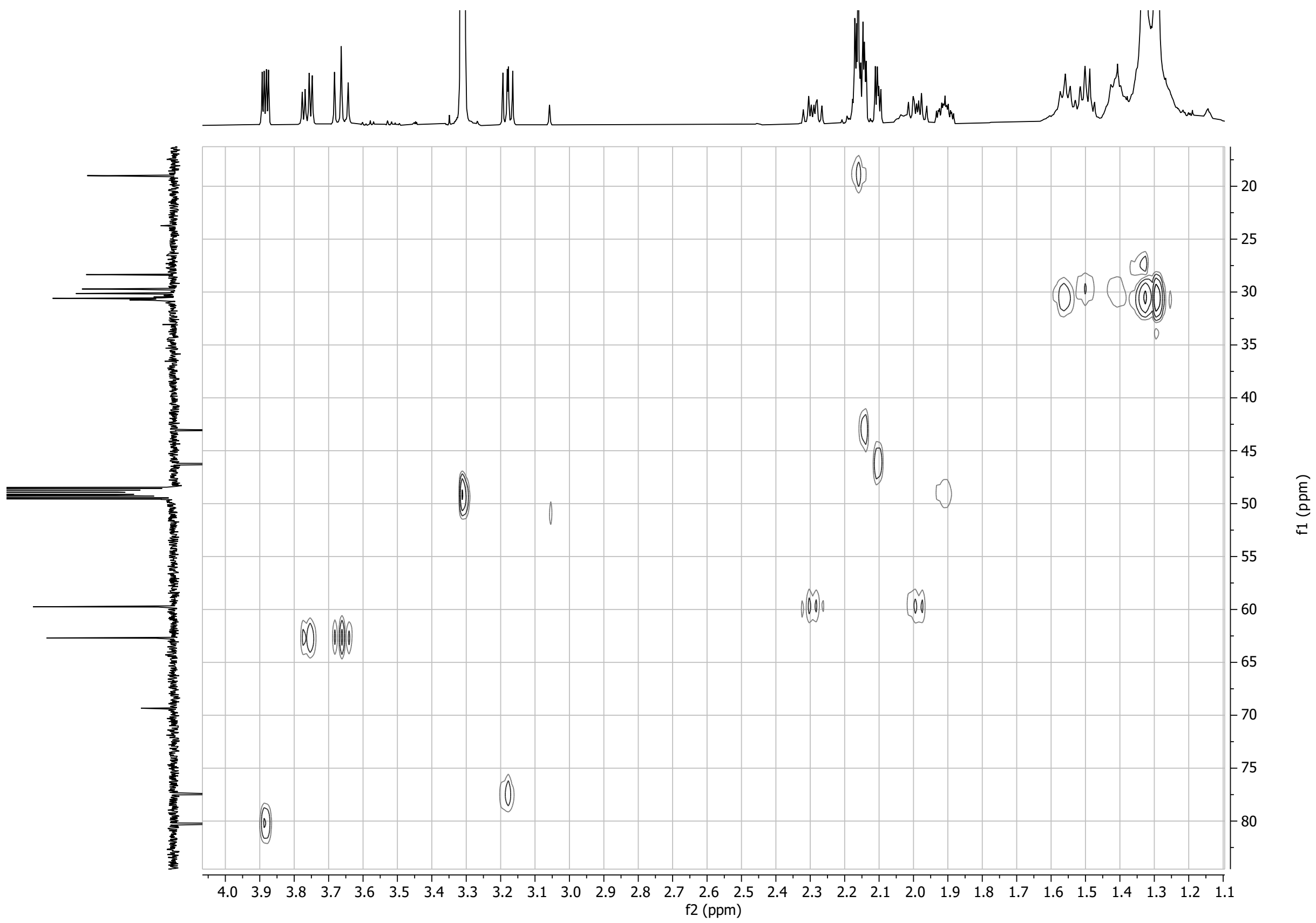

¹H-NMR spectrum of compound 5 (Batch 2)

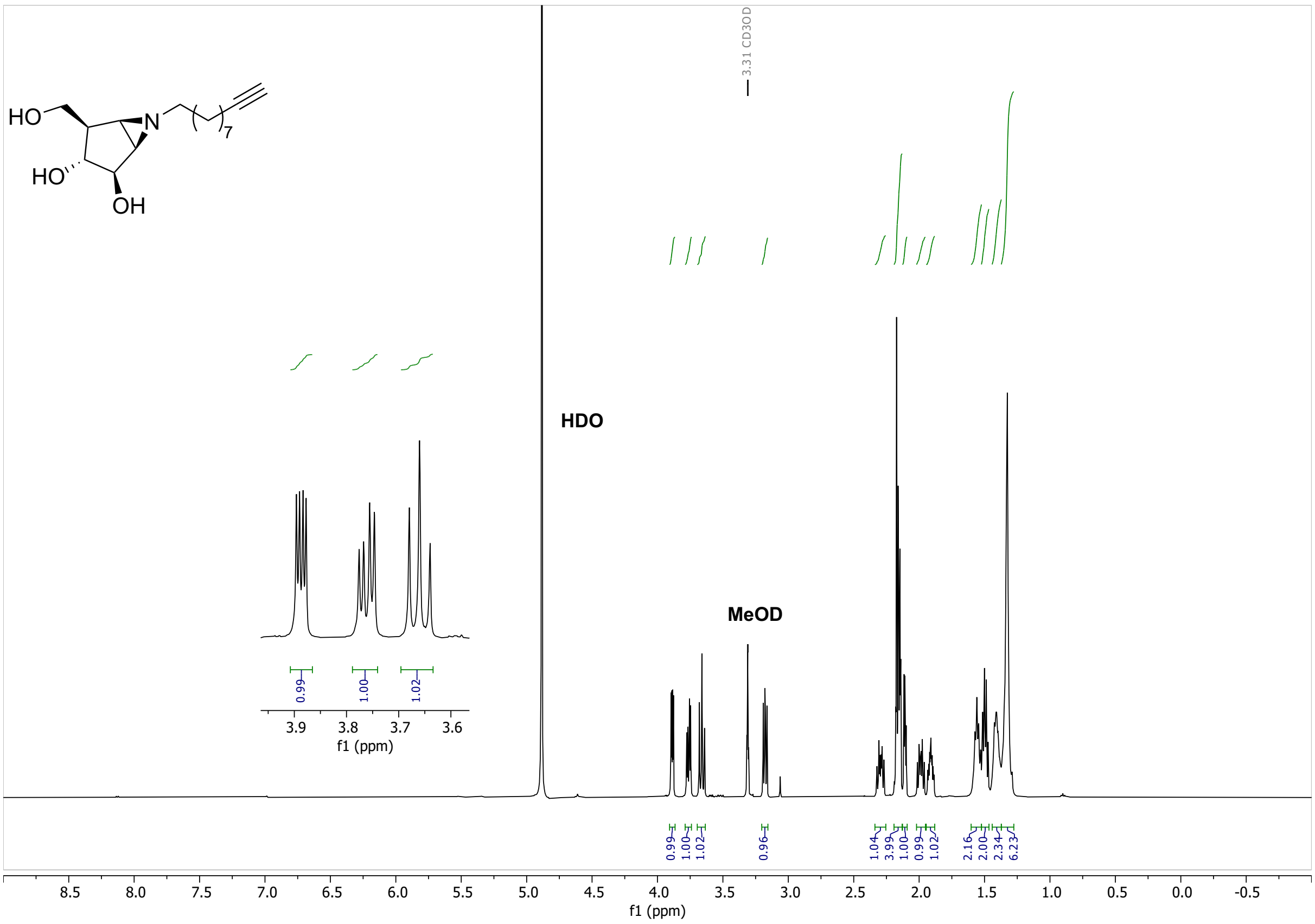

APT-NMR spectrum of compound 5 (Batch 2)

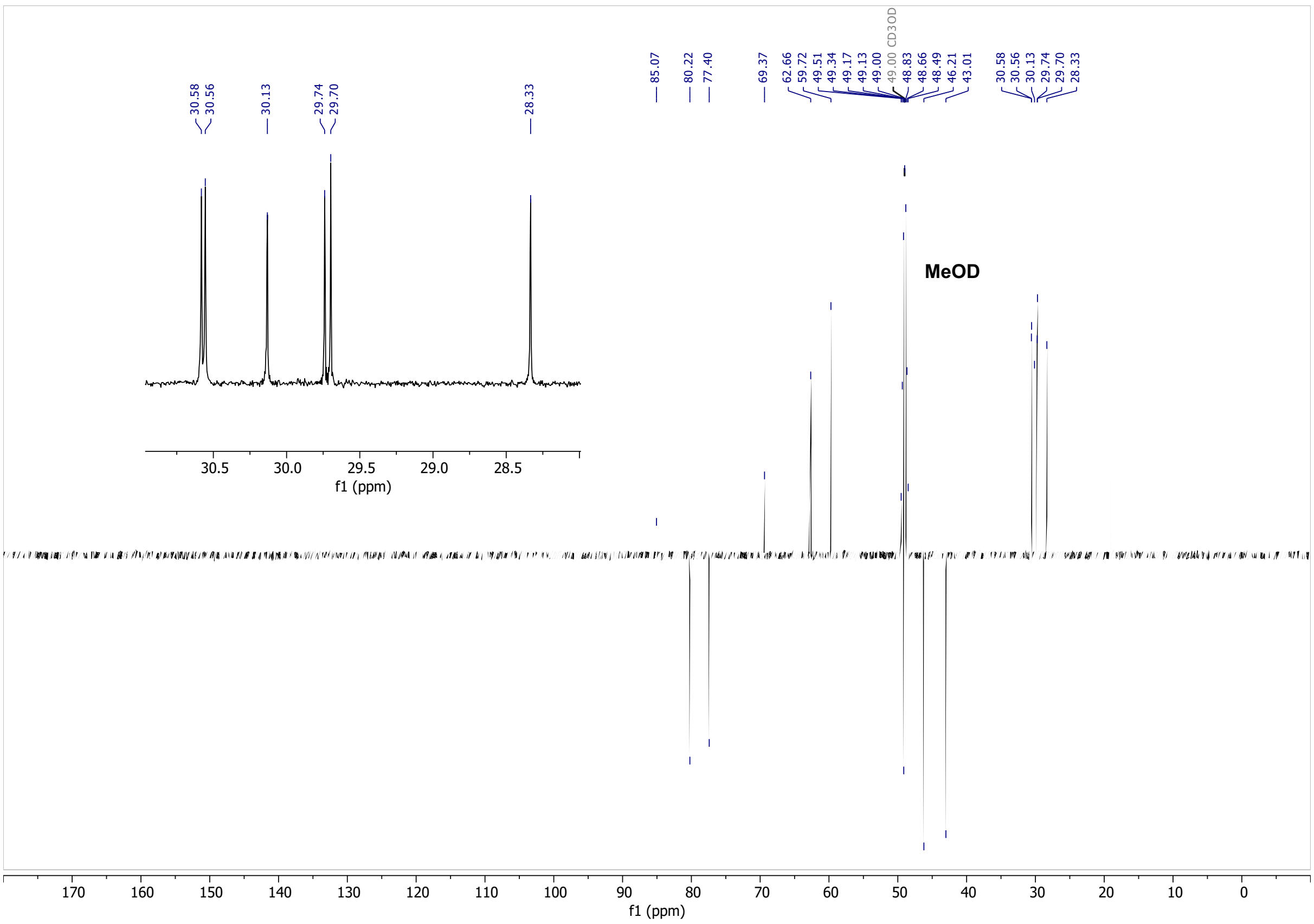

COSY-NMR spectrum of compound 5 (Batch 2)

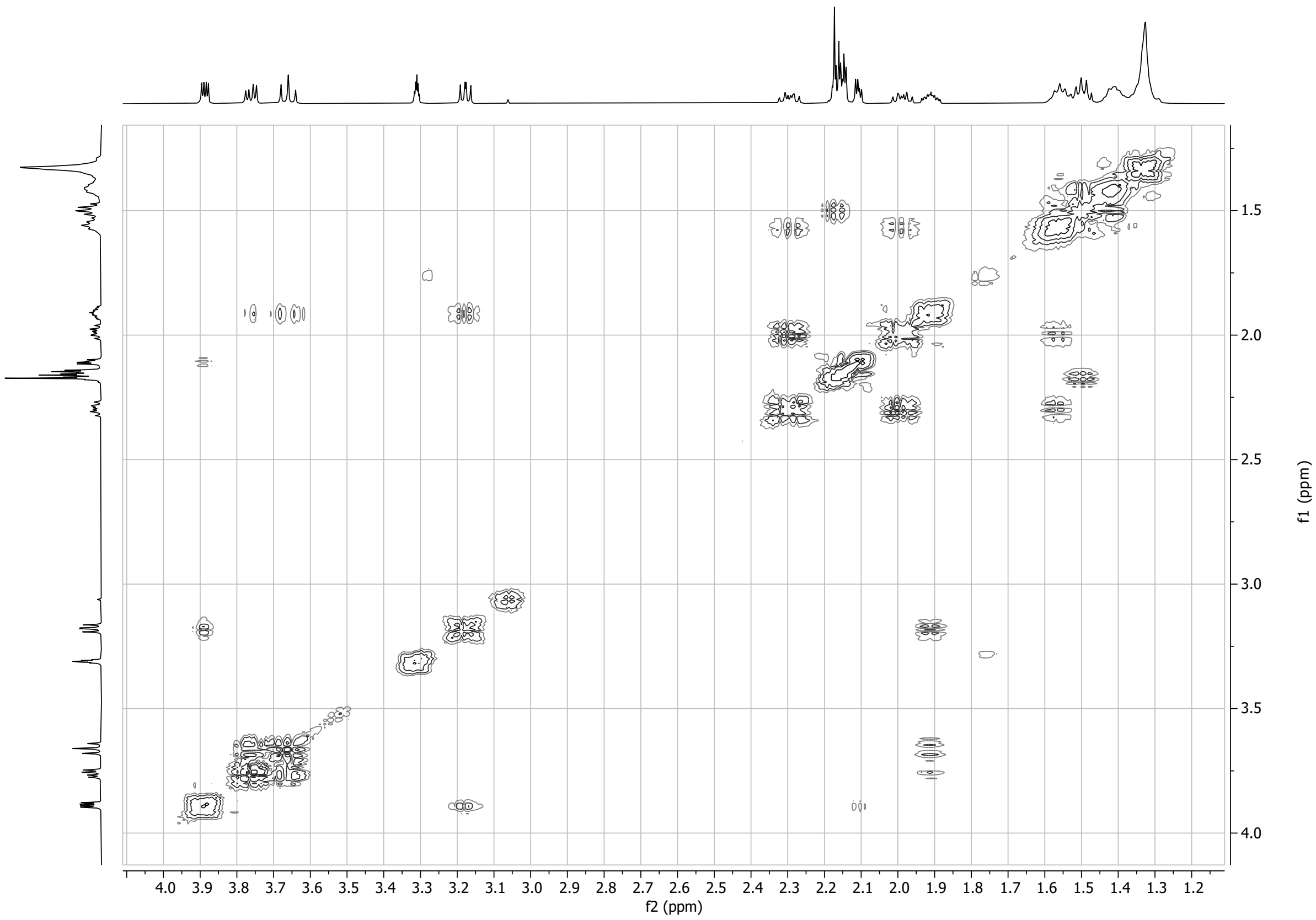

HSQC-NMR spectrum of compound 5 (Batch 2)

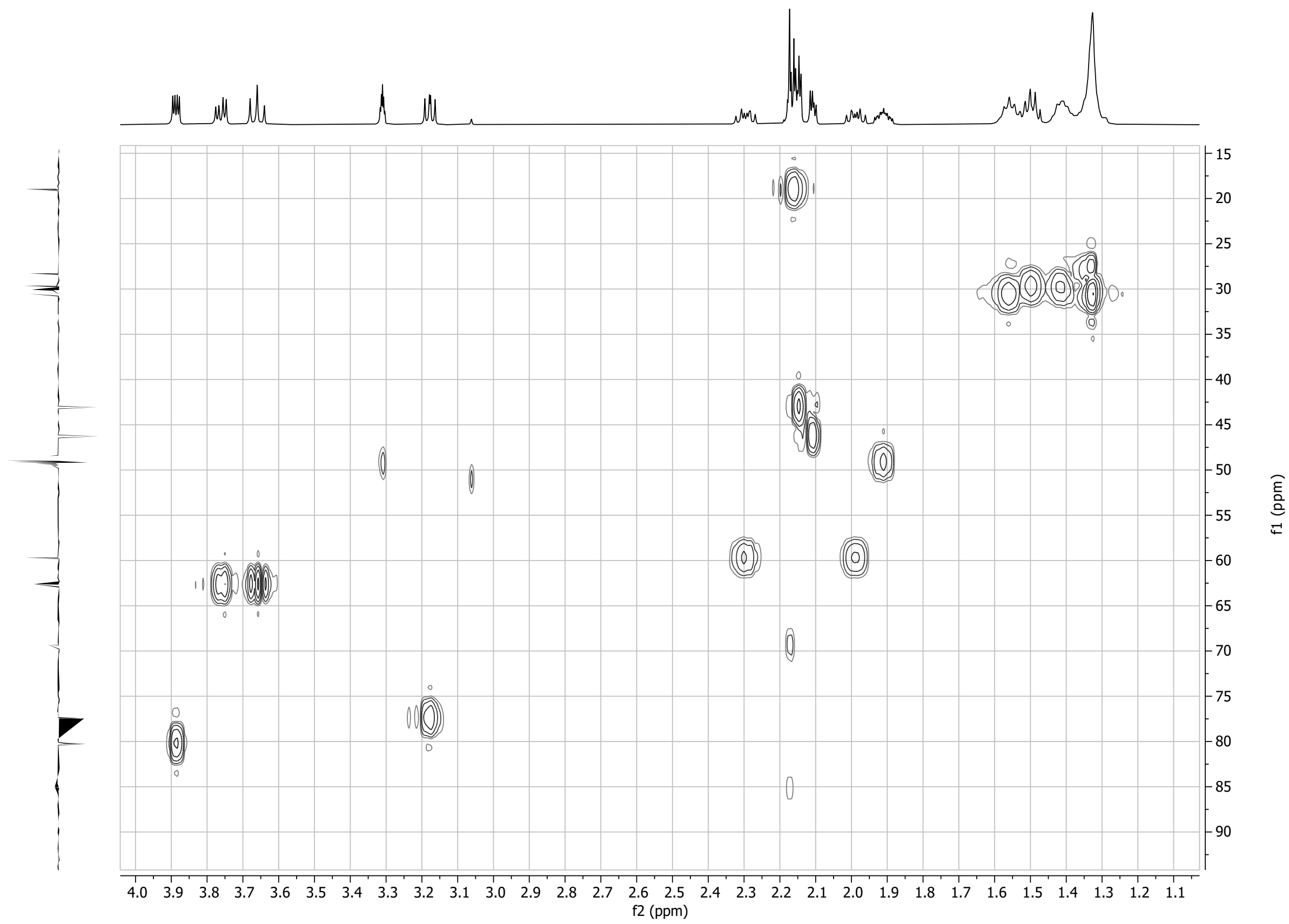

1H-NMR spectrum of compound 6

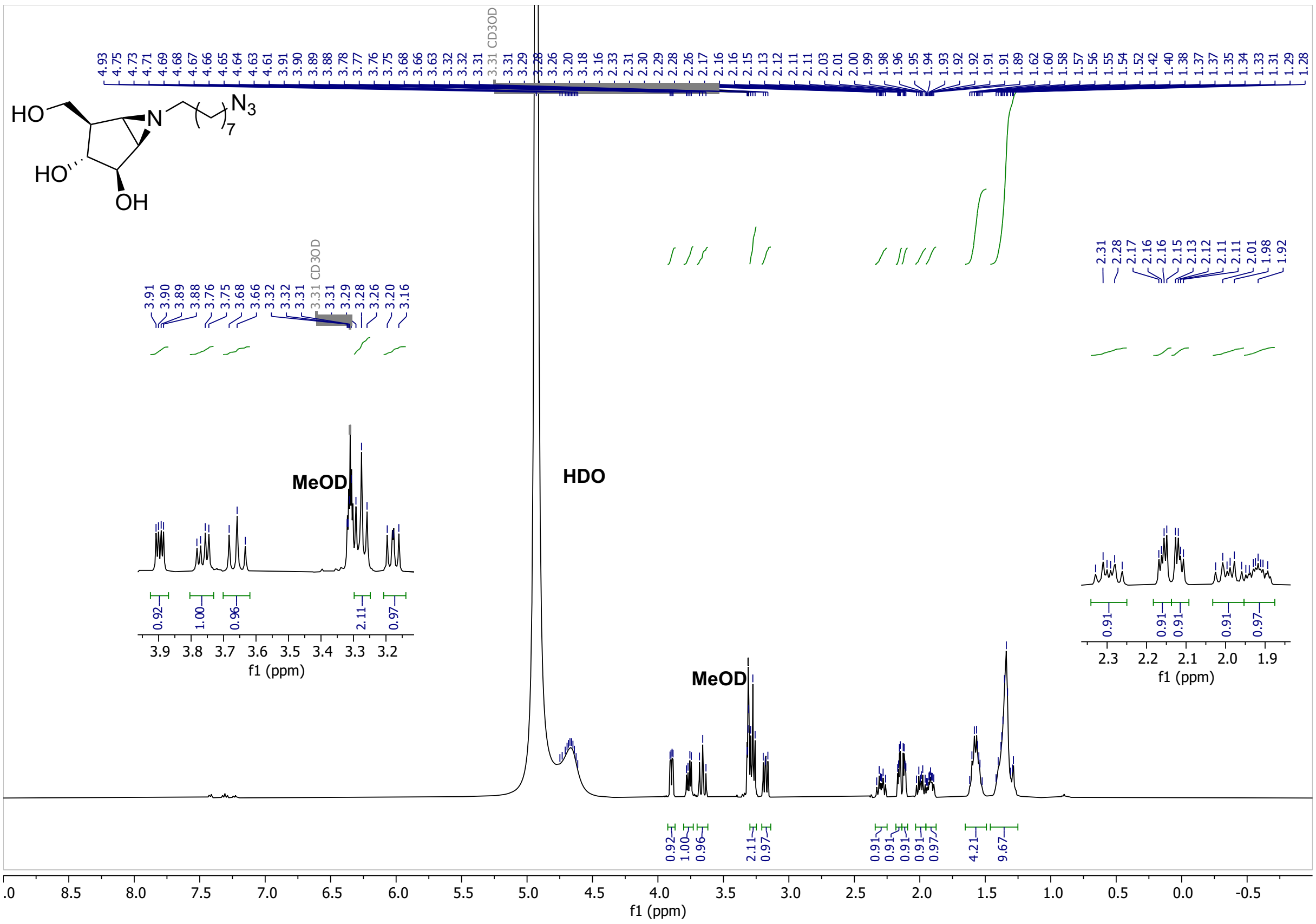

APT-NMR spectrum of compound 6

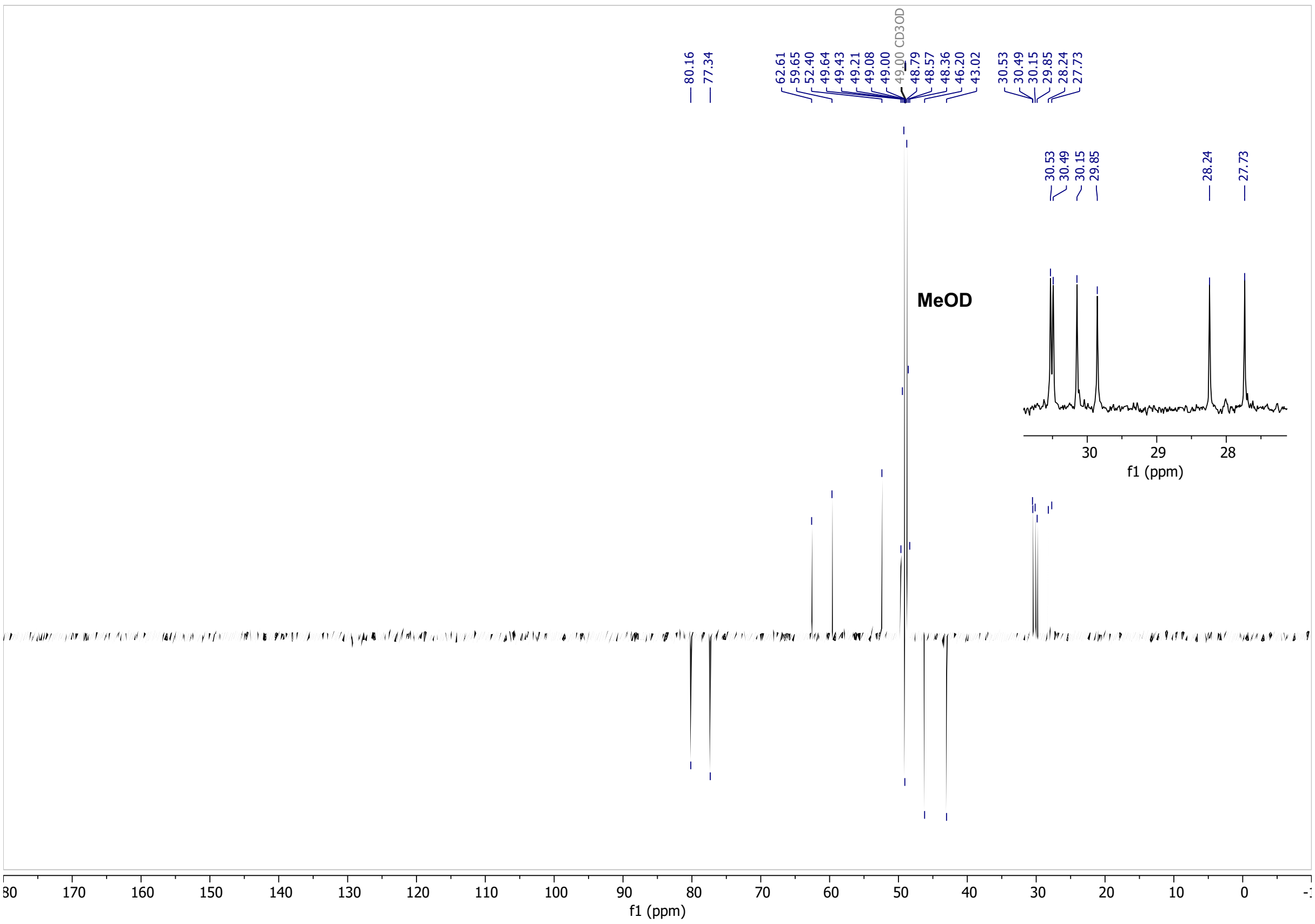

COSY-NMR spectrum of compound 6

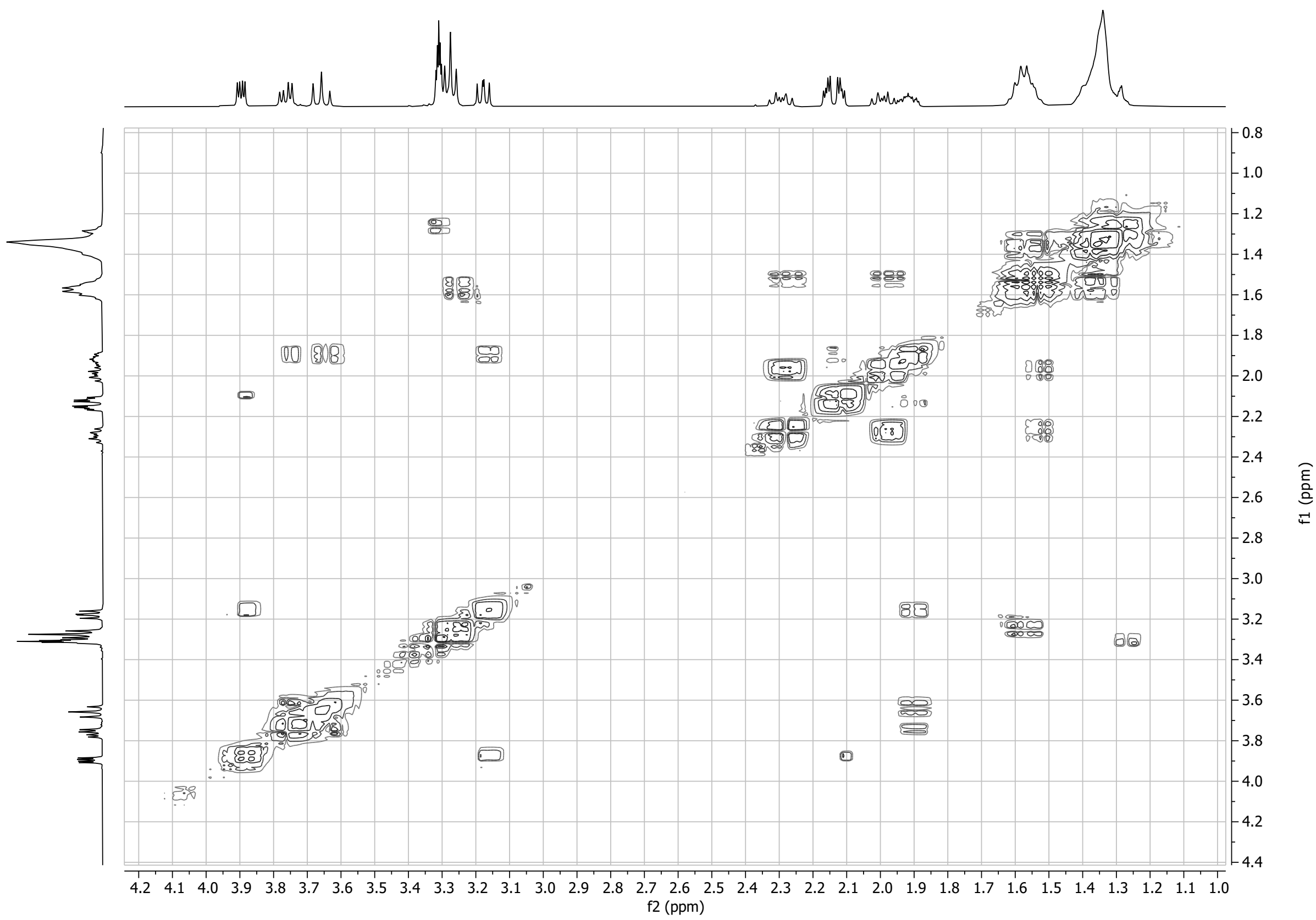

HSQC-NMR spectrum of compound 6

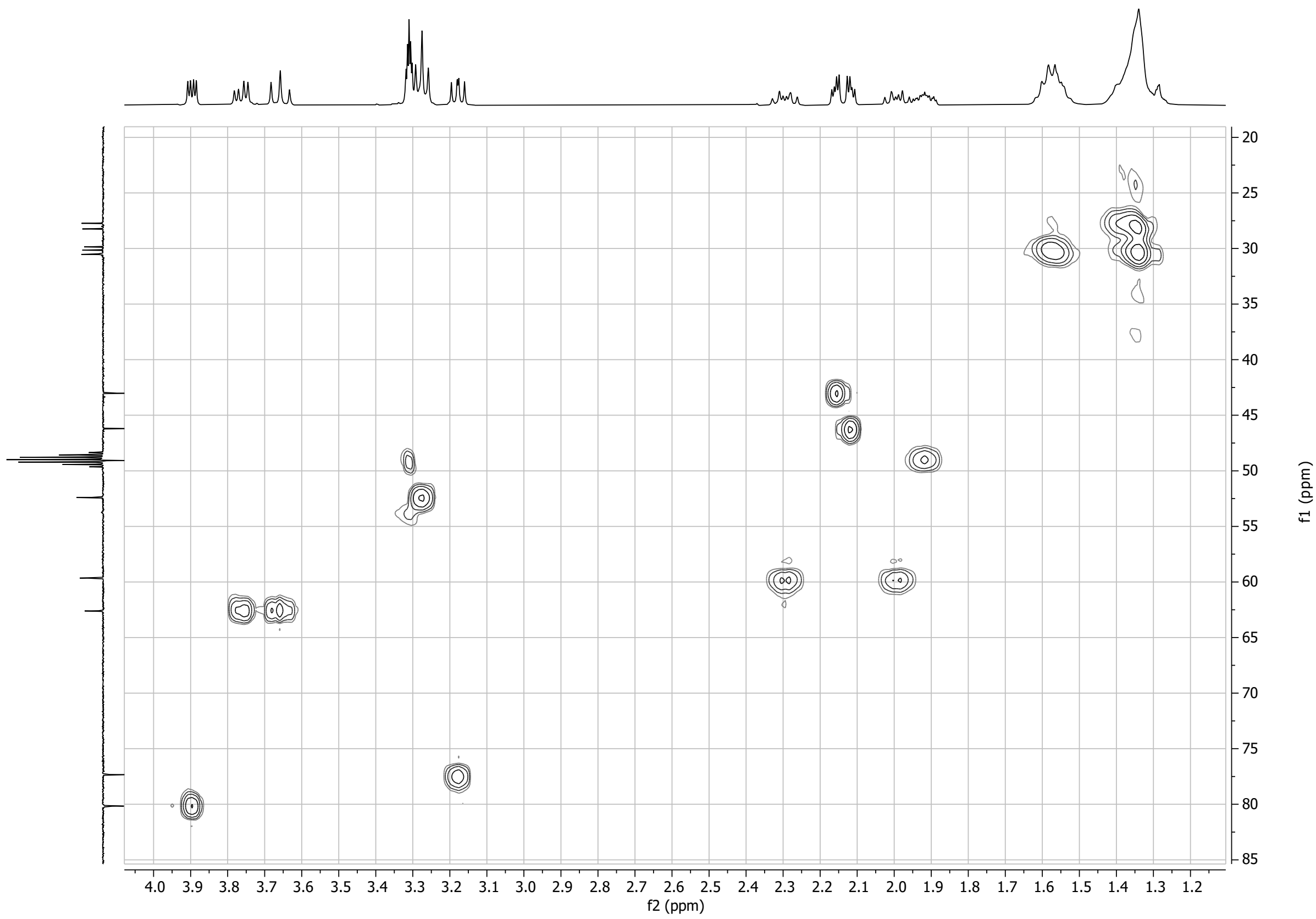

^{1}H-NMR spectrum of compound 29

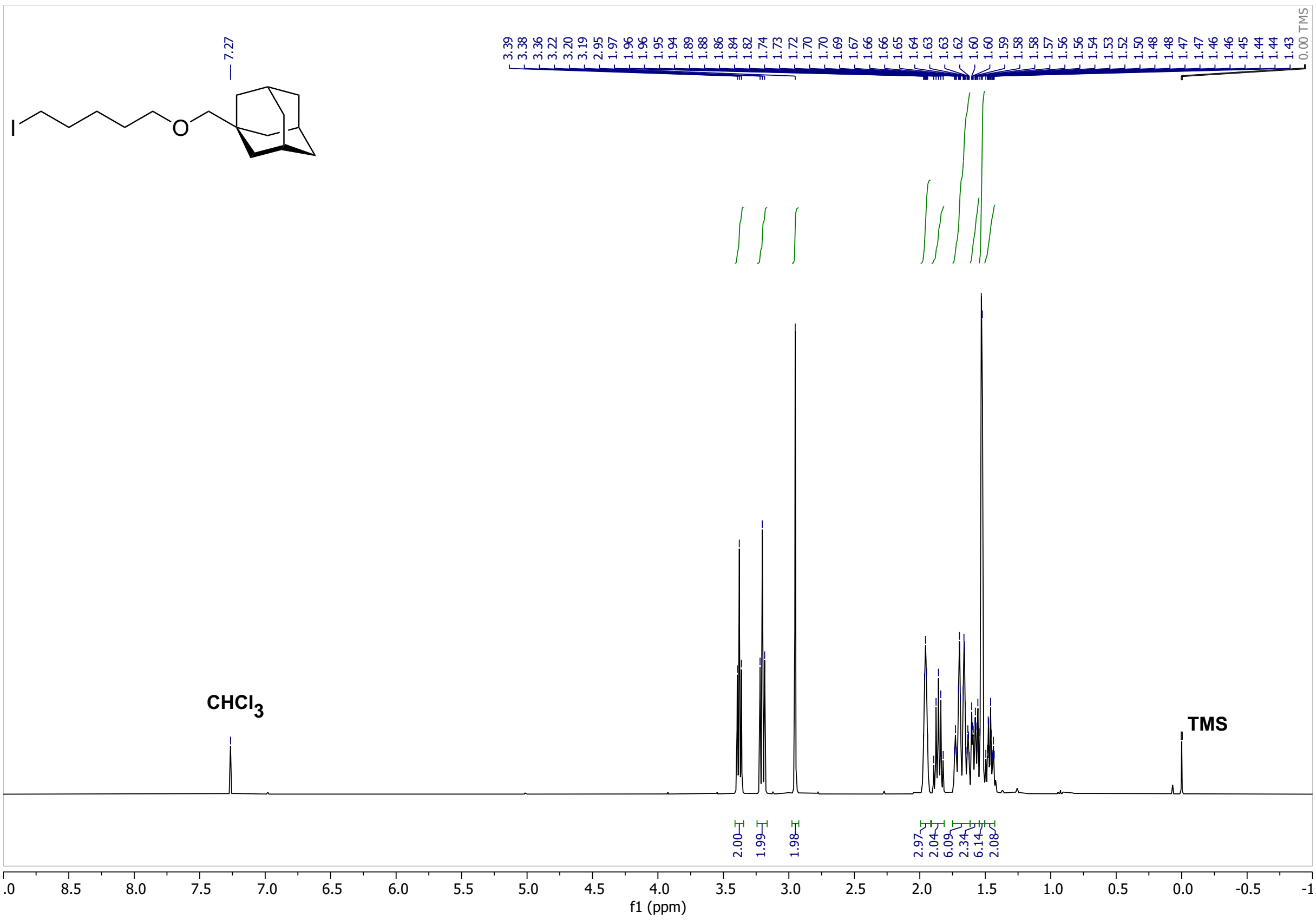

APT-NMR spectrum of compound 29

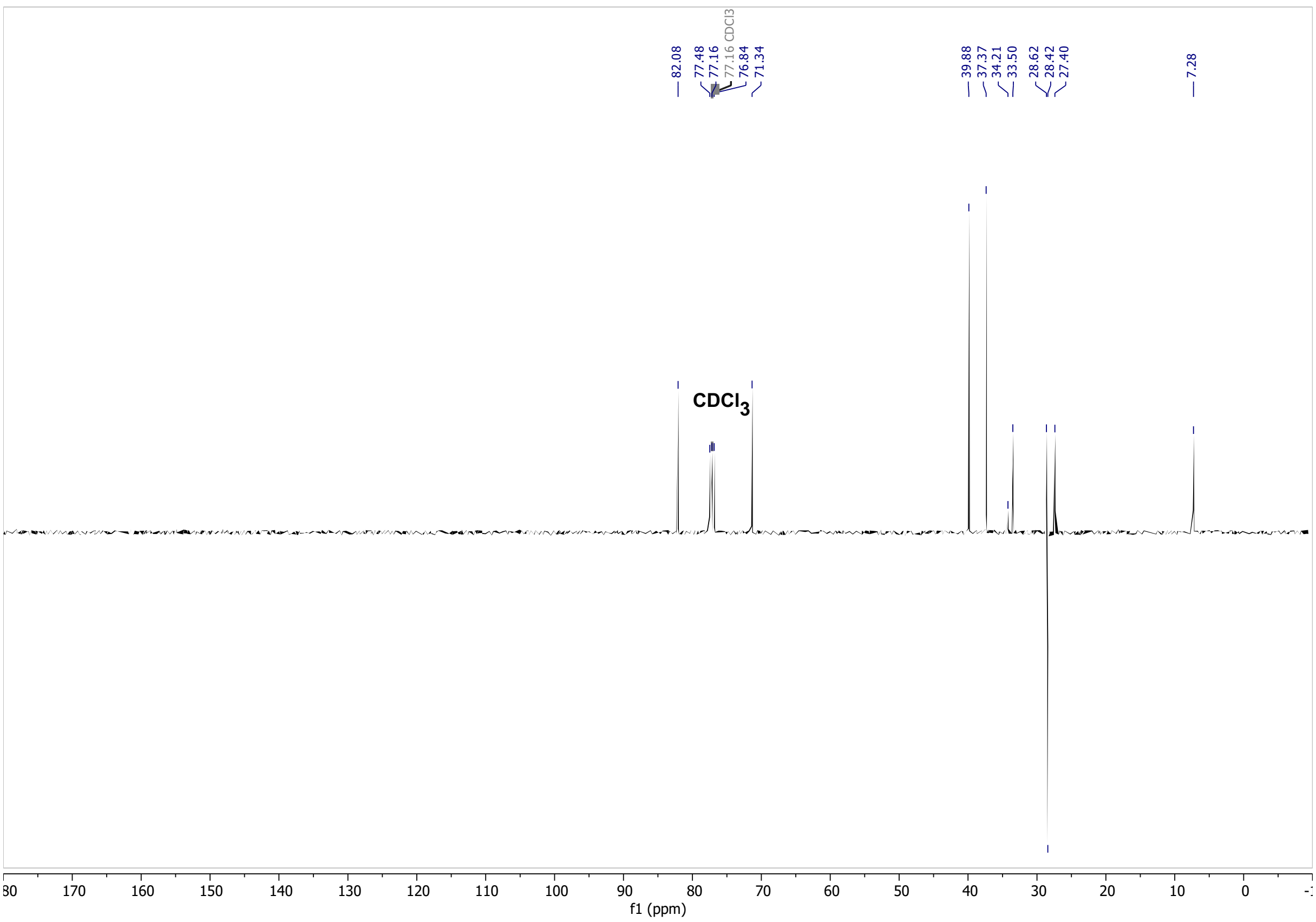

COSY-NMR spectrum of compound 29

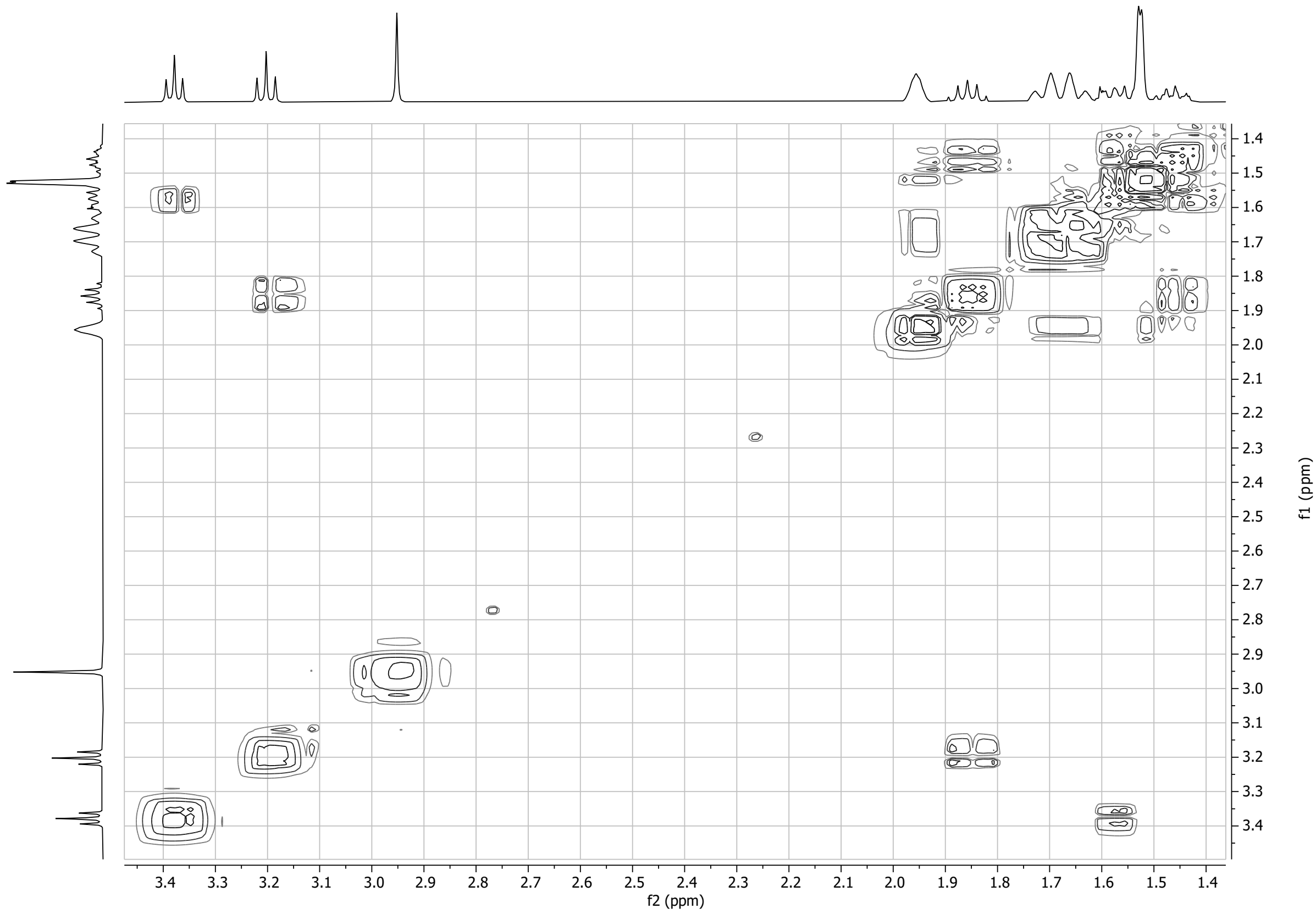

HSQC-NMR spectrum of compound 29

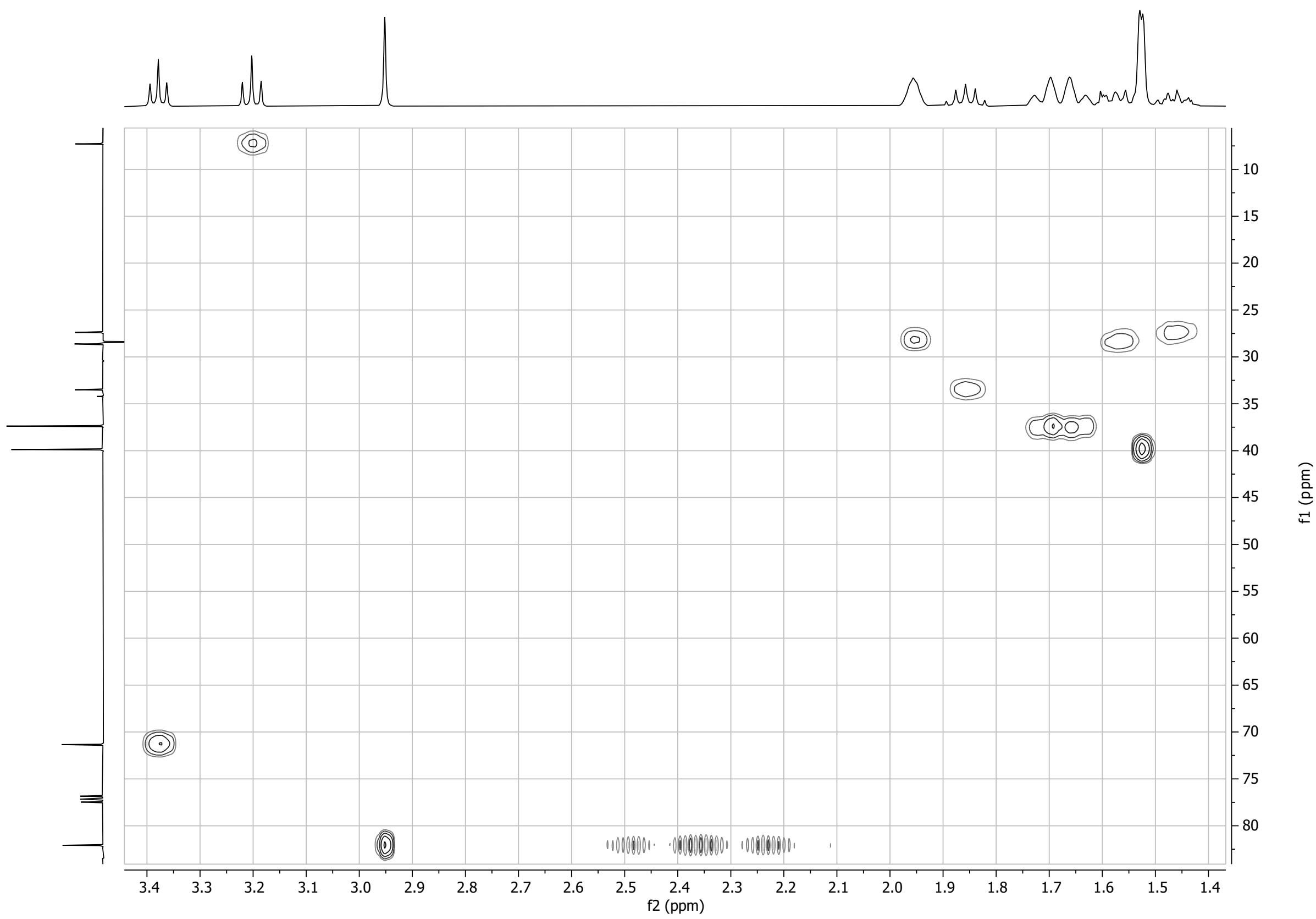

¹H-NMR spectrum of compound 7

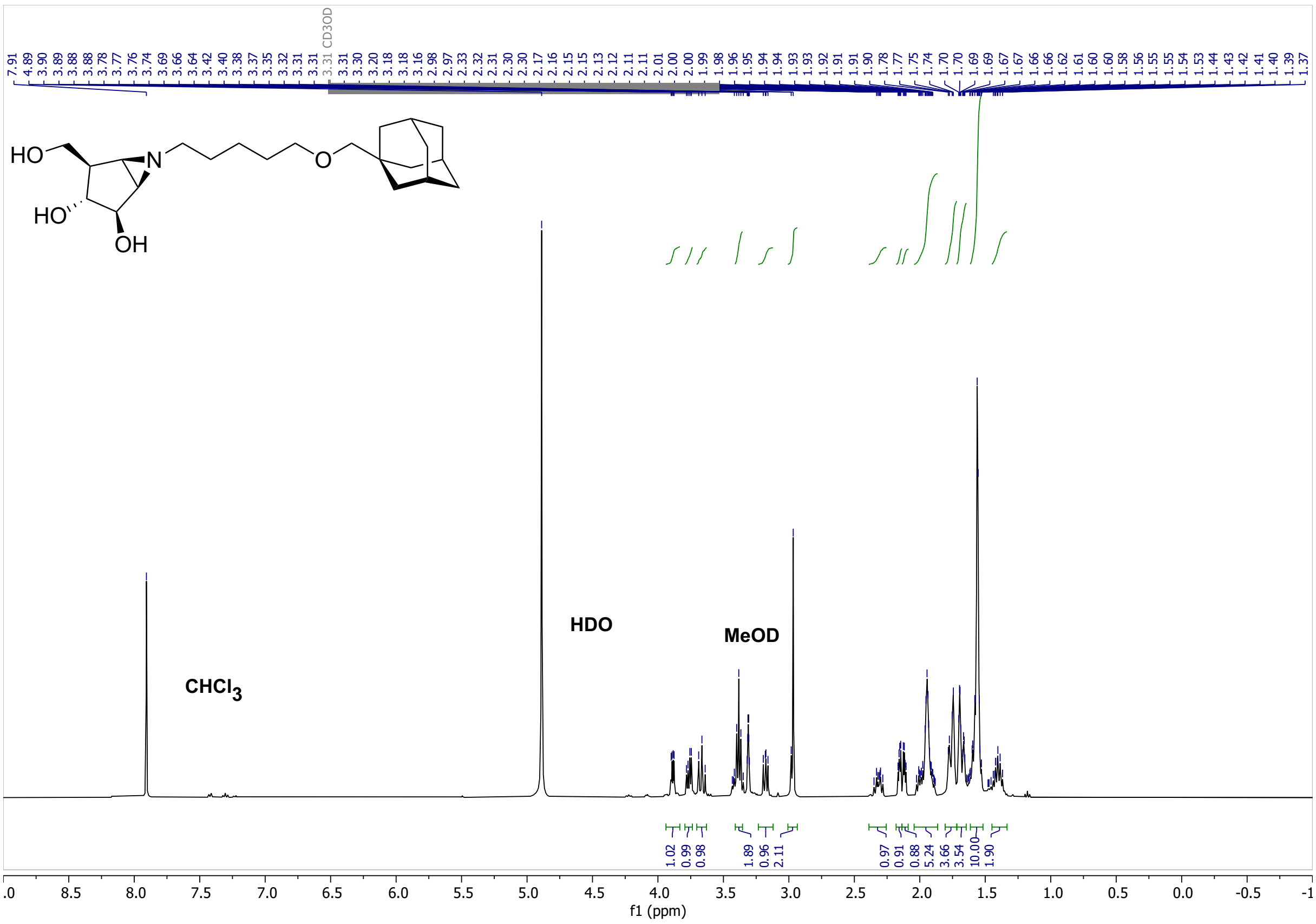

APT-NMR spectrum of compound 7

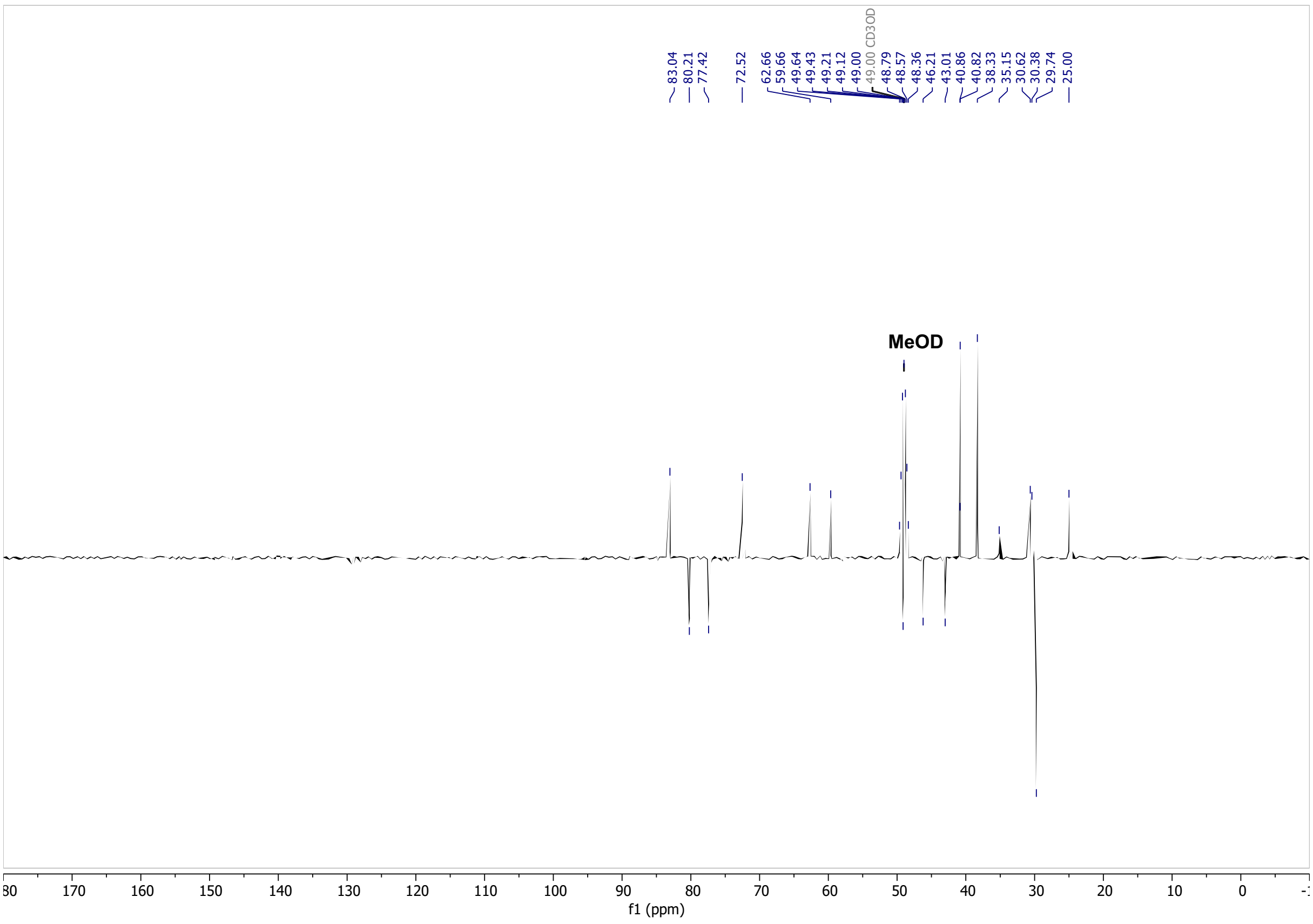

COSY-NMR spectrum of compound 7

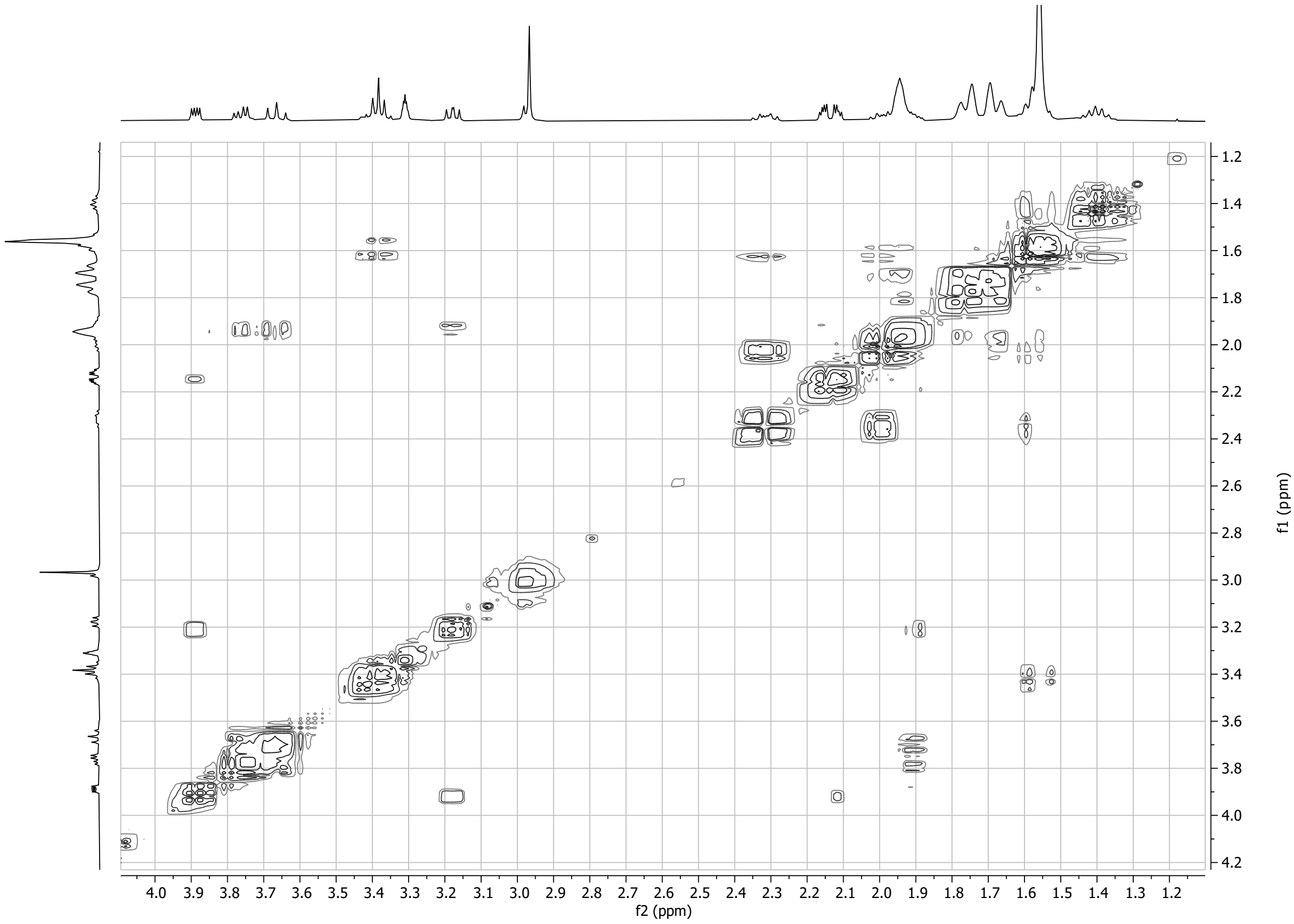

HSQC-NMR spectrum of compound 7

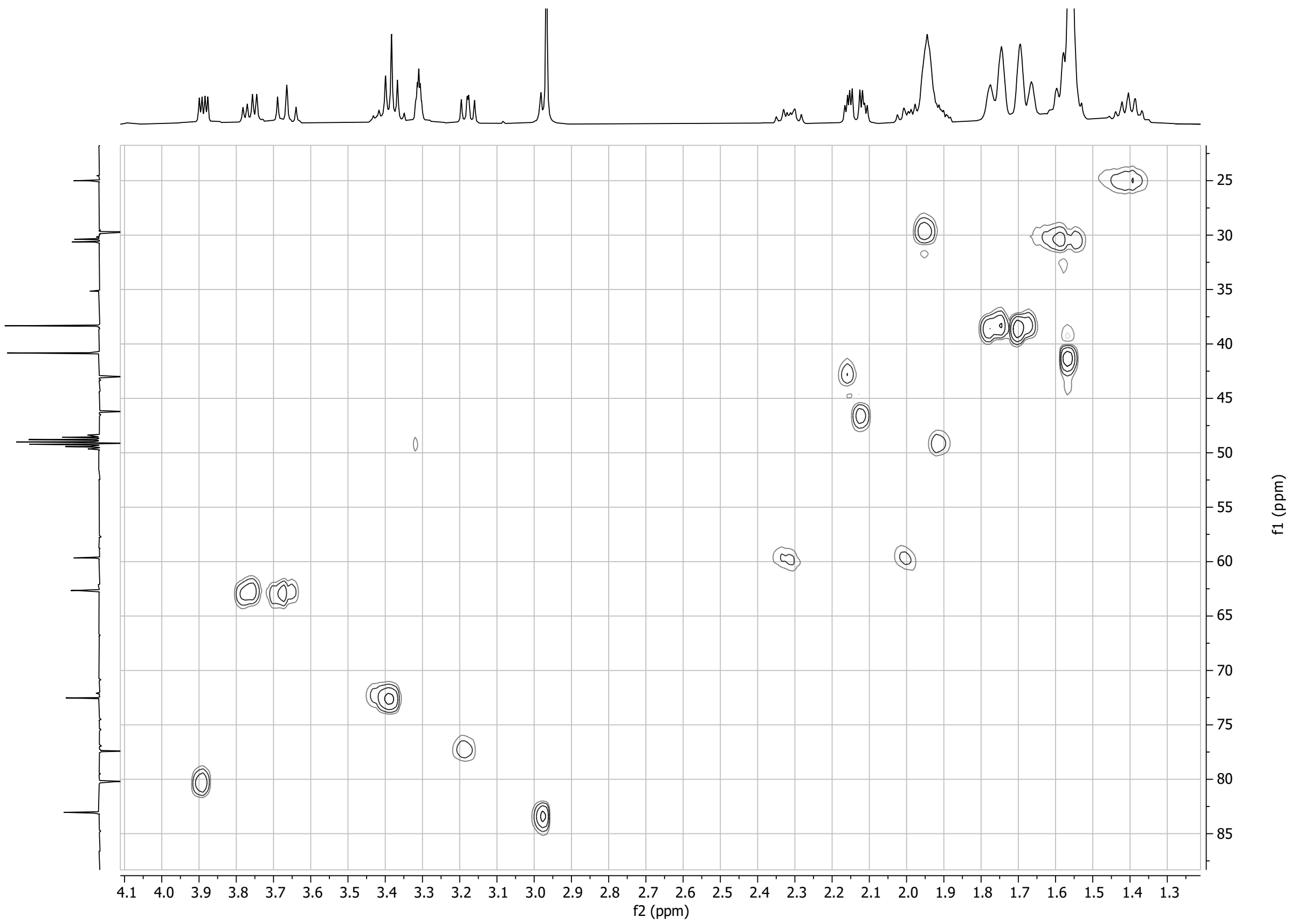

^{1}H-NMR spectrum of compound 30

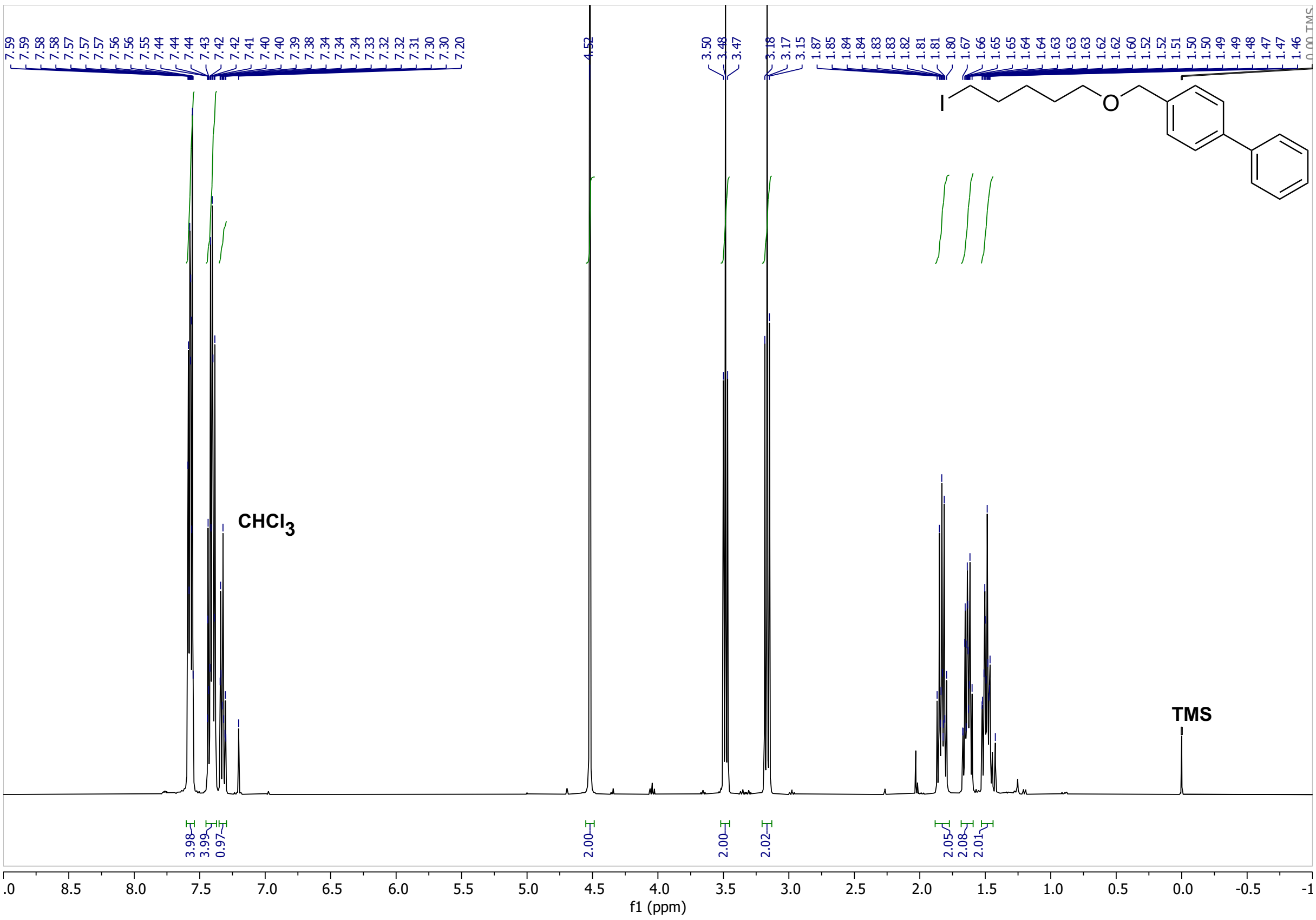

APT-NMR spectrum of compound 30

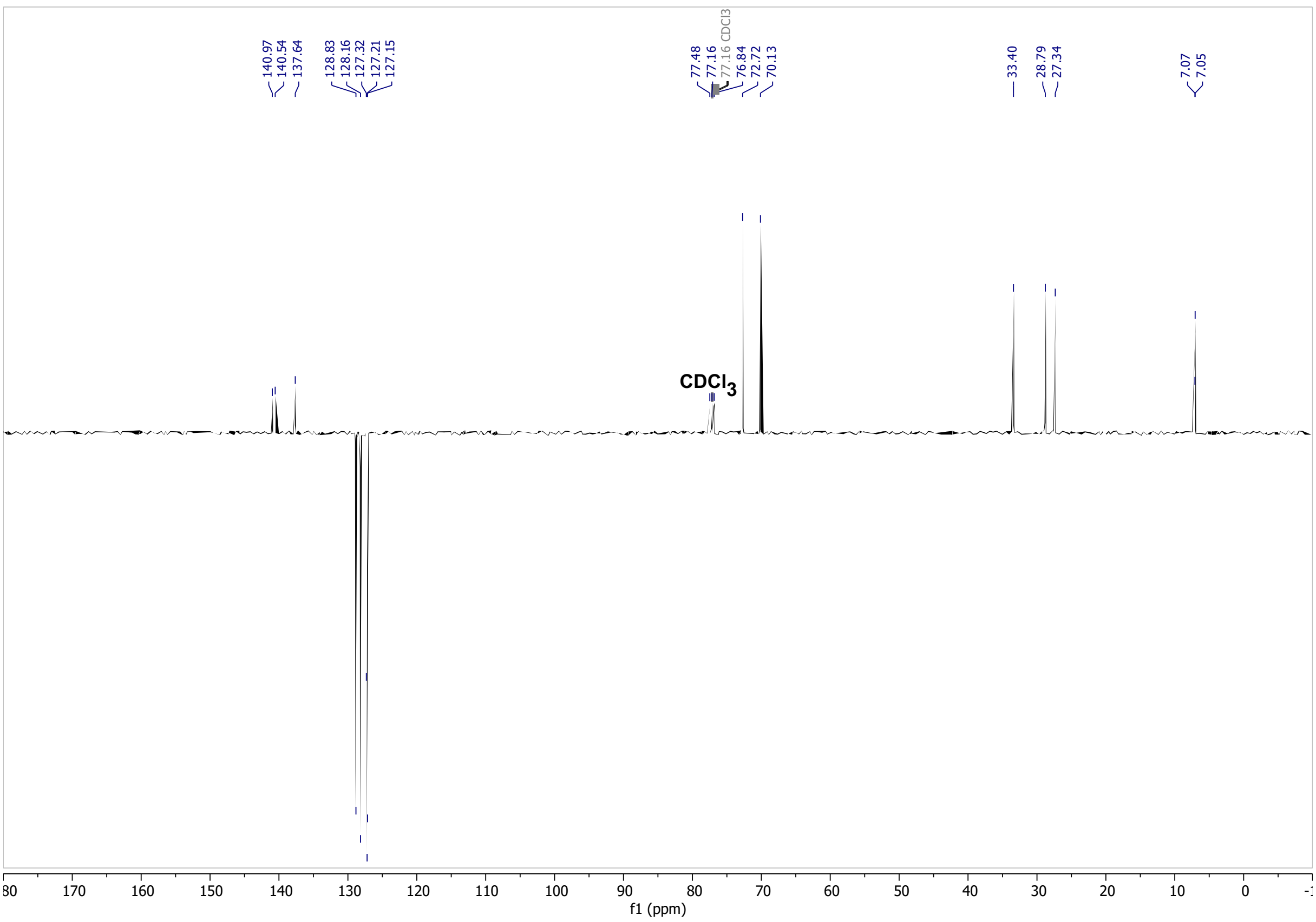

COSY-NMR spectrum of compound 30

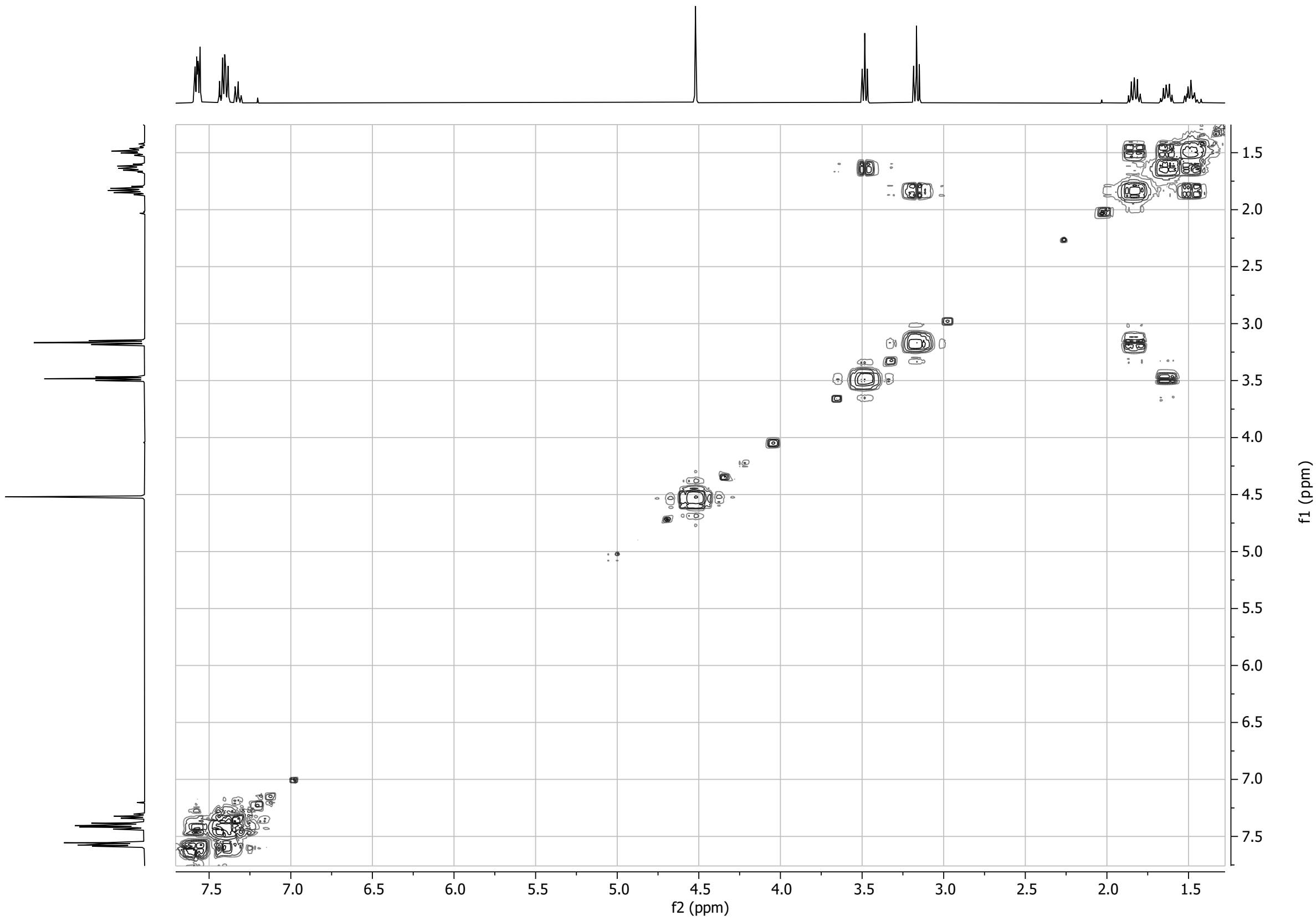

HSQC-NMR spectrum of compound 30

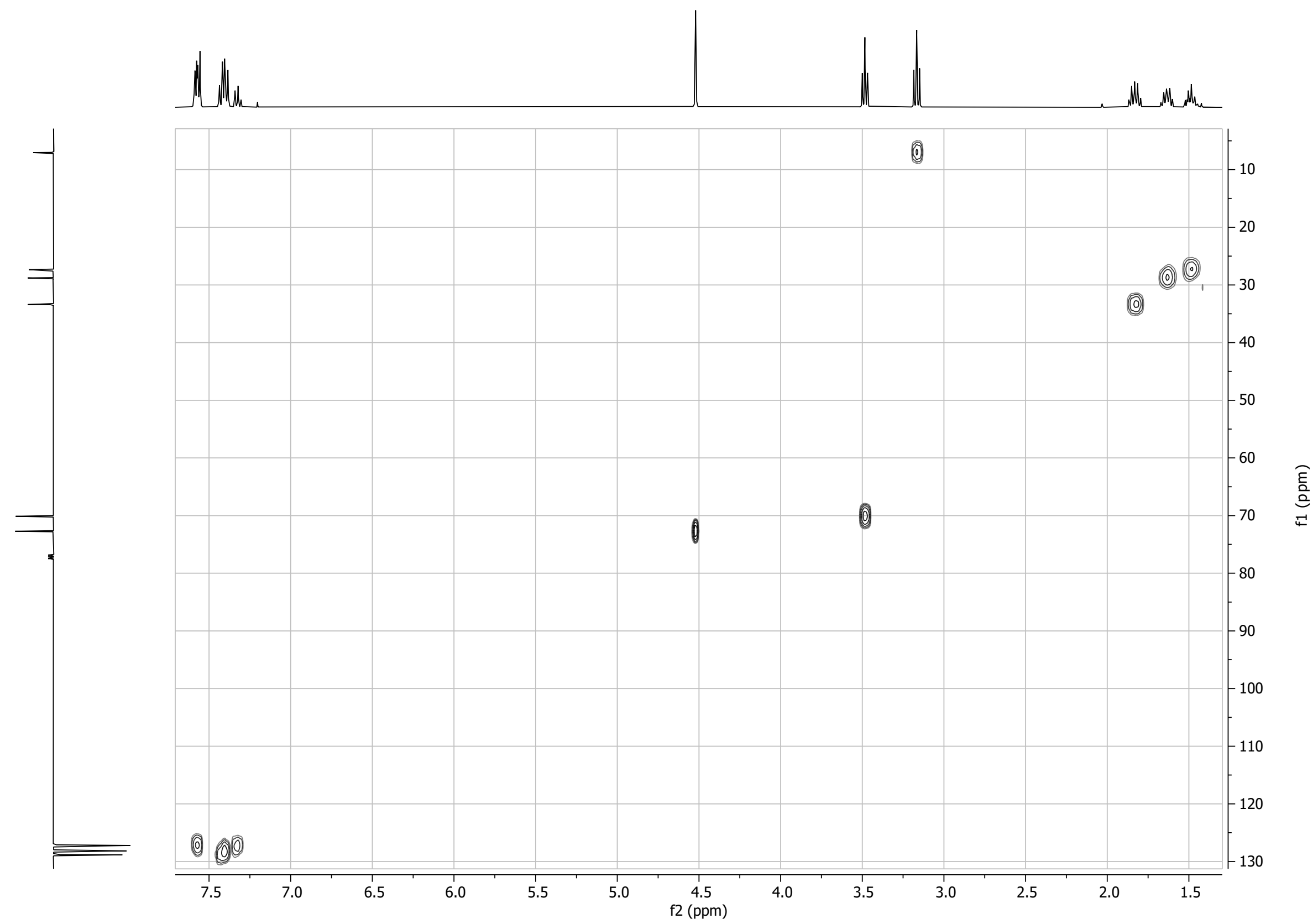

1H-NMR spectrum of compound 8

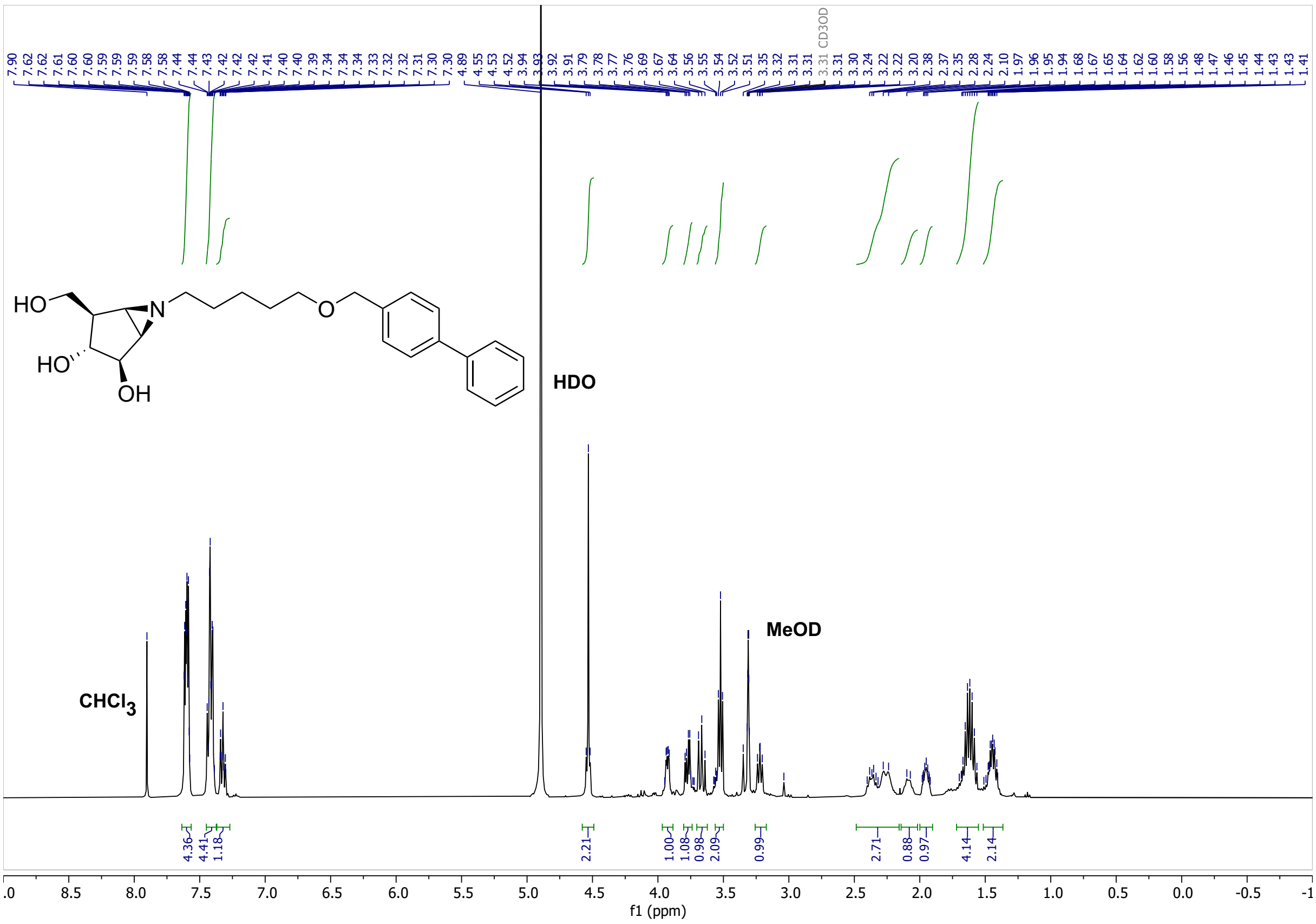

APT-NMR spectrum of compound 8

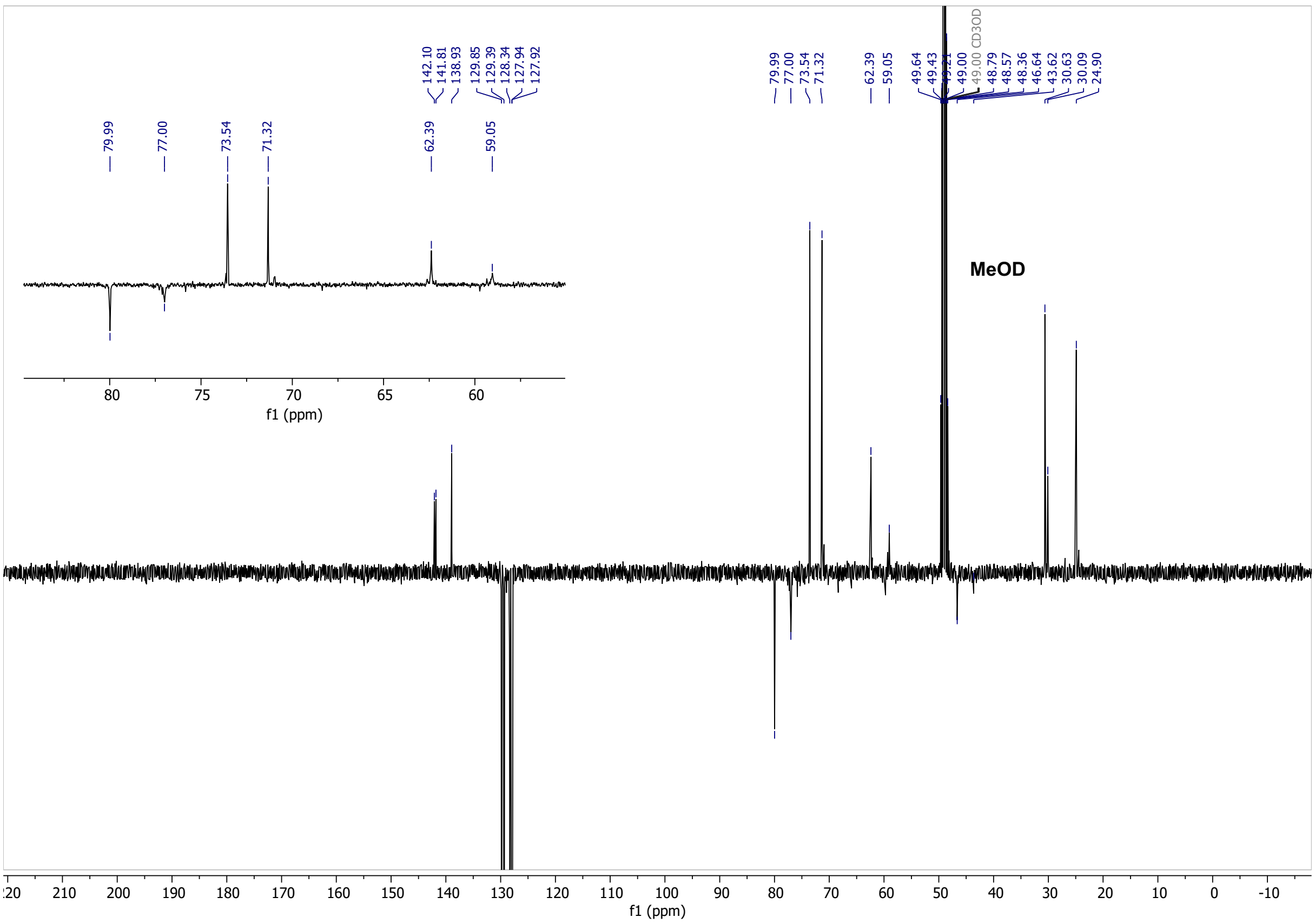

COSY-NMR spectrum of compound 8

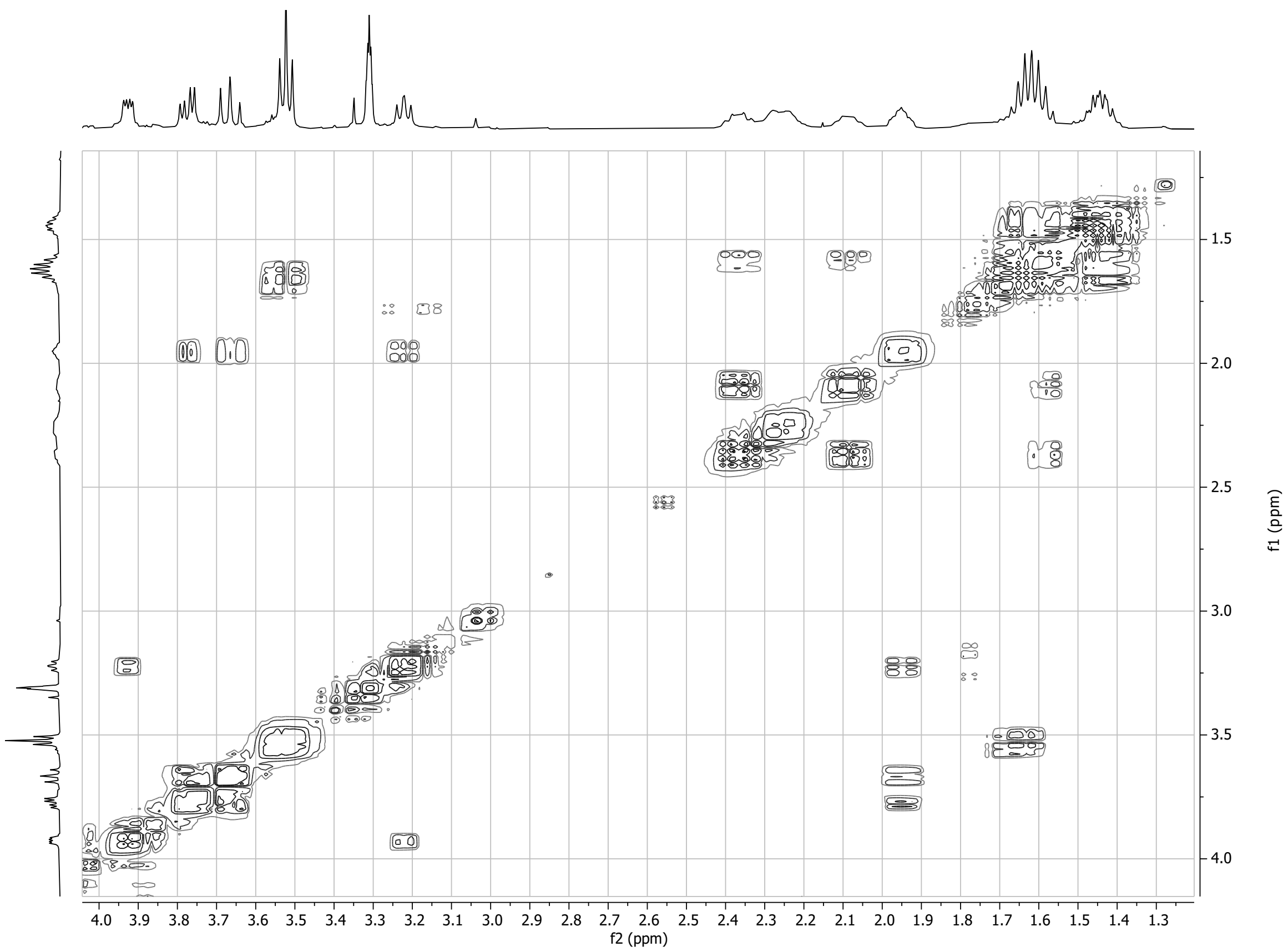

HSQC-NMR spectrum of compound 8

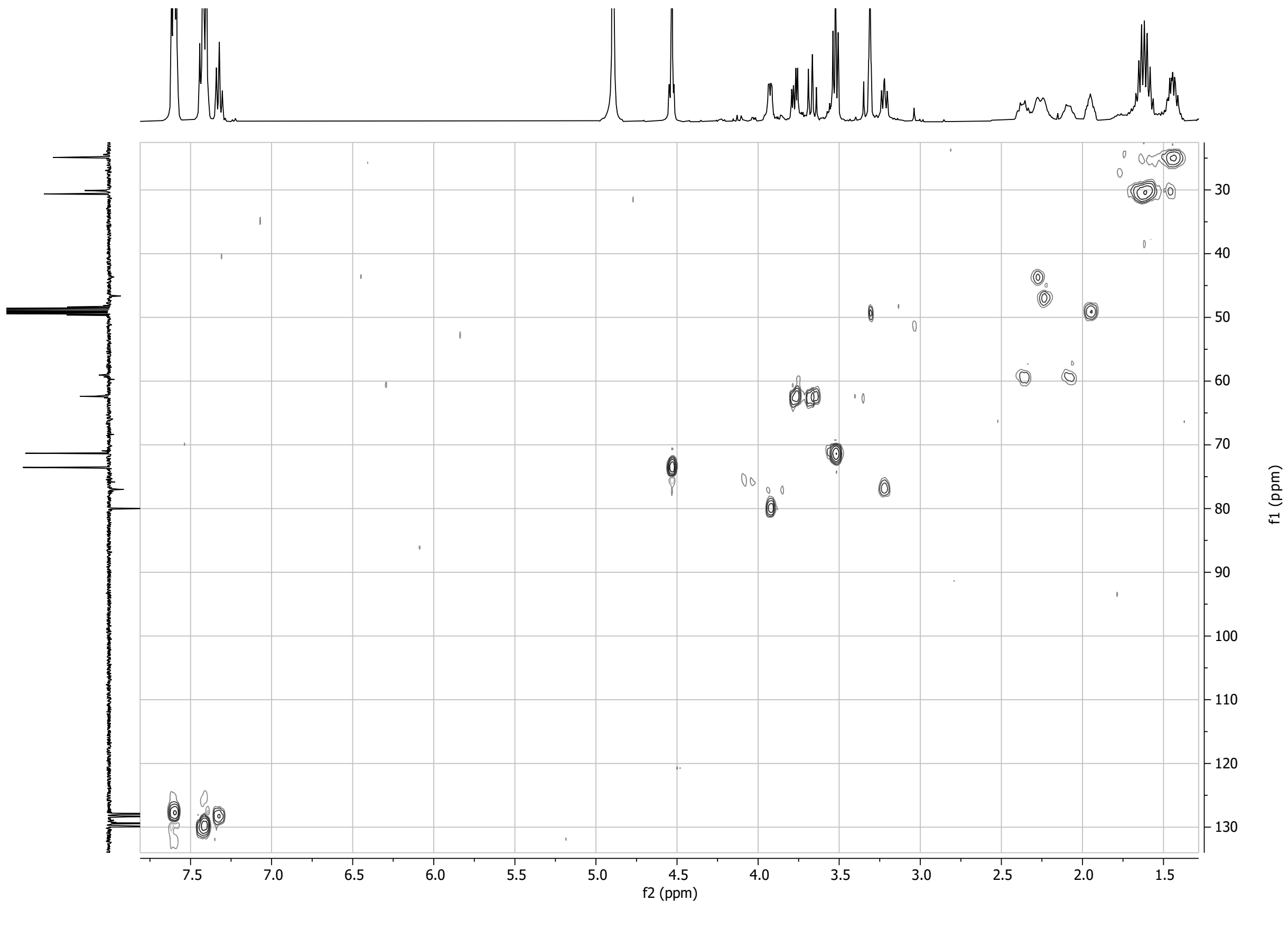

¹H-NMR spectrum of compound 9

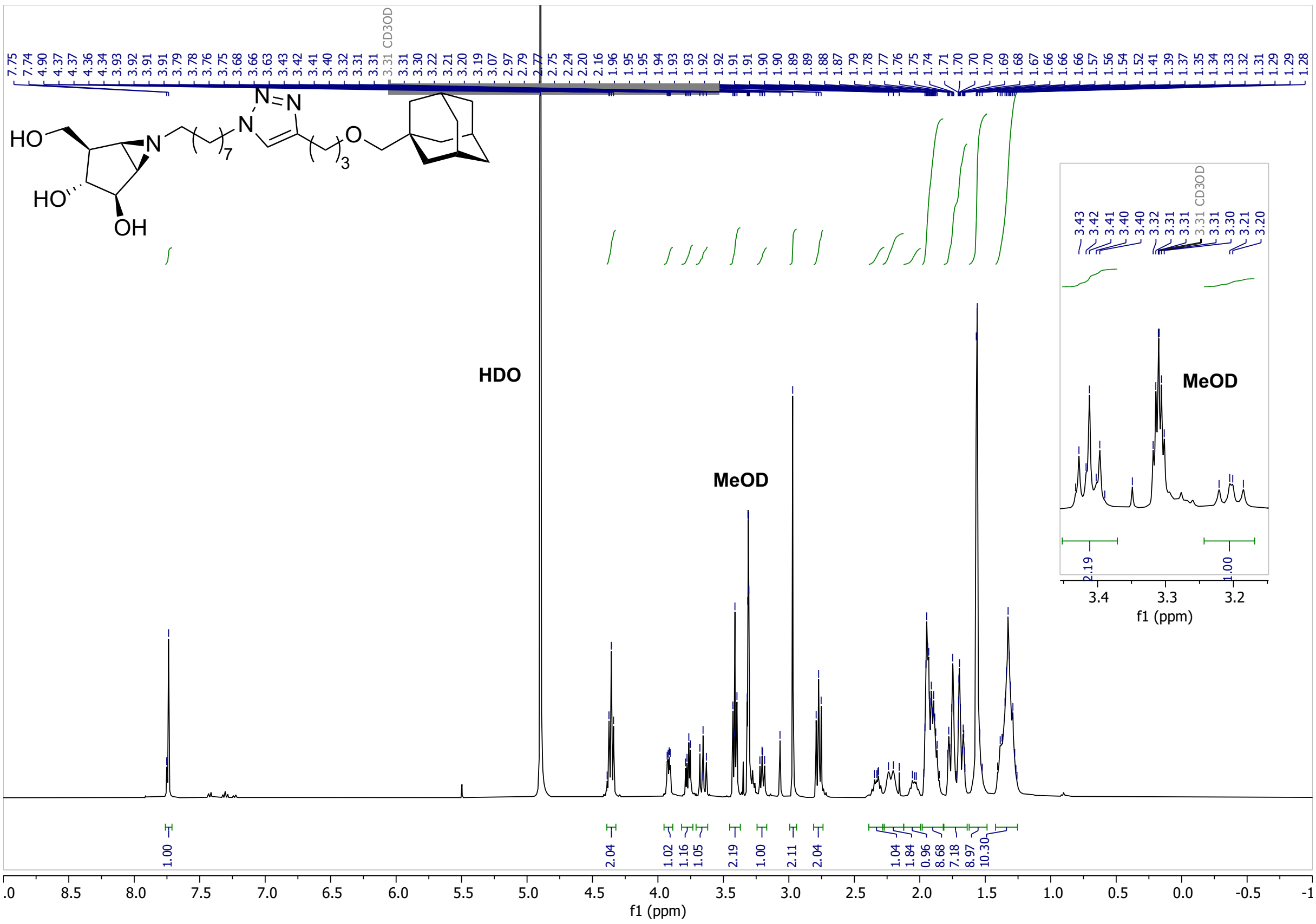

APT-NMR spectrum of compound 9

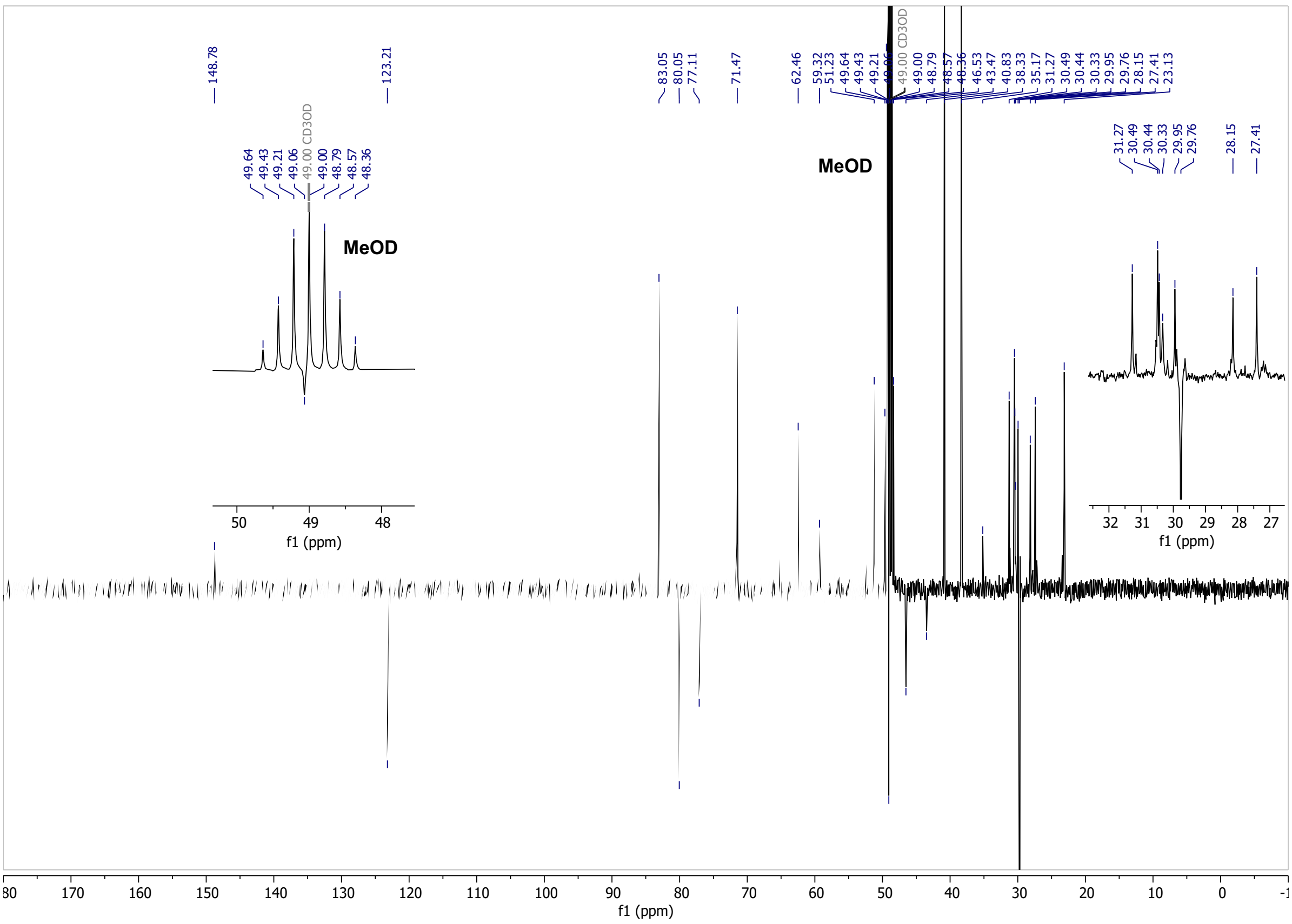

COSY-NMR spectrum of compound 9

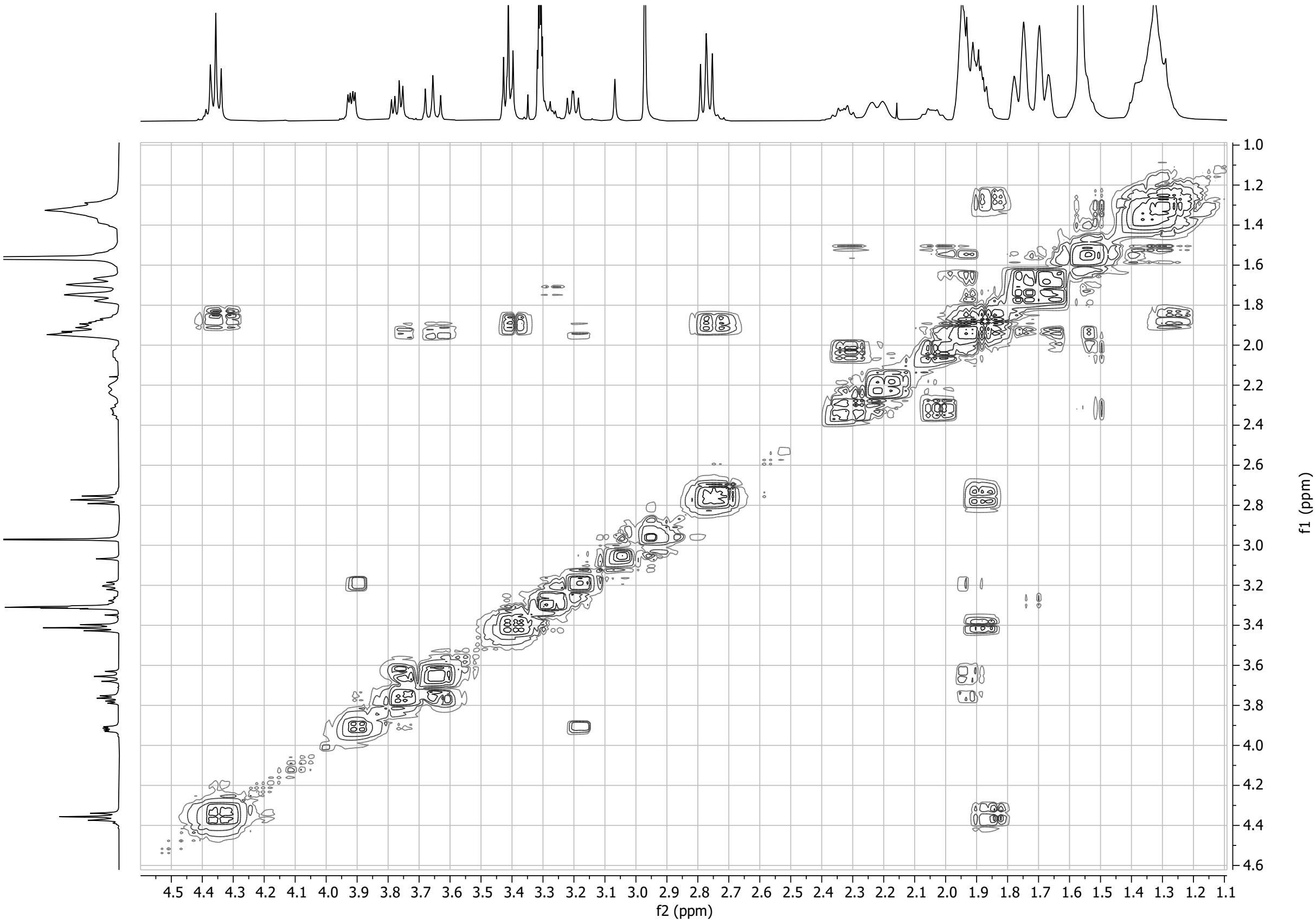

HSQC-NMR spectrum of compound 9

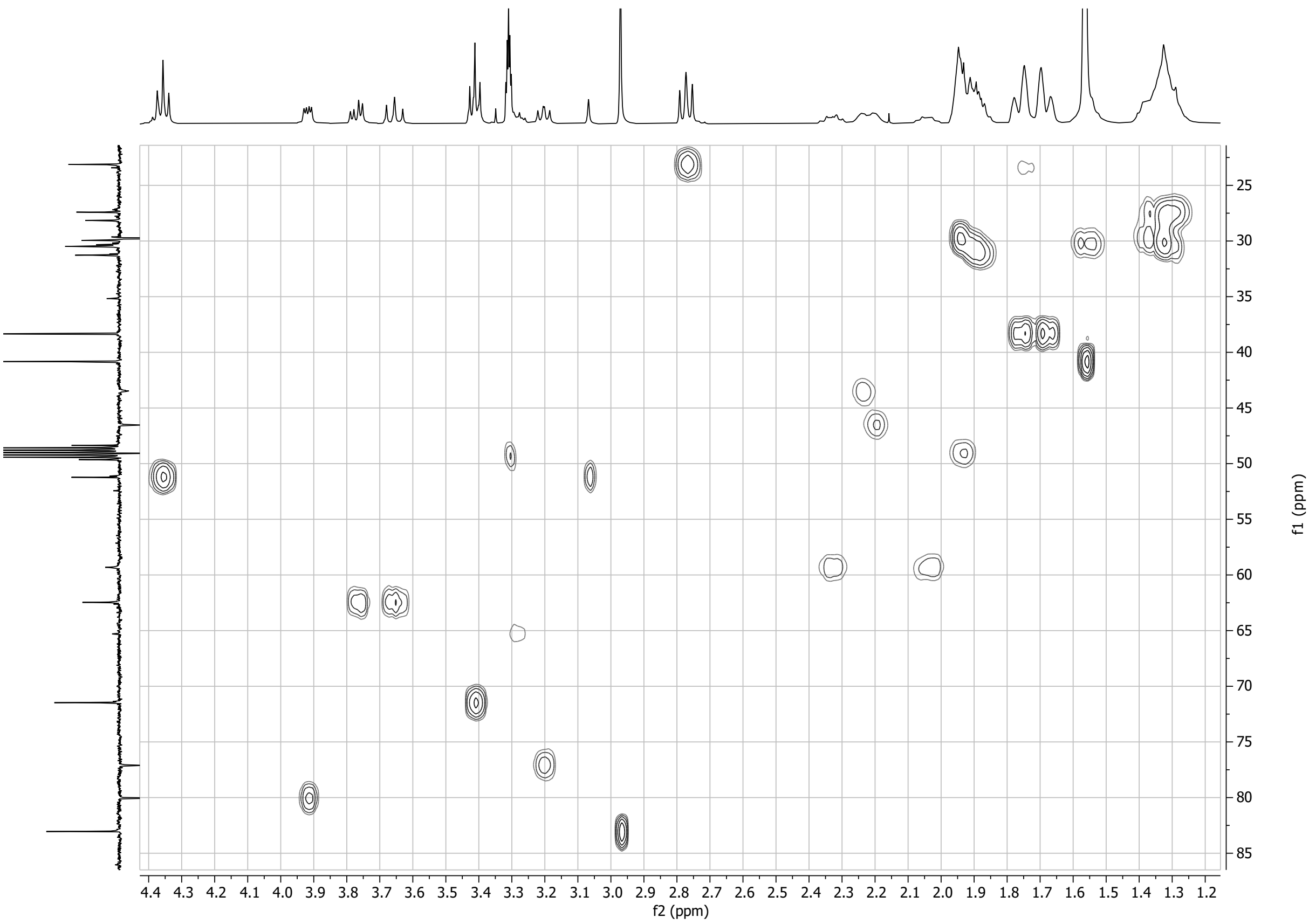

¹H-NMR spectrum of compound 31

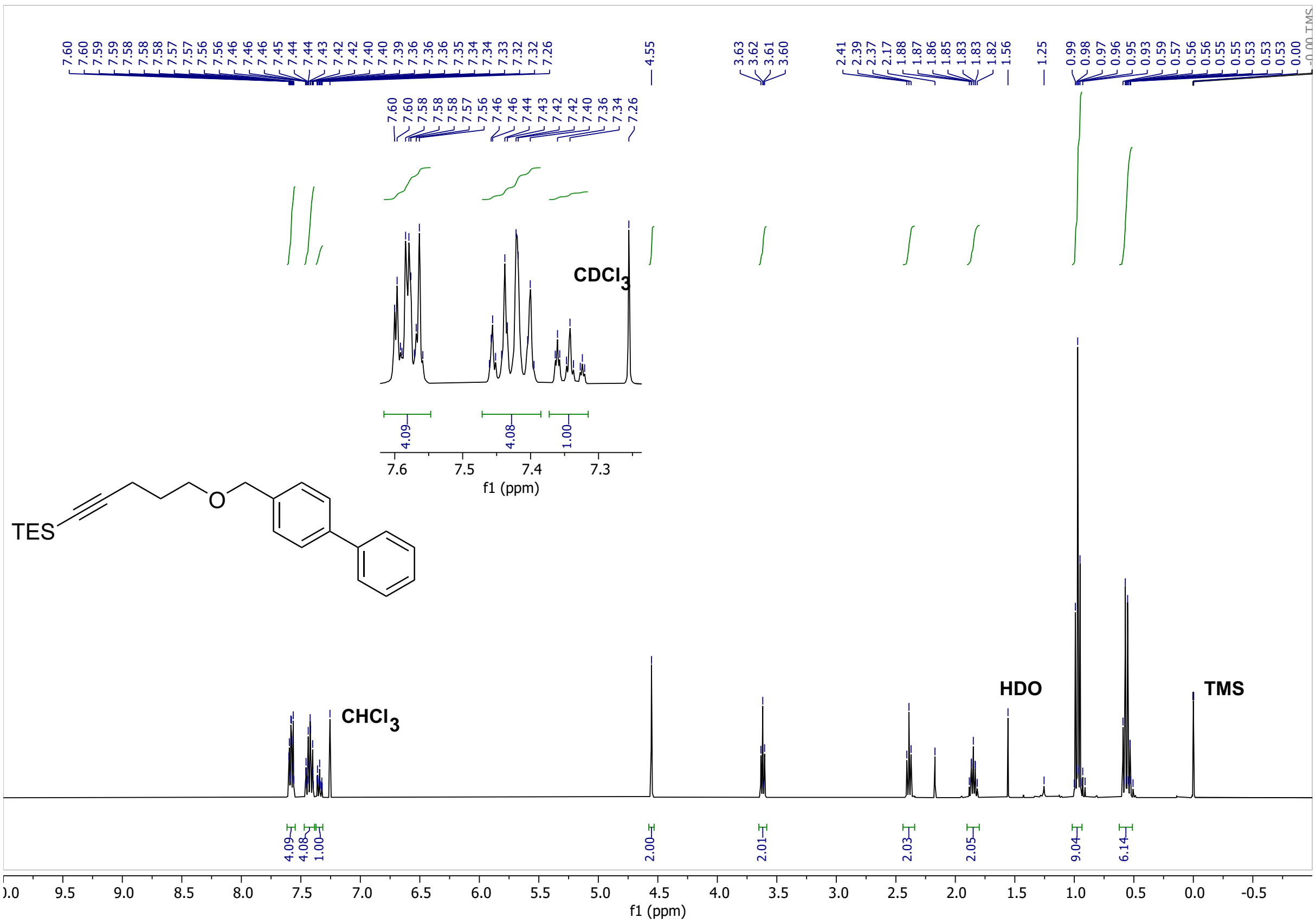

APT-NMR spectrum of compound 31

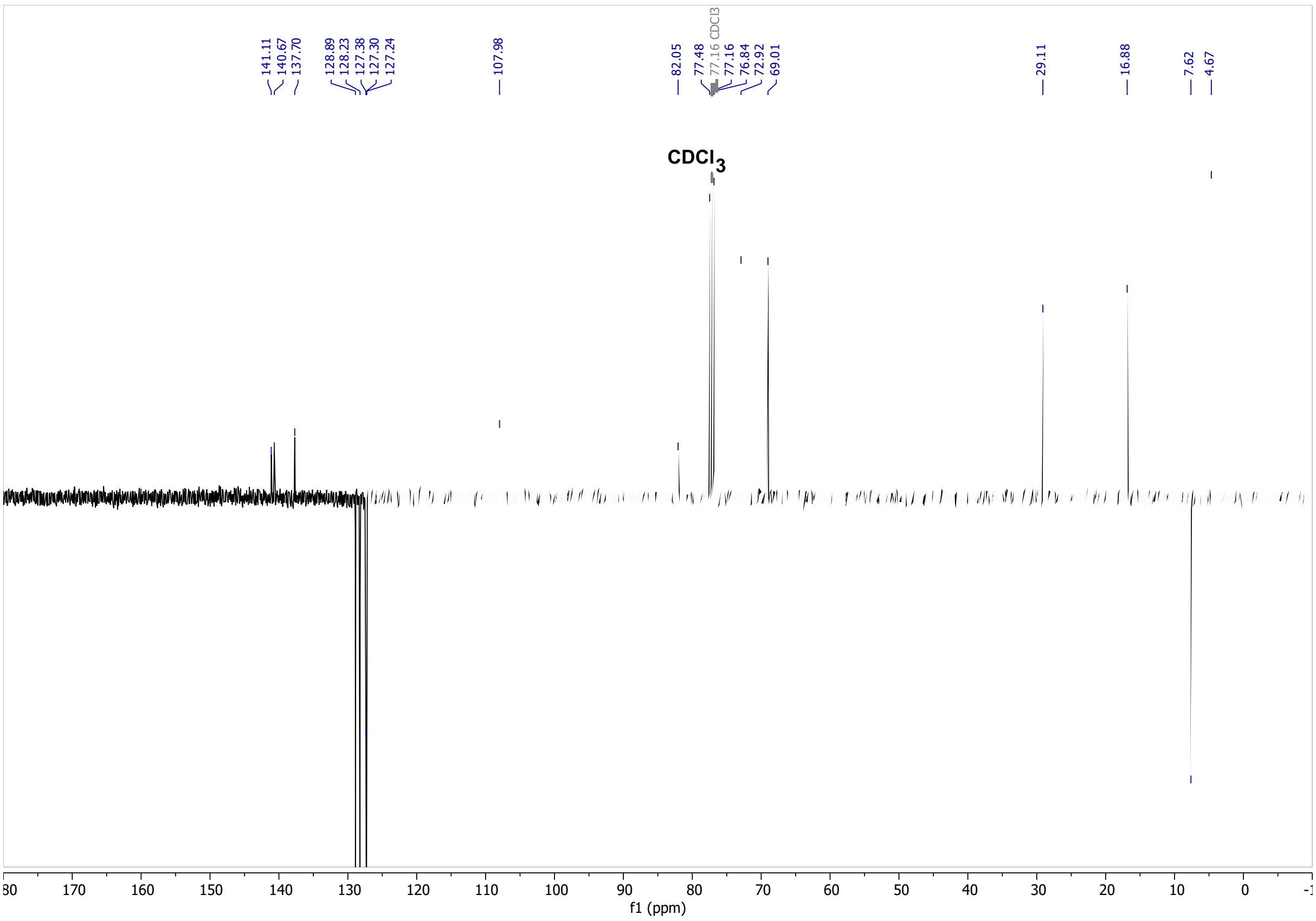

COSY-NMR spectrum of compound 31

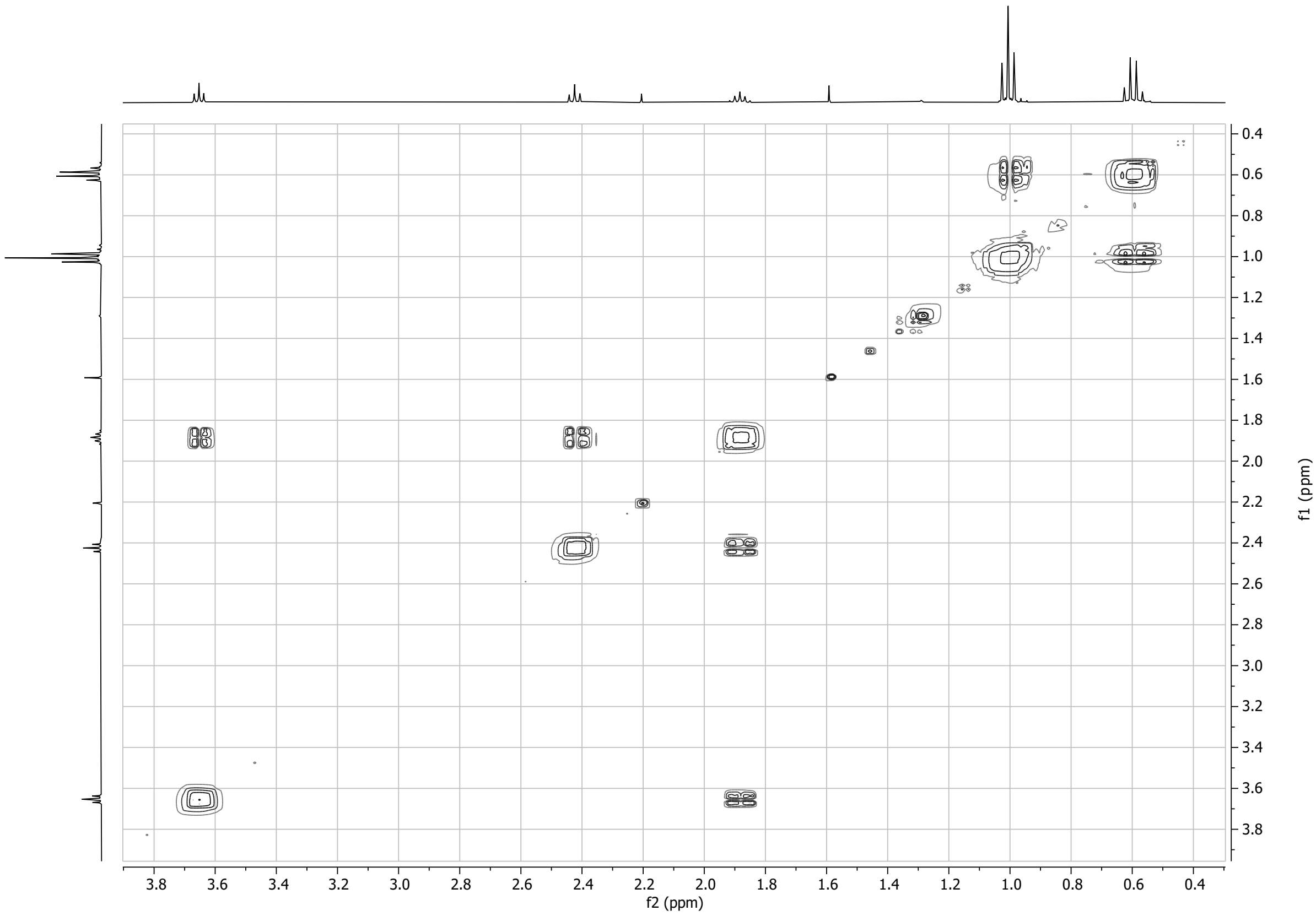

HSQC-NMR spectrum of compound 31

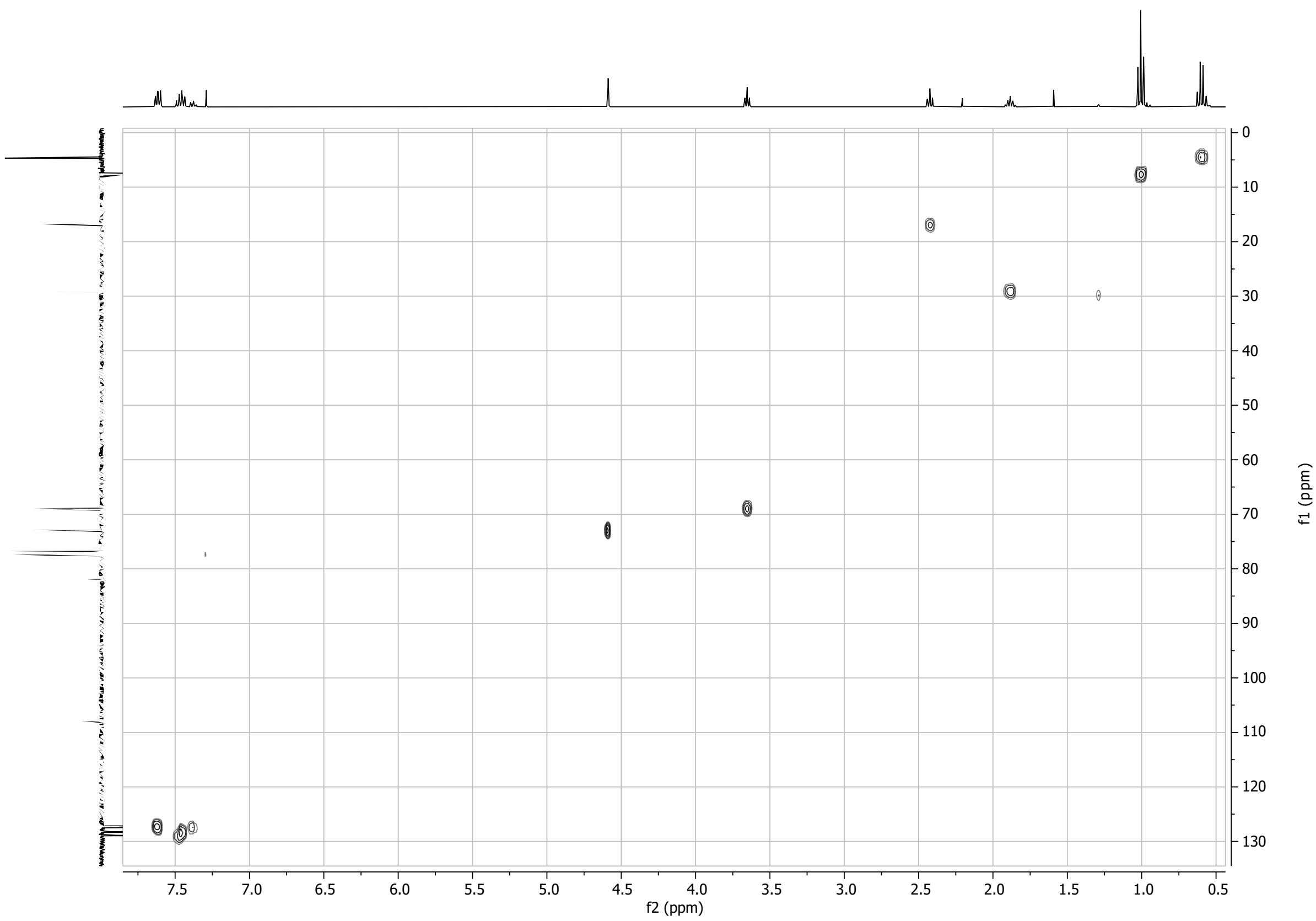

^{1}H-NMR spectrum of compound 32

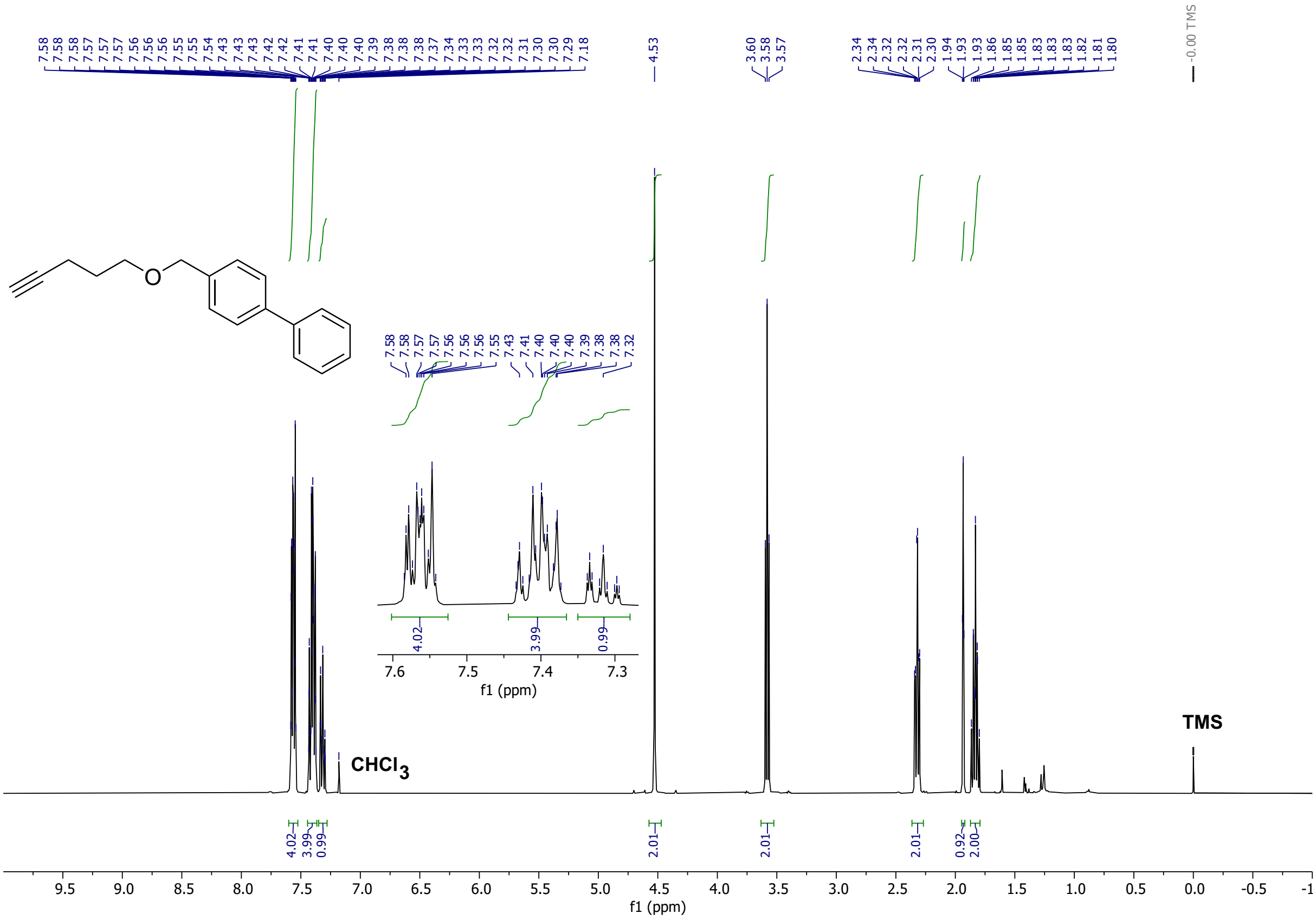

APT-NMR spectrum of compound 32

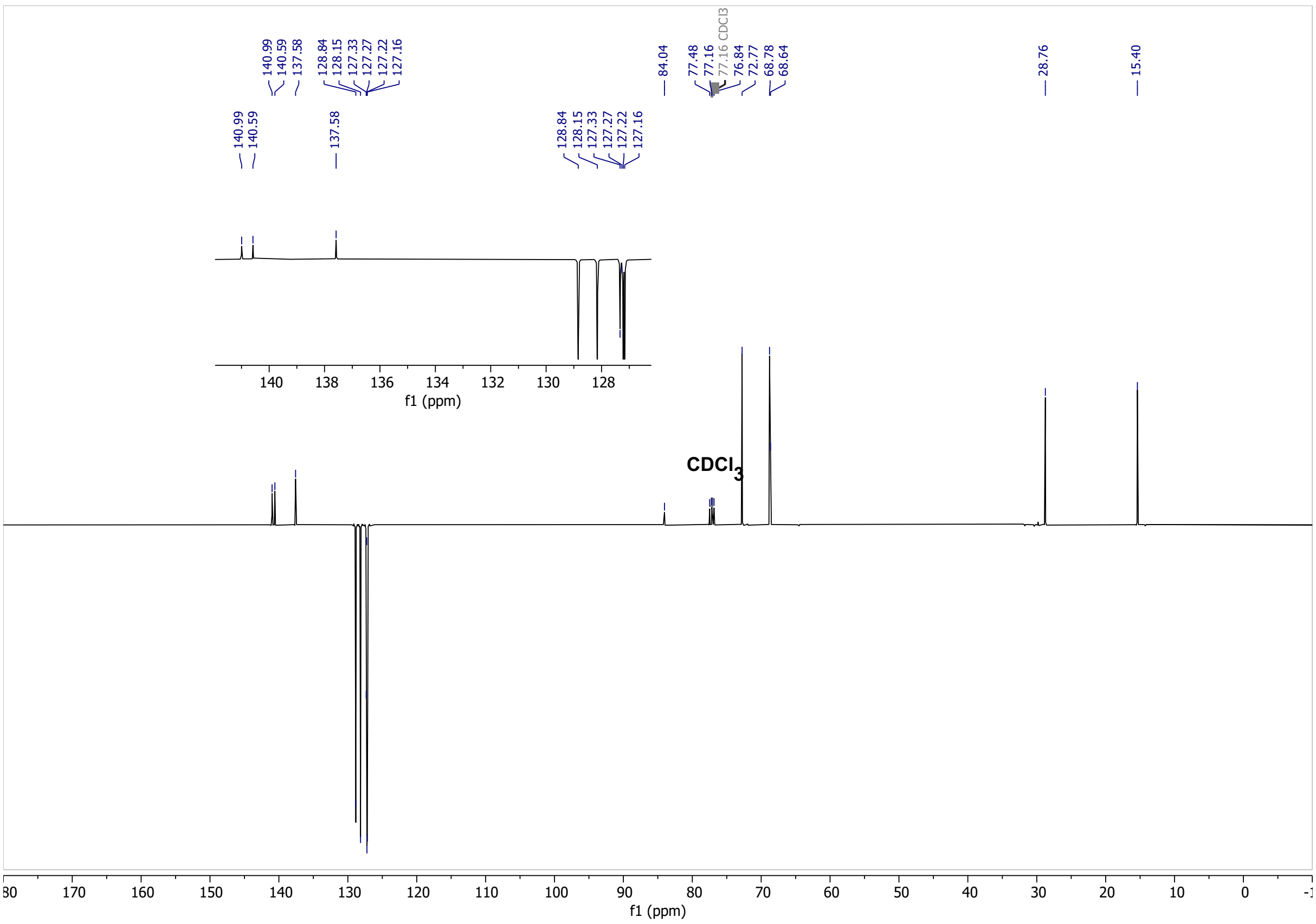

1H-NMR spectrum of compound 10

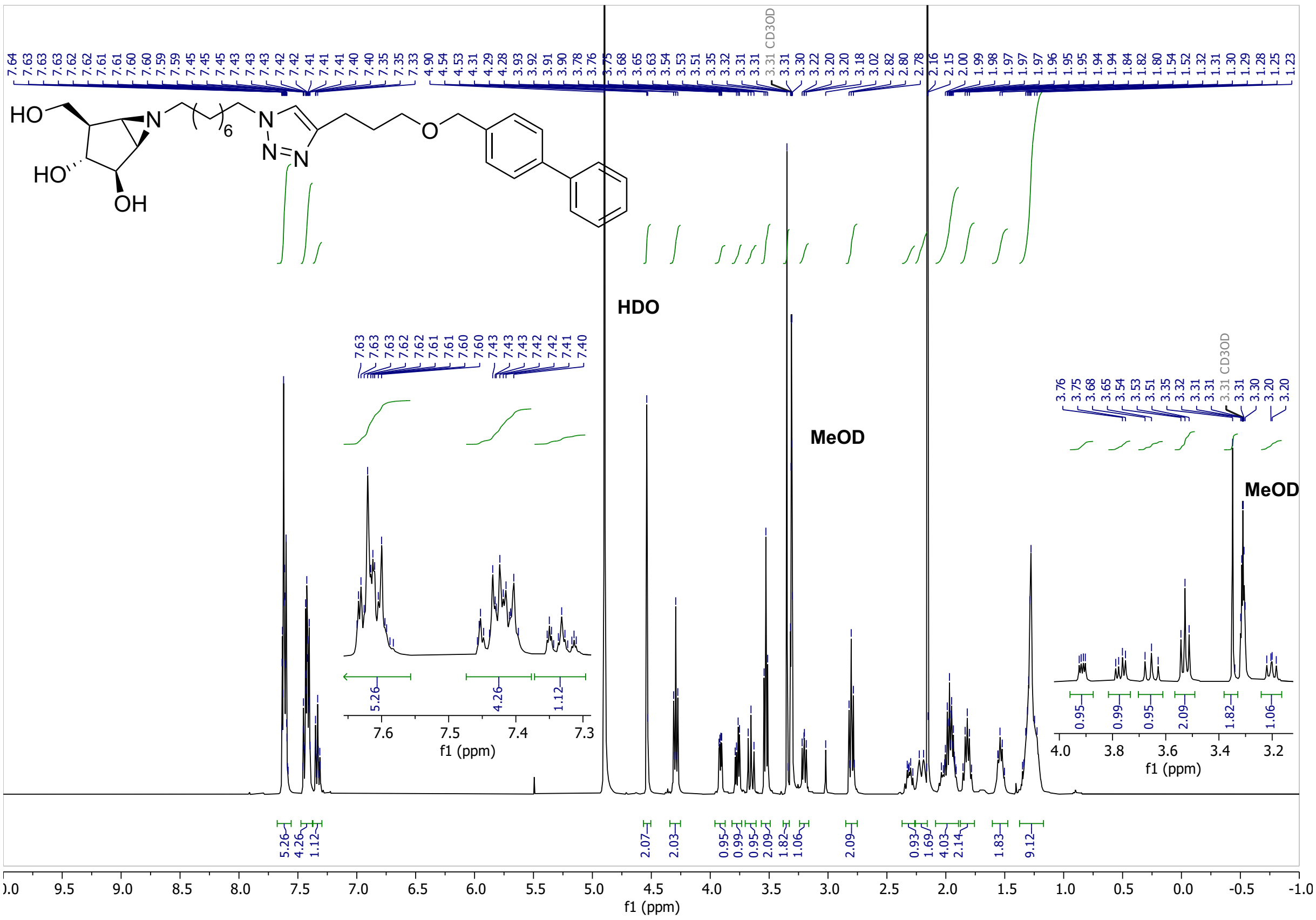

APT-NMR spectrum of compound 10

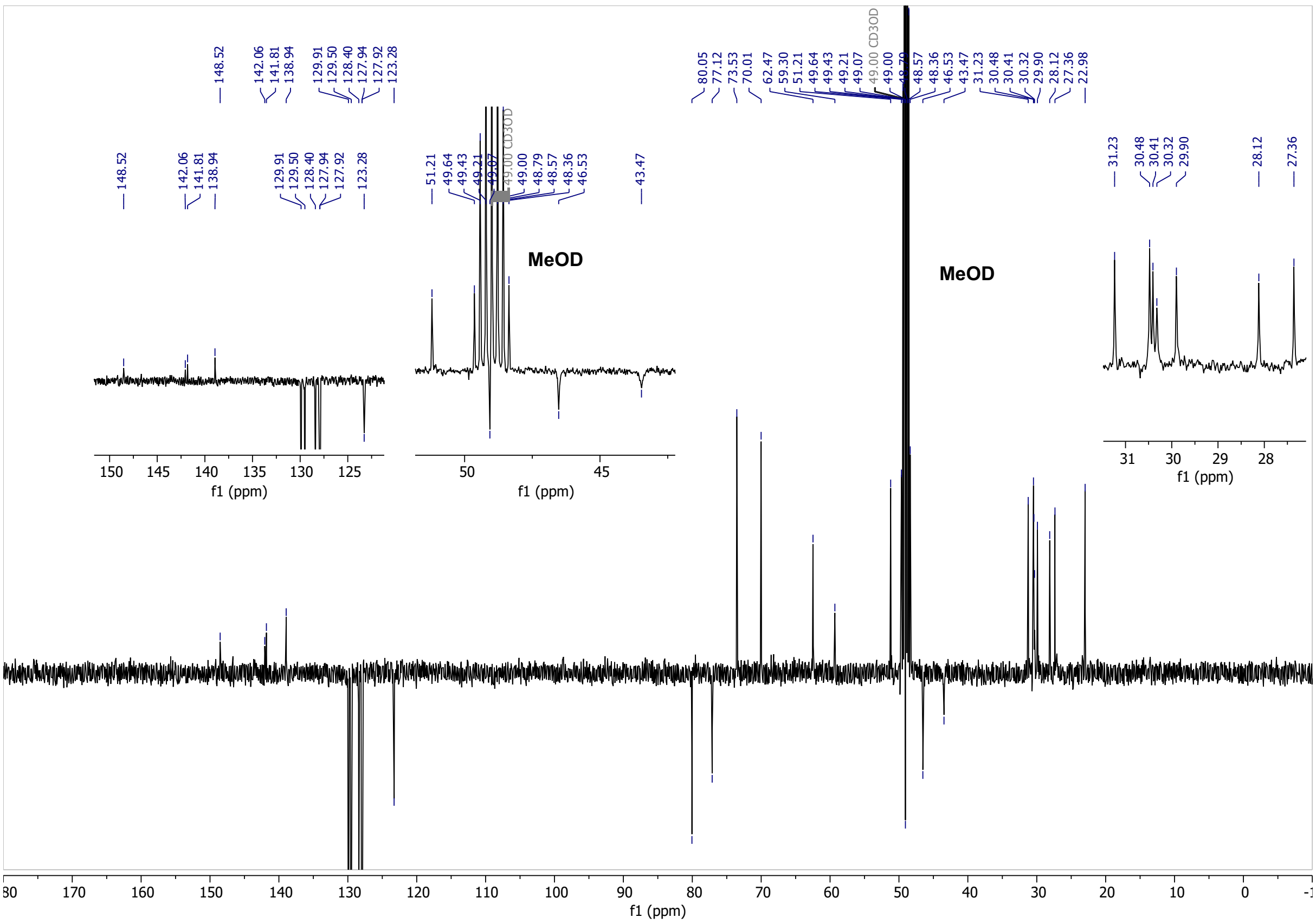

COSY-NMR spectrum of compound 10

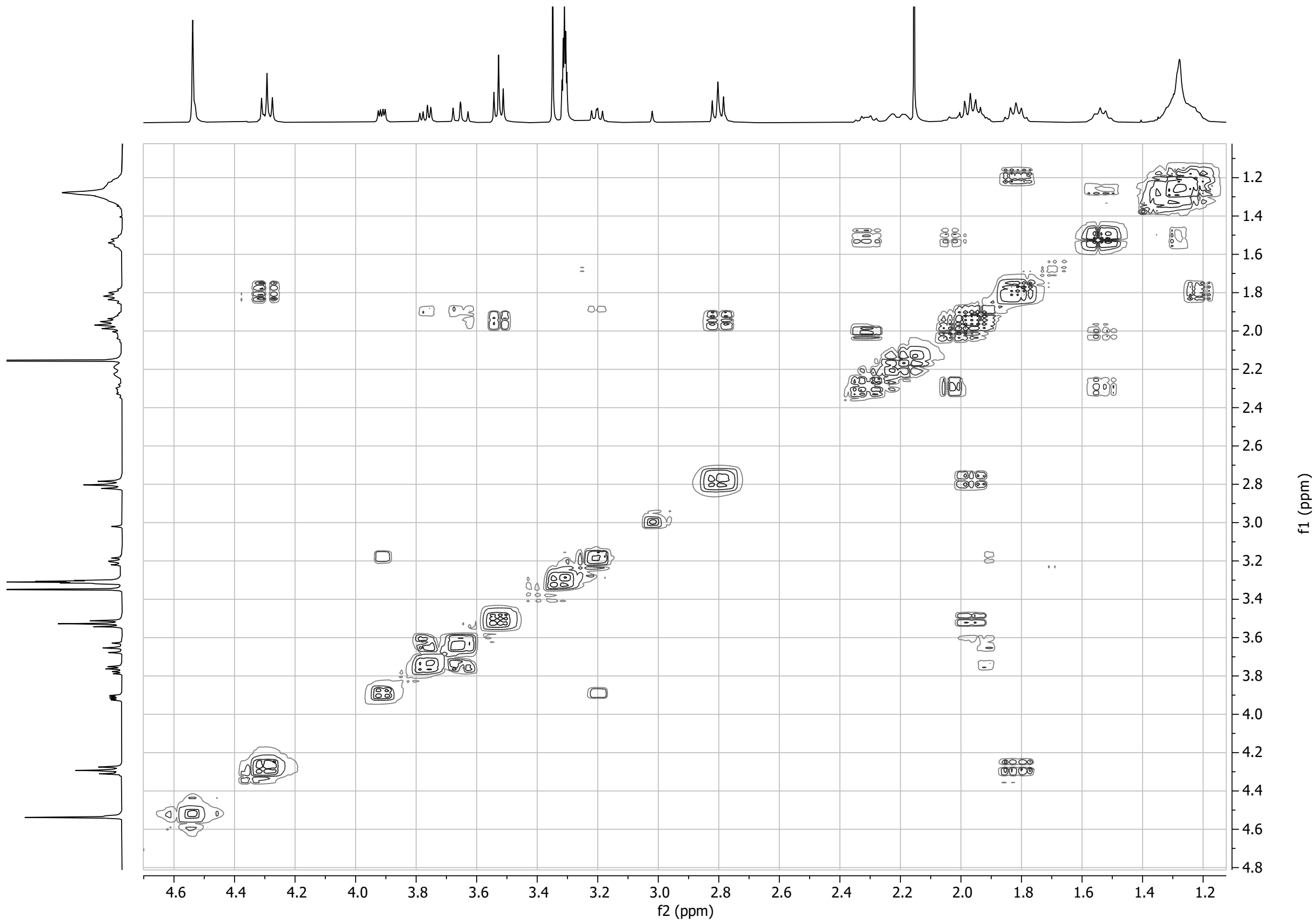

HSQC-NMR spectrum of compound 10

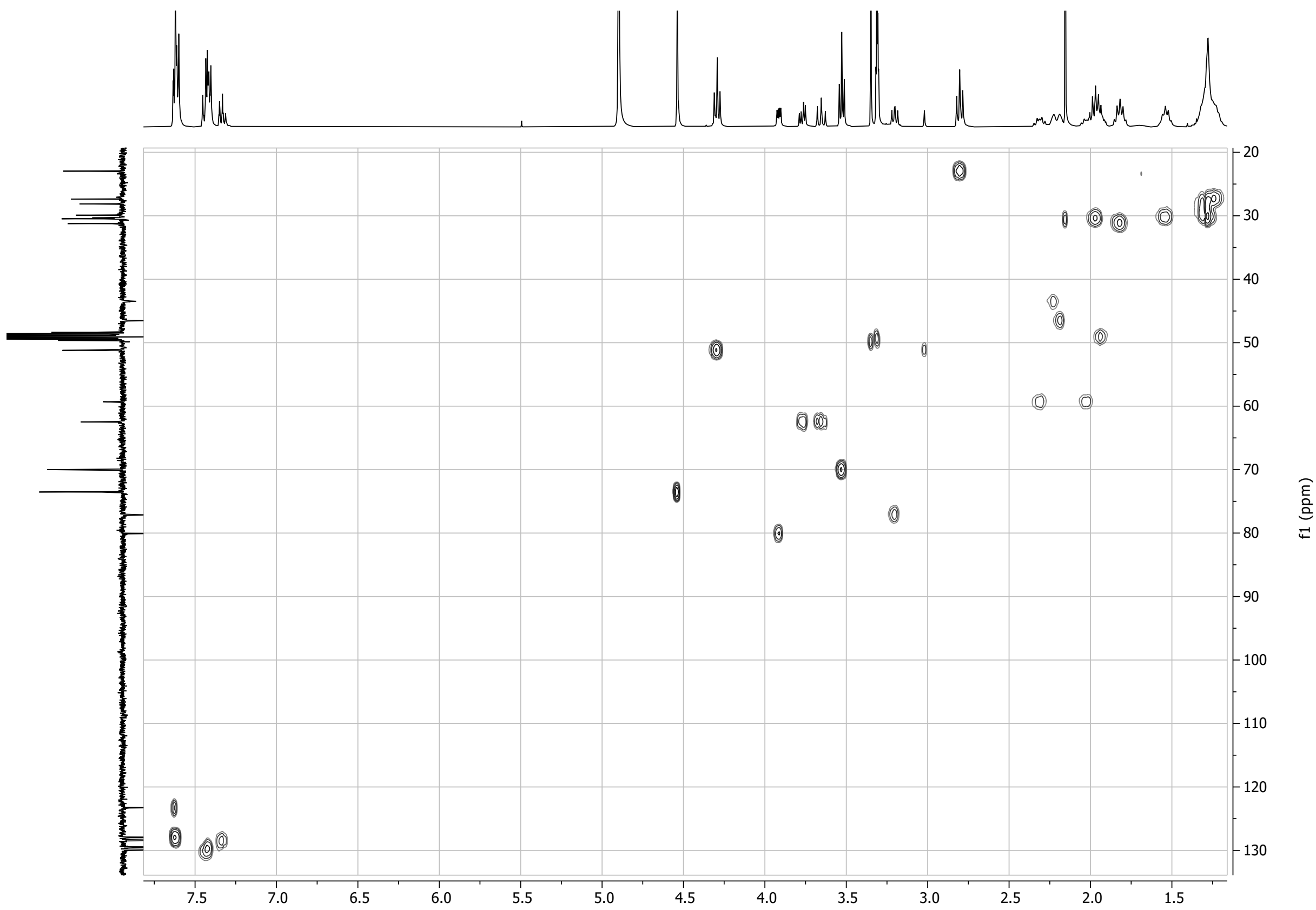

HRMS spectra

HRMS spectrum of Compound **12**

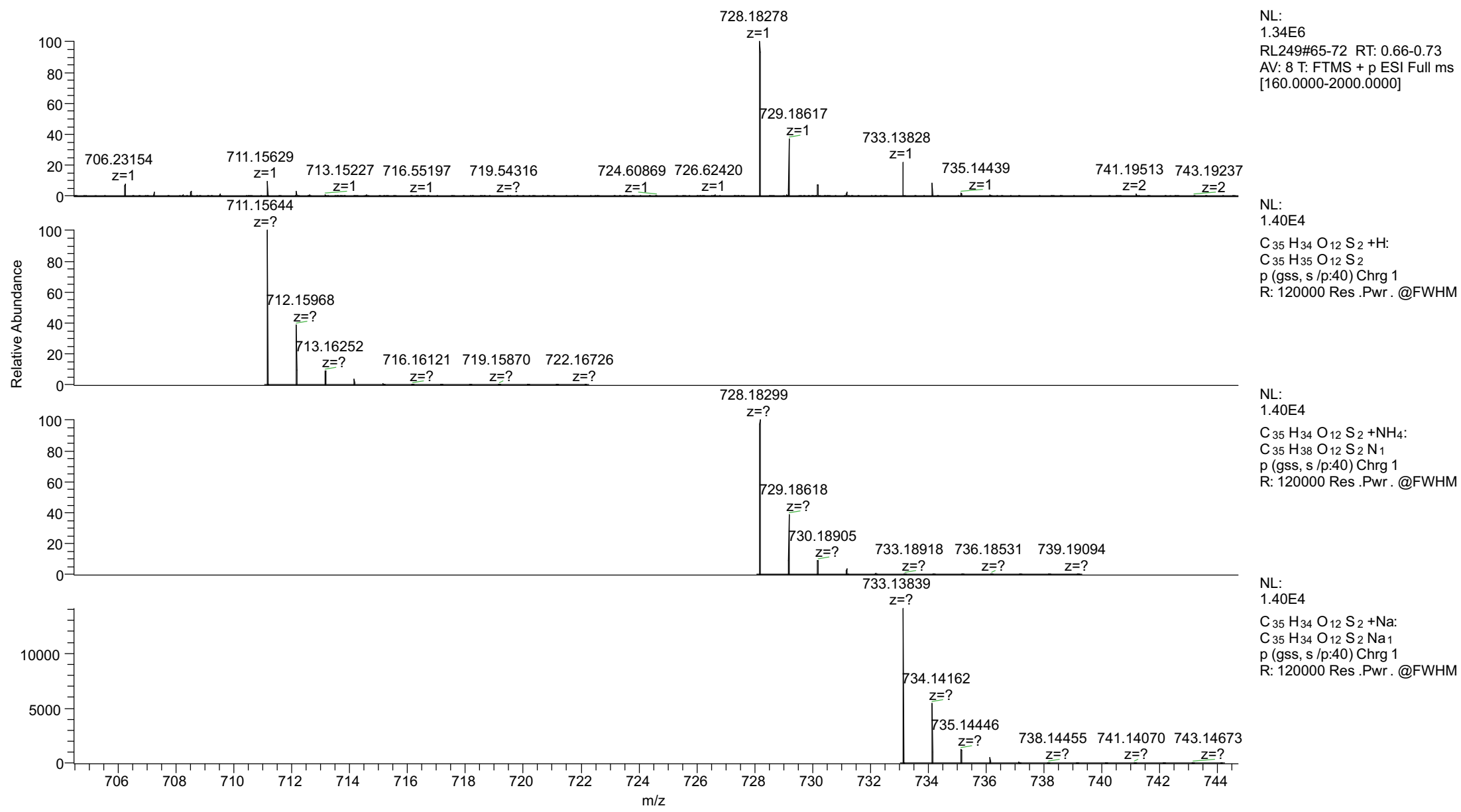

HRMS spectrum of Compound **13**

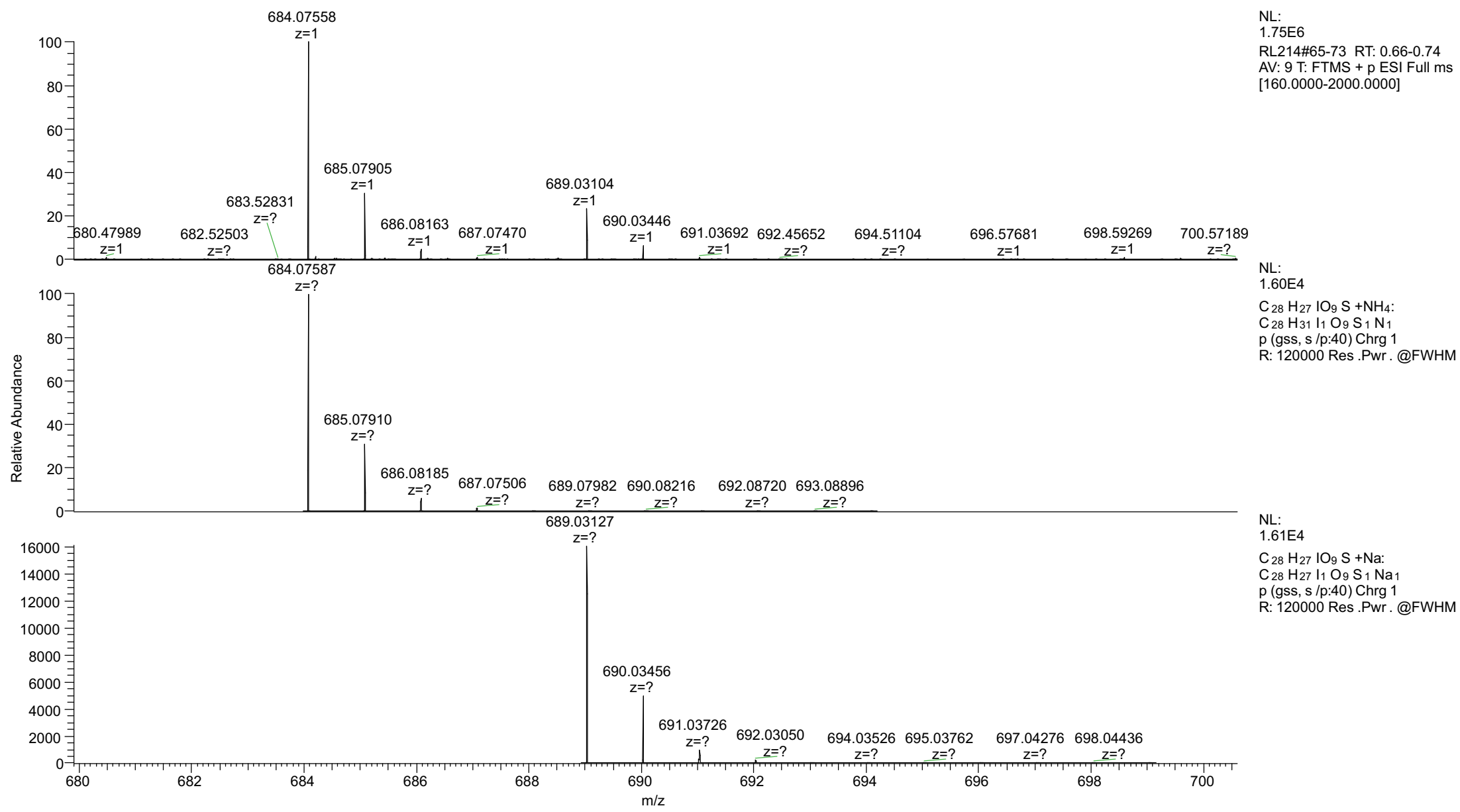

HRMS spectrum of Compound **14**

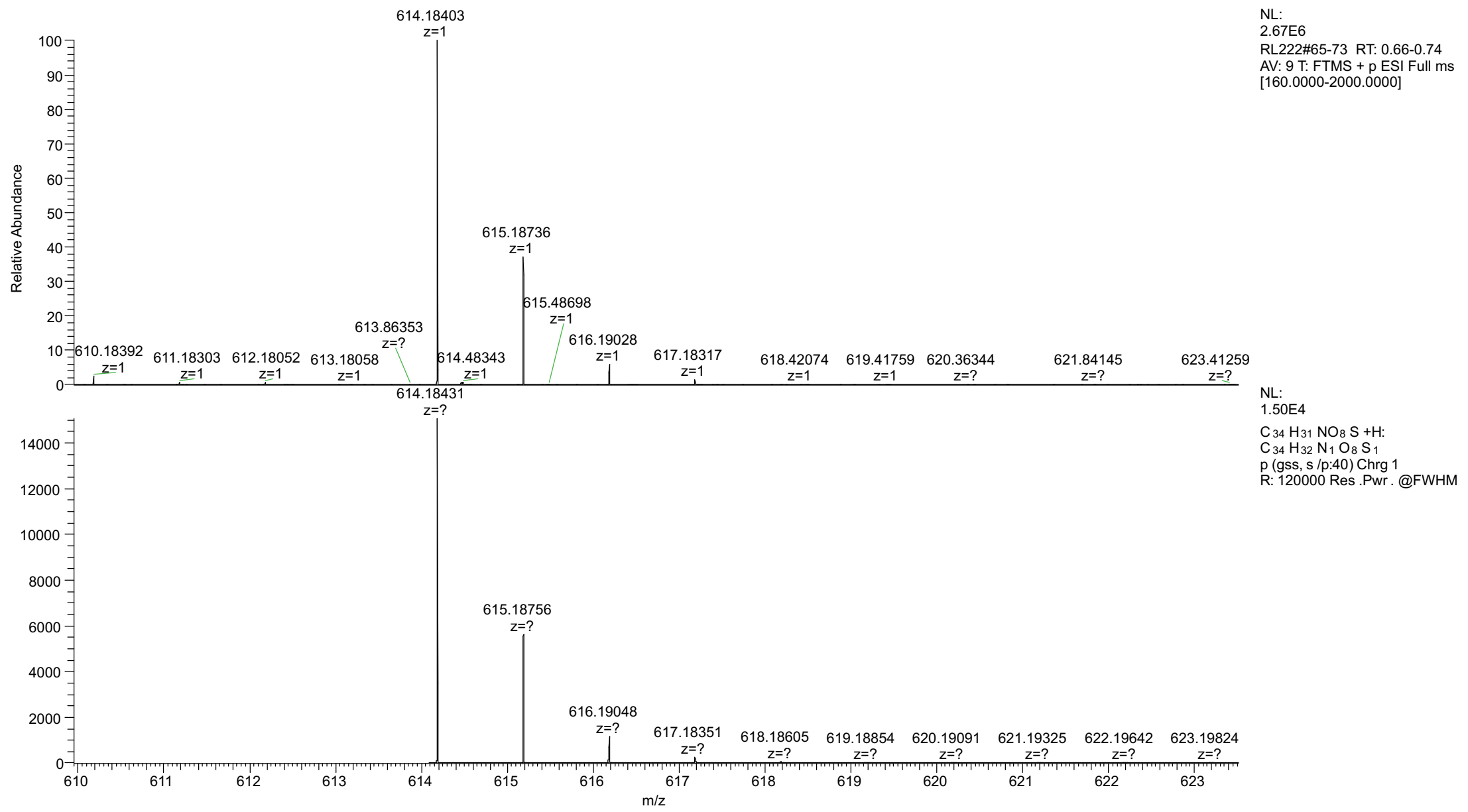

HRMS spectrum of Compound **15**

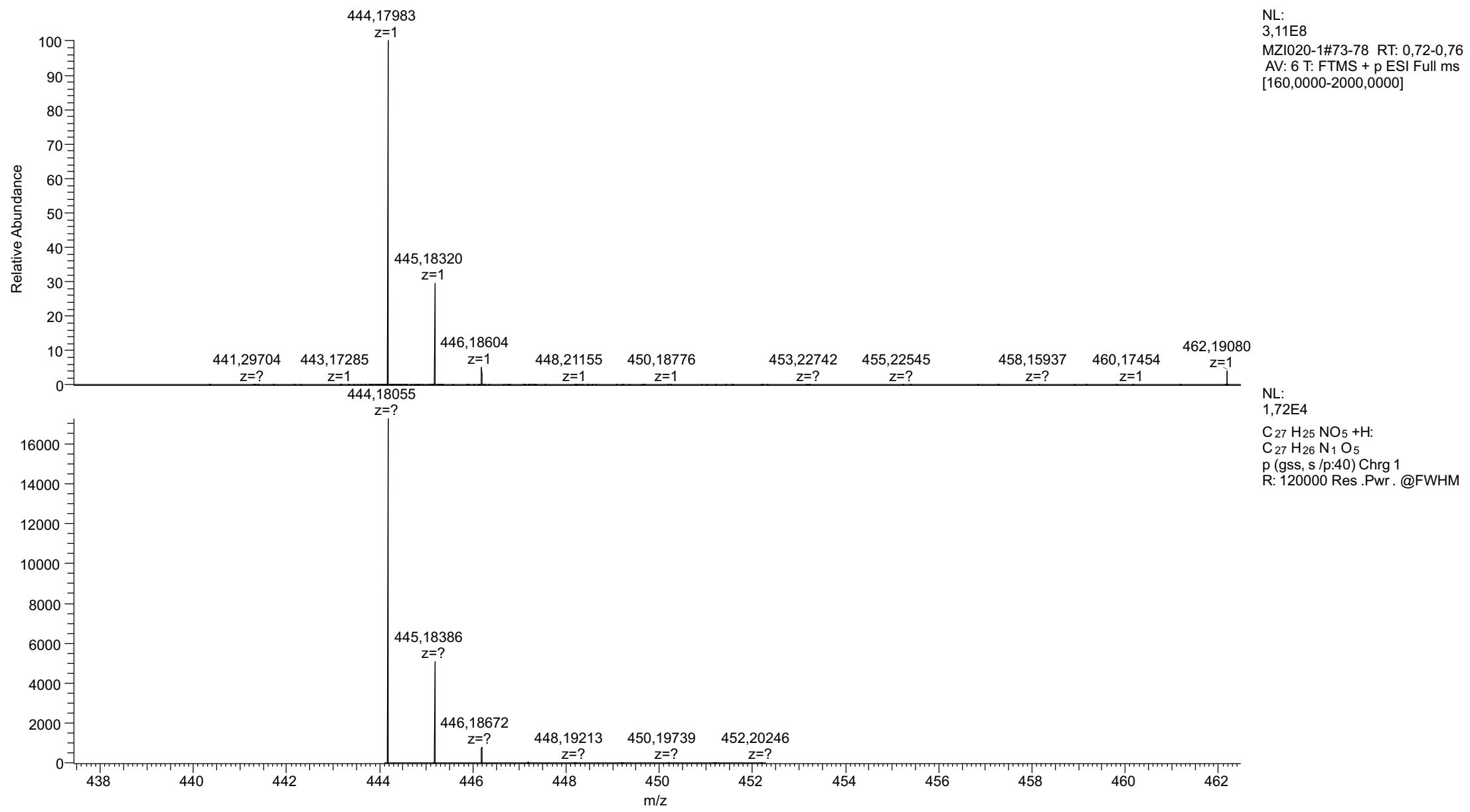

HRMS spectrum of Compound **16**

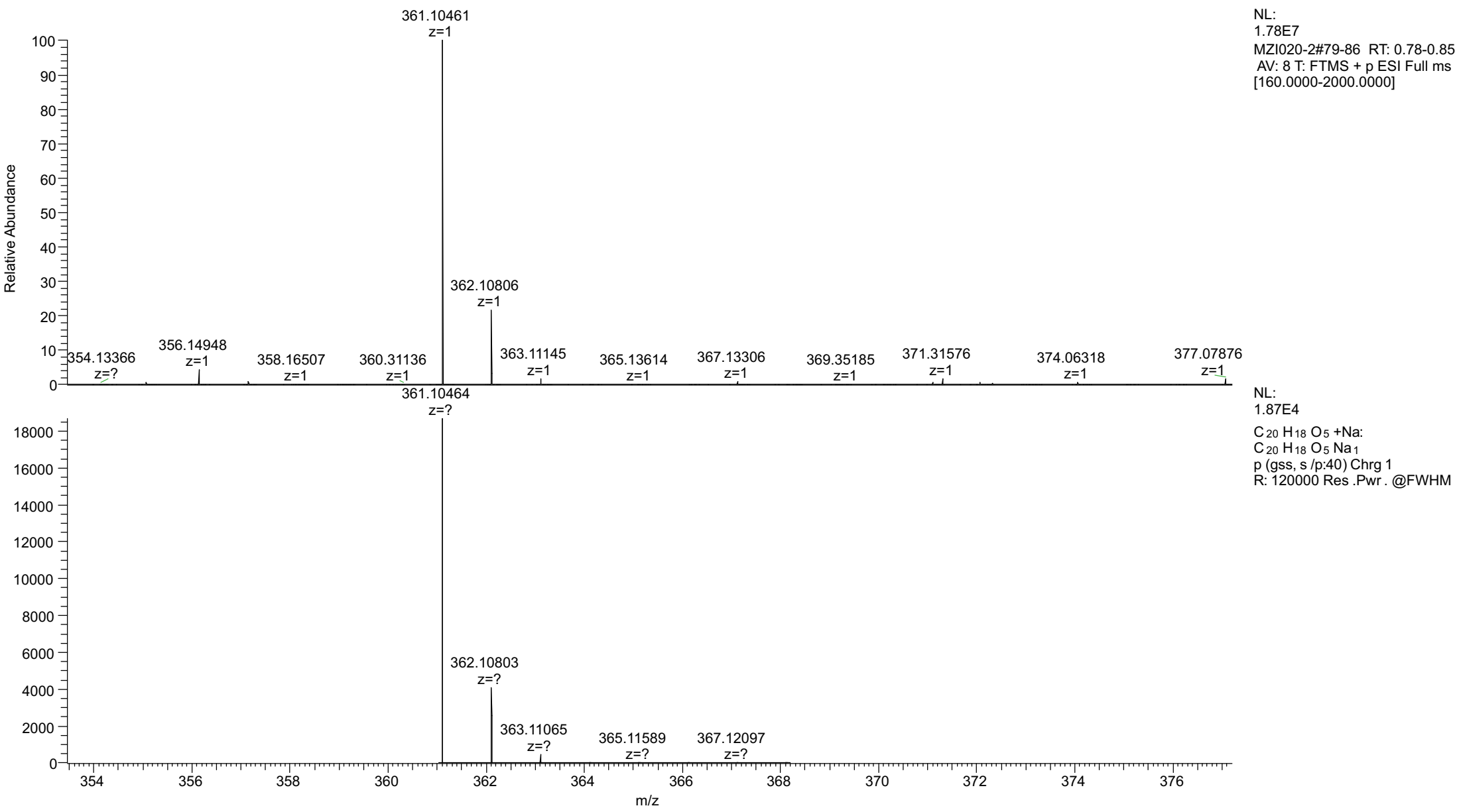

HRMS spectrum of Compound **17**

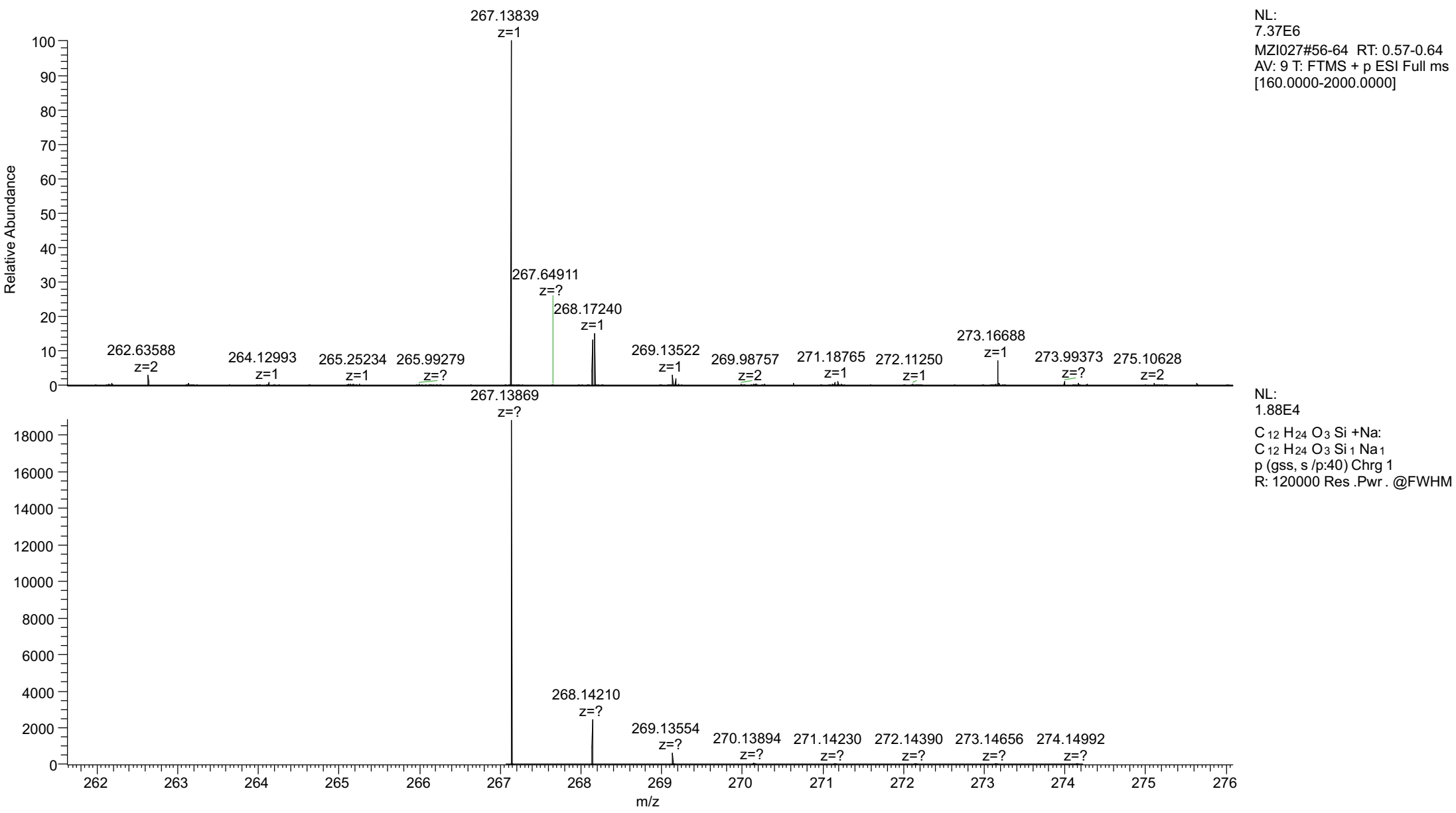

HRMS spectrum of Compound **18**

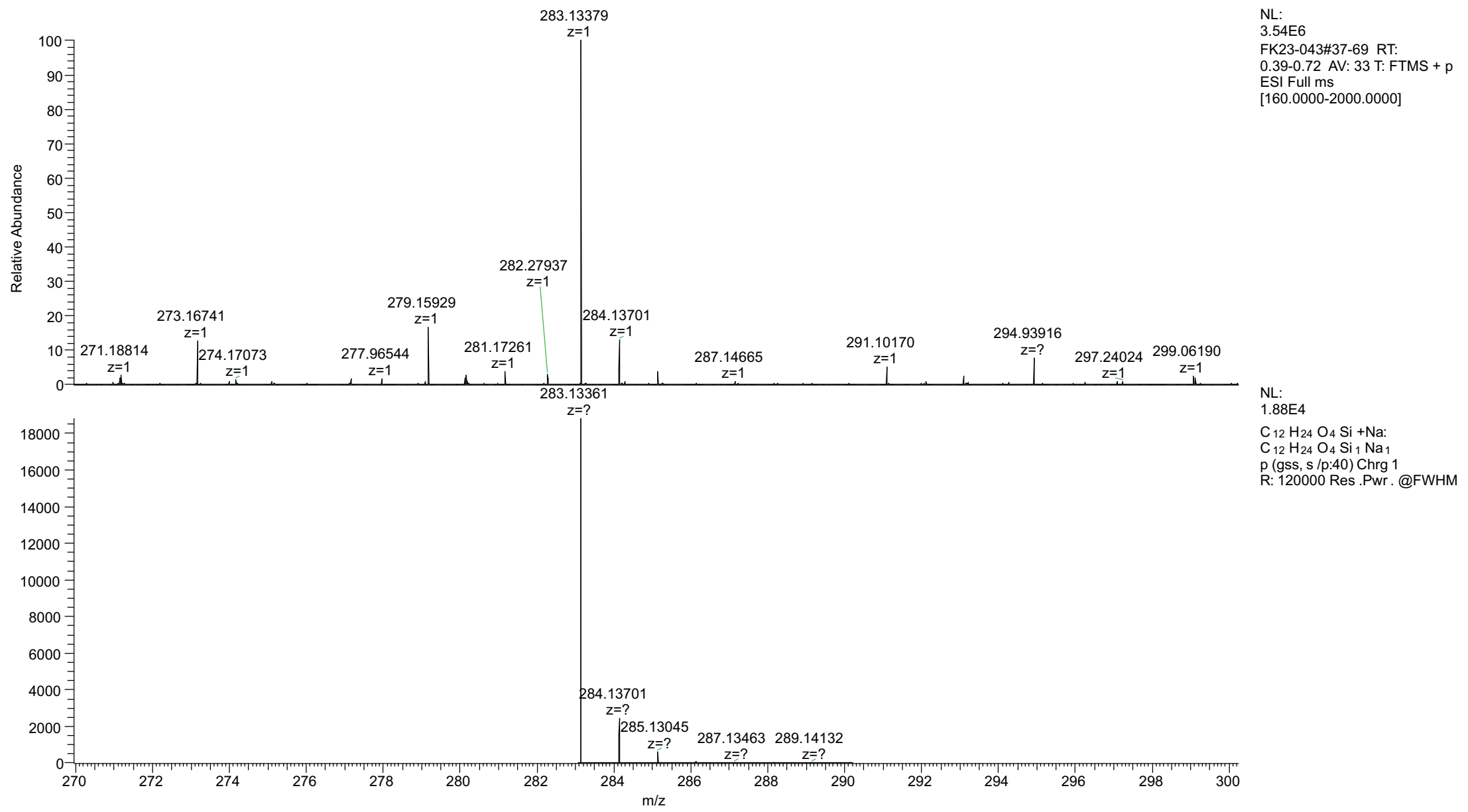

HRMS spectrum of Compound **1**

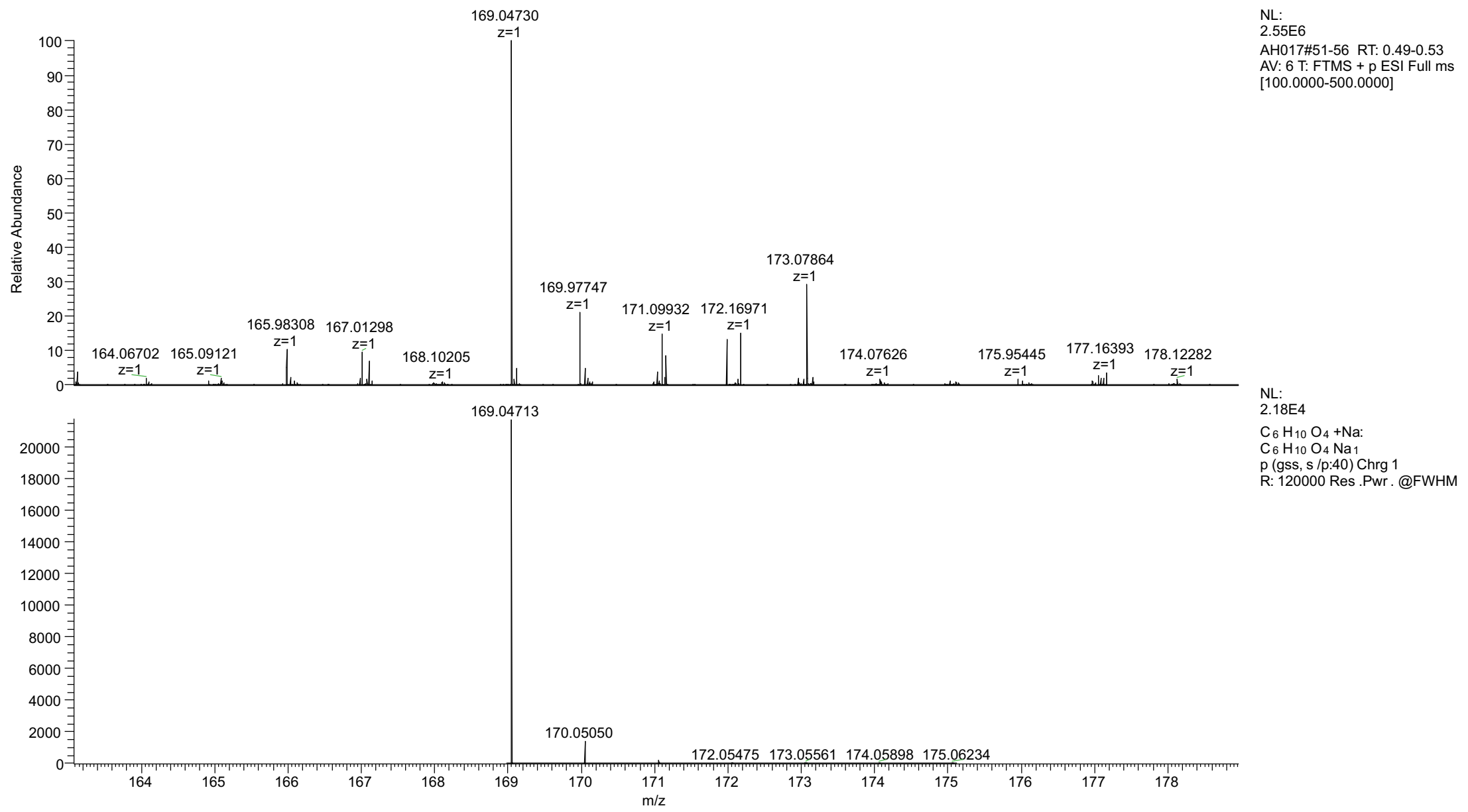

HRMS spectrum of Compound **19**

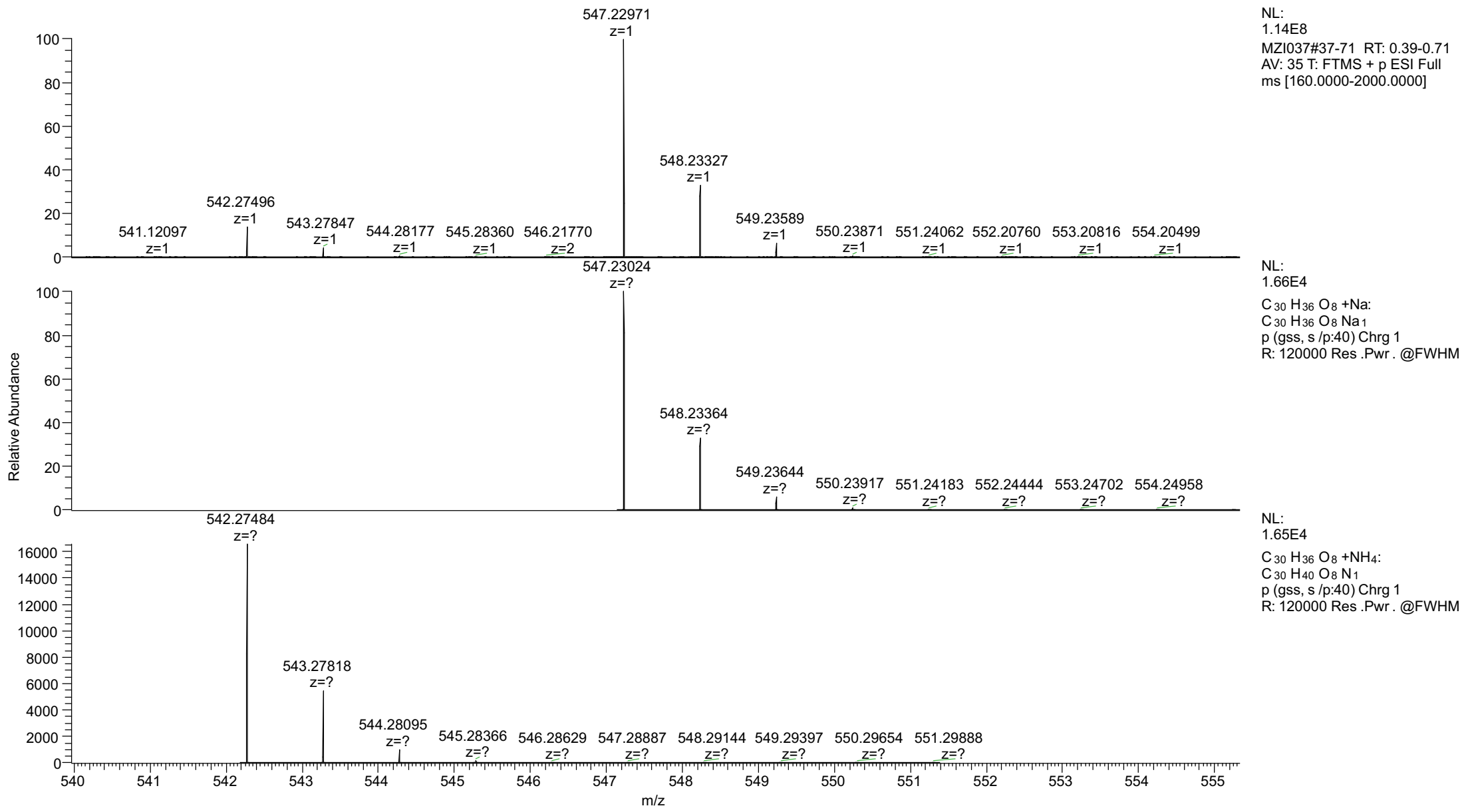

HRMS spectrum of Compound **20**

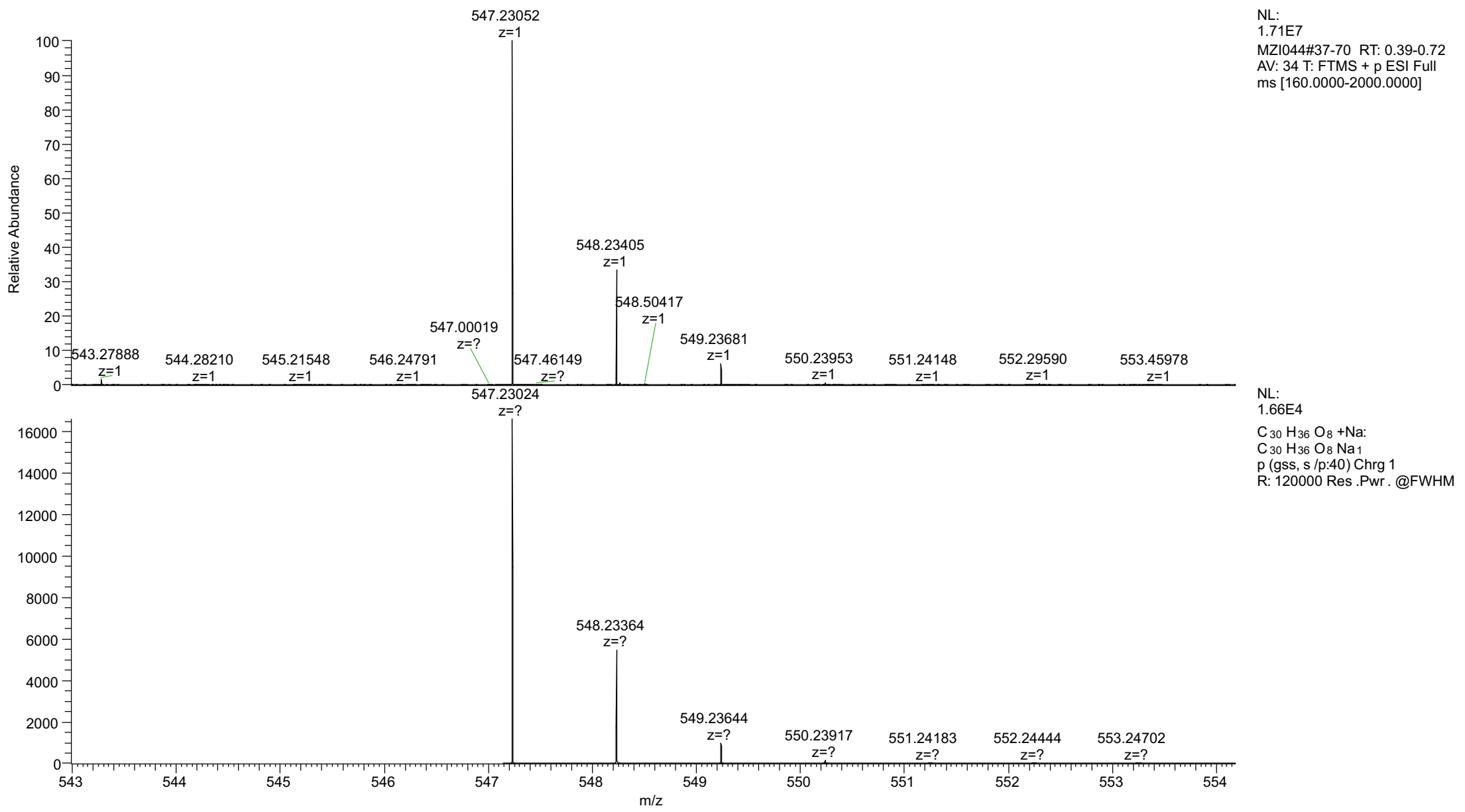

HRMS spectrum of Compound **21**

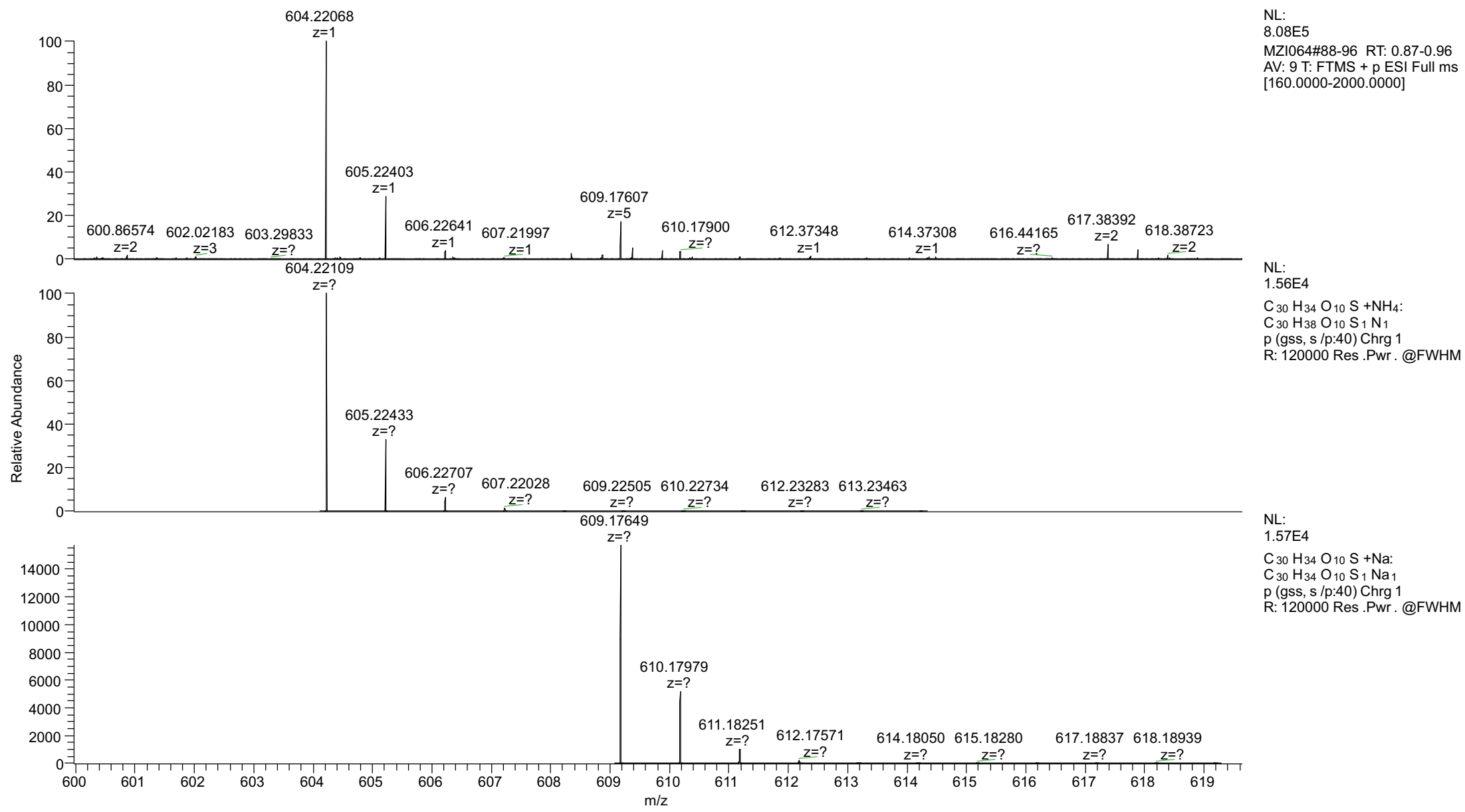

HRMS spectrum of Compound **22**

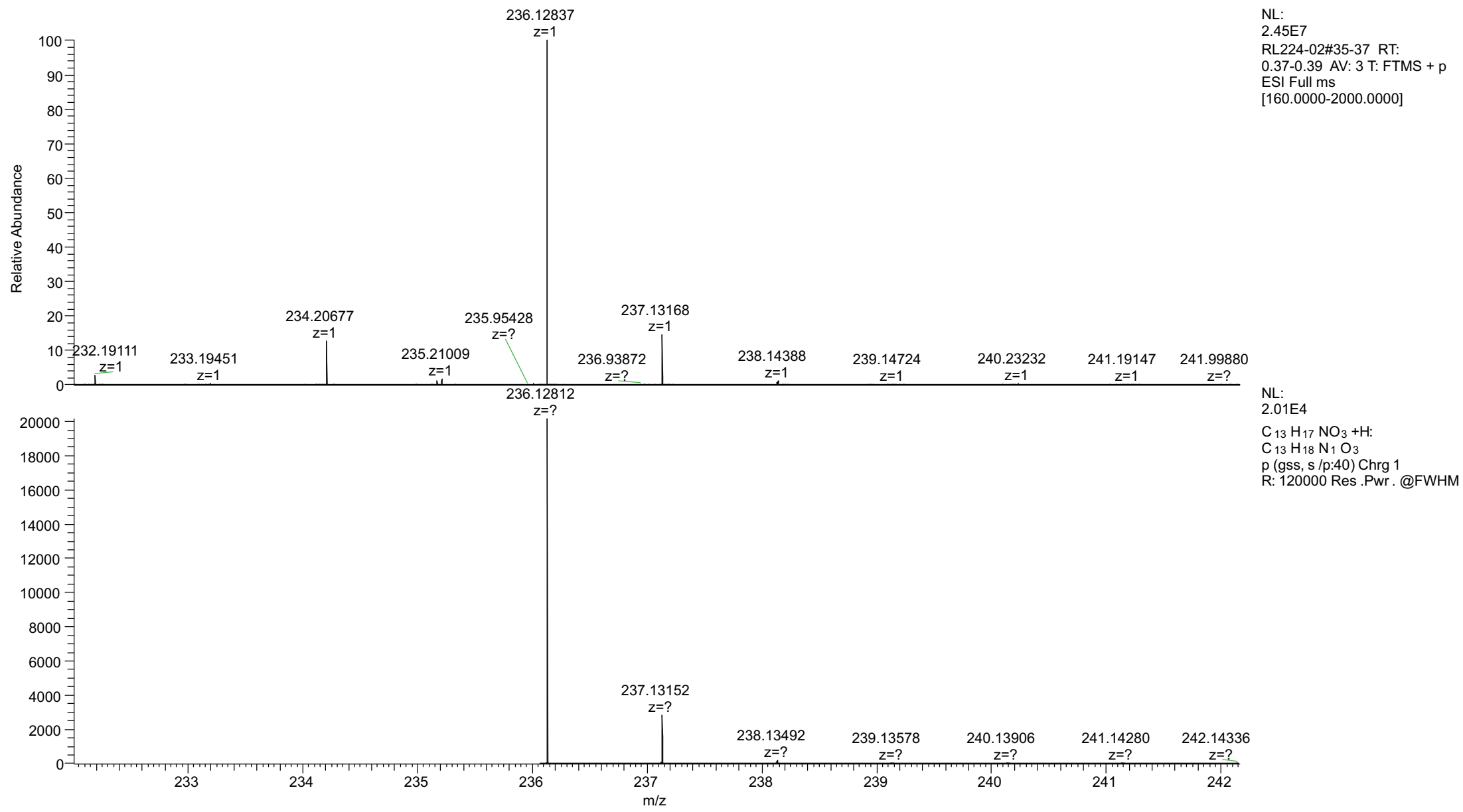

HRMS spectrum of Compound **3**

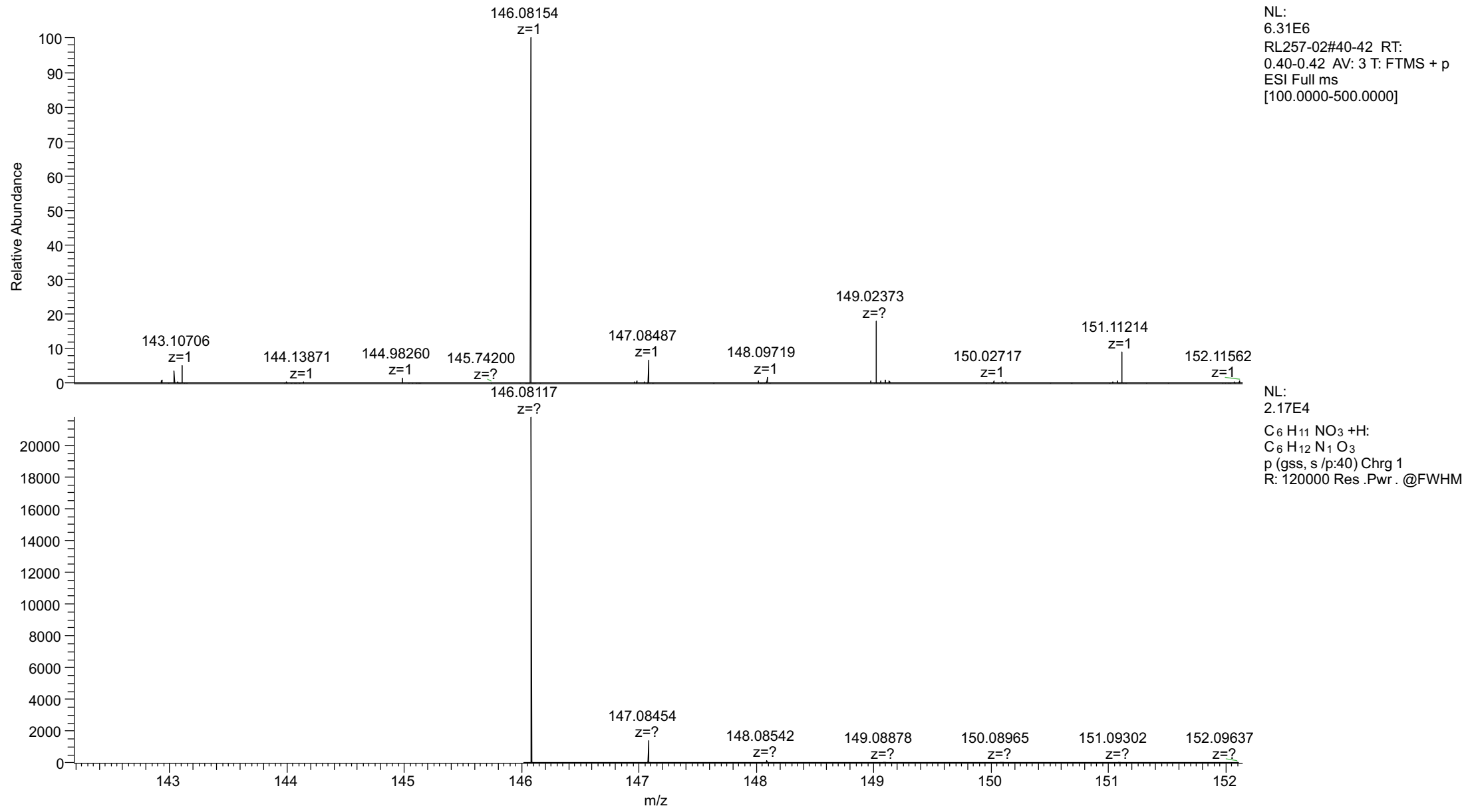

HRMS spectrum of Compound **4**

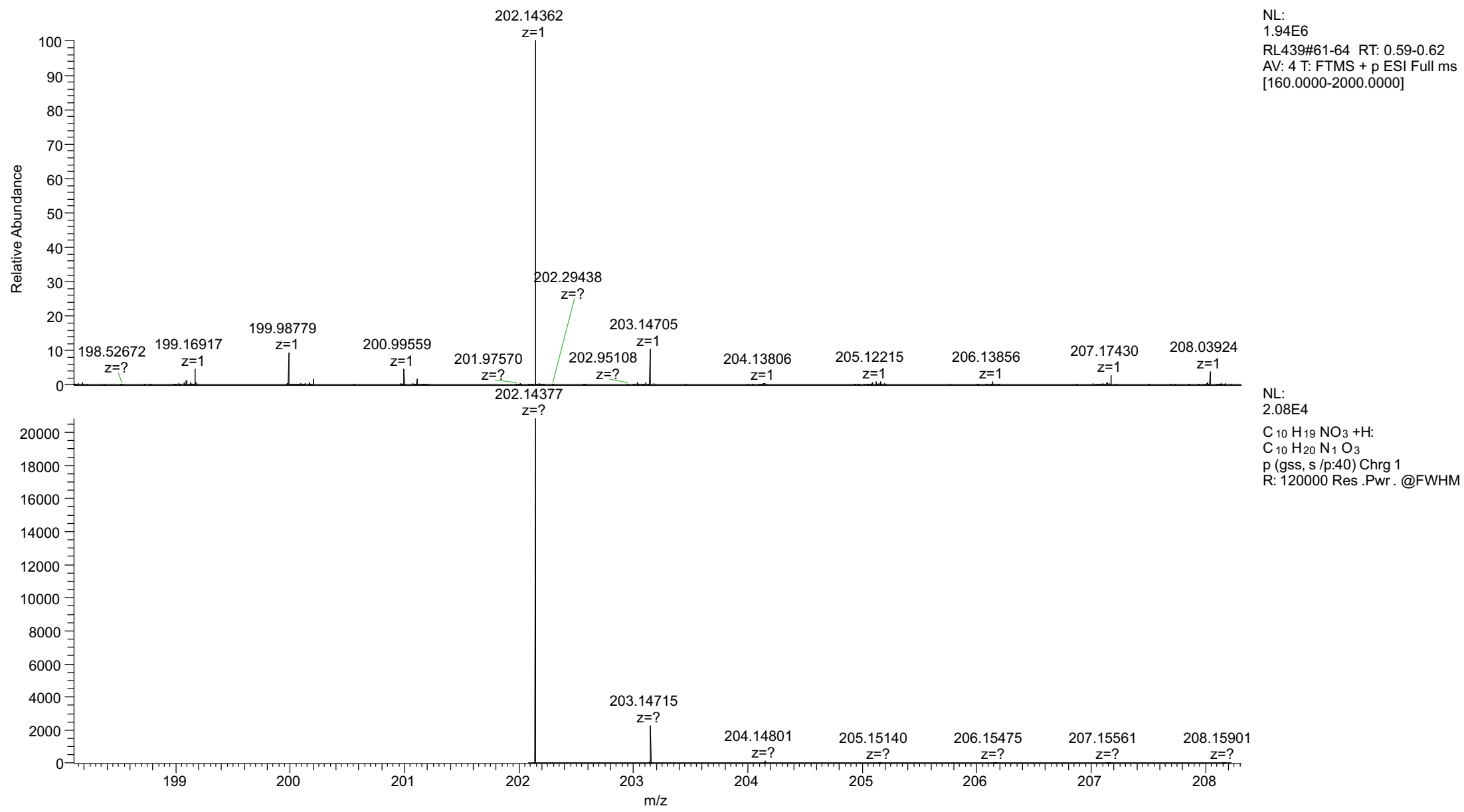

HRMS spectrum of Compound **5**

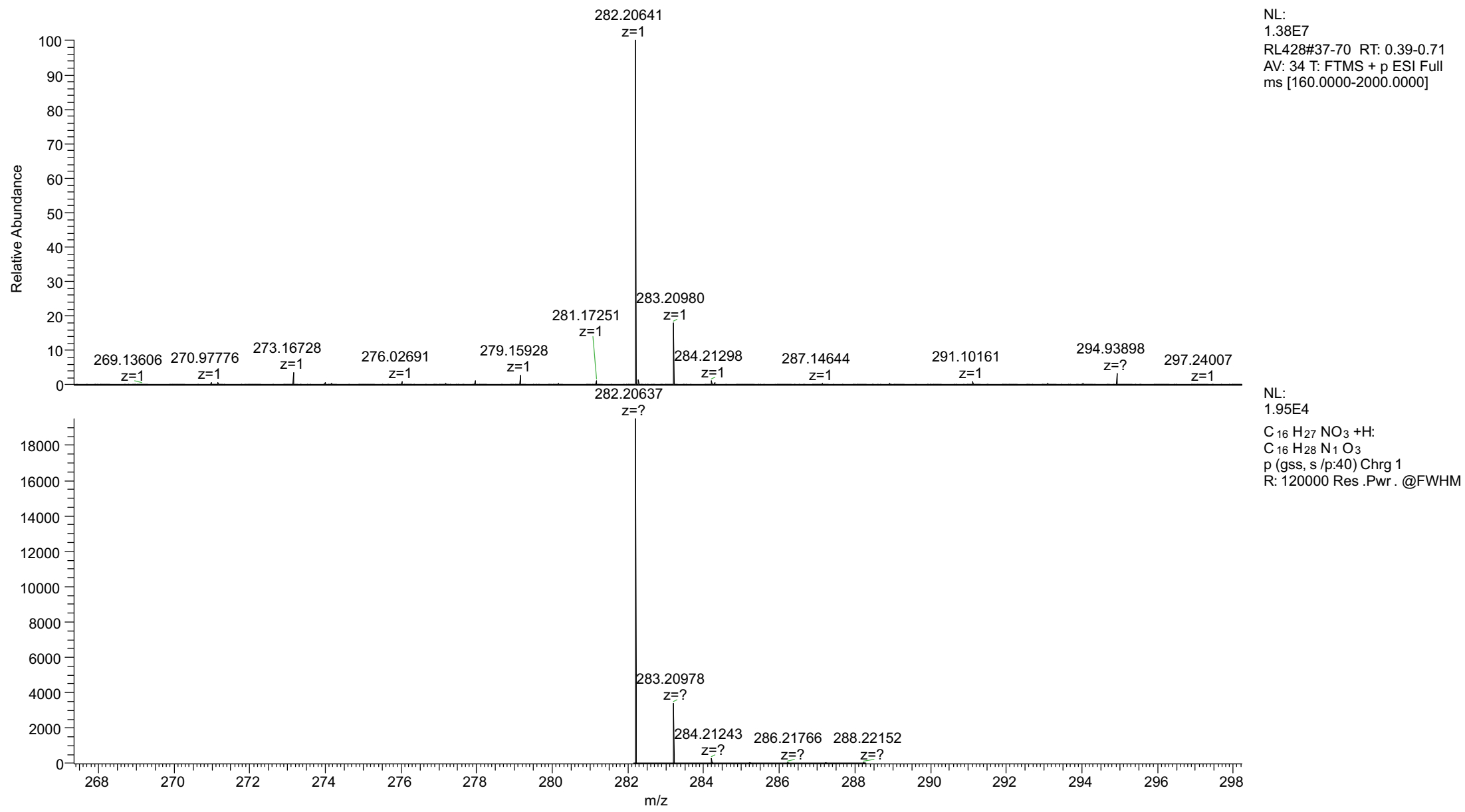

HRMS spectrum of Compound **7**

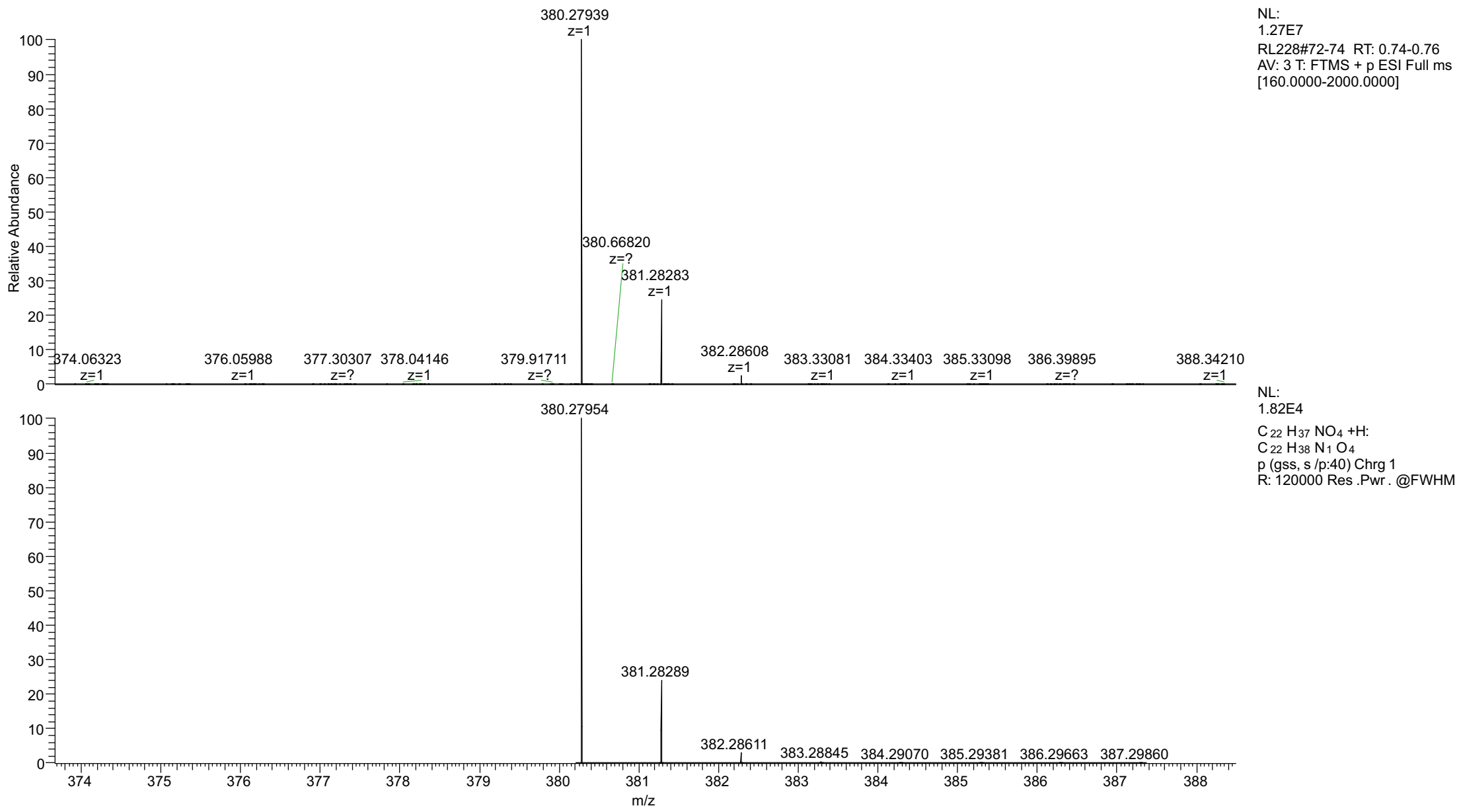

HRMS spectrum of Compound **8**

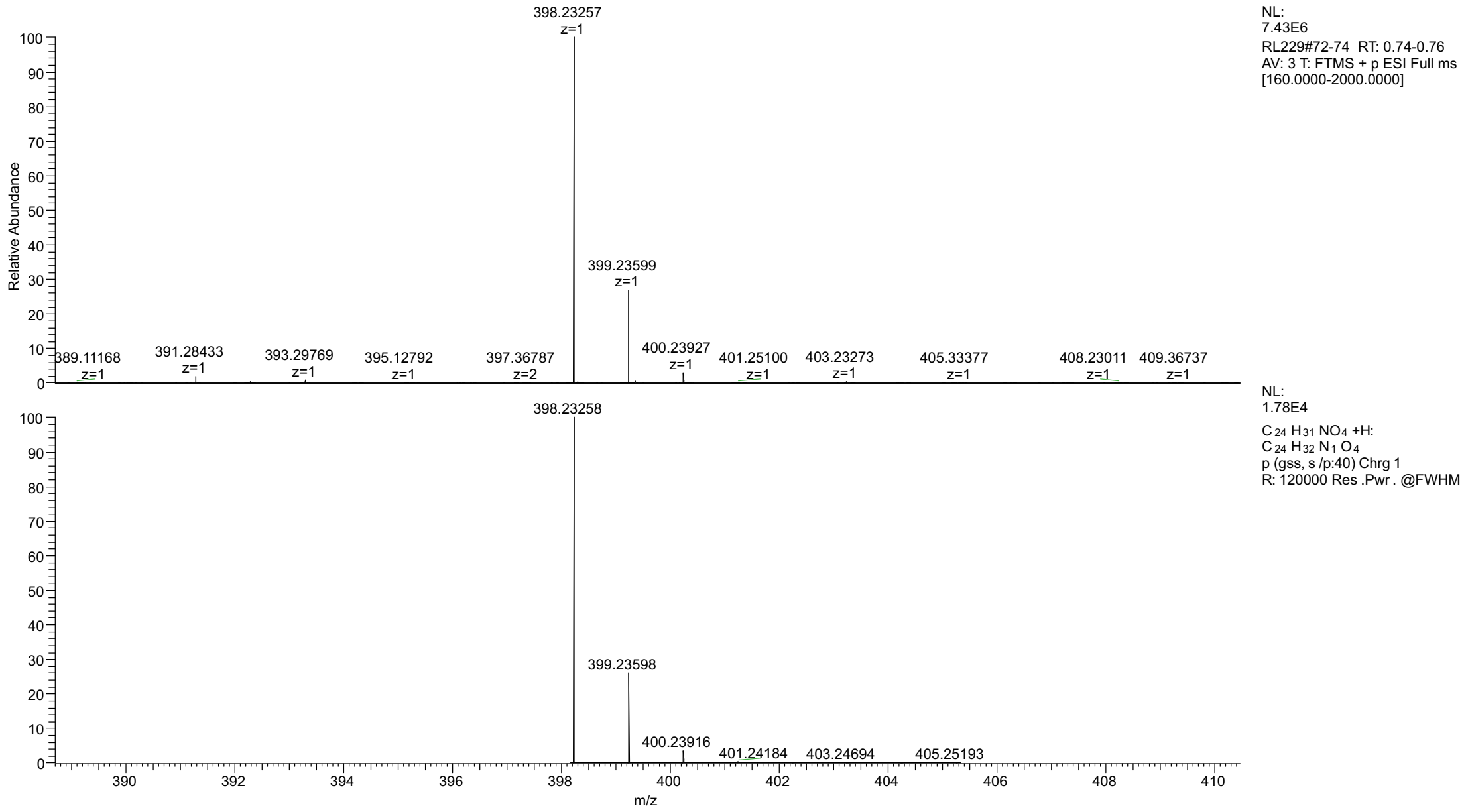

HRMS spectrum of Compound **9**

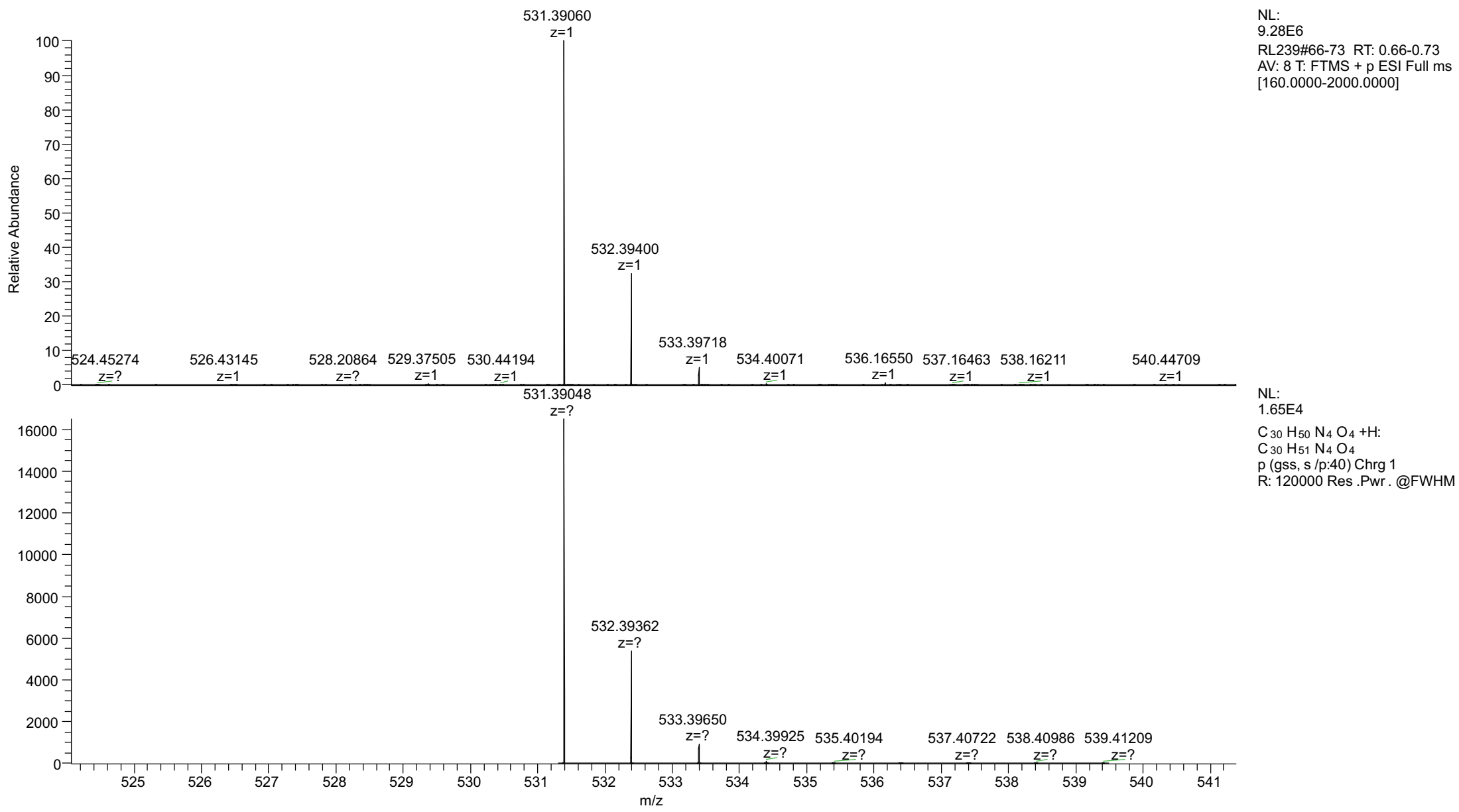

HRMS spectrum of Compound **31**

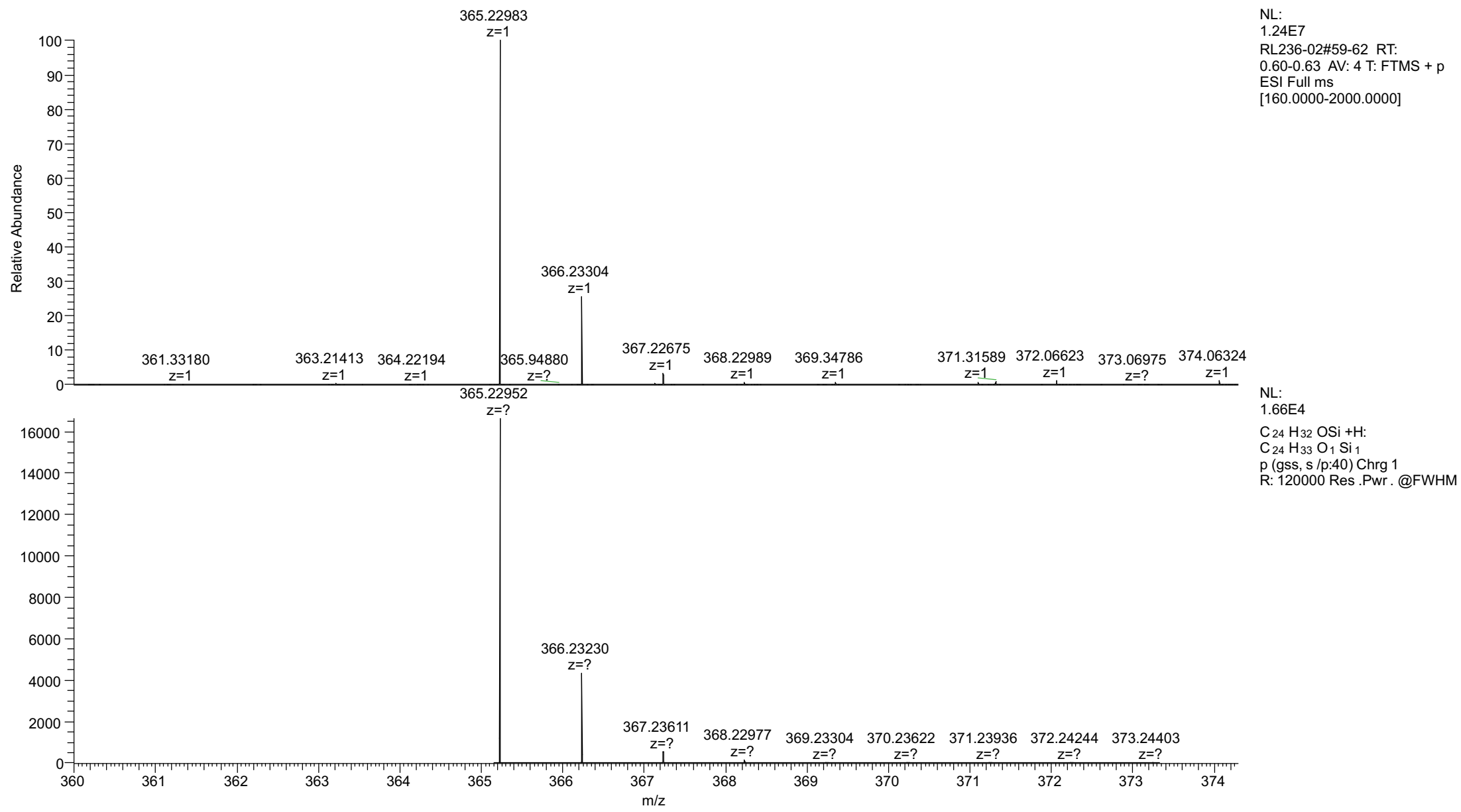

HRMS spectrum of Compound **32**

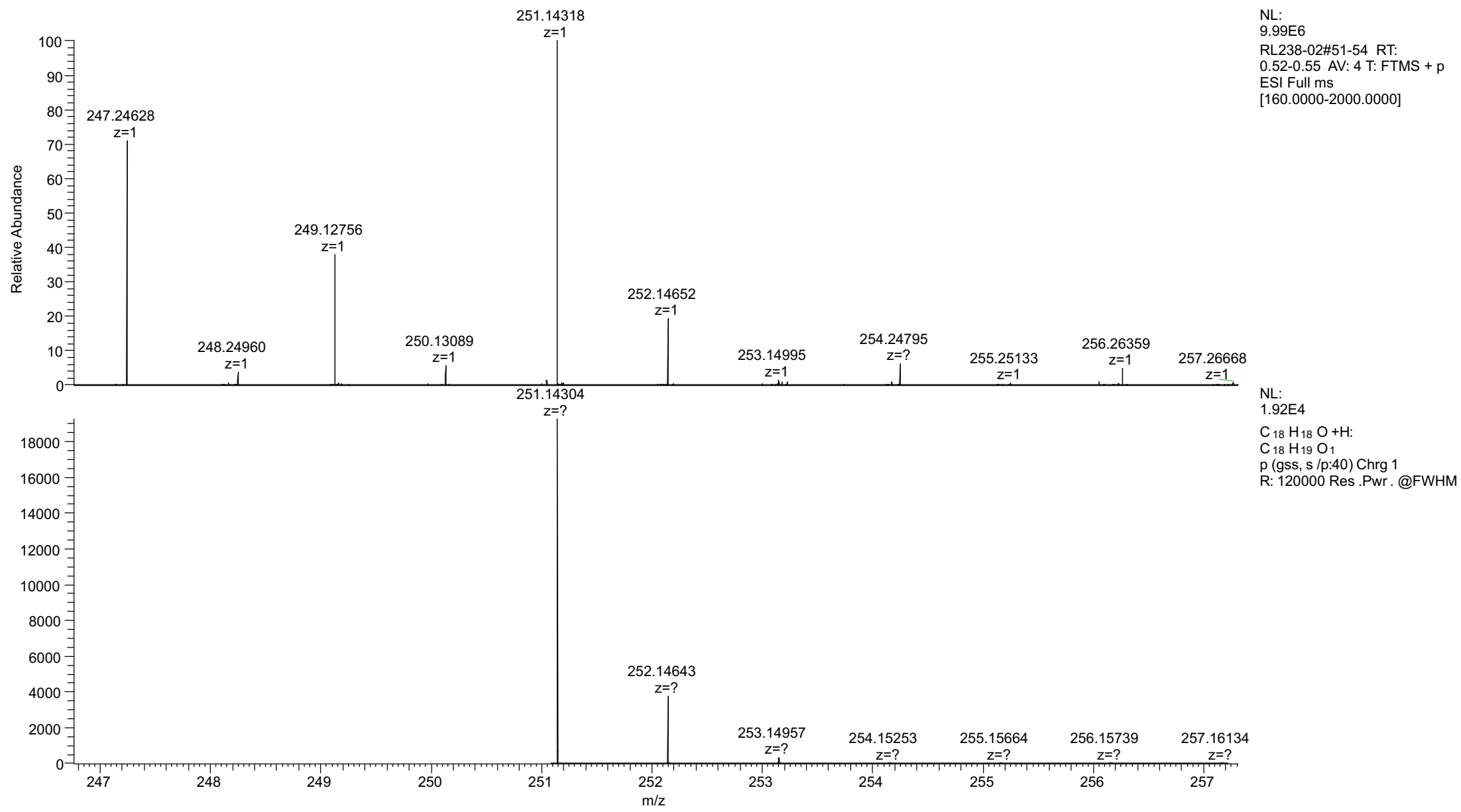

HRMS spectrum of Compound **10**

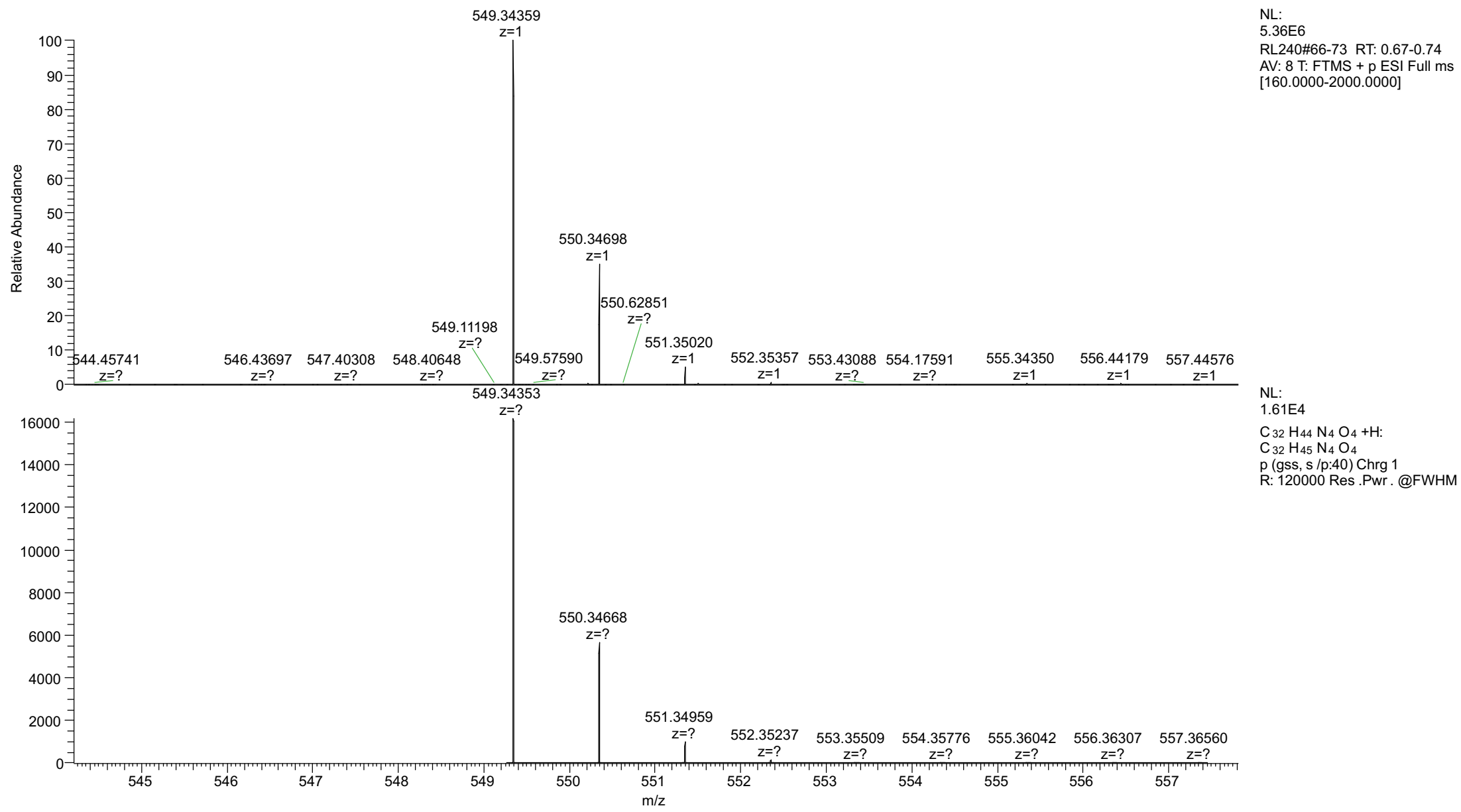

LC-MS spectra

LC-MS spectrum of Compound **4**

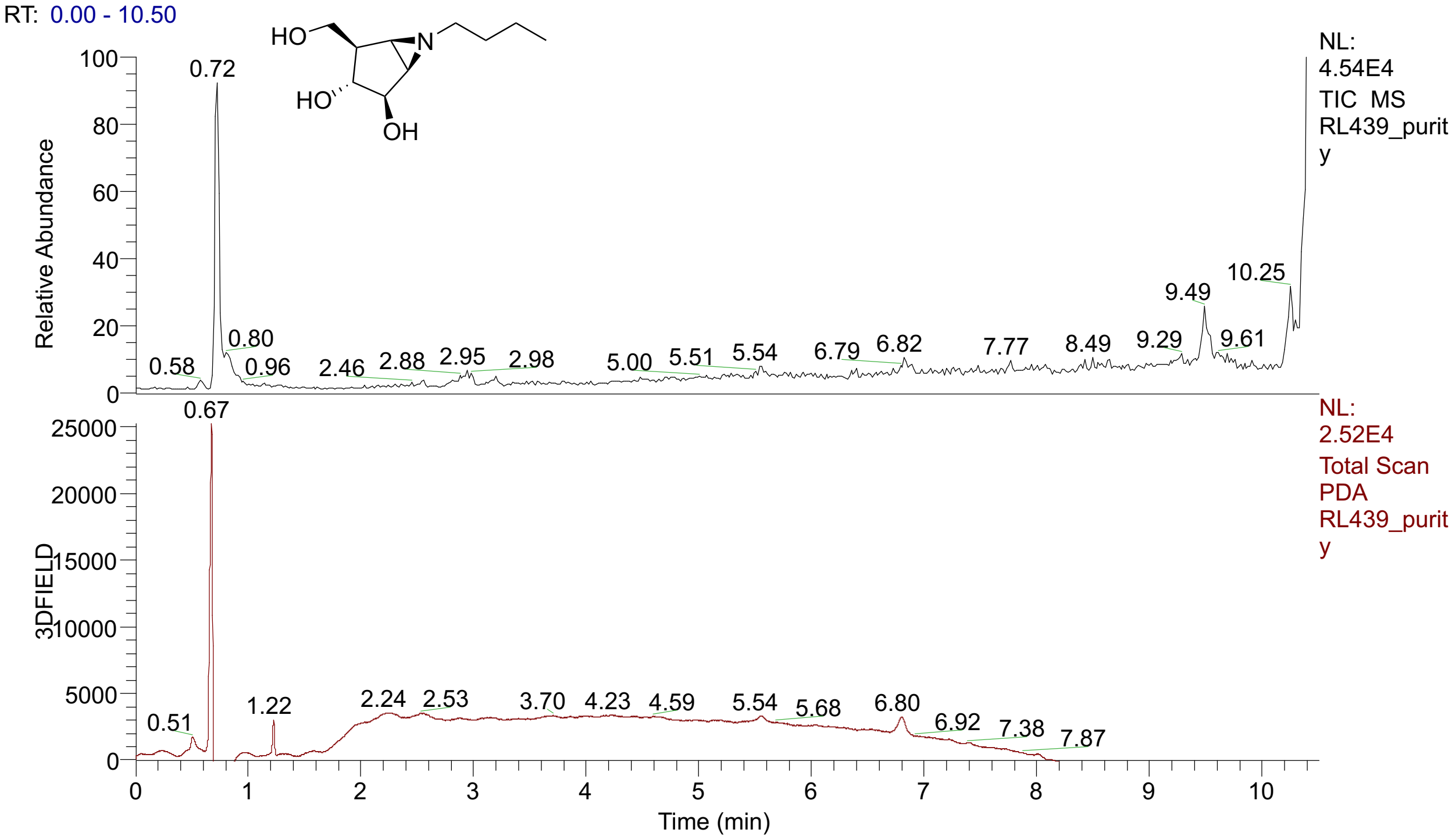

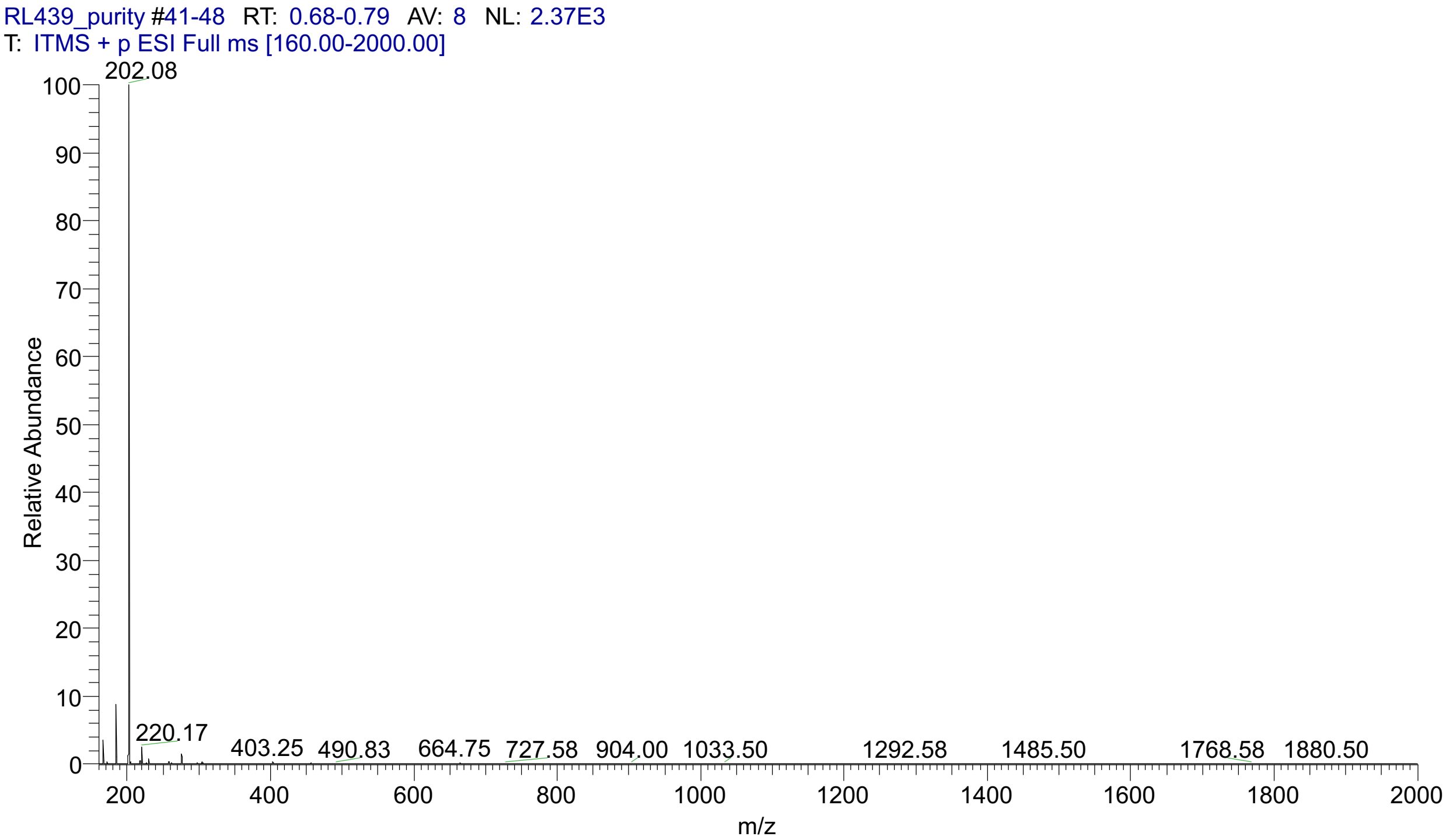

LC-MS spectrum of Compound **5** (Batch 1)

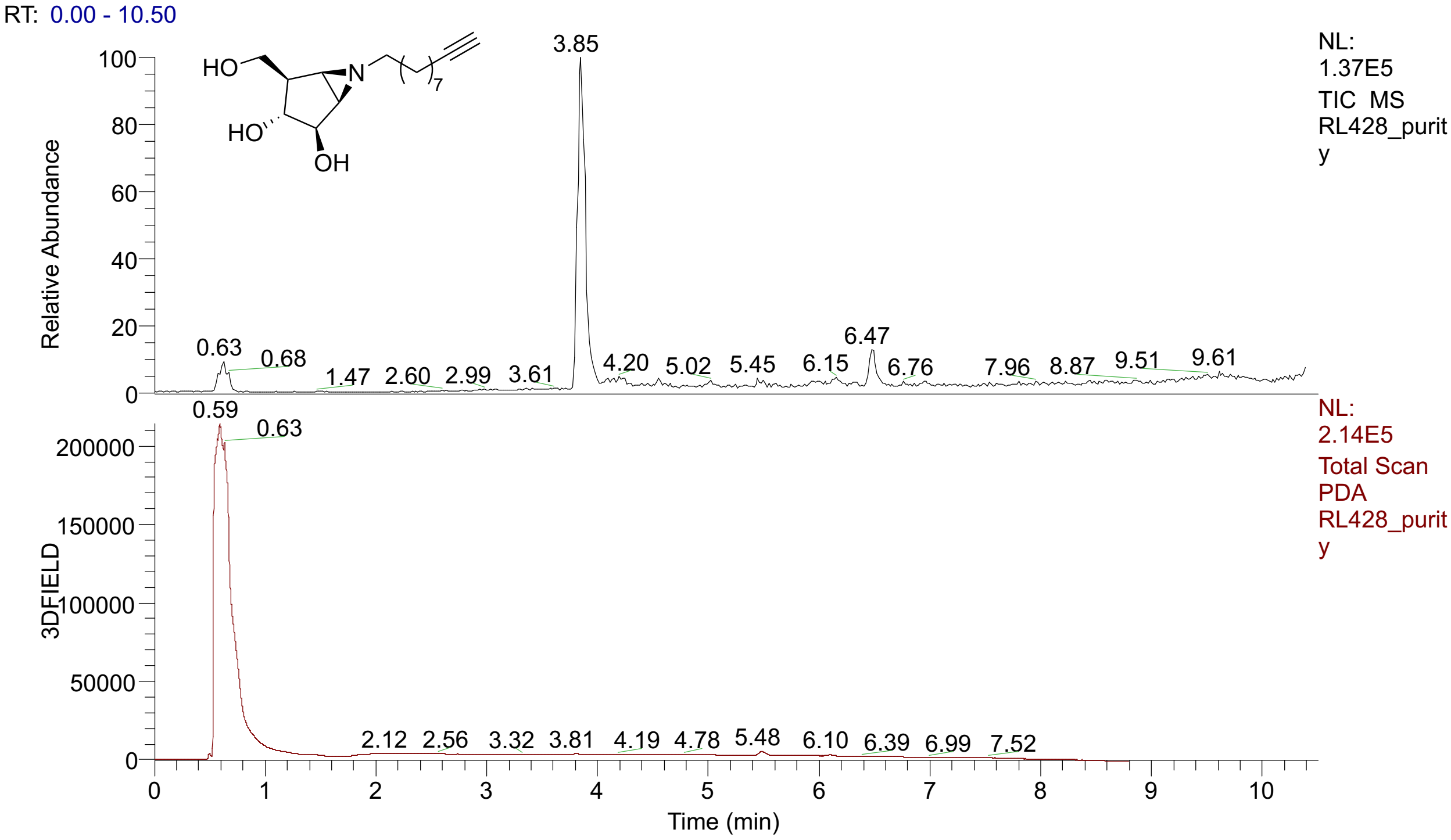

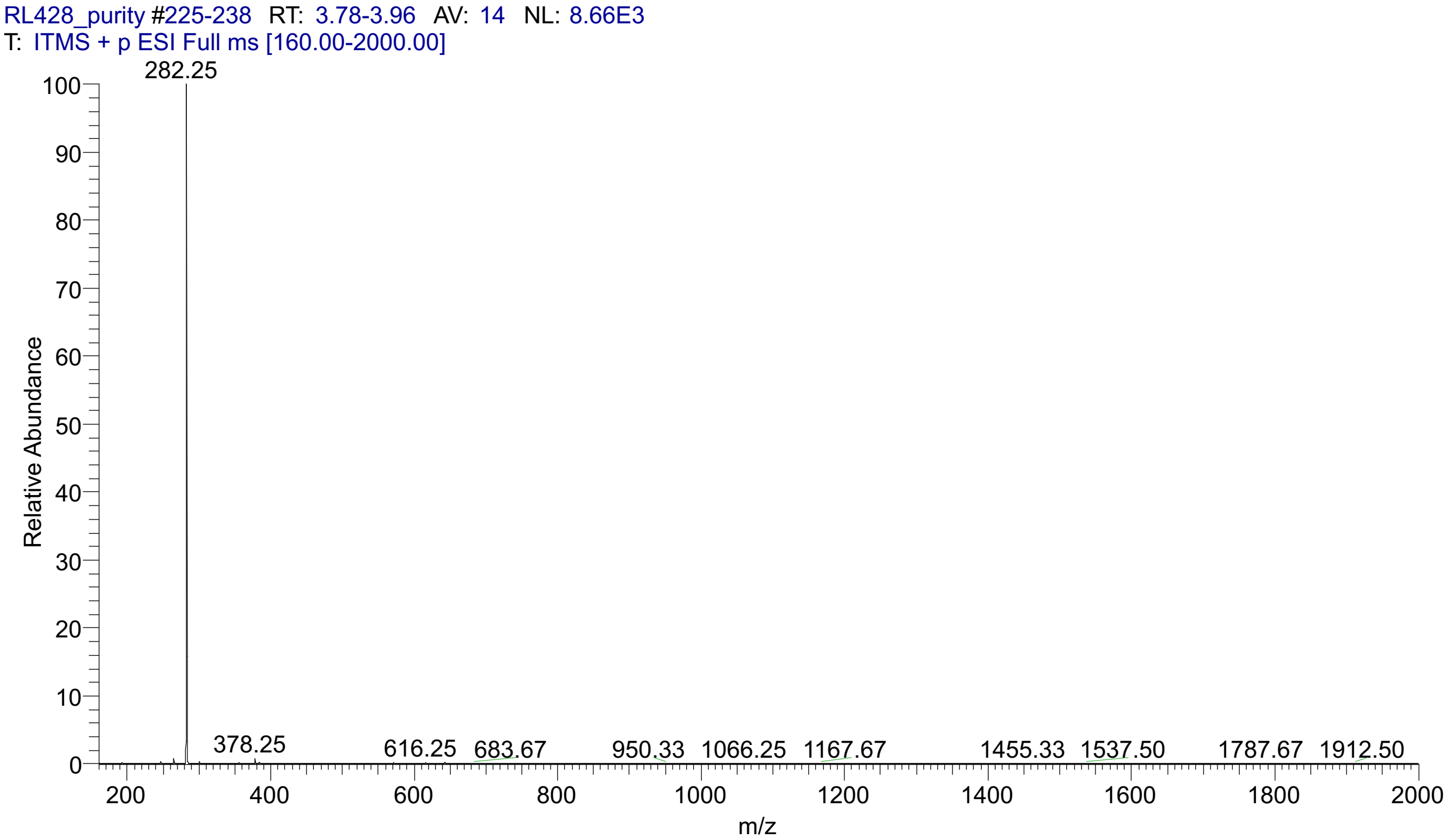

LC-MS spectrum of Compound **5** (Batch 2)

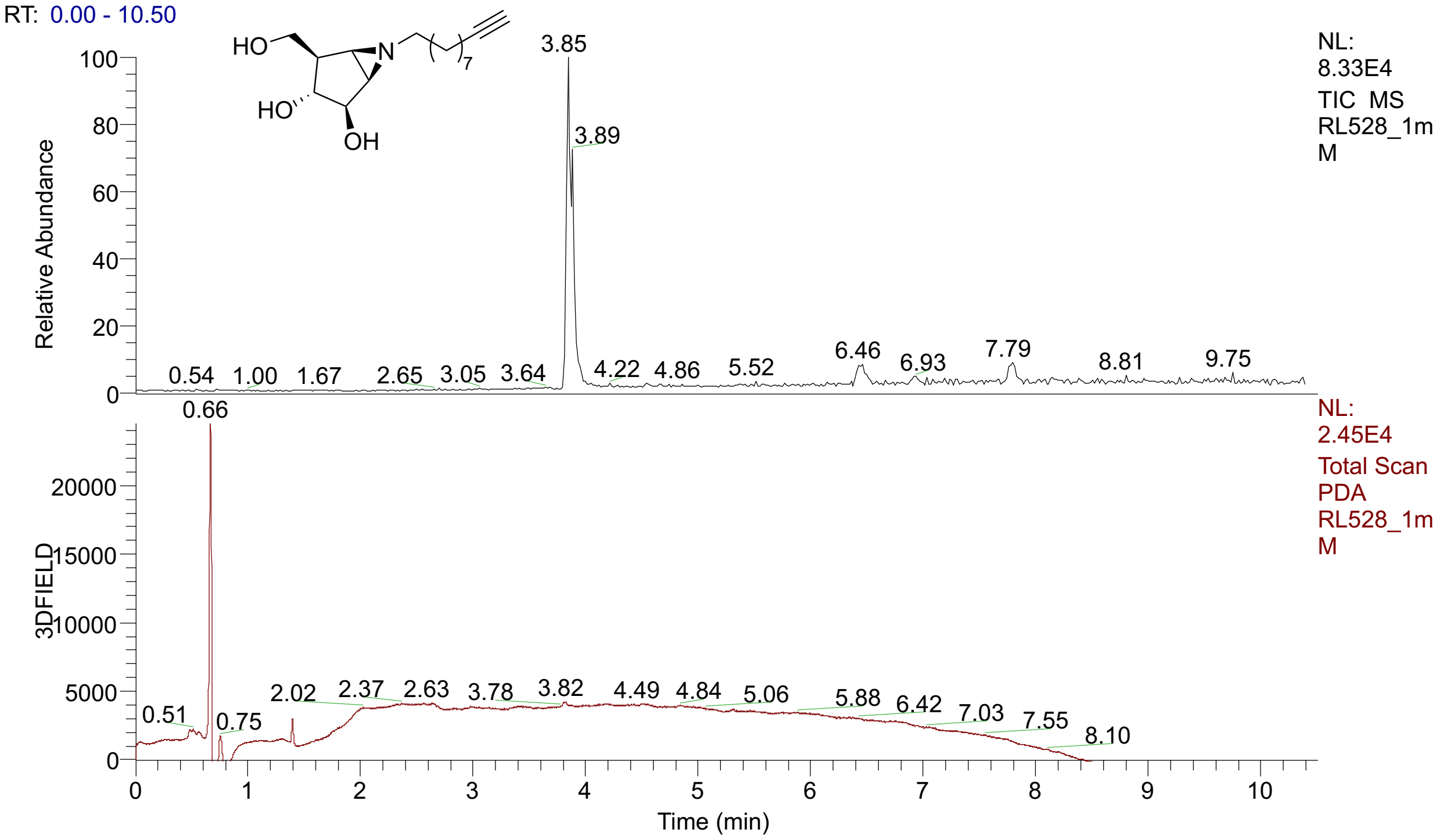

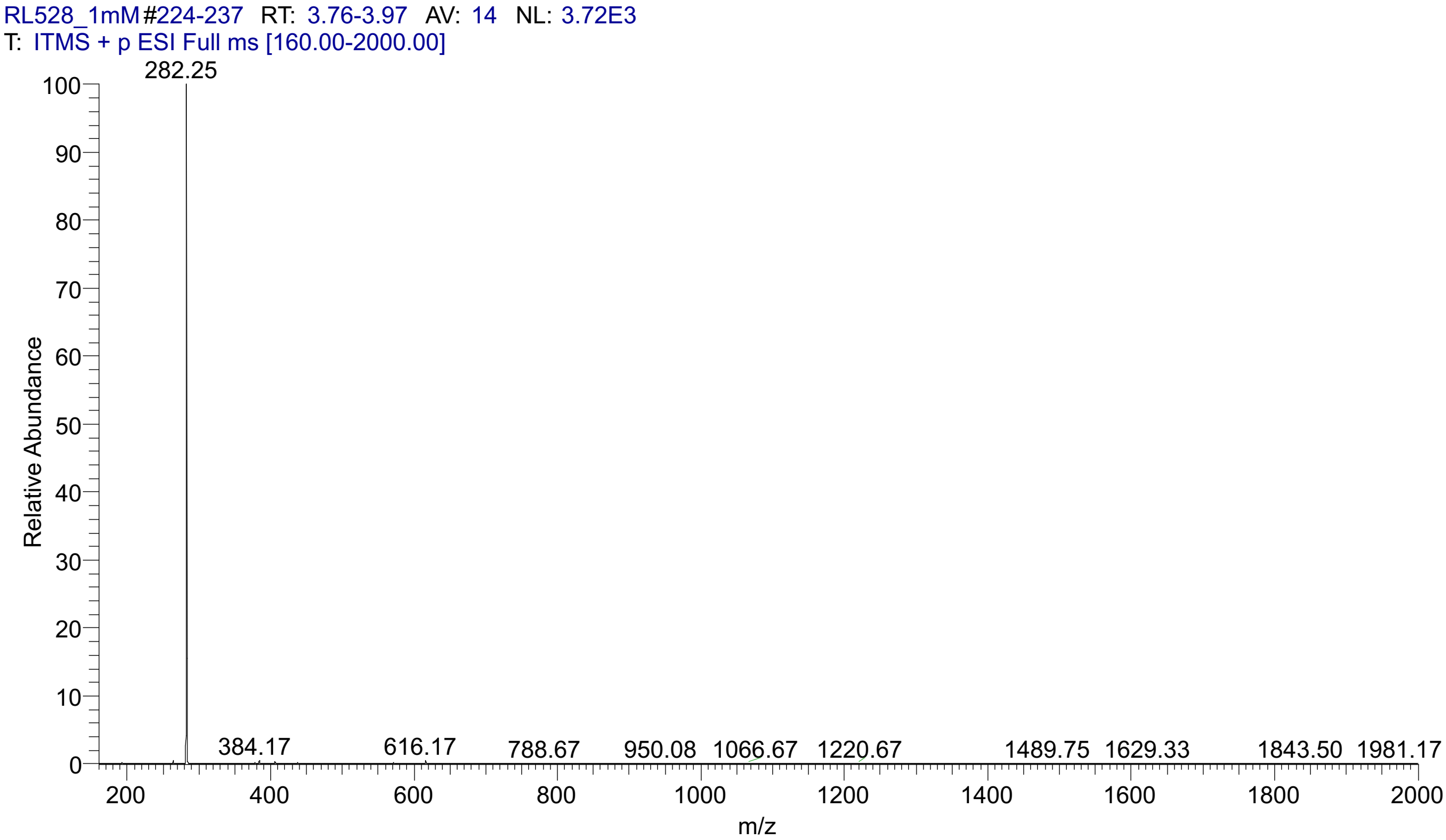

LC-MS spectrum of Compound **7**

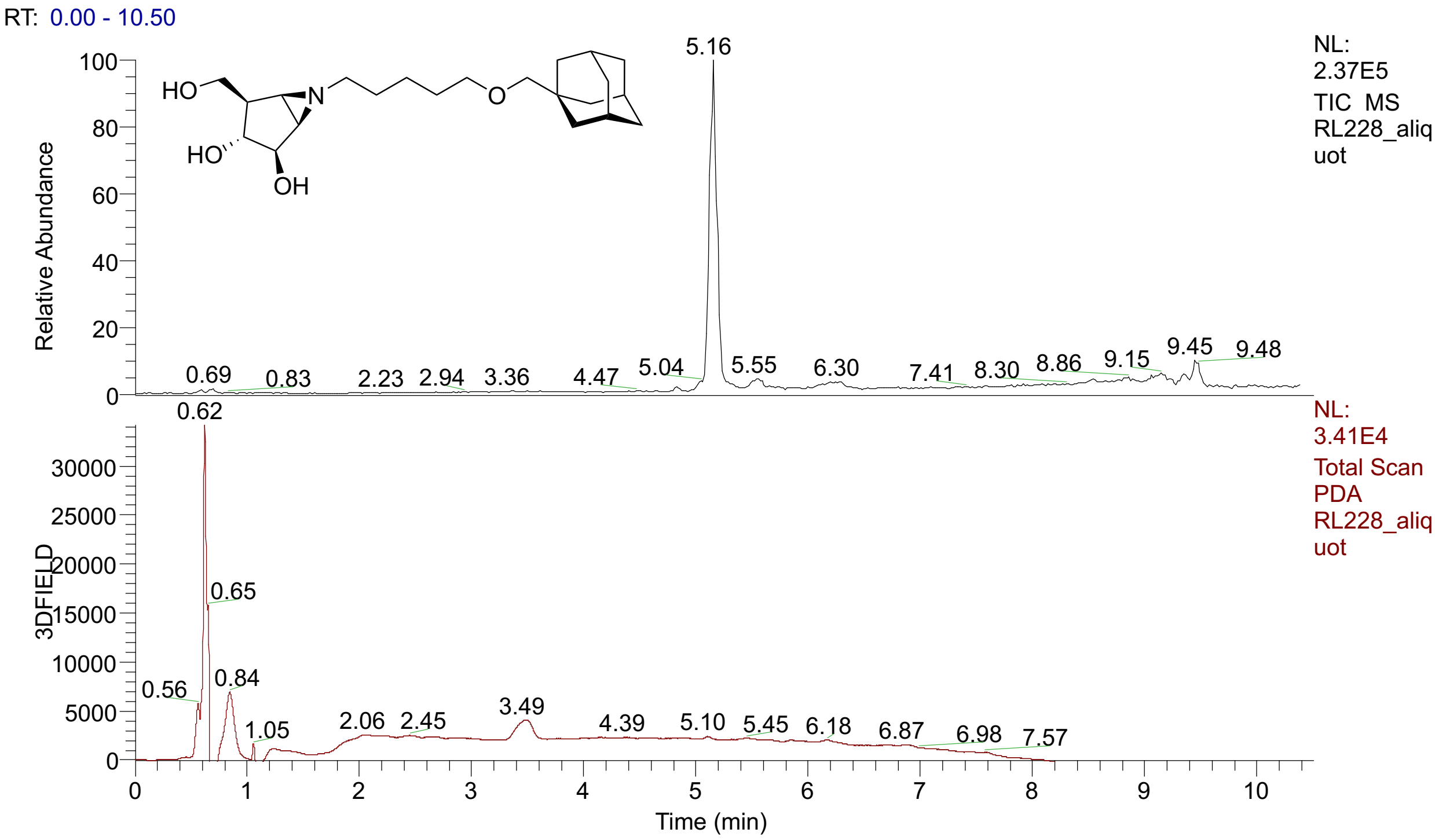

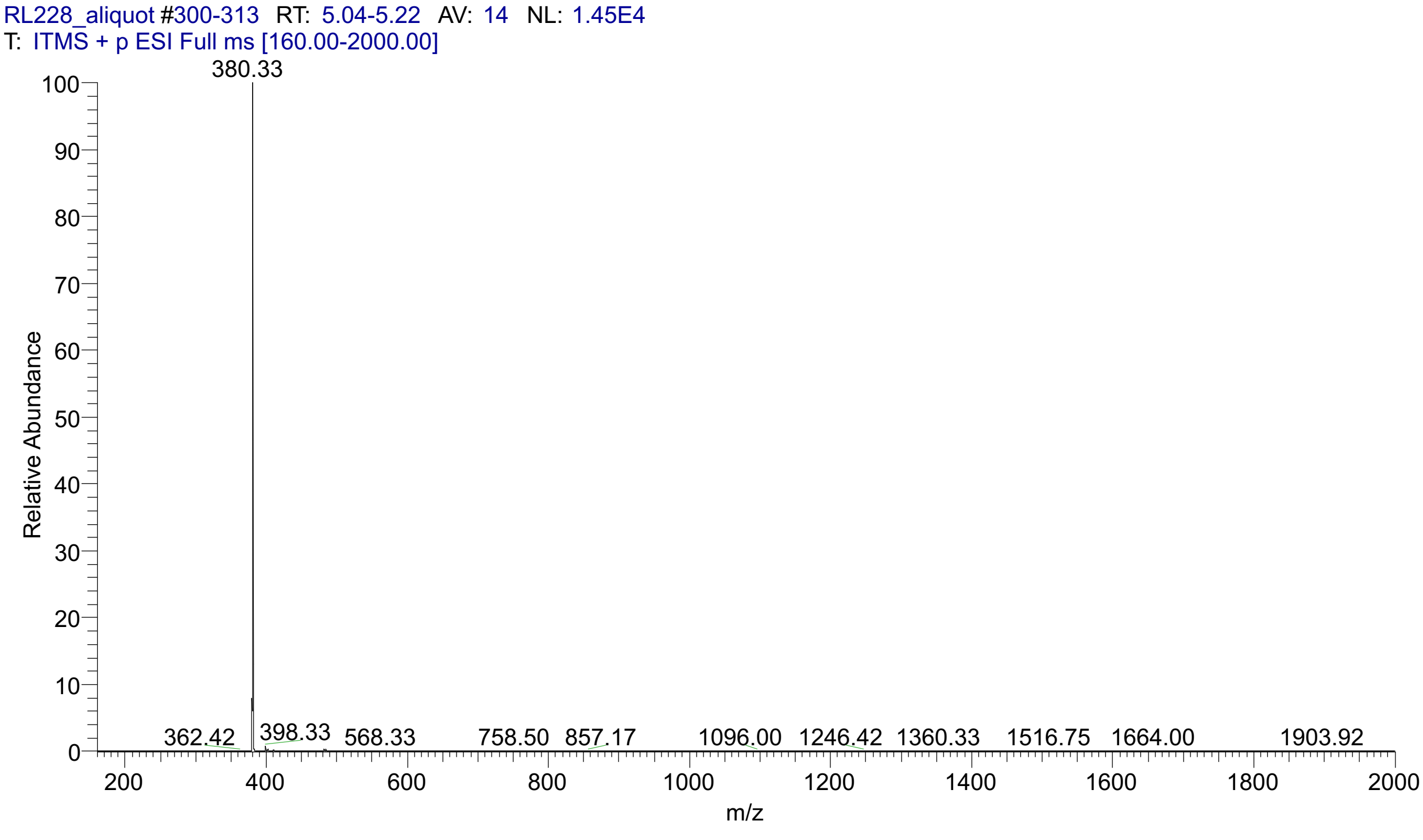

LC-MS spectrum of Compound **8**

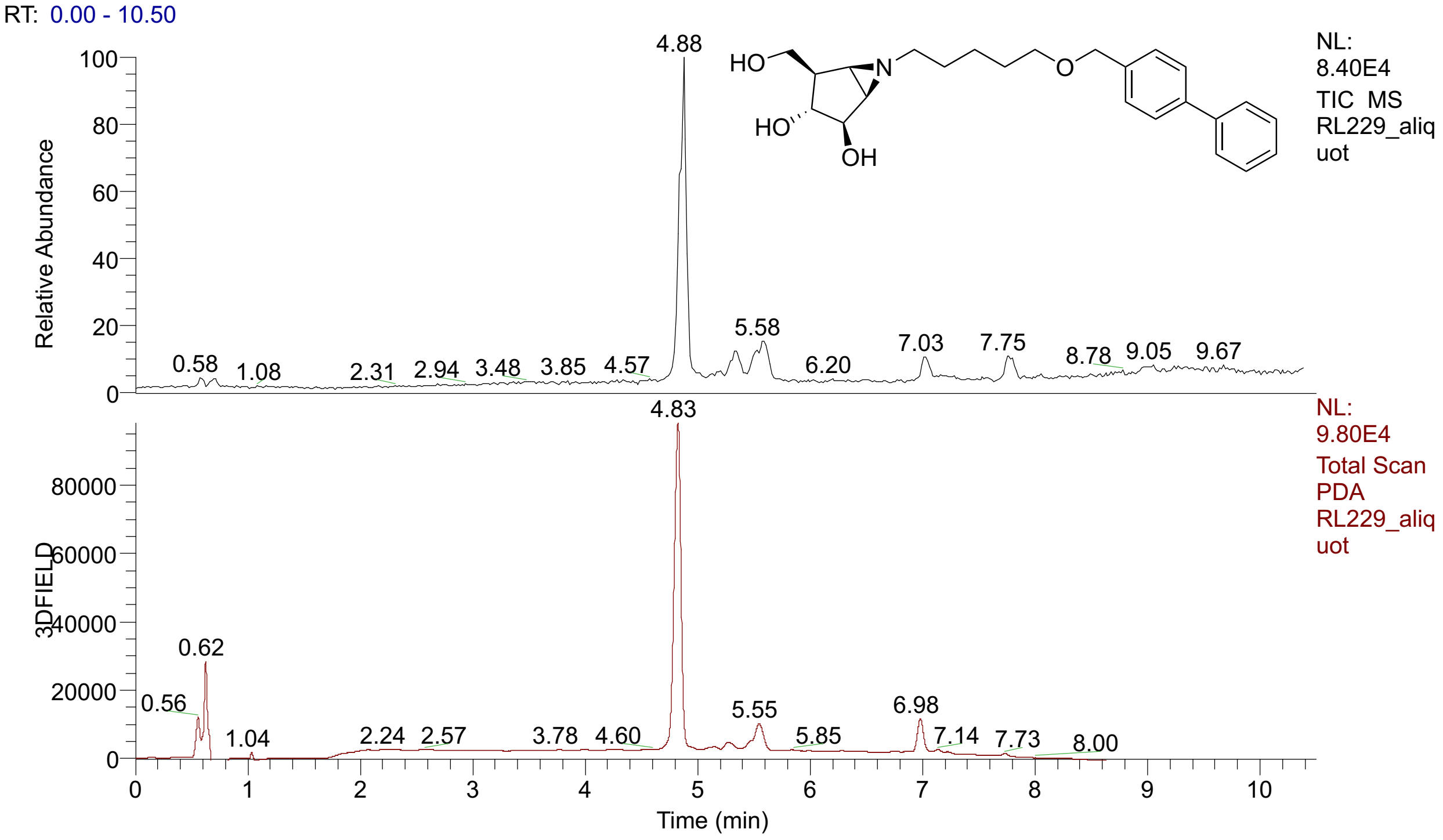

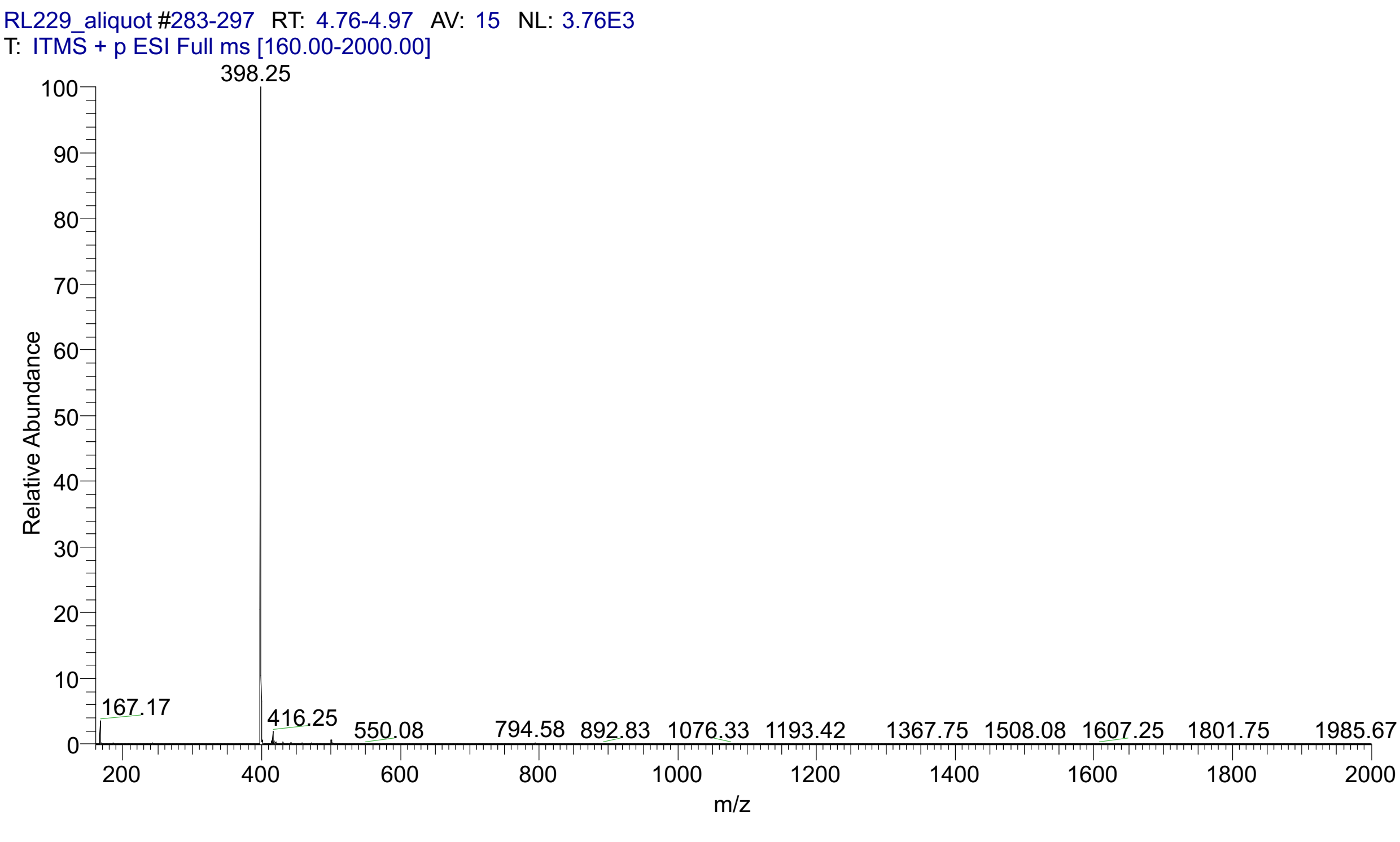

LC-MS spectrum of Compound **9**

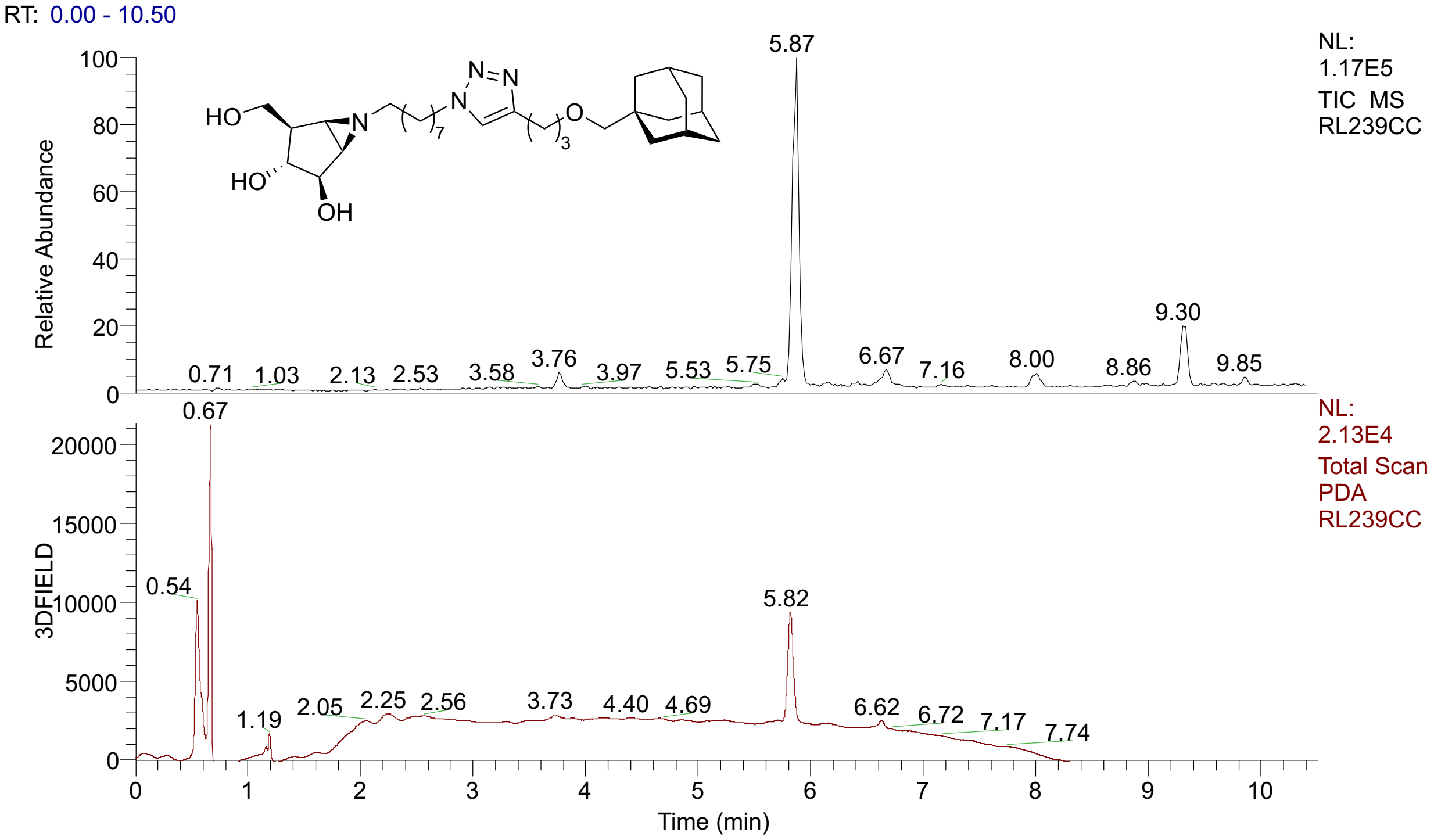

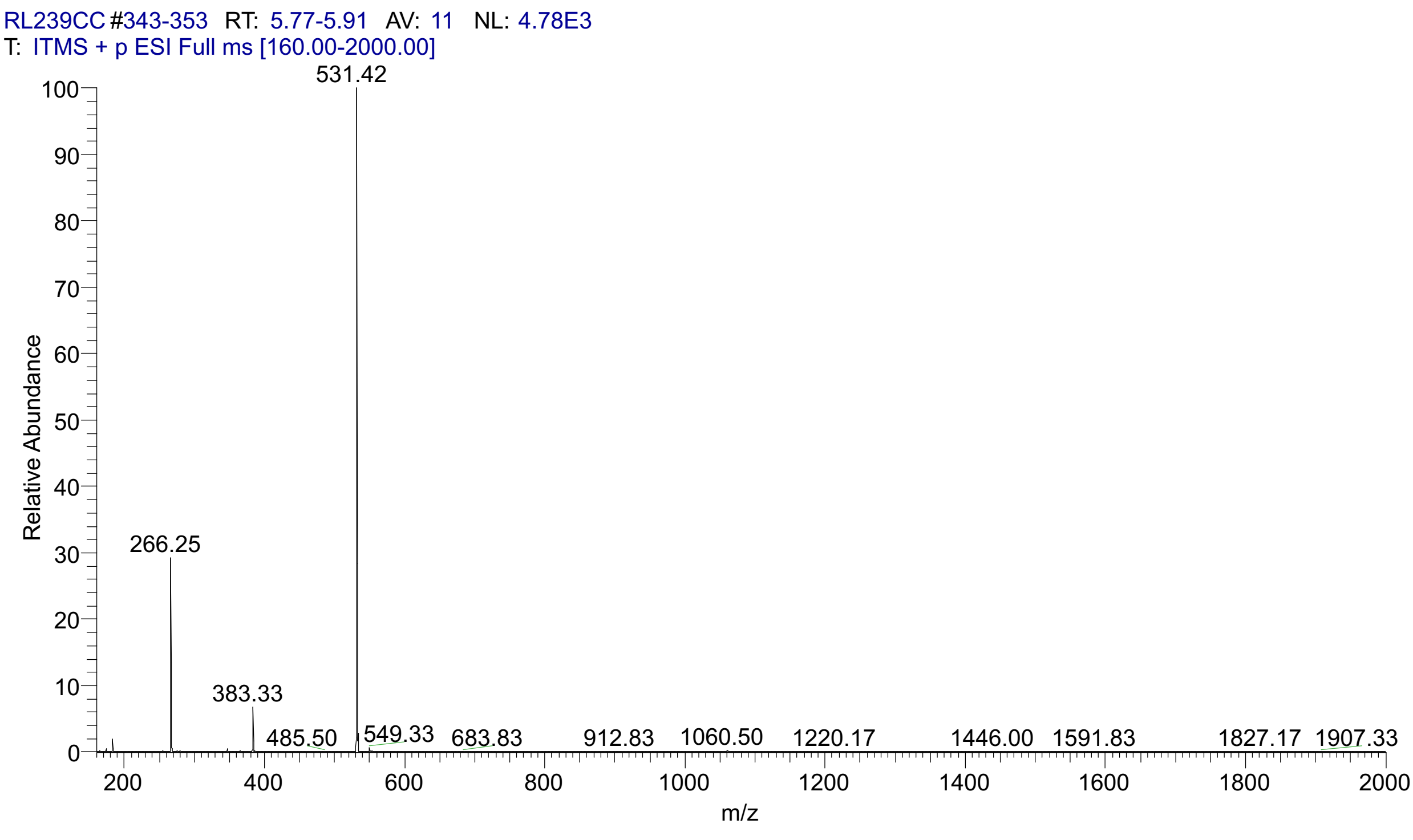

LC-MS spectrum of Compound **10**

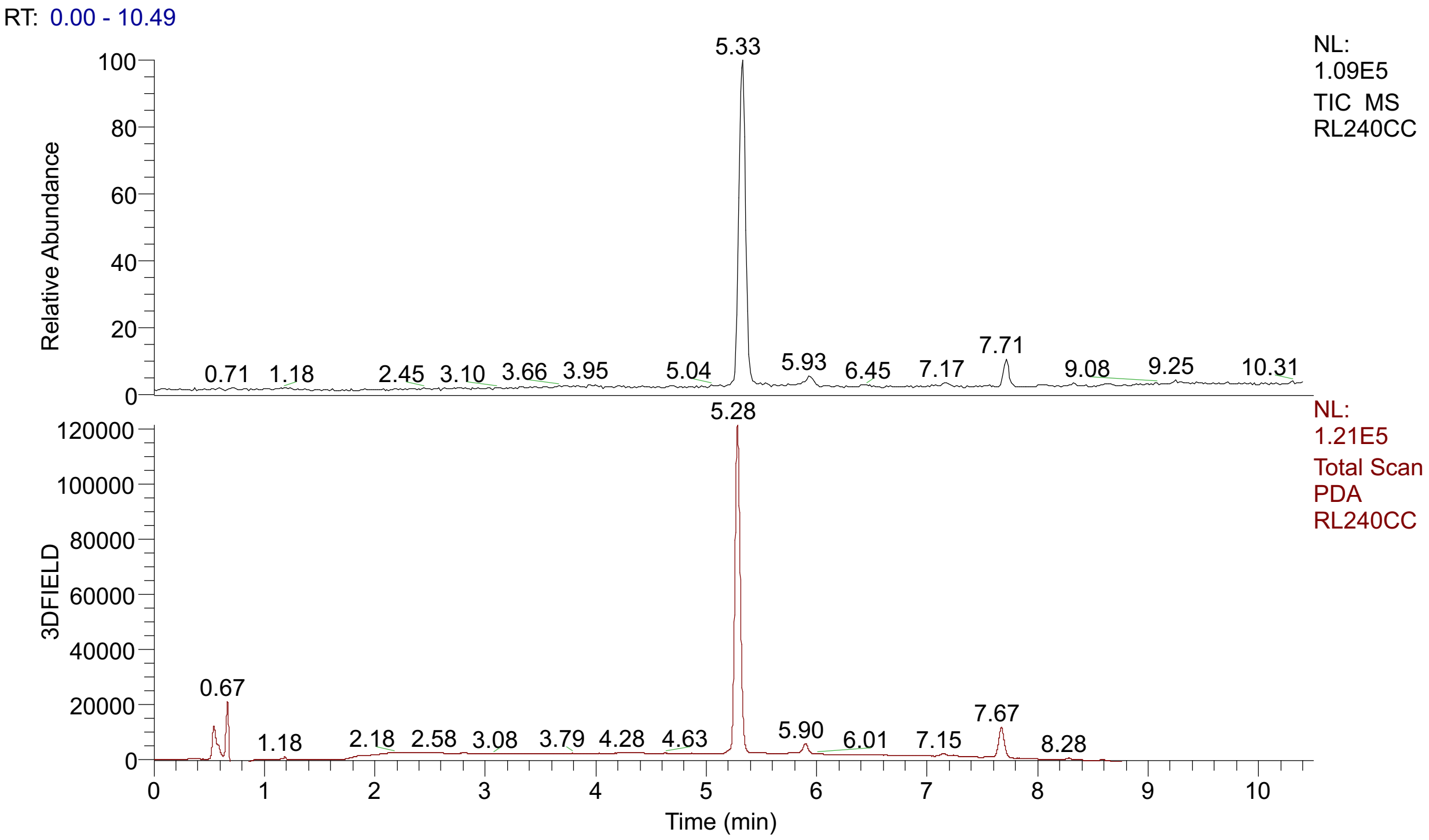

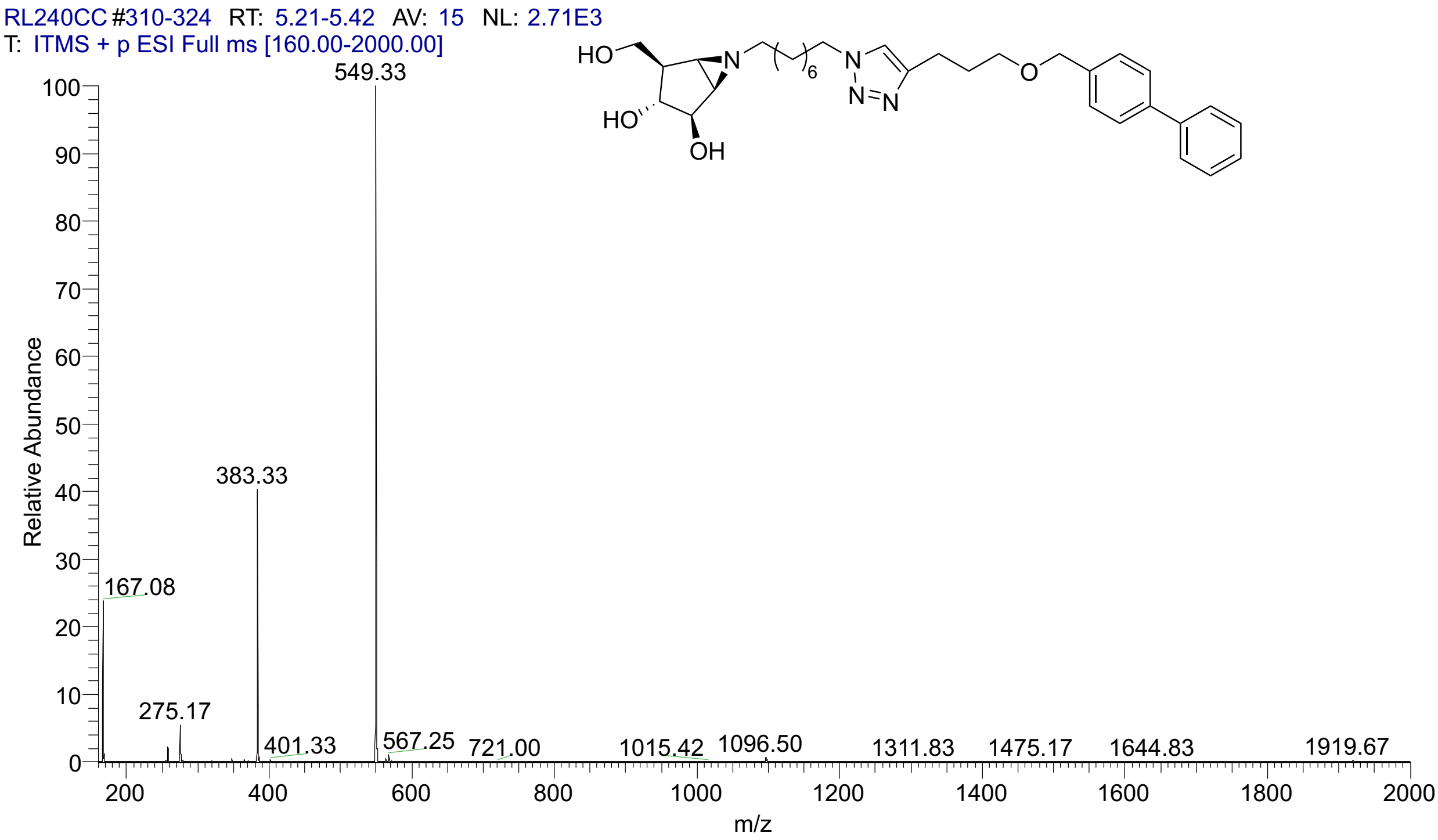

Supplement: Supplementary file 1 [file ja6c09754_si_001.pdf]
